# Dual antithrombotic therapy using potent antiplatelet inhibitors in atrial fibrillation and acute coronary syndrome: a randomized controlled trial

**DOI:** 10.1038/s41591-026-04629-7

**Published:** 2026-08-29

**Authors:** Konstantinos D. Rizas, Konstantinos Mourouzis, Dominik Rath, Christoph B. Olivier, Laura Elisa Villegas Sierra, Theofanis Korovesis, Amin Polzin, Amani Al Tawil, Reza Wakili, Lukas von Stuelpnagel, Katharina Mayer, Marion Janisch, Tanja Ulrich, Sarah Lang, Sebastian K. G. Maier, Florian Brattinger, Lorenz Bott-Flügel, Harilaos Bogossian, Konstantinos Iliodromitis, Luisa Freyer, Julius Steffen, Konstantin Stark, Elodie Eiffener, Lauren E. Sams, Tobias Petzold, Axel Linke, Felix M. Heidrich, Samuel Sossala, Saif Zako, Nikolaos Dagkonakis, Andreas Schäfer, Frank Edelmann, David M. Leistner, Mariya Maslarska, Fabian Knebel, Georg Nickenig, Blerim Luani, Tienush Rassaf, Hüseyin Ince, Ibrahim Akin, Micha T. Maeder, Meinrad Gawaz, Hans-Josef Feistritzer, Norbert Frey, Stefan Kääb, Riza Sahin, Matthias Pauschinger, Thomas Stiermaier, Ingo Eitel, Marc Kollum, Jan Sebastian Wolter, Michael Schreinlechner, Axel Bauer, Christoph Stellbrink, Ulf Landmesser, Paulus Kirchhof, Dirk Sibbing, Holger Thiele, Adnan Kastrati, Ulrich Mansmann, Steffen Massberg

**Affiliations:** 1 Clinic for Cardiology, HOCH Health Ostschweiz, Cantonal Hospital St. Gallen, St. Gallen, Switzerland; 2 Medizinische Klinik und Poliklinik I, LMU University Hospital, LMU Medizin, Ludwig Maximilians Universität München, Munich, Germany; 3 German Center for Cardiovascular Research (DZHK) Partner Site Munich, Munich, Germany; 4 Department of Cardiology and Angiology, University Hospital Tübingen, Eberhard Karls University Tübingen, Tübingen, Germany; 5 Department of Cardiology and Angiology, University Heart Center Freiburg-Bad Krozingen, Faculty of Medicine, University of Freiburg, Freiburg, Germany; 6 Department of Cardiology Pulmonology and Vascular Medicine, Cardiovascular Research Institute Düsseldorf (CARID), Medical Faculty of the Heinrich Heine University Düsseldorf, Düsseldorf, Germany; 7 Institute for Medical Information Processing, Biometry, and Epidemiology, Medical Faculty, Ludwig-Maximilians-University Munich, Munich, Germany; 8 Department of Cardiology/Vascular Medicine, University Heart and Vascular Center, Goethe University Frankfurt, Frankfurt, Germany; 9 German Center for Cardiovascular Research (DZHK) Partner Site Rhine-Main, Frankfurt, Germany; 10 Department of Cardiology, TUM University Hospital German Heart Center, Munich, Germany; 11 ISAResearch Zentrum, TUM University Hospital German Heart Center, Munich, Germany; 12 Department of Internal Medicine II, Cardiology, Intensive Care and Angiology, Klinikum St. Elisabeth Straubing, Medical Campus Lower Bavaria (MCN), Straubing, Germany; 13 Clinic for Cardiology and Pneumology, Klinikum Landkreis Erding, Erding, Germany; 14 Clinic for Cardiology and Electrophysiology, Evangelical Hospital Hagen-Haspe, Hagen, Germany; 15 School of Medicine, Witten/Herdecke University, Witten, Germany; 16 Institute of Environmental Medicine, Karolinska Institutet, Stockholm, Sweden; 17 Department of Cardiology, Angiology and Intensive Care Medicine - Deutsches Herzzentrum der Charité (DHZC), University Medical Center Berlin, Campus Benjamin Franklin, Berlin, Germany; 18 German Center for Cardiovascular Research (DZHK) Partner Site Berlin, Berlin, Germany; 19 Department of Internal Medicine and Cardiology, Heart Center Dresden University Hospital, Technische Universität Dresden, Dresden, Germany; 20 Medical Clinic I, Cardiology and Angiology, Gießen and Department of Cardiology, Campus Kerckhoff, Bad Nauheim, both Justus-Liebig-Universität, Gießen, Germany; 21 Cardio-Pulmonary Institute (CPI), Gießen, Germany; 22 Medizinische Klinik, Klinikum Konstanz, Konstanz, Germany; 23 Department of Cardiology and Angiology, Hannover Medical School, Hannover, Germany; 24 Department of Cardiology, Angiology and Intensive Care Medicine, Deutsches Herzzentrum der Charité (DHZC) − Medical Heart Centre of Charité and German Heart Institute Berlin, Berlin, Germany; 25 Charité − Universitätsmedizin Berlin, corporate member of Freie Universität Berlin and Humboldt Universität zu Berlin, Berlin, Germany; 26 Klinik für Innere Medizin mit Schwerpunkt Kardiologie, Sana Klinikum Lichtenberg, Berlin, Germany; 27 Department of Cardiology, Angiology and Pneumology − Internal Medicine II, University Hospital Bonn, Bonn, Germany; 28 Department of Cardiology, Klinikum Ingolstadt, Ingolstadt, Germany; 29 Department of Cardiology and Vascular Medicine, West German Heart and Vascular Center, University Hospital Essen, Essen, Germany; 30 Department of Cardiology, University of Rostock, Rostock, Germany; 31 Sektion Invasive Kardiologie, I. Medizinische Klinik, Universitätsmedizin Mannheim, Universität Heidelberg, Heidelberg, Germany; 32 Department of Cardiology, Heart Center Leipzig at Leipzig University, Leipzig, Germany; 33 Department of Cardiology, Angiology and Pulmology, University Hospital Heidelberg, Heidelberg, Germany; 34 Department of Cardiology, Klinikum Nürnberg Paracelsus Medical University Nuremberg, Nuremberg, Germany; 35 University of Luebeck, Heart Center − Department of Cardiology, Angiology and Intensive Care Medicine, University Hospital Schleswig-Holstein (UKSH) – Campus Luebeck, Luebeck, Germany; 36 German Center for Cardiovascular Research (DZHK) Partner Site North, Luebeck, Germany; 37 Hegau Bodensee Klinikum Singen, Gesundheitsverbund Landkreis Konstanz, Singen, Germany; 38 University Hospital for Internal Medicine III, Medical University of Innsbruck, Innsbruck, Austria; 39 Department of Cardiology and Intensive Care Medicine Bielefeld University, Klinikum Bielefeld Mitte, Bielefeld, Germany; 40 Department of Cardiology, University Heart and Vascular Center Hamburg, University Medical Center Hamburg-Eppendorf, Hamburg, Germany; 41 Institute of Cardiovascular Sciences, University of Birmingham, Birmingham, UK; 42 German Center for Cardiovascular Research (DZHK) Partner Site North, Hamburg, Germany; 43 Privatklinik Lauterbacher Mühle am Ostersee, Seeshaupt, Germany

**Keywords:** Myocardial infarction, Atrial fibrillation

## Abstract

The selection of the optimal antithrombotic regimen in patients with atrial fibrillation and acute coronary syndrome remains challenging. Previous trials have demonstrated that dual antithrombotic therapy (DAT), consisting of direct oral anticoagulants (DOACs) plus a P2Y12 inhibitor, reduces bleeding compared to a triple-therapy regimen using vitamin K antagonists. However, subsequent meta-analyses have suggested an increased risk of ischemic events with DAT, particularly within the first month of treatment. Clopidogrel has been the predominant P2Y12 inhibitor used across these studies, despite the risk of high on-treatment platelet reactivity when using this drug. In this study, we conducted an open-label, randomized controlled trial (EPIDAURUS) in patients with atrial fibrillation and acute coronary syndrome, designed to assess the efficacy and safety of a 1-month regimen of DOAC plus potent P2Y12 inhibitor (prasugrel or ticagrelor) compared to DOAC plus clopidogrel and in-hospital aspirin. The primary outcomes of the trial were an efficacy endpoint, recurrent ischemic events and safety endpoints, including death and major bleeding, which were evaluated 6 weeks after randomization using separate win−loss ratio analyses. The study was prematurely terminated after enrollment of 602 patients (154 female) of an expected 1,474 patients, owing to safety concerns raised by the Data and Safety Monitoring Board. Exploratory analyses of secondary safety endpoints, including bleeding type ≥2 and ≥3 according to the Bleeding Academic Research Consortium scale, revealed that, compared to clopidogrel and in-hospital aspirin, treatment with a potent P2Y12 inhibitor was associated with higher bleeding rates without a clear reduction in the risk of ischemic complications. These findings do not support the routine use of potent P2Y12 inhibitors in combination with DOACs in this patient population. ClinicalTrials.gov identifier: NCT04981041.

## Abstract (web summary)

As presented at the 2026 European Society of Cardiology Congress, use of direct oral anticoagulants together with potent P2Y12 inhibitors in patients with atrial fibrillation and acute coronary syndrome was associated with higher bleeding rates without evidence of reduced ischemic events, as compared to direct oral anticoagulants together with clopidogrel and aspirin.

## Main

The optimal antithrombotic regimen in patients with atrial fibrillation (AF) and acute coronary syndrome (ACS) undergoing percutaneous coronary intervention (PCI) remains challenging, owing to the required balance between prevention of ischemic events and avoidance of bleeding. Dual antiplatelet therapy (DAPT) with aspirin and a P2Y12 inhibitor has been proven to reduce recurrent cardiovascular events in patients undergoing PCI but provides limited protection against AF-related cardioembolic stroke^1^. Conversely, oral anticoagulants (OACs), the standard therapy for stroke prevention in AF, do not adequately address the increased risk of coronary thrombosis after PCI^2^. As a result, triple antithrombotic therapy (TAT), combining DAPT with an OAC, emerged in the past as the standard approach to provide protection against both coronary and cardioembolic events, albeit at the cost of increased bleeding.

Subsequent trials demonstrated that a DAT, consisting of DOACs plus P2Y12 inhibitor, reduces bleeding compared to vitamin K antagonist (VKA)-based TAT^3–6^. However, meta-analyses of these trials suggested a potentially higher risk of ischemic events, particularly stent thrombosis, with DAT^7^. A post hoc analysis of the AUGUSTUS trial showed that most stent thrombosis events occurred within the first month after the index PCI^8^.

Notably, clopidogrel was the predominant P2Y12 inhibitor used across these trials despite its variable antiplatelet effects and the risk of high on-treatment platelet reactivity. Approximately 30−40% of patients show low response to clopidogrel and are, therefore, inadequately protected against ischemic events early after PCI, particularly when presenting with ACS^9^. Consequently, clinical outcomes of patients with ACS undergoing PCI without AF have been substantially improved by the use of the potent P2Y12 inhibitors ticagrelor or prasugrel^10,11^.

However, there is little evidence on the use of potent P2Y12 inhibitors in combination with OACs in patients with AF and ACS. In a recently published retrospective analysis, including 2,259 patients with ACS and AF treated with DOAC and P2Y12 inhibitors, treatment with prasugrel or ticagrelor compared to clopidogrel was associated with significantly fewer major adverse cardiac events, without a significant increase in bleeding complications after 12 months of follow-up^12^. The 2020 European Society of Cardiology (ESC) guidelines for the management of ACS in patients without ST-segment elevation recommended considering the combination of a DOAC with prasugrel or ticagrelor in patients at high risk of stent thrombosis^13^. However, given that the supporting evidence for this strategy was available for fewer than 10% of patients enrolled in previous randomized trials, this recommendation was removed from the 2023 ESC guidelines for the management of ACS^14^, underscoring the need for dedicated prospective randomized controlled studies.

In the EPIDAURUS (Escalated Single Platelet Inhibition for One Month plus Direct Oral Anticoagulation in Patients with Atrial Fibrillation and Acute Coronary Syndrome Undergoing Percutaneous Coronary Intervention) trial, we, therefore, tested the hypothesis that, in patients with AF with ACS undergoing PCI, DAT using direct factor Xa inhibitors plus prasugrel or ticagrelor for 1 month followed by clopidogrel could reduce ischemic events without a relevant increase in bleeding complications compared to standard in-hospital TAT followed by DAT with direct factor Xa inhibitors plus clopidogrel.

## Results

### Study population

**Fig. 1:**
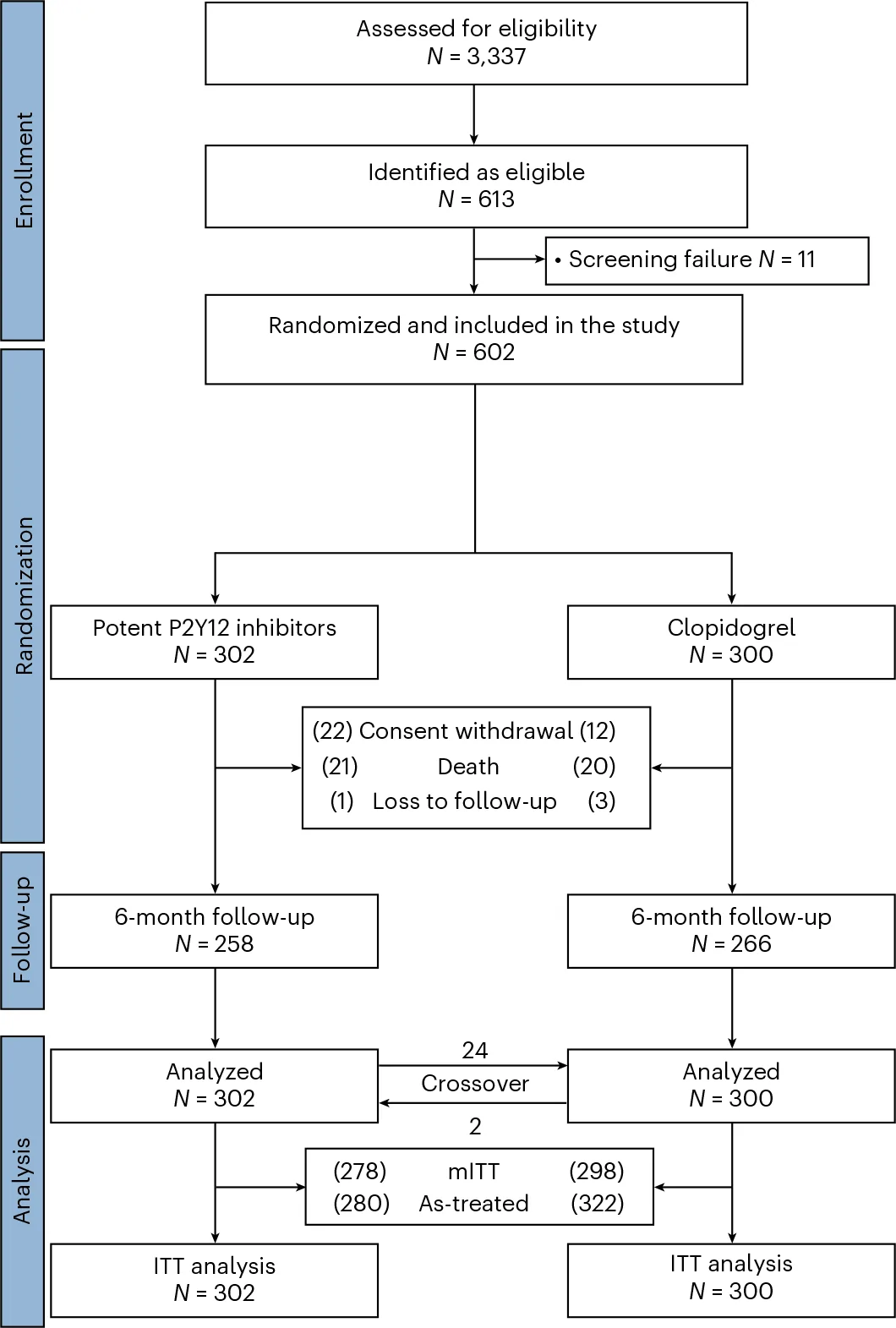
CONSORT flow diagram showing screening, enrollment, randomization and follow-up. The control group included patients treated with clopidogrel and in-hospital aspirin but is indicated here in a simplified fashion as clopidogrel.

Between December 2021 and September 2025, 3,337 patients were assessed for eligibility at 37 medical centers in Germany and Austria, and 602 patients were enrolled in the trial. The trial, which was planned to be completed by the end of 2027, was terminated early in September 2025 after a recommendation by the independent Data and Safety Monitoring Board (DSMB) at its third regular meeting. No formal interim analyses were planned, and the prespecified mortality stopping criterion had not been met. The DSMB recommendation was based on the totality of the accumulating data, including a low likelihood of superiority of the experimental intervention and concordant safety signals characterized by greater numbers of deaths, ischemic strokes and bleeding events in the experimental group. After consultation with the Steering Committee, the sponsor accepted the DSMB recommendation and immediately discontinued further recruitment. Details of the DSMB monitoring strategy and decision-making process, together with the DSMB charter, are provided in the DSMB section of the Supplementary Information and Supplementary Note 1. Of the 602 patients included in the trial (full analysis set (FAS)) at the time of trial termination, 302 were randomized to the experimental group and 300 were randomized to the control group. The rate of protocol deviations (24 versus 2) was higher in the experimental group than in the control group. The per-protocol set (PPS) consisted of 576 patients, including 278 in the experimental group and 298 in the control group (Fig. 1).

The median age was 78 (lower−upper quartile (Q1−Q3) 69−83) years, and 154 (26%) patients were female. The median CHA_2_DS_2_-VA score (a clinical score routinely used to predict the risk of ischemic stroke in patients with AF ranging from 0 to 8, with higher scores indicating a greater risk of stroke) was 4 (Q1−Q3 3−4). The median HAS-BLED score (a clinical score used to predict bleeding risk in patients with AF under treatment with OAC ranging from 0 to 9, with higher scores indicating a greater risk of bleeding) was 3 (Q1−Q3 3−3). Baseline demographic, clinical, angiographic and medication information is depicted in Table 1 (FAS) and in Supplementary Table 1 (PPS). Adherence to the assigned antithrombotic strategy was assessed at day 7 after randomization and is summarized in Extended Data Table 1. Among patients with available medication data, 96.2% of patients in the experimental group were receiving a potent P2Y12 inhibitor, whereas 99.3% of patients in the control group were receiving clopidogrel. Oral anticoagulation was paused or discontinued in 17 patients in the experimental group and in six patients in the control group; individual reasons are provided in Extended Data Table 2. According to the protocol, patients randomized to the experimental strategy were planned to transition from prasugrel or ticagrelor to clopidogrel between days 27 and 33 after randomization. Of the 277 patients in the experimental group who reached day 34, 260 (93.9%) had been successfully switched to clopidogrel according to the study protocol. Reasons for delayed or absent switching are provided in Extended Data Table 3.

**Table 1:** Demographic, clinical and angiographic characteristics stratified by randomization group in the FAS

Characteristics	Experimental group	Control group	Total
*N*	302	300	602
Demographic characteristics			
Age, years (Q1−Q3)	78 (71−83)	78 (68−83)	78 (69−83)
Female sex, *N*/total *N* (%)	87/302 (28.8%)	67/300 (22.3%)	154/602 (25.6%)
Diabetes mellitus, *N*/total *N* (%)	108/302 (35.8%)	95/300 (31.7%)	203/602 (33.7%)
Arterial hypertension, *N* total *N* (%)	260/299 (87.0%)	252/299 (84.3%)	512/598 (85.6%)
Hypercholesterolemia, *N*/total *N*	187/297 (63.0%)	194/295 (65.8%)	381/592 (64.4%)
Active smoker, *N*/total *N* (%)	52/302 (17.2%)	50/300 (16.7)	102/602 (16.9%)
Family history of CAD, *N*/total *N*	72/251 (28.7%)	76/253 (30.0%)	148/504 (29.4%)
Clinical characteristics			
Previous MI, *N*/total *N* (%)	80/302 (26.5%)	78/295 (26.4%)	158/597 (26.5%)
Previous PCI, *N*/total *N* (%)	127/299 (42.5%)	126/297 (42.4%)	253/596 (42.4%)
Previous CABG, *N*/total *N* (%)	24/302 (7.9%)	22/300 (7.3%)	46/602 (7.6%)
Previous stroke, *N*/total *N* (%)	11/302 (3.6%)	21/300 (7.0%)	32/602 (5.3%)
AF, *N*/total *N* (%)			
New diagnosis	79 /294 (26.9%)	79/295 (26.8%)	158/589 (26.8%)
Paroxysmal	135/294 (45.9%)	132/295 (44.7%)	267/589 (45.3%)
Persistent	47/294 (16.0%)	60/295 (20.3%)	107/589 (18.2%)
Longstanding persistent	3/294 (1.0%)	0/295 (0.0%)	3/589 (0.0%)
Permanent	30/294 (10.2%)	24/295 (8.1%)	54/589 (9.2%)
MI type, *N*/total *N* (%)			
STEMI	59/301 (19.6%)	60/300 (20.0%)	119/601 (19.8%)
NSTEMI	242/301 (80.4%)	240/300 (80.0%)	482/601 (80.2%)
LVEF, *N*/total *N* (%)			
≥50%	147/294 (50.0%)	148/294 (50.3%)	295/588 (50.2%)
40−49%	75/294 (25.5%)	77/294 (26.2%)	152/588 (25.8%)
30−39%	46/294 (15.6%)	41/294 (13.9%)	87/588 (14.8%)
<30%	26/294 (8.8%)	28/294 (9.5%)	54/588 (9.2%)
NYHA, *N*/total *N* (%)			
I	136/300 (45.3%)	142/300 (47.3%)	278/600 (46.3%)
II	52/300 (17.3%)	55/300 (18.3%)	107/600 (17.8%)
III	66/300 (22.0%)	74/300 (24.7%)	140/600 (23.3%)
IV	46/300 (15.3%)	29/300 (9.7%)	75/600 (12.5%)
CCS, *N*/total *N* (%)			
0	107/299 (35.8 %)	89/297 (30.0%)	196/596 (32.9%)
I	10/299 (3.3%)	15/297 (5.1%)	25/596 (4.2%)
II	30/299 (10.0%)	37/297 (12.5%)	67/596 (11.2%)
III	63/299 (21.1%)	40/297 (13.5%)	103/596 (17.3%)
IV	89/299 (29.8%)	116/297 (39.1%)	205/596 (34.4%)
Bleeding predisposition, *N*/total *N* (%)	15/300 (5.0)	17/297 (5.7%)	32/597 (5.3%)
CHA_2_DS_2_-VA score, score (Q1− Q3)	4 (3−5)	4 (3−4)	4 (3−4)
HAS-BLED score, score (Q1−Q3)	3 (3−3)	3 (2−3)	3 (3−3)
PAOD, *N*/total *N* (%)	32/300 (10.7%)	30/299 (10.0%)	62/599 (10.4%)
Chronic renal failure, *N*/total *N* (%)	71/302 (23.5%)	81/300 (27.0%)	152/602 (25.2%)
Diseased vessels, *N*/total *N* (%)			
1	78/302 (25.8%)	74/300 (24.7%)	152/602 (25.2%)
2	78/302 (25.8%)	82/300 (27.3%)	160/602 (26.6%)
3	146/302 (48.3%)	144/300 (48.0%)	290/602 (48.2%)
Interventional characteristics			
Access site, *N*/total *N* (%)			
Radial	221/302 (73.2%)	229/300 (76.3%)	450/602 (74.8%)
Femoral	78/302 (25.8%)	68/300 (22.7%)	146/602 (24.3%)
Other	3/302 (1.0%)	3/300 (1.0%)	6/602 (1.0%)
Culprit lesion, *N*/total *N* (%)^a^			
LM	16/302 (5.3%)	15/300 (5.0%)	31/602 (5.1%)
LAD	120/302 (39.7%)	128/300 (42.7%)	248/602 (41.2%)
LCx	78/302 (25.8%)	79/300 (26.3%)	157/602 (26.1%)
RCA	99/302 (32.8%)	91/300 (30.3%)	190/602 (31.6%)
Bypass graft	4/302 (1.3%)	2/300 (0.7%)	6/602 (1.0%)
TIMI III flow after procedure, *N*/total *N* (%)	285/302 (94.4%)	284/300 (94.7%)	569/602 (94.5%)
Ostial PCI, *N*/total *N* (%)	48/302 (15.9%)	55/300 (18.3%)	103/602 (17.1%)
Bifurcation PCI, *N*/total *N* (%)	44/302 (14.6%)	60/300 (20.0%)	104/602 (17.3%)
Multivessel PCI, *N*/total *N* (%)	54/302 (17.9%)	57/300 (19.0%)	111/602 (18.4%)
Number of stents, median (Q1−Q3)	2 (1−2)	1 (1−2)	1 (1−2)
Length of stents, mm (Q1−Q3)	28 (18−40)	28 (18−44)	28 (18−43)
Medication			
ASA on admission	59/299 (19.7%)	71/297 (23.9%)	130/596 (21.8%)
P2Y12 inhibitors on admission	34/301 (11.3%)	46/297 (15.5%)	80/598 (13.4%)
OAC on admission	181/300 (60.3%)	184/298 (61.7%)	365/598 (61.0%)

^a^Some patients presented with more than one culprit lesions.

Only available data have been used for calculation of proportions.

ASA, aspirin; CAD, coronary artery disease; CCS, Canadian Cardiovascular Society angina score; CHA_2_DS_2_-VA score, congestive heart failure, hypertension, age ≥75 (doubled), diabetes, stroke (doubled), vascular disease, age 65−74; HAS-BLED score, hypertension, abnormal renal and/or liver function, stroke, bleeding history or predisposition, labile INR, elderly (age >65 years), drugs; LAD, left anterior descending artery; LCx, left circumflex artery; LVEF, left ventricular ejection fraction; LM, left main coronary artery; NYHA, New York Heart Association classification; NSTEMI, non-ST elevation myocardial infarction; MI, myocardial infarction; PAOD, peripheral arterial occlusive disease; RCA, right coronary artery; TIMI, thrombolysis in myocardial infarction.

### Primary outcomes

**Fig. 2:**
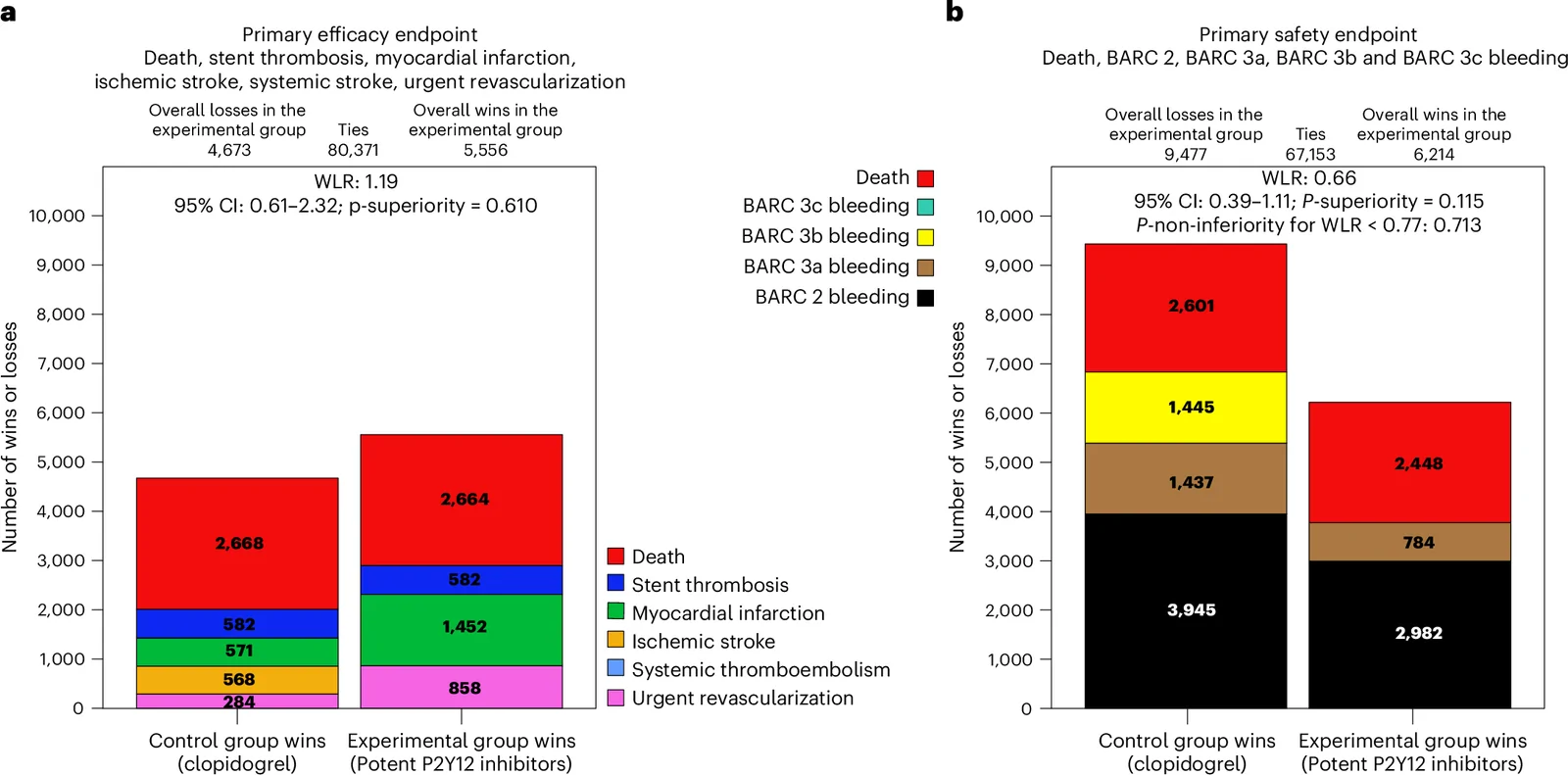
Primary efficacy and safety outcomes. **a**, WLR and its composition in wins and losses for the primary efficacy endpoint, as stratified by the randomization group (treatment with DOACs and potent P2Y12 inhibitors (experimental group) versus DOAs, clopidogrel and in-hospital aspirin (control group)). The primary efficacy endpoint was defined as the hierarchical evaluation of death, stent thrombosis, myocardial infarction, ischemic stroke, systemic thromboembolism and urgent revascularization and was evaluated using the ITT principle 6 weeks after randomization. No episodes of systemic thromboembolism were noted in either group. **b**, The WLR and its composition for the primary safety endpoint, as stratified by randomization group. The primary safety endpoint was defined as the hierarchical evaluation of death and BARC 3c, BARC 3b, BARC 3a and BARC 2 bleeding and was evaluated 6 weeks after randomization using an mITT principle in the PPS. No BARC 3c bleeding was noted in either group. BARC 3b bleeding was noted only in the experimental group and, therefore, appears only as losses. The WLR, its CI and two-sided superiority *P* values were calculated using the Finkelstein and Schoenfeld method, whereas one-sided non-inferiority *P* values were calculated using bootstrapping and 1,000 iterations. No adjustments were made for multiple comparisons. The control group included clopidogrel and in-hospital aspirin but is indicated here a simplified fashion as clopidogrel.

Based on a total of 90,600 (302 × 300) paired comparisons between both groups, 5,556 wins occurred in the experimental group and 4,673 wins occurred in the control group (losses), leaving a total of 80,371 ties. The win−loss ratio (WLR) 6 weeks after randomization for the primary efficacy endpoint, defined as the hierarchic composite of death, stent thrombosis, myocardial infarction, ischemic stroke, systemic thromboembolism and urgent revascularization, was 1.19 (95% confidence interval (CI): 0.61−2.32, *P* = 0.610; Fig. 2). For the primary safety endpoint 6 weeks after randomization, defined as the hierarchic composite of death and Bleeding Academic Research Consortium (BARC) 3c, BARC 3b, BARC 3a and BARC 2 bleeding, there have been 82,844 (278 × 298) paired comparisons, resulting in 6,214 wins, 9,477 losses and 67,153 ties. This corresponds to a WLR of 0.66 (95% CI: 0.39−1.11, *P-*superiority for the control group = 0.115, *P* for non-inferiority = 0.713; Fig. 2).

### Secondary outcomes

**Fig. 3:**
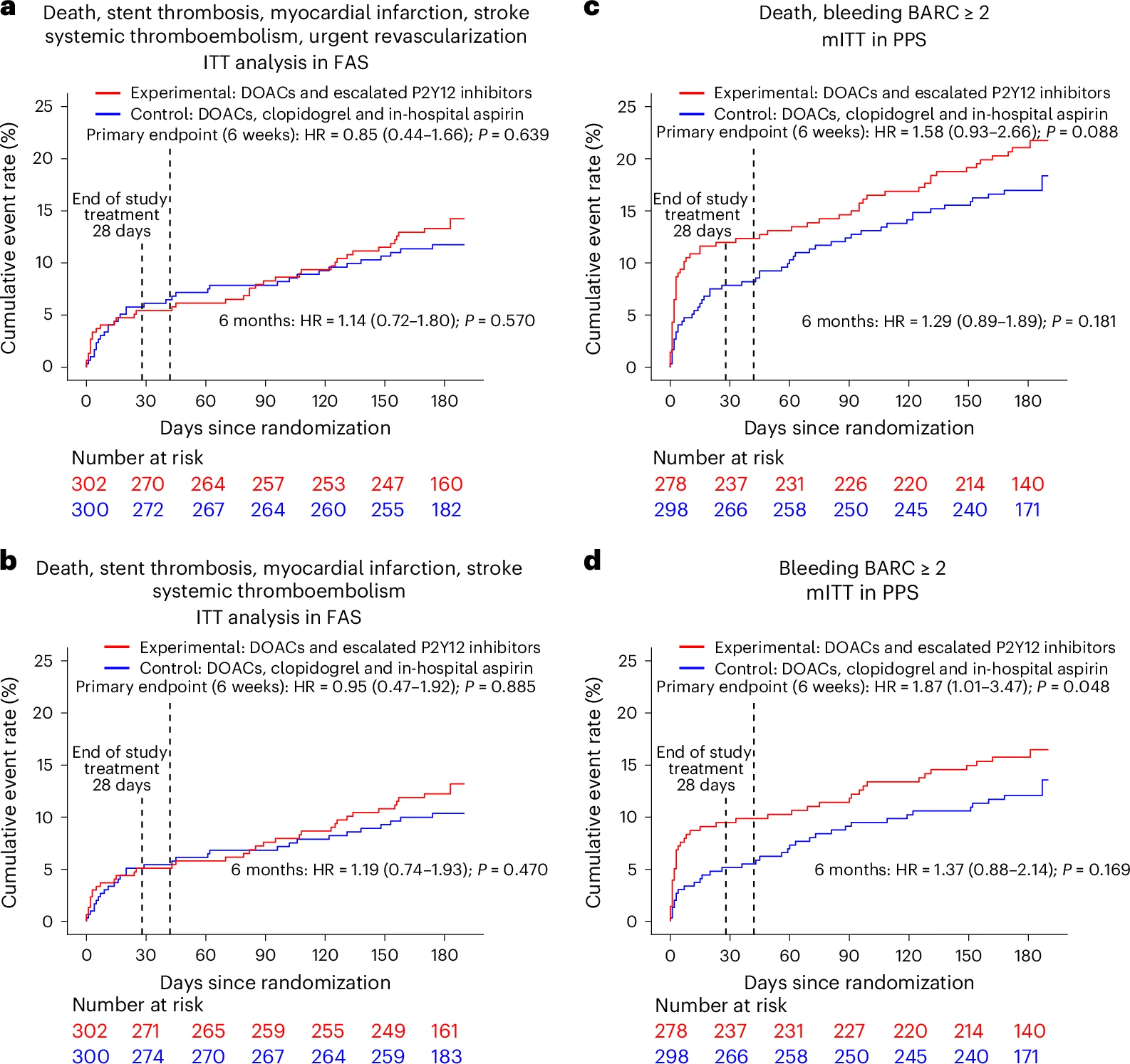
Kaplan−Meier analyses for efficacy and safety endpoints. **a**−**d**, Kaplan−Meier curves for efficacy (**a**,**b**) and safety endpoints (**c**,**d**), as stratified by treatment with DOACs and potent P2Y12 inhibitors (experimental group) versus treatment with DOACs, clopidogrel and in-hospital aspirin (control group). Efficacy endpoints were evaluated using an ITT analysis in the FAS, and safety endpoints were evaluated using an mITT analysis in the PPS. HRs and their corresponding two-sided *P* values were calculated using Cox regression analysis. No adjustments were made for multiple comparisons.

**Fig. 4:**
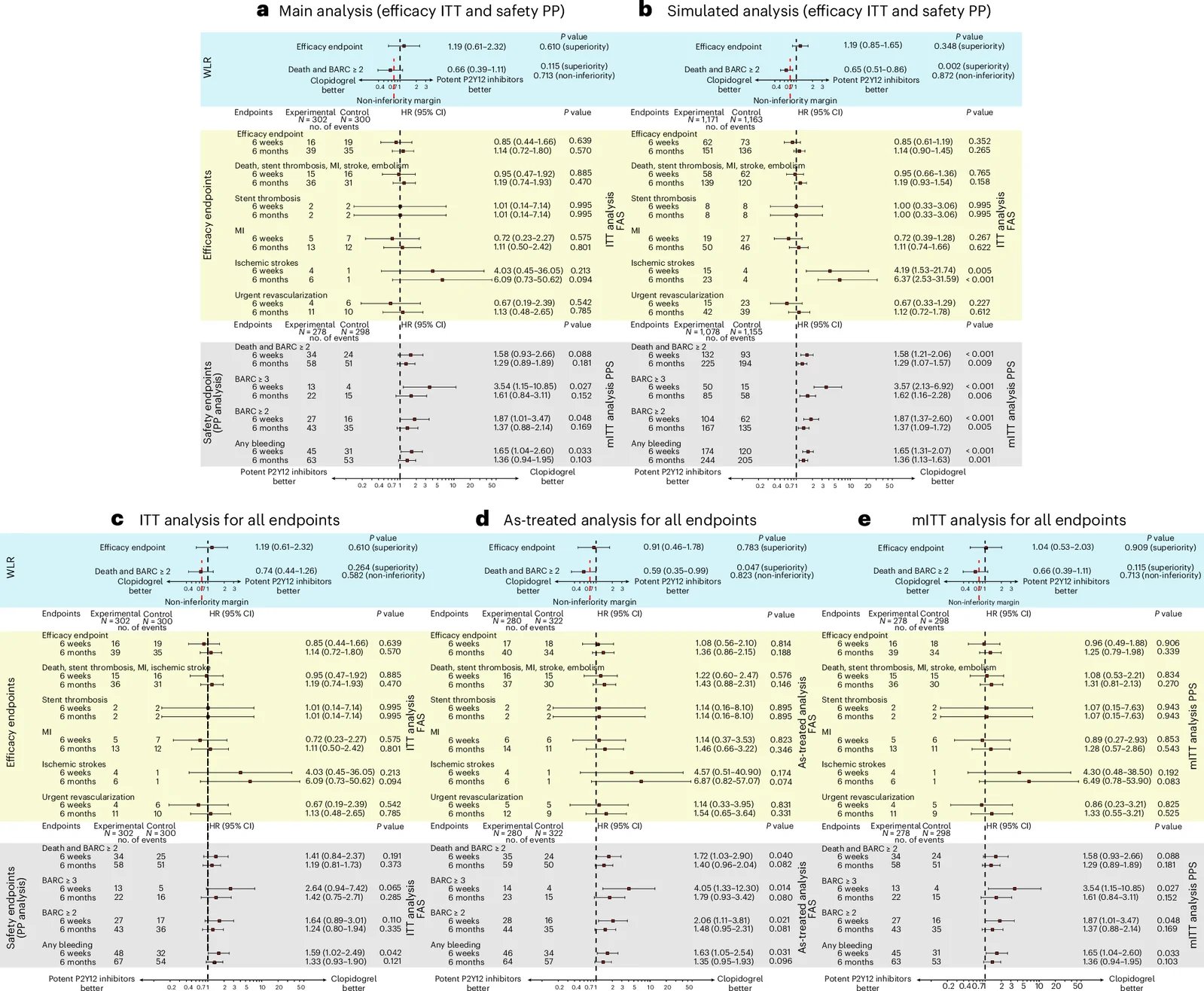
Sensitivity analyses. **a**, The in-protocol predefined analysis is shown, which was performed using an ITT analysis for efficacy endpoints and an mITT analysis for safety endpoints. The WLR, its CI and two-sided superiority *P* values were calculated using the Finkelstein and Schoenfeld method, whereas one-sided non-inferiority *P* values were calculated using bootstrapping and 1,000 iterations. HRs and their corresponding two-sided *P* values were calculated using Cox regression analysis. No adjustments were made for multiple comparisons. **b**, A post hoc bootstrap extrapolation to the originally planned sample size is shown, which simulates the results shown in **a** to a hypothetical cohort involving the initially planned size of study cohort (*N* = 2,334). All CIs and *P* values (two-sided for superiority and one-sided for non-inferiority) were calculated using bootstrapping with resampling and 1,000 iterations. Therefore, the minimum *P* value that can be reported is 0.001. No adjustments were made for multiple comparisons. **c**−**e**, All endpoints and their CIs analyzed are shown using ITT (**c**), as-treated (**d**) and mITT (**e**) approaches. The WLR, its CI and two-sided superiority *P* values were calculated using the Finkelstein and Schoenfeld method, whereas one-sided non-inferiority *P* values were calculated using bootstrapping and 1,000 iterations. HRs and their corresponding two-sided *P* values were calculated using Cox regression analysis. No adjustments were made for multiple comparisons. The control group was treated with clopidogrel and in-hospital aspirin but is indicated here in a simplified fashion as clopidogrel. Data are presented as median HR or median WLR with 95% CIs. PP, per-protocol.

Treatment with DOACs and potent P2Y12 inhibitors was not associated with a significant reduction in any ischemic endpoint. It led to a nominal significant increase in all groups of BARC ≥ 3, BARC ≥ 2 and BARC ≥ 1 bleeding complications 6 weeks after randomization (Fig. 3 and Extended Data Tables 4 and 5). The difference in bleeding events between the treatment groups gradually disappeared after switching from potent P2Y12 inhibitors back to clopidogrel and was no longer statistically significant 6 months after randomization (Figs. 3 and 4 and Extended Data Tables 4 and 5). A BARC bleeding event ≥2 was associated with a nominal significant increase in the incidence of subsequent ischemic complications, regardless of treatment allocation (hazard ratio (HR) 2.18, 95% CI 1.16−4.11, *P* = 0.016).

### Sensitivity analyses

**Extended Data Fig. 1:**
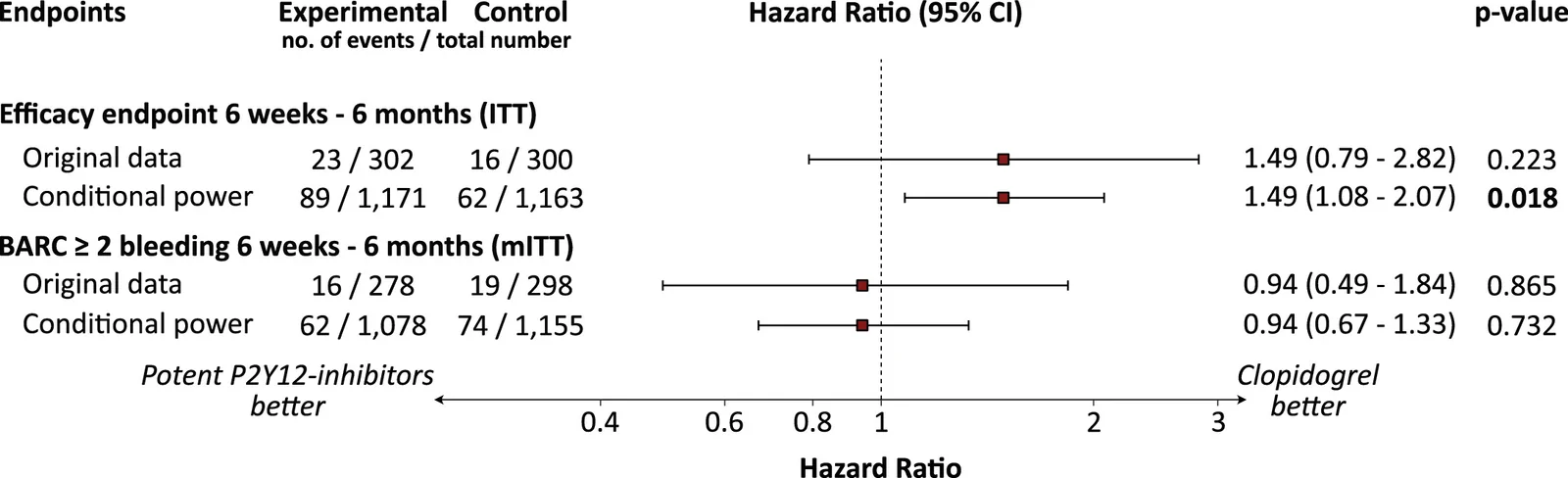
Landmark analysis. Effects of treatment on efficacy and BARC ≥ 2 bleeding between 6 weeks and 6 months after randomization in the original population and using conditional powering. Hazard ratios and their corresponding two-sided p-values were calculated using Cox-regression analysis for original data and using bootstrapping with resampling and 1,000 iterations for conditional power analysis. No adjustments were made for multiple comparisons. Data are presented as median hazard ratio with 95% confidence intervals. BARC: Bleeding Academic Research Consortium; ITT = intention-to-treat.

Figure 4 illustrates the effect of experimental treatment on ischemic and safety endpoints using intention-to-treat (ITT) (Fig. 4c), as-treated (Fig. 4d) and per-protocol (Fig. 4e) analyses for all endpoints. In the explorative as-treated analysis, the safety WLR showed significantly higher safety complications in the experimental group (WLR = 0.59, 95% CI: 0.35–0.99, *P* = 0.047; Fig. 4d). Figure 4b illustrates a sensitivity analysis that emulates the conditional power of the trial at its originally planned sample size, based on the patients enrolled in the trial at the time of termination. In this conditional power analysis, no significant reduction was observed in ischemic events but a nominal significant increase in all bleeding complications. The conditional power analysis also revealed a potentially higher rate of ischemic complications in the experimental group than in the control group in a landmark analysis between week 6 and month 6 after randomization HR 1.49, 95% CI: 1.08−2.07, *P* = 0.018; Extended Data Fig. 1). Extended Data Table 6 depicts the effect of treatment with potent P2Y12 inhibitors on various ischemic and safety endpoints at the end of the intervention period, 4 weeks after randomization.

### Subgroup analyses

**Fig. 5:**
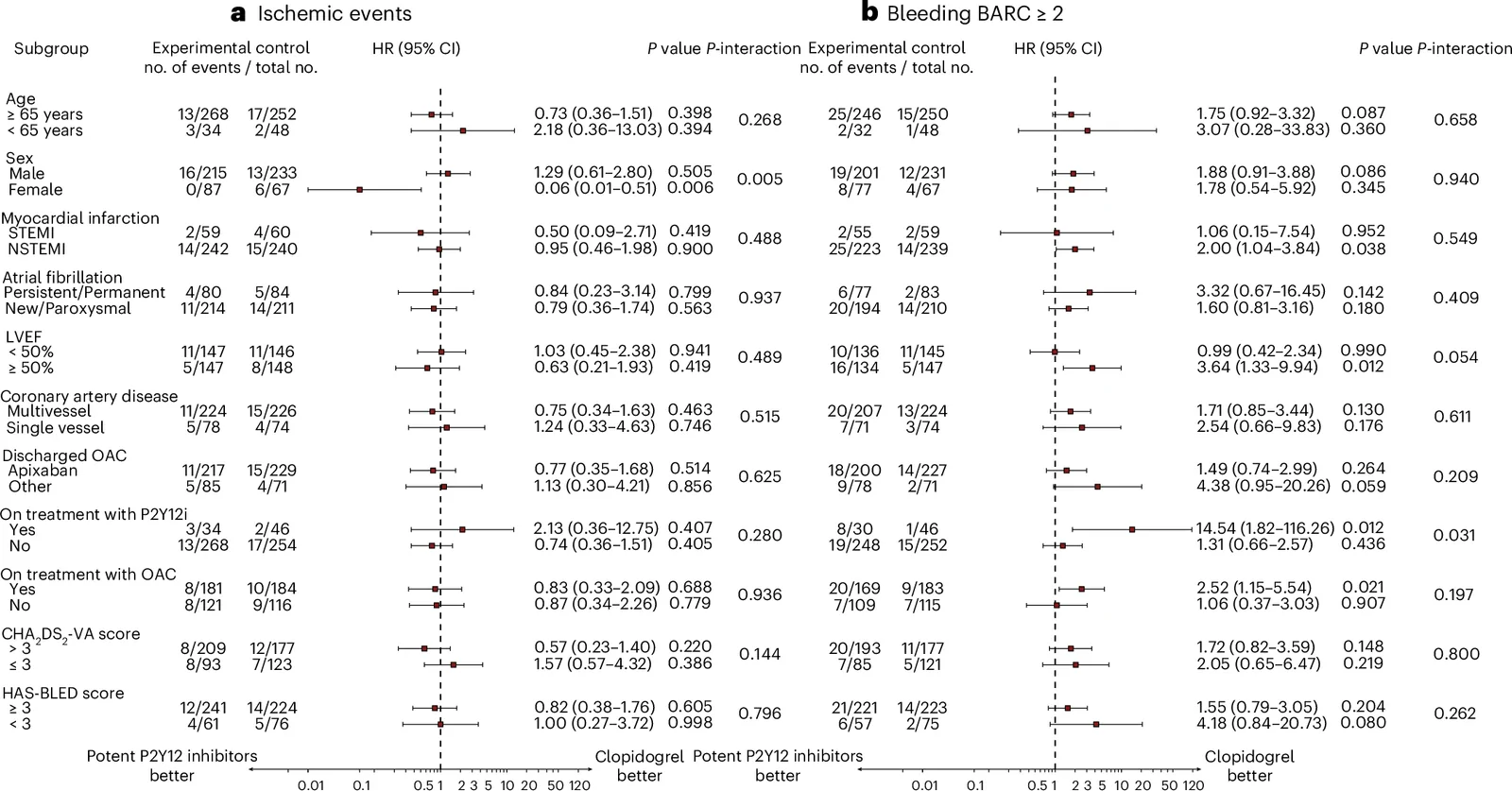
Subgroup analyses. **a**,**b**, Effects of treatment on ischemic (**a**) and BARC ≥ 2 bleeding (**b**) events for predefined subgroups. Ischemic endpoints were defined as the composite of death, stent thrombosis, myocardial infarction, ischemic stroke, systemic thromboembolism and urgent revascularization. The control group included patients treated with clopidogrel and in-hospital aspirin but is indicated in a simplified fashion as clopidogrel. HRs and their corresponding two-sided *P* values were calculated using Cox regression analysis. No adjustments were made for multiple comparisons. Data are presented as median HR with 95% CIs.

Exploratory predefined subgroup analyses of the efficacy endpoint revealed a nominal significant interaction between sex and the reduction in ischemic complications resulting from treatment with potent P2Y12 inhibitors (Fig. 5). Among the 87 females treated with prasugrel or ticagrelor, no ischemic events were recorded 6 weeks after randomization. Conversely, among the 67 females treated with clopidogrel and in-hospital aspirin, six ischemic events were noted. For the safety endpoints, pre-existing treatment with a P2Y12 inhibitor was associated with a nominal significant increased rate of bleeding complications among patients randomized to escalated P2Y12 inhibitors. Among the 30 patients receiving a P2Y12 inhibitor at baseline who were randomized to intensified P2Y12 inhibition, eight experienced BARC ≥ 2 bleeding. Notably, six of these patients received a loading dose of ticagrelor (180 mg) or prasugrel (60 mg) during or shortly after PCI. In all other subgroups, no significant interactions were noted for either efficacy or safety endpoints.

## Discussion

EPIDAURUS is, to our knowledge, the first randomized trial to evaluate a strategy of intensified P2Y12 inhibition with prasugrel or ticagrelor in combination with a direct factor Xa inhibitor in patients with AF and ACS. The trial was terminated early after a recommendation from the DSMB. Intensified P2Y12 inhibition was associated with a consistent bleeding signal, including higher rates of bleeding across conventional BARC bleeding secondary endpoints, without evidence of reduced ischemic events. Although current guidelines discourage TAT consisting of aspirin, plus OAC and a potent P2Y12 inhibitor (class III recommendation)^14^, uncertainty has remained regarding DAT incorporating OAC plus prasugrel or ticagrelor. Although the 2020 ESC guidelines for the management of ACS in patients without ST-segment elevation recommended considering the combination of a DOAC with prasugrel or ticagrelor in patients at high risk of stent thrombosis^13^, the latest ESC guidelines for the management of ACS^14^ removed this recommendation, emphasizing the need of randomized trials to test the efficacy and safety of this antithrombotic combination therapy. The present trial provides randomized evidence addressing this knowledge gap and reinforces current guideline preference for clopidogrel and in-hospital aspirin in most patients with AF and ACS requiring OAC. Limited by early termination and low event numbers, the findings of EPIDAURUS do not support routine short-term escalation to prasugrel or ticagrelor.

The limited evidence regarding the combination of DOACs with potent P2Y12 inhibitors in patients with ACS and AF largely reflects the design of the four landmark randomized trials that established DOAC-based DAT as an alternative to VKA-based TAT^3–6^. In PIONEER AF-PCI, a total of 2,124 patients with AF undergoing PCI were randomized in a 1:1:1 ratio to a standard TAT regimen with VKA and DAPT (control group), a DAT group receiving rivaroxaban in a reduced dose (15 mg) plus a P2Y12 inhibitor for 12 months (first intervention group) and a TAT group treated with very-low-dose rivaroxaban (2.5 mg twice daily) plus DAPT, followed by rivaroxaban 15 mg at the time of P2Y12 inhibitor discontinuation (second intervention group)^6^. In this trial, 30% of the patients presented with biomarker-positive ACS. Clopidogrel was the predominant P2Y12 inhibitor used, whereas only 6% of the patients received potent P2Y12 inhibitors^6^. RE-DUAL PCI randomized 2,725 patients with AF undergoing PCI (54% presented with ACS) to two regimens with dabigatran (110 mg and 150 mg) plus a P2Y12 inhibitor (clopidogrel in 88%, ticagrelor in 12%) or a standard TAT regimen with VKA, clopidogrel and aspirin for 1−3 months^5^. In ENTRUST-AF PCI, 1,506 patients with AF undergoing PCI were randomized to a DAT regimen based on edoxaban and a TAT regimen with VKA, P2Y12 inhibitor and aspirin. Fifty-two percent of enrolled patients presented with an ACS, and only 7% were treated with a potent P2Y12 inhibitor^3^. Finally, in the AUGUSTUS trial, 4,614 patients with coronary artery disease and AF were randomized in a two-by-two factorial design to P2Y12 inhibitor plus apixaban versus VKA and to aspirin (TAT regimen) versus placebo (DAT regimen) for 6 months. Only 37% of the patients presented with an ACS treated with PCI, and only 7% of the patients received potent P2Y12 inhibitors^15^.

Collectively, these trials enrolled 10,969 patients with an indication for OAC and a P2Y12 inhibitor. However, only 36% underwent PCI because of ACS, and only 8% received a potent P2Y12 inhibitor, predominantly ticagrelor^16^. This corresponds to approximately 320 patients with AF undergoing PCI for ACS who were treated with a DOAC in combination with a potent P2Y12 inhibitor. Consequently, these studies cannot reliably determine the safety and efficacy of potent P2Y12 inhibition in this population. Instead, their primary objective was to compare DOAC-based DAT with VKA-based TAT and, in AUGUSTUS, to determine the optimal duration of aspirin therapy within TAT. A meta-analysis of these trials demonstrated that TAT reduced stent thromboses and ischemic events, particularly during the first month after ACS, albeit at the expense of increased clinically relevant bleeding^7^. These findings suggest that intensified antithrombotic therapy can reduce residual ischemic risk in patients with AF and ACS at the cost of increased bleeding. However, because potent P2Y12 inhibitors were used in only a small minority of patients, and all comparisons were made against VKA-based TAT, it remains unclear whether a more selective strategy of transiently intensified platelet inhibition could achieve similar ischemic protection without the bleeding liability associated with TAT. Whether DAT with a potent P2Y12 inhibitor and a DOAC can reduce ischemic events with acceptable increase in bleeding was not known.

EPIDAURUS is, to our knowledge, the first randomized trial specifically designed to address this question. Contrary to our hypothesis, a strategy of transiently intensified platelet inhibition with ticagrelor or prasugrel did not significantly reduce ischemic events during the first month after PCI. Instead, treatment was associated with an increased risk of bleeding and a numerical excess of late ischemic events. The strength of the bleeding signal led the DSMB to recommend early termination of the trial. These findings raise the possibility that excess bleeding associated with intensified P2Y12 inhibition may have attenuated any potential ischemic benefit. Several mechanisms have been proposed as possible pathophysiological mechanisms explaining the link between bleeding and ischemic complications in patients after PCI^17^. First, bleeding provokes prothrombotic responses^18^. Moreover, disruption or antagonization of antithrombotic therapy in response to bleeding leads to a progressive recovery of platelet function and coagulation activity^17^ and has been identified as an independent predictor of subsequent ischemic events and mortality^19^. In addition, patient adherence to therapy may decrease after a bleeding event because of the fear of recurrent bleeding. Finally, transfusion of blood and coagulation products may increase oxidative stress and activate thrombotic pathways^17^.

An unexpected finding was the numerically higher incidence of ischemic stroke among patients treated with potent P2Y12 inhibitors at both 6 weeks and 6 months. In a sensitivity analysis using conditional power, this effect would be statistically significant at the original sample size. Notably, the association between bleeding and ischemic complications observed in previous studies did not extend to ischemic stroke in EPIDAURUS, as no stroke event was preceded by a bleeding complication. Although the small number of events precludes definitive conclusions, and a chance finding cannot be excluded, it deserves careful monitoring in future trials and pooled analyses.

Explorative subgroup analyses revealed no decisive evidence on the interaction between treatment with potent P2Y12 inhibitors and sex with respect to ischemic events despite a significant nominal *P* value. Previous studies showed that women exhibit greater platelet reactivity than men in response to adenosine diphosphate (ADP)^20^. This observation should be interpreted only as hypothesis generating, as the study was prematurely stopped, but may justify prespecified evaluation in future meta-analyses in view of the early termination of the trial and of the low number of women enrolled. Subgroup analyses also provided an explorative signal regarding higher bleeding rates among patients who were on treatment with P2Y12 inhibitors at the time of randomization and were switched to prasugrel or ticagrelor. Although the number of events was small and this analysis is only explorative and hypothesis generating, these findings raise the possibility that switching between P2Y12 inhibitors in the presence of concomitant DOAC therapy may be associated with an increased bleeding risk. No evidence for interactions was observed between treatment and other subgroups. However, it should be noted that patients younger than 65 years, women and patients with ST-elevation myocardial infarction (STEMI) were underrepresented in this cohort.

EPIDAURUS enrolled patients with AF and ACS undergoing PCI but did not specifically select patients according to clinical or procedural characteristics associated with a particularly high risk of recurrent ischemic events or stent thrombosis. Accordingly, the potential absolute ischemic benefit of intensified P2Y12 inhibition may have been limited in a population not specifically enriched for high ischemic risk, whereas the associated bleeding risk remained relevant. The present findings, therefore, apply to the overall population studied and do not exclude a different benefit−risk balance in selected patients at particularly high ischemic or procedural risk. Furthermore, detailed measures of PCI complexity and the use of intravascular imaging were not systematically collected. This is relevant because intravascular imaging has increasingly been shown to improve PCI outcomes^21^ and is now recommended for selected patients undergoing PCI^14^. Whether specific clinical or procedural high-risk features identify patients with AF and ACS in whom the ischemic benefits of intensified P2Y12 inhibition outweigh the bleeding risk remains to be determined.

Another clinical trial (ADONIS-PCI; ClinicalTrials.gov NCT04695106)^22^ is evaluating the combination of a DOAC and potent P2Y12 inhibition in patients with ACS and AF. ADONIS-PCI plans to enroll 2,230 patients and differs from EPIDAURUS in several important aspects. First, dabigatran is the only permitted anticoagulant, whereas EPIDAURUS exclusively evaluated direct factor Xa inhibitors. Second, ticagrelor, but not prasugrel, is allowed as the potent P2Y12 inhibitor. Third, intensified platelet inhibition is maintained for 12 months rather than 1 month, although the ticagrelor dose is reduced from 90 mg to 60 mg twice daily after the first month. Whether the increased bleeding observed in EPIDAURUS reflects the use of direct factor Xa inhibitors, inclusion of prasugrel or other differences in treatment strategy remains to be determined and may be clarified by ADONIS-PCI. Together, EPIDAURUS and ADONIS-PCI will provide complementary evidence regarding the safety and efficacy of combining potent P2Y12 inhibitors with DOAC in patients with ACS and AF.

EPIDAURUS also has relevance beyond its standalone trial result. The present findings could be incorporated into updated meta-analyses of randomized evidence. Such analyses may refine the estimated balance between ischemic protection and bleeding risk in this high-risk population. However, aggregate data meta-analysis will have important limitations, because treatment strategies differ with respect to the type of OAC, the choice and dose of P2Y12 inhibitor, aspirin exposure, timing of de-escalation and endpoint definitions. An individual patient data meta-analysis would be particularly valuable. It would allow harmonized endpoint definitions, consistent landmark analyses and direct modeling of early versus late treatment effects. It would also permit more reliable exploration of clinically relevant effect modifiers, including sex, age, ACS type, baseline bleeding risk, renal function, prior P2Y12 inhibitor exposure, loading at the time of switching between P2Y12 inhibitors and the occurrence of bleeding as a time-dependent predictor of subsequent ischemic events. Together with ongoing trials such as ADONIS-PCI, EPIDAURUS can, therefore, contribute to a more precise evidence base for tailoring antithrombotic therapy in patients with AF and ACS undergoing PCI. To maximize this contribution, harmonized data sharing and prospective planning of individual patient data meta-analyses should be encouraged.

Several limitations of the present study should be acknowledged. First, the trial was terminated after enrolment of 602 of 1,474 patients after a recommendation from the DSMB because of safety concerns. Despite the reduced sample size, a clear signal for increased bleeding associated with potent P2Y12 inhibition was observed. However, the possibility cannot be excluded that a larger trial might have detected a reduction in ischemic events. To address this issue, we performed a sensitivity analysis based on the conditional expansion of the cohort to the originally planned sample size, which did not suggest a materially different pattern under the assumptions of the extrapolation. Second, because the trial was designed using a hierarchical WLR approach and the primary endpoint was not met, all subsequent analyses should be considered exploratory and interpreted with caution. Moreover, it is important to note that all standalone bleeding complications were secondary endpoints in this trial and should, therefore, be interpreted as supportive rather than confirmatory. Third, the selection of the escalated P2Y12 inhibitor (ticagrelor or prasugrel) and the factor Xa inhibitor (edoxaban, apixaban or rivaroxaban) and the decision to administer a loading dose at the time of switching between P2Y12 inhibitors were at the discretion of the treating physician. Consequently, the results can be interpreted only in the context of a treatment strategy trial, and no conclusions can be drawn about specific drug combinations. Given the differing bleeding rates observed in current head-to-head comparisons of DOACs, further studies testing different drug combinations are warranted^23^. Fourth, all patients underwent peri-interventional loading with aspirin, which irreversibly inhibits cyclooxygenase for the lifespan of a thrombocyte. Therefore, it cannot be excluded that a different trial design, involving randomization before aspirin loading, could result in fewer bleeding events in the experimental group. Fifth, the primary safety analysis was performed using a modified ITT (mITT) approach in the PPS, as prespecified in the trial protocol and statistical analysis plan. Protocol deviations and crossovers occurred at different frequencies between the randomized treatment groups, which may have introduced bias. To provide a comprehensive assessment of the safety findings, we, therefore, additionally present ITT and as-treated analyses in the FAS alongside the prespecified mITT analysis in the PPS. Sixth, only 18% of screened patients met the inclusion criteria and agreed to participate in the study, which limits the generalizability of the results. Detailed reasons for non-enrollment were not available for all centers. Investigators considered the original requirement for randomization within 24 h after PCI an important feasibility barrier, leading to its subsequent extension to 5 days. Finally, the statistical design was modified during the course of the trial, resulting in a transition from the original primary endpoint analysis to the WLR methodology. To ensure transparency and facilitate interpretation, results based on both statistical approaches are presented throughout the paper.

Nevertheless, EPIDAURUS remains, to our knowledge, the first randomized controlled trial comparing the strategy of intensified platelet inhibition using ticagrelor or prasugrel in combination with a direct factor Xa inhibitor with standard clopidogrel-based therapy and in-hospital aspirin in patients with AF and ACS. The trial was designed to address an important unresolved question regarding the role of potent P2Y12 inhibitors in patients at high ischemic risk. In EPIDAURUS, explorative results provide a signal that intensified P2Y12 inhibition was associated with a higher incidence of bleeding without evidence of a reduction in ischemic events. Within the limitations of premature termination, low overall ischemic event rates and the fact that standalone bleeding outcomes were secondary endpoints, these findings do not support the routine use of potent P2Y12 inhibitors in combination with direct factor Xa inhibitors in patients with AF and ACS undergoing PCI. Instead, they reinforce the current evidence base that supports the use of clopidogrel as the preferred P2Y12 inhibitor in most patients requiring concomitant OAC.

## Methods

### Trial design and oversight

EPIDAURUS was a prospective, investigator-initiated, parallel-group, open-label, randomized controlled trial conducted at 37 tertiary sites in Germany and Austria. The trial was approved by the local ethics committee of the Ludwig Maximilians University Munich (number 21-0807 fed), at all participating centers and by the legal authorities (German Federal Institute for Drugs and Medical Devices (BfArM) and Austrian Federal Office for Safety in Health Care (BASG)). The study was performed in accordance with the Declaration of Helsinki and Good Clinical Practice principles. The trial was overseen by a Steering Committee and a DSMB. The sponsor of the trial was the Ludwig Maximilians University Hospital Munich. Safety assessment, monitoring and data management were done by the ISAResearch Center, TUM University Hospital German Heart Center, Munich. The study is registered with ClinicalTrials.gov (NCT04981041). The first and latest study protocol versions, as well as the statistical analysis plan of the trial, are appended in Supplementary Notes 2 and 3, respectively. Daiichi Sankyo Europe and the Ludwig Maximilians University Hospital Munich funded the trial. Daiichi Sankyo Europe did not participate in the trial design, data collection or data analysis or in the decision to submit the manuscript for publication. There were no agreements between the investigators and Daiichi Sankyo Europe. The three principal investigators (K.D.R., U.M. and S.M.) had unrestricted access to the data.

### Participants

Eligible patients were 18 years of age or older who had an indication for OAC because of AF. All patients underwent successful PCI no more than 5 days prior to randomization, because of a biomarker-positive myocardial infarction with (STEMI) or without (NSTEMI) ST elevation. Patients were excluded if they had chronic renal insufficiency with estimated glomerular filtration rate < 15 ml min^−1^ per 1.73 m^2^, had chronic liver disease Child−Pugh class C, had a history of both ischemic stroke/transient ischemic attack and intracranial bleeding, had an indication for OAC with VKA, had severe mitral stenosis, had an implanted mechanical heart valve, had a contraindication for clopidogrel or aspirin or had a contraindication for both prasugrel and ticagrelor. Individuals were also excluded if they were treated with glycoprotein IIb/IIIa inhibitors within the last 12 h, had BARC ≥ 2 bleeding within the last 4 weeks before the index PCI or had a known genetic disorder involved in the metabolism of the study medication. All study participants provided written informed consent. At baseline medical history, comorbidities, symptoms, laboratory values, medication and electrocardiographic, echocardiographic and interventional data were recorded. All sites were encouraged to provide detailed screening logs. For sites with no availability of screening log, we considered the same number of screened and randomized patients.

### Intervention

Eligible patients were randomly assigned in a 1:1 ratio to receive DOAC and prasugrel or ticagrelor (experimental group) for 4 weeks followed by DOAC and clopidogrel or DOAC, clopidogrel and aspirin (control group). The trial was designed to estimate the effect of a treatment strategy of transient intensified P2Y12 inhibition with a direct factor Xa inhibitor rather than the isolated pharmacological effect of a specific P2Y12 inhibitor or DOAC. Therefore, the selection of prasugrel or ticagrelor in the experimental group, the dosage of prasugrel (5 mg versus 10 mg) and the selection of DOAC medication were at the discretion of the treating physician. Randomization was internet based and was performed with the use of blocks of block size 8 and was stratified by center. The default duration of aspirin therapy in the control arm was in-hospital and up to 1 week. However, the treating physicians were allowed to extend the therapy with aspirin in the control arm for a period of up to 4 weeks in patients with high ischemic risk. Loading during switching between P2Y12 inhibitors was performed according to the discretion of the treating physician. The recommendation of the Steering Committee, which was documented in the study protocol and question-and-answer manual of the trial, was to load with 180 mg ticagrelor during switching from clopidogrel to ticagrelor and to load with 600 mg clopidogrel during switching from ticagrelor to clopidogrel. Switching between clopidogrel to prasugrel and vice versa was recommended to take place without concomitant loading. With respect to DOACs, only factor Xa inhibitors (edoxaban, apixaban and rivaroxaban) were allowed to be used in the trial. Patients on treatment with dabigatran or VKAs were permitted to participate, provided that their OAC was switched to a factor Xa inhibitor. Patients who withdrew their consent and had been randomized to the experimental group were switched to the control treatment, according to current guidelines^14^.

### Outcomes

The primary efficacy endpoint of the study was defined as the composite of (1) all-cause mortality, (2) definite or probable stent thrombosis, (3) myocardial infarction, (4) ischemic stroke, (5) systemic thromboembolism, and (6) urgent revascularization within 6 weeks after randomization. The composite safety endpoint was defined as the composite of (1) death, (2) BARC 3c bleeding, (3) BARC 3b bleeding, (4) BARC 3a bleeding and (5) BARC 2 bleeding. All components of both primary endpoints were adjudicated in a blinded manner by an independent Endpoint Adjudication Committee consisting of three experienced cardiologists. Secondary endpoints included the primary endpoints evaluated at 6 months, all individual components of the primary endpoints evaluated at 6 weeks and 6 months, unplanned hospitalization due to ACS or acute decompensated heart failure at 6 months and all bleeding complications (including BARC 1 bleeding) at 6 weeks and 6 months.

### Follow-up

The patients received a personal discharge visit and a personal or telephonic visit at 42 ± 7 days (primary endpoint) and at 180 ± 10 days (secondary endpoints). Patients who withdrew their consent completely were censored at the time of withdrawal and analyzed up to that point. Those who partially withdrew their consent but agreed to receive follow-up visits were switched to control therapy and were registered as protocol deviations.

### Sample size calculation

The trial was designed to detect superiority of the experimental treatment compared to the control treatment with respect to the efficacy endpoint and non-inferiority with respect to the safety endpoint using a hierarchical testing. The analysis of both endpoints was planned to take place using the Finkelstein and Schoenfeld WLR according to the defined ranking of endpoints in the outcomes section. For sample size calculation, we used aggregate component-specific event rates observed among the first 223 control group participants. No between-group treatment effect estimates or comparative analyses were reviewed. Sample size operating characteristics under the revised WLR analysis were evaluated using 1,000,000 simulation iterations. For the efficacy endpoint, we found that with a two-sided *α* of 0.05 and 6-week hazard rates and standard errors of hazard rates according to the values provided in Extended Data Table 7, the power would be 80.0%, if comparison of WLR would be performed on a total sample size of 615 per group. To account for dependency between efficacy endpoints, the sample size was increased by 10%, according to previous suggestions^24^, which led to a calculated sample size of 677 per group. For the safety endpoint, we assumed an effect size of 0.95 and a non-inferiority delta of 30%. We found that with a total sample size of 695 per group, a two-group large-sample normal approximation test of proportions with a one-sided 2.5% significance level would have 80% power to reject the null hypothesis that the lower 97.5% CI of the WLR compared between the experimental arm and control arm is lower than 0.77 (1/(1 + delta), with delta = 0.3) in favor of the alternative hypothesis, that the lower 97.5% CI is greater than or equal to 0.77. For safety endpoints, no dependency between the endpoints was considered, as the endpoints of the safety analysis are mutually exclusive. The sample size of the trial was given by the maximum of both sample sizes—that is, 695 per group. Assuming an allocation ratio *n*_control_/*n*_interventional_ = 1.0 and a 6% drop-out rate, the sample study of the study was calculated to be *N* = 1,474.

### Statistical analysis

Continuous data are presented as medians with lower and upper quartiles (Q1−Q3). Categorical data are summarized with the use of frequencies and proportions. A hierarchical testing was applied to test the superiority of the experimental treatment compared to the control treatment with respect to the efficacy endpoint and the non-inferiority with respect to the safety endpoint. Because the primary efficacy superiority test was the first step in the testing hierarchy, all subsequent safety, secondary, subgroup, landmark and sensitivity analyses are interpreted descriptively and exploratively unless the first step is met. Outcomes were analyzed using WLR and time-to-event methods. For WLR, CIs and superiority *P* values were calculated using the Finkelstein and Schoenfeld method^24^, whereas non-inferiority *P* values were calculated using bootstrapping and 1,000 iterations. For time-to-event analysis, we used the Kaplan−Meier method and Cox regression analysis. Event rates and their CIs were estimated using the Kaplan−Meier method. The absolute risk difference and its CI were calculated using bootstrapping with 2,000 iterations, by estimating and subtracting the event rates calculated using the Kaplan−Meier method. The effect of bleeding on ischemic complications was exploratively tested by introducing bleeding as a time-dependent covariate in a Cox regression model stratified by treatment. Ischemic events occurring before the bleeding event were not counted as subsequent ischemic events. Subgroup analyses were performed by introduction of the product of treatment and subgroup identifiers in a multivariable Cox regression model. For subgroups without events, we calculated CIs using Cox regression with Firth’s penalized likelihood implementation. The proportional hazards assumption was tested using the method proposed by Grambsch and Therneau^25^. Landmark analysis for ischemic events was performed between week 6 and month 6. According to the study protocol, we analyzed efficacy endpoints in the FAS according to the ITT principle. Safety endpoints were analyzed in the predefined mITT/PPS population, comprising randomized patients who received the assigned treatment strategy without major protocol deviations affecting safety analysis. Non-inferiority testing for the safety WLR was tested using a one-sided *α* of 0.025 and a non-inferiority margin of 30%. For all other analyses, differences were considered statistically significant when the two-sided *P* value was less than 0.05. All statistical analyses were performed using CRAN R, version 4.6.0, and the following packages: survival (version 3.8.6), coxphf (version 1.13.4), ggplot2 (version 4.0.3) and survminer (version 0.5.2).

### WLR approach

For the primary WLR analysis, each patient in the experimental group was compared with each patient in the control group, resulting in all possible unmatched pairwise comparisons between treatment groups. Pairwise comparisons were performed according to the prespecified hierarchical order of the endpoint components. For the primary efficacy endpoint, the hierarchy was all-cause death, definite or probable stent thrombosis, myocardial infarction, ischemic stroke, systemic thromboembolism and urgent revascularization. For the primary safety endpoint, the hierarchy was all-cause death, BARC 3c bleeding, BARC 3b bleeding, BARC 3a bleeding and BARC 2 bleeding.

For each component, only the first occurrence of that component within the 6-week assessment window was considered. If a patient experienced more than one component event, the comparison was determined by the highest-ranking component in the hierarchy that allowed a distinction between the two patients. Lower-ranking events were considered only if higher-ranking components did not distinguish the pair. For each pair, the patient with the less favorable outcome was assigned the loss. If only one patient experienced the relevant component event, that patient lost the pairwise comparison. If both patients experienced the same component event, the patient with the earlier event lost the comparison. If both patients experienced the same component event on the same day, that component did not distinguish the pair. If neither patient experienced the component event, the comparison proceeded to the next component in the hierarchy.

All-cause death was included as the highest-ranking component in both the efficacy and safety hierarchies. Therefore, death took precedence over all non-fatal ischemic and bleeding events when it distinguished a patient pair. For patients who experienced death after a non-fatal event, death was still considered the highest-ranking event for the hierarchical comparison. Patients who withdrew consent or were lost to follow-up before 6 weeks were censored at the date of last available information. Events occurring before censoring were included. If a patient had no event before censoring, that patient was considered event free on day 42 (6 weeks). A tie was recorded if none of the hierarchical components allowed a conclusive distinction between the two patients, either because neither patient had an event or because both had the same event at the same time. The WLR was calculated as the total number of wins for the experimental group divided by the total number of losses for the experimental group^24^. Thus, a WLR greater than 1 favored the experimental group, whereas a value less than 1 favored the control group. This direction applied to both the efficacy and safety endpoints, because all endpoint components represented unfavorable clinical events.

### Protocol revisions

The EPIDAURUS trial was initially planned to include 2,334 patients. The primary efficacy endpoint was defined as the composite of death, stent thrombosis, myocardial infarction, ischemic stroke and systemic thromboembolism. The primary safety endpoint was defined as BARC bleeding ≥2. Both endpoints were intended to be evaluated using a logistic regression analysis. During the course of the trial, the protocol was revised in the following ways: (1) the timeframe between successful PCI and randomization was extended from 24 h to 5 days (protocol version 6; 20 March 2024; a total of 337 patients were included at this time in the trial); (2) the primary efficacy endpoint was extended to include urgent revascularization (protocol version 8; 5 January 2025; a total of 487 patients were included at this time in the trial); (3) the primary safety endpoint was revised to include all-cause mortality and BARC bleeding categories 2, 3a, 3b and 3c (protocol version 8; 5 January 2025); and (4) the evaluation of the primary endpoints was revised to use the Finkelstein and Schoenfeld WLR (protocol version 8; 5 January 2025). Protocol version 8 reduced the required sample size from 2,334 to 1,474 patients. A chronological summary of all protocol revisions, the changes and their rationale is depicted in Extended Data Table 8. All protocol revisions were approved by the Steering Committee. The decision for protocol revisions did not involve any interim analysis or access to comparative outcome data. The first and latest protocol versions (versions 4 and 8 and all protocol changes) are included in Supplementary Note 2.

### Sensitivity analyses

The following sensitivity analyses were included to ensure robustness against bias. First, the FAS/ITT safety analysis was prespecified as a sensitivity analysis and is presented to assess robustness against post-randomization exclusions. Second, a conditional power analysis using bootstrapping with resampling and 1,000 iteration was used to simulate the initial sample size population. Third, we tested the association of treatment with ischemic and safety endpoints at the following intervals: 4 weeks (end of intervention), 6 weeks (predefined evaluation of the primary endpoints), 6 months (predefined evaluation of secondary endpoints) and between week 6 and month 6 (landmark analysis). Finally, the initial and revised efficacy and safety endpoints are presented throughout the paper.

### Reporting summary

Further information on research design is available in the Nature Portfolio Reporting Summary linked to this article.

## Online content

Any methods, additional references, Nature Portfolio reporting summaries, source data, extended data, supplementary information, acknowledgements, peer review information; details of author contributions and competing interests; and statements of data and code availability are available at https://doi.org/10.1038/s41591-026-04629-7.

## Supplementary information

Supplementary Information 1. EPIDAURUS trial investigators; 2. Boards; 3. Supplementary Table 1; 4. DSMB

Reporting Summary

Supplementary Note 1 DSMB charter and DSMB decisions

Supplementary Note 2 EPIDAURUS protocol, protocol revisions and changes

Supplementary Note 3 EPIDAURUS statistical analysis plan and amendment

## Data Availability

Upon request, investigators can be given access to anonymized raw data for further scientific projects provided the positive evaluation of the steering committee, the local ethics committee of the Ludwig Maximilians University Munich and the data protection officer of the LMU University Hospital. Free availability of raw data is not possible due to legal restrictions. Requests for data availability can be made to the corresponding author (K.D.R., konstantinos.rizas@h-och.ch). The expected response time is 2 weeks, and the decision-making process takes 3 months.

## Extended data

**Extended Data Table 1:** Medication on day 7 after randomization stratified by randomization group in the full-analysis set

Treatment 7 days after randomization			
Medication	Experimental group	Control group	Total
ASA	7 / 288 (2.4%)	56 / 291 (19.2%)	63 / 579 (10.9%)
Clopidogrel, N/total N (%)	11 / 288 (3.8%)	289/ 291 (99.3%)	300 / 579 (51.8%)
Ticagrelor, N/total N (%)	119 / 288 (41.3%)	0 / 291 (0.0 %)	119 / 579 (20.6%)
Prasugrel, N/total N (%)	158 / 288 (54.9%)	0 / 291 (0.0 %)	158 / 579 (27.3%)
Apixaban, N/total N (%)	199 / 288 (69.1%)	217 / 291 (74.6%)	418 / 579 (72.2%)
Edoxaban, N/total N (%)	43 / 288 (15.0%)	29 / 291 (9.9%)	72 / 579 (12.4%)
Rivaroxaban, N/total N (%)	28 / 288 (9.8%)	35 / 291 (12.0%)	63 / 579 (10.9%)
Dabigatran, N/total N (%)	1 / 288 (0.3%)	3 / 291 (1.0%)	4 / 579 (0.7%)
Vit. K antagonist, N/total N (%)	0 / 288 (0.0%)	1 / 291 (0.3%)	1 / 579 (0.2%)
OAC paused	17 / 288 (5.9%)	6 / 291 (2.1%)	23 / 578 (4.0%)

ASA: Aspirin; OAC: oral anticoagulation.

**Extended Data Table 2:** Reasons for stopping oral anticoagulation on day 7 after randomization

Pseudo-id	Randomization group	Reason for stopping DOAC
S3-01	Experimental	Stroke
S3-02	Experimental	Bleeding
S3-03	Experimental	Bleeding
S3-04	Experimental	Cardiogenic shock
S3-05	Experimental	Acute myocardial infarction
S3-06	Experimental	Septic shock
S3-07	Experimental	Incompliance
S3-08	Experimental	Planned ICD-implantation
S3-09	Experimental	Unknown
S3-010	Experimental	Incompliance
S3-011	Experimental	Decision of treating physician
S3-012	Experimental	Bleeding
S3-013	Experimental	Bleeding
S3-014	Experimental	Anemia of unknown aetiology
S3-015	Experimental	Bleeding
S3-016	Experimental	Bleeding
S3-017	Experimental	Unknown
S3-018	Control	Acute renal failure
S3-019	Control	Unknown
S3-020	Control	Unknown
S3-021	Control	Bridging from Vitamin K antagonist
S3-022	Control	Planned ICD-implantation
S3-023	Control	Acute-on-chronic renal failure

**Extended Data Table 3:** Patients on treatment with potent P2Y12-inhibitors on day 34 after randomization

Pseudo-id	Randomization group	Reason for prolonged treatment with potent P2Y12-inhibitors
S4-01	Experimental	Switched later
S4-02	Experimental	Switched later due to incompliance
S4-03	Experimental	Patient was hospitalized and received Prasugrel instead of Clopidogrel
S4-04	Experimental	Incompliance
S4-05	Experimental	Incompliance
S4-06	Experimental	Not switch in external hospital
S4-07	Experimental	Switched later due to incompliance
S4-08	Experimental	Switched later due to incompliance
S4-09	Experimental	Switched later from Ticagrelor to Clopidogrel due to decision of the treating physician
S4-010	Experimental	Switched later from the center
S4-011	Experimental	Switched later from Ticagrelor to Clopidogrel due to decision of the treating physician
S4-012	Experimental	Was not switched due to decision of the treating physician
S4-013	Experimental	Decision of attending physician to proceed with ASS/Ticagrelor and no DOAC
S4-014	Experimental	Switch later - decision of the general practitioner
S4-015	Experimental	Switched later from Prasugrel to Clopidogrel due to decision of the treating physician
S4-016	Experimental	Switched later due to unknown reasons
S4-017	Experimental	Switched later due to unknown reasons
S4-018	Control	Treated with Prasugrel up day 9 due to decision of the treating physician

**Extended Data Table 4:** Effect of treatment with direct oral anticoagulants and potent P2Y12 inhibitors on efficacy endpoints

Endpoint	Experimental	Control	Absolute Risk Difference % (95% CI)	Hazard Ratio (95% CI)	p-value
	No. events / No. patients	No. events / No. patients			
	Event Rate % (95% CI)				
**Efficacy Endpoints at 6 weeks (analyzed according to the intention-to-treat principle)**					
Death, ST, MI, Ischemic Stroke, Systemic Thromboembolism, Urgent Revascularization	16 / 302 5.4 (2.8–8.0)	19 / 300 6.5 (3.6–9.2)	- 1.1 (−4.7–2.2)	0.85 (0.44–1.66)	0.639
Death, ST, MI, Ischemic Stroke, Systemic Thromboembolism	15 / 302 5.1 (2.5–7.6)	16 / 300 5.4 (2.8–8.0)	- 0.4 (−3.9–3.2)	0.95 (0.47–1.92)	0.885
Death	9 / 302 3.1 (1.1–5.0)	9 / 300 3.1 (1.1–5.0)	0.0 (−2.9–2.7)	1.01 (0.40–2.54)	0.983
Cardiovascular Death	5 / 302 2.7 (0.2–3.2)	9 / 300 3.1 (1.1–5.0)	−1.4 (−3.9–1.1)	0.56 (0.19–1.67)	0.301
Stent thrombosis	2 / 302 0.7 (0.0–1.6)	2 / 300 (0.7 (0.0–1.6)	0.0 (−1.3–1.3)	1.01 (0.14–7.14)	0.995
Myocardial infarction	5 / 302 1.7 (0.2–3.2)	7 / 300 2.4 (0.6–4.2)	−0.7 (−3.0–1.6)	0.72 (0.23–2.27)	0.575
Ischemic stroke	4 / 302 1.4 (0.0–2.7)	1 / 300 0.3 (0.0–1.0)	1.0 (−0.3–2.7)	4.03 (0.45–36.05)	0.213
Urgent revascularization	4 / 302 1.4 (0.0–2.7)	6 / 300 2.1 (0.4–3.7)	−0.7 (−3.2–1.6)	0.67 (0.19–2.39)	0.542
**Efficacy Endpoints at 6 months (analyzed according to the intention-to-treat principle)**					
Death, ST, MI, Ischemic Stroke, Systemic Thromboembolism, Urgent Revascularization	39 / 302 14.2 (9.8–18.5)	35 / 300 14.1 (8.1 –19.6)	0.4 (−7.2–7.0)	1.14 (0.72–1.80)	0.570
Death, ST, MI, Ischemic Stroke, Systemic Thromboembolism	36 / 302 13.2 (8.9–17.3)	31 / 300 12.7 (6.8 –18.3)	0.6 (−6.9–7.0)	1.19 (0.74–1.93)	0.470
Death	21 / 302 7.4 (4.3–10.4)	20 / 300 8.9 (3.4–14.0)	−1.4 (−7.7–4.1)	1.07 (0.58–1.98)	0.817
Cardiovascular Death	12 / 302 4.3 (1.9–6.7)	15 / 300 7.2 (1.9–12.2)	-2.7 (−8.9–2.0)	0.82 (0.38–1.75)	0.608
Stent thrombosis	2 / 302 0.7 (0.0–1.6)	2 / 300 0.7 (0.0–1.6)	0.0 (−1.3–1.3)	1.01 (0.14–7.14)	0.995
Myocardial infarction	13 / 302 5.4 (2.2–8.4)	12 / 300 4.3 (1.9–6.7)	1.0 (−2.7–4.9)	1.11 (0.50–2.42)	0.801
Ischemic stroke	6 / 302 2.1 (0.4–3.8)	1 / 300 0.3 (0.0–1.0)	1.8 (0.1–3.7)	6.09 (0.73–50.62)	0.094
Urgent revascularization	11 / 302 4.7 (1.6–7.6)	10 / 300 3.5 (1.4–5.7)	1.1 (−2.5–4.9)	1.13 (0.48–2.65)	0.785

MI: myocardial infarction, ST: stent thrombosis.

**Extended Data Table 5:** Effect of treatment with direct oral anticoagulants and potent P2Y12 inhibitors on safety endpoints

Endpoint	Experimental	Control	Absolute Risk Difference % (95% CI)	Hazard Ratio (95% CI)	p-value
	No. events / No. patients	No. events / No. patients			
	Event Rate % (95% CI)				
**Safety Endpoints at 6 weeks (analyzed according to the modified intention-to-treat principle)**					
Death, BARC bleeding ≥2	34 / 278 12.3 (8.4–16.2)	24 / 298 8.2 (5.0–11.3)	4.1 (−0.9–9.3)	1.58 (0.93–2.66)	0.088
BARC bleeding ≥ 3	13 / 278 4.8 (2.2–7.3)	4 / 298 1.4 (0.0–2.7)	3.4 (0.7–6.1)	3.54 (1.15–10.85)	0.027
BARC bleeding ≥ 2	27 / 278 9.9 (6.3–13.3)	16 / 298 5.5 (2.9–8.1)	4.3 (−0.1–8.9)	1.87 (1.01–3.47)	0.048
Any bleeding (BARC ≥ 1)	45 / 278 16.5 (12.0–20.8)	31 / 298 11.7 (7.1 –14.2)	5.6 (0.1–11.6)	1.65 (1.04–2.60)	0.033
**Safety Endpoints at 6 months (analyzed according to the modified intention-to-treat principle)**					
Death, BARC bleeding ≥2	58 / 278 21.7 (16.6–26.6)	51 / 298 18.4(13.1–23.3)	3.2 (−4.0–10.3)	1.29 (0.89–1.89)	0.181
BARC bleeding ≥ 3	22 / 278 8.6 (5.0–12.1)	15 / 298 6.5 (2.6–10.3)	2.1 (−3.4–7.0)	1.61 (0.84–3.11)	0.152
BARC bleeding ≥ 2	43 / 278 16.5 (11.8–20.9)	35 / 298 13.6 (8.7 –18.2)	2.7 (−3.8–9.3)	1.37 (0.88–2.14)	0.164
Any bleeding (BARC ≥ 1)	63 / 278 23.9 (18.5–29.0)	53 / 298 19.2(14.4–24.9)	4.1 (−3.4–11.6)	1.36 (0.94–1.95)	0.103

BARC: Bleeding Academic Research Consortium.

**Extended Data Table 6:** Effect of treatment on ischemic and safety endpoints 4-weeks after randomization

Endpoint	Hazard Ratio (95% CI)	p-value
Death, Stent thrombosis, MI, Ischemic Stroke, Systemic Thromboembolism, Urgent Revascularization	0.95 (0.48–1.89)	0.890
Death, Stent thrombosis, MI, Ischemic Stroke, Systemic Thromboembolism	1.01 (0.49–2.07)	0.973
Death	1.01 (0.40–2.54)	0.983
Cardiovascular Death	0.56 (0.19–1.67)	0.301
Stent thrombosis	1.01 (0.14–7.16)	0.993
Myocardial infarction	0.84 (0.26–2.75)	0.773
Ischemic stroke	4.03 (0.45–36.05)	0.213
Urgent revascularization	0.67 (0.19–2.39)	0.541
Death, BARC bleeding ≥ 2	1.60 (0.94–2.72)	0.085
BARC bleeding ≥ 3	3.54 (1.15–10.85)	0.027
BARC bleeding ≥ 2	1.92 (1.01–3.62)	0.045
Any bleeding (BARC ≥ 1)	1.99 (1.22–3.25)	0006

BARC: Bleeding Academic Research Consortium.

**Extended Data Table 7:** Event-rates used for sample size calculation

Endpoint	Control group			Intervention group			HR
	Event rate	Hazard rate	SE of hazard rate	Event rate	Hazard rate	SE of hazard rate	
**Death**	4.35%	4.44%	1.57%	3.28%	3.33%	1.18%	0.75
**Stent thrombosis**	1.17%	1.18%	0.84%	0.34%	0.35%	0.25%	0.30
**Myocardial infarction**	3.79%	3.87%	1.46%	1.91%	1.93%	0.73%	0.50
**Ischaemic stroke**	1.00%	1.01%	0.50%	0.70%	0.70%	0.35%	0.70
**Systemic embolism**	1.00%	1.01%	0.50%	0.70%	0.70%	0.40%	0.70
**Urgent revascularization**	2.77%	2.81%	1.26%	1.39%	1.40%	0.63%	0.50
**BARC 2 bleeding**	7.85%	8.18%	2.13%	7.48%	7.77%	2.02%	0.95
**BARC 3a bleeding**	3.22%	3.28%	1.34%	3.06%	3.11%	1.28%	0.95
**BARC 3b bleeding**	1.07%	1.07%	0.77%	1.01%	1.02%	0.73%	0.95
**BARC 3c bleeding**	1.00%	1.01%	0.50%	0.95%	0.95%	0.47%	0.95

6-week event-rates, hazard-rates and their corresponding standard errors (SE), which were used for sample-size calculation.

BARC: Bleeding Academic Research Consortium.

**Extended Data Table 8:** Study protocol and revisions

Protocol Version	Date	Main Changes	Rationale and Comments
1	30-Mar-2021		First submission to the German regulatory authority (BfArM)
2	21-Apr-2021	Minor Changes	Second submission to the German regulatory authority (BfArM)
3	27-May-2021	Minor Changes	First submission to the Ethics Committee
4	27-Oct-2021	Minor Changes	This was the valid protocol at the time the trial was initiated.
5	23-Dec-2021	Minor Changes	Submission to the Austrian regulatory authority (BASG)
6	20-Mar-2024	Increased the window between successful PCI and randomization from 24 h to 5 days	Version 6.0 was never submitted to the regulatory authorities or ethics committee. The rationale of this change was to improve feasibility of the study. Version 7, which contained minor changes compared to version 6, was submitted to the regulatory authorities and the ethics committee.
7	10-Jun-2024		
8	05-Jan-2025	- Changed the evaluation of the primary endpoint from logistic regression analysis to a win-loss ratio analysis. - Included urgent revascularization in the primary efficacy endpoint - Included death in the primary safety endpoint - Through the above mentioned changes, the sample size was reduced from 2,334 to 1,474 patients.	Due to the impact of COVID-19 pandemic the trial needed to be extended. The provided list of changes were performed in order to decrease the required sample size and ensure the successful completion of the trial.

Protocol versions, revisions, content changes and the rationale of the changes.

## Code availability

Custom statistical scripts (R scripts) can be accessed via https://osf.io/mc46d/overview?view_only=1c85943857a844e19ee5d78c84f82c58.
