# Supplementary material for: Dual antithrombotic therapy using potent antiplatelet inhibitors in atrial fibrillation and acute coronary syndrome: a randomized controlled trial
Source: Nat Med. 2026 Aug 29;32(11):4036–46. doi: 10.1038/s41591-026-04629-7 (PMC13768177; doi:10.1038/s41591-026-04629-7)
Supplement: Supplementary file 1 [file 41591_2026_4629_MOESM1_ESM.pdf]

nature medicine

Supplementary information
https://doi.org/10.1038/s41591-026-04629-7

Dual antithrombotic therapy using potent antiplatelet inhibitors in atrial fibrillation and acute coronary syndrome: a randomized controlled trial

In the format provided by the authors and unedited

Supplementary Information

EPIDAURUS

Table of Contents

EPIDAURUS trial Investigators 2

Boards 11

Supplementary Tables........ 14

Supplementary Table 1: 14

Data and Safety Monitoring Board 17

EPIDAURUS trial Investigators

Ludwig-Maximilians University hospital Munich - Medizinische Klinik und Poliklinik I – Campus Grosshadern Marchioninistr. 15 81377 München

PI: Prof. Dr. med. Konstantinos Rizas

Deput PI: Prof. Dr. med. Stefan Kääb

Investigators: Dr. Konstantinos Mourouzis, Dr. Laura Elisa Villegas Sierra, Dr. Peter Spielbichler, Dr. Luisa Freyer, Dr. Lauren Sams, Dr. Mathias Klemm, Dr. Enzo Lüsebrink, Dr. Aresa Krasniqi, Karin Hohenleitner-Lührßen, Katharina Grimm

Ludwig-Maximilians University hospital Munich - Medizinische Klinik und Poliklinik I – Campus Innenstadt Ziemssenstr. 5 80336 Munich

PI: Prof. Dr. med. Konstantinos Rizas

Deput PI: Prof. Dr. med. Stefan Kääb

Investigators: Dr. Konstantinos Mourouzis, Dr. Laura Elisa Villegas Sierra, Dr. Peter Spielbichler, Dr. Luisa Freyer, Dr. Lauren Sams, Dr. Mathias Klemm, Dr. Enzo Lüsebrink, Dr. Aresa Krasniqi, Monika Baylacher, Maria Fackler

TUM University Hospital German Heart Center Lazarettstr. 36 80636 Munich

PI: Prof. Dr. med. Adnan Kastrati

Deputy PI: Prof. Dr. med. Katharina Mayer, Dr. med. Constantin Kuna

Investigators: Dr. med. Constantin Kuna, Dr. med. Fabian Starnecker, Katharina Seifert, Nonglav Rifatov

Charité University Hospital – Campus Benjamin Franklin, Hindenburgdamm 30 12203 Berlin

PI: Prof. Dr. med. Tobias Petzold

Deputy PI: Dr. med Youssef Abdelawed

Investigators: Dr. med. Julius Glasmacher, Dr. med. Cinzia Tarallo , Adrian Jayanata, Doreen Kupsch, Thomas Haese, Stefanie Wengerodt, Saskia-Maria Dörge, Katja Hubert, Lukas Ley, Fardin Hamidi, Yasmin Esmati, Thomas Haese, Julia Leibiger, Anja Posselt

Charité University Hospital – Campus Clinik Virchow, Augustenburger Platz 1 13353 Berlin

PI: Prof. Dr. med. Frank Edelmann

Deputy PI: Dr. med. Tobias Daniel Trippel

Investigator: Dr. Andreas Kind, Katharina Gille, Claus Kamml, Amelie Hermel, Engy Nasr, Bianca Papke, Jeus Brestnich, Svenja Broschag, Christin Irrgang, Fabian Spinka, Roxandra-Cecilia Ionescu

Essen University Hospital, Hufelandstr. 55, 45147 Essen

PI: Prof. Dr. med. Amir A. Mahabadi

Deputy PI: Prof. Dr. med. Tienush Rassaf

Investigators: Martin Dumin, Sinem-Hilal Özlap, Becka Jurgeleit

Düsseldorf University Hospital, Moorenstr. 5, 40225 Düsseldorf

PI: Prof. Dr. med. Amin Polzin

Deputy PI: Prof. Dr. med. Malte Kelm

Investigators: Dr. med. Saif Zako, Dr. med. David Naguib, Dr. med. Philip Mourikis, Dr. med. Shazia Afzal, Dr. med. Kathrin Klein, David Naguib, Lisa Dannenberg, Mairéad Quinn, Elisabetha Gharib, Saskia Pietrucha, Sabrina Seggewiß, Alicia Timm, Carolin Helten

Protestant Hospital Hagen-Hapse, Brusebrinkstraße 20, 58135 Hagen

PI: Prof. Dr. med. Harilaos Bogossian

Deputy PI: Dr. med. Nana Yaw Bimpong-Buta

Investigators: Dr. med. Konstantinos Iliodromitis, Dr. med. Mustafa Rahel, Sebastian Robl, Ivane Kochiashvili, Mouhammed Eliliwi, Roland Ngong, Nicole Weiriech

Mannheim University Hospital, Theodor-Kutzer-Ufer 1, 368167 Mannheim

PI: Prof. Dr. med Ibrahim Akin

Deputy PI: Dr. med. Reinhard Sauter

Investigators: Prof. Dr. med. Michael Behnes, Gökhan Yücel, Mohammad Abumayyaleh, Tobias Schupp, Amanda Schölch, Stefanie Dorn, Chantal Chevalard

Tübingen University Hospital, Otfried-Müller-Straße 10, 72076 Tübingen

PI: Prof. Dr. med. Meinrad Paul Gawaz

Deputy PI: Prof. Dr. med. Dominik Rath

Investigators: Prof. Dr. Tobias Geisler, Timea Keller, Isabella Kast, Gabriela Lopez, Dr. Tatsiana Castor, Deborah Seiferth, Noel Berenzen, Katarina Ustenko, Ida Buchner, Rocyn Alarcon

Heart Center Leipzig at Leipzig University, Strümpellstr. 39, 04289 Leipzig

PI: Prof. Dr. med Holger Thiele

Deputy PI: PD Dr. med. Janine Pöss

Investigators: PD. Dr. med. Hans-Josef Feistritzer, PD. Dr. med. Maximilian von Roeder Dr. med. Maria Bukse, Dr. med. Ines Richter, Dr. med. Philipp Hartung, Dr. med. Nicolas Majunke, Ina Wagner, Maren Zieger, Roby Schurzmann, Jennifer Ottlinger, Ines Koehler, Julia Krebs, PD Dr. med. Marcus Sandri , Prof. Dr. med. Mohammed Abdel-Wahab, Julia Hechtl, , Robby Schumann, Matias Lesche, Dr. med. Niklas Lankisch, Anna Schulz, Cornelia Jeßner, Josephine Koch

Rostock University Hospital, Ernst-Heydemann-Straße 6, 18057 Rostock

PI: Prof. Dr. med. Hüseyin Ince

Deputy PI: Univ.-Prof. Dr. med. Alper Öner

Investigators: Dr. med. Univ. Stephan Valenta, Dr. med. Daniel Robinson, Dr. Nina Kleinfeldt, Dr. med. Christin Machinek, Dr. med. Daniel Robinson, Kathrin Schneider, Christiene Saam, Kerry Theelke Kathrin Schneider, Plamen Kochev, Christine Saam, Jerry Theelke

Heidelberg University Hospital, Neuenheimer Feld 410, 69120 Heidelberg

PI: Prof. Dr. med. Norbert Frey

Deputy PI: Prof. Dr. med. Moritz Biener

Investigators: PD. Dr. med. Mathias Müller-Hennessen, Dr. med. Barbara Ruth, Dr. med. Univ. Mustafa Yildirim, Dr. med. Christian Salbach, Dr. med. Constanze Winkler, Heidi Deigentasch, Amelie Werner

Hannover Medical School, Carl-Neuberg-Straße, 30625 Hannover

PI: Prof. Dr. med. Andreas Schäfer

Deputy PI: Dr. med. Tobias König

Investigators: Mircea-Andrei Sandu, Dr. med. Univ. Welf-Axel Geller, Dr. med. Welf-Axel Geller, Nicole Schäfer, Antje Jürgens, Lea Habel

Heart Center, University of Freiburg Hugstetter Str. 55, 79106 Freiburg

PI: Prof. Dr. med. Christoph Olivier

Deputy PIs: Dr. med. Stefan Leggewie, Adrian Heidenreich, Dr. Diona Gjermeni

Investigators: Dr. Lucas Bacmeister, Dr. David Hesselbarth, Mariya Maslarska, Kilian Franke, Daniela Reichenbach, Joana Meisenzahl, Julia Droese, Carina Julch, Martina Siefer, Erik De Frenne, Marius Wessinger

Study Center of the Hegau-Bodensee Klinikum Singen, Virchowstr. 10, 78224 Singen

PI: PD. Dr. med. Marc Kollum

Deputy PI: Dr. med. Christopher Stephan Richter

Investigators: Dr. med. Steffen Dengler, Heidi Zimmermann, Melissa Knaupp, Luca Herewek

Heart Center, Kerckhoff Clinic, Benekestraße 2-8. 61231 Bad Nauheim

PI: Dr. med. Jan Sebastian Wolter

Deputy PI: Dr. med Moritz Haas

Investigators: Prof. Dr. med. Roland Klingerberg, PD. Dr. med. Steffen Kriechbaum, Dr. med. Thore Körschgen, Dr. med. Catharina Hamm, Annegret Köpke, Stefan Lan Cheong Wah, Nikolai Busch, Eva Wagner, Gabriele Hellwig-Bahavar, Anett Kirchhof, Ajith Kantharajah, Dr. med. Maren Weferling

Center for Cardiology, Mainz Medical School, Langenbeckstrasse 1, 55131 Mainz

PI: Univ.-Prof. Dr. med. Tommaso Gori

Deputy PI: PD. Dr. med. Maike Knorr

Investigators: Dr. med.Steffen Daub, Dr. med. Johannes Herzog, Dr. med. Ornella Velollari, Dr. med. Helen Ullrich-Daub, Melanie Hochmuth, Maximilian Olschewski, Britta Haberkamp, Stefanie Schwibinger

Innsbruck Medical University, Anichstraße 35, 6020 Innsbruck

PI Dr. med. univ. Michael Schreinlechner

Deputy PI: Prof. Dr. Axel Bauer

Investigators: Dr. Theresa Dolejsi, Dr. Florian Hofer, Dr. Fabian Theurl, Kristin Tessadri

Barmherzige Brüder Hospital St. Elisabeth Straubing, St.-Elisabeth-Str. 23, 94315 Straubing.

PI: Prof. Dr. med Sebastian Maier

Deputy PI: Dr. med Florian Brattinger

Investigator: Stilla Jacobs

Helios Amper Hospital Dachau, Krankenhausstraße 15, 85221 Dachau

PI: PD. Dr. med. Bernhard Witzenbichler

Deputy PI: Dr. med. Hedda von zur Mühlen

Investigator: Marlene Swoboda

Schleswig-Holstein University Hospital, Campus Kiel, Arnold-Heller-Str. 3, 25105 Kiel

PI: Prof. Dr. med. Derk Frank

Deputy PI: Dr. med. Johanne Frank

Investigators: Maria Arndt, Wiebke Fentens, Andrea Koch, Iwona Pieper

Dresden Heart Center, University Hospital at T.U. Dresden, Fetscherstr. 76, 01307 Dresden

PI: Prof. Dr. med. Axel Linke

Deputy PI: Prof. Dr. med. Felix M. Heidrich

Investigators: Dr. med. Johannes Miercke , Dr. med. Manja Hubald, Dr. med. Yvonne Thomsen, Jonathan Biedermann, Martin Schönberg, Anne Trausch, Christoph Döring, Medea Adeishivili, Christoph Heitmann, Jennifer Hommel, Kristina Fache, Jan Trefny, Zouhir Dindane, Marion Mai

Erding District Hospital, Bajuwarenstr. 5, 85435 Erding

PI: PD. Dr. med. Lorenz Bott-Flügel

Deputy PI: Tobias Fröhlich-Rehfeld

Investigators: Dr. med. Isabel Suckart, Dr. med. Asllan Tahiraj, Markus Völkl, Yvonne Anders

St. Vincent Hospital Paderborn, Am Busdorf 2, 33098 Paderborn

PI: Prof. Dr. med. Andreas Götte

Deputy PI: Dr. med. Achim Wiederhold

Investigators: Fouzi Alnour, Britta Möhring

Heart Center, University of Freiburg, Campus Bad Kronzigen, Südring 15, 79189 Bad Krozingen

PI: Prof. Dr. med. Christoph Olivier

Deputy PIs: Dr. med. Stefan Leggewie, Adrian Heidenreich

Investigators: Lucas Bacmeister, Dr. David Hesselbarth, Mariya Maslarska, Kilian Franke, Daniela Reichenbach, Joana Meisenzahl, Julia Droese, Carina Julch, Martina Siefer, Erik De Frenne, Cornelia Ebel

University Heart Center, Frankfurt University Hospital, Goethe University Frankfurt am Main, Theodor-Stern-Kai 7, 60590 Frankfurt am Main

PI: Prof. Dr. med Reza Wakili

Deputy PI: PD. Dr. med Sebastian Cremer

Investigators: PD. Dr. med. Philipp Seppelt, Dr. med. Victoria Pfeiffer, Dr. med. Anastasia Falagkari, Ricarda Locker, Jana Kupusovic, Maiken Skarke, Meaza Tekeste, Victoria Johnson, Christian, Gold, Dennis Niclas, Laura Schultze-Rhonhof, Ricarda Locker, Andrin Schuff,

South Nürnberg Hospital, Breslauer Straße 201,90471 Nürnberg

PI: Riza Sahin

Deputy PI: Prof. Dr. med. Matthias Pauschinger

Investigator: Undine Sattelberger

Schleswig-Holstein University Hospital, Campus Lübeck, Ratzeburger Allee 160, 23538 Lübeck

PI: Prof. Dr. med. Ingo Eitel

Deputy PI: Prof. Dr. med. Thomas Stiermaier

Investigators: Dr. med. Christoph Morgenstern, Katrin Schönhoff, Alexandra Gottschalk, Luise Warwas, Thea Bruns, Mathias Mezger, Mulham Alhagi,

University Hospital Bielefeld Mitte, Teutoburger Straße 50D, 33604 Bielefeld

PI: Univ.-Prof. Dr. med. Christoph Stellbrink

Deputy PI: PD. Dr. med. Lawrenz Thorsten

Investigators: Dr. med. Ekaterina Stellbrink, Dr. med. Andrej Teren, Dr. med. Lisa Marten, Dr. med.Kristin Marx, Britta Brettschneider, Stephanie Watson, Britta Brettschneider, Marion Iselt

Sana Klinikum Lichtenberg Fanningerstraße 32, 10365 Berlin

PI: Prof. Dr. med. Fabian Knebel

Deputy PI: Prof. Dr. med. Adrian Constantin Borges

Investigators: Dr. Niki Spyrantis, Dr. Aleksandar Aleksandrov, Dr. Sven Tessin, Monique Feist

Bonn University Hospital, Herzzentrum Venusberg-Campus 1, 53127 Bonn

PI: Prof. Dr. med. Georg Nickenig

Deputy PI: Univ.-Prof. Dr. med. Sebastian Zimmer

Investigators: Dr. med. Margarita Schulz, Dr. med. Johanna Vogelhuber, Dr. med. Dominik Nelles, Dr. med Leonida Tusha, Sarah Simon, Manuela Levis , Franziska Wienberg

Munich Bogenhausen Hospital, Academic Teaching Hospital of the TU Munich, Englschalkinger Str. 77, 81925 München

PI: PD. Dr. med. Florian Straube

Deputy PI: Prof. Dr. med. Ellen Hoffmann

Investigators: Dr. med. univ. Alexander Mohl, Annika El Gamal

Coburg Hospital, Ketschendorfer Str. 33, 96450 Coburg

PI: Dr. med. Steffen Schnupp

Deputy PI: Dr. med. Mathias Forkmann

Investigators: Dr med. Christian Mahnkopf, Ute Göbel, Swetlana Rube, Andrea Lins, Petra Sünkel, Thomas Mischke, Mhd Nawar Alachkar

Constance Hospital, Mainaustr. 35, 78464 Konstanz

PI: Dr. med. Nikolaos Dagkonakis

Deputy PI: Dr. med. Ludwig Bernd

Investigator: Ulrike Petzsche, Ute Ettl, Aleksandra Gvozdenac

Friedrichshafen Hospital, Röntgenstraße 2, 88048 Friedrichshafen

PI: PD. Dr. med. Julia Seeger

Deputy PI: Mario Eduardo Itrube Orbe

Investigators: Rebecca-Ann Garman, Sandra Kramer

Ingolstadt Hospital, Krumenauerstraße 25, 85049 Ingolstadt

PI: PD. Dr. med. Luani Blerim

Deputy PI: Seudin Hasic,

Investigators: Dr. med. Dan Liviu Pascut, Hogir Housen. Dr. med. Vjosa Thaci-Kadriu, Ndricim Tobli, Silke Gläser, Ioana Pascut

Boards

Sponsor: Ludwig Maximilians University hospital Munich (LMU university hospital)

Principal investigators (PI)

Prof. Dr. med. Steffen Massberg (LMU University Hospital Munich)

Prof. Dr. med. Konstantinos D. Rizas (LMU University Hospital Munich and Kantonsspital St. Gallen, St. Gallen, Switzerland)

Prof. Dr. med. Ulrich Mansmann (Institute for Medical Information Processing, Biometry, and Epidemiology, IBE)

Steering Committee (SC)

Prof. Dr. med. Steffen Massberg (Chair, LMU University Hospital Munich)

Prof. Dr. med. Konstantinos D. Rizas (Co-ordinating investigator LMU University Hospital Munich and Kantonsspital St. Gallen, St. Gallen, Switzerland)

Prof. Dr. med. Ingo Eitel (University Heart Center Lübeck)

Prof. Dr. med. Thomas Stiermaier (University Heart Center Lübeck)

Prof. Dr. Adnan Kastrati (TUM University Hospital German Heart Center)

Prof. Dr. med. Dirk Sibbing (Privatklinik Lauterbacher Mühle am Ostersee)

Prof. Dr. med. Paulus Kirchhof (Universitäres Herz- und Gefäßzentrum UKE Hamburg)

Prof. Dr. med. Christoph Stellbrink (Klinikum Bielefeld)

Prof. Dr. med. Holger Thiele (Herzzentrum Leipzig)

Prof. Dr. med. Ulf Landmesser (Campus Benjamin Franklin, Charité – Universitätsmedizin Berlin)

PD Dr. med. Marc Kollum (Hegau-Bodensee-Klinikum Singen)

Data Safety and Monitoring Board (DSMB)

Prof. Dr. med. Nikolaus Marx (Chair, Uniklinik RWTH Aachen, Germany)

Prof. Dr. Christine Stefanie Mayer Zürn (University hospital Basel, Switzerland)

Prof. Dr. Efstratios Charitos (Kerckhoff-Klinik, Bad Nauheim, Germany)

Endpoint Adjudication Committee (EAC)

Prof. Dr. med. Björn Krämer (Chair, Kreisklinik Ebersberg, Germany)

Prof. Dr. med. Bruno Huber (Herz-Gefäss-Zentrum Munich)

Dr. med. Wolfgang Hamm (Klinikum Garmisch-Partenkirchen, Germany)

Projekt Management:

Ludwig-Maximilians-University Hospital Munich

Thefoanis Korovesis

Dr. Lukas von Stuelpnagel

Dr. Elodie Eiffener

Lisa Kretz

Monika Baylacher

ISAResearch Center - TUM University Hospital German Heart Center

Dr. Tanja Ulrich

Sarah Lang

Dr. Marion Janisch

Dr. Stefanie Brunner

Julia Horst

Data Management: ISAResearch Center - TUM University Hospital German Heart Center

Coordinating Research Center

ISAResearch Center – TUM University Hospital German Heart Center

Prof. Dr. Adnan Kastrati

Dr. Marion Janisch

Dr. Tanja Ulrich

Sarah Lang

Dr. Stefanie Brunner

Julia Horst

Marjorie Grimm

Valentina Meyer

Barbara von Merzljak

Dr. Inge Berthold

Franziska Prömm

Monitoring: ISAResearch Center - TUM University Hospital German Heart Center

Dr. Beate May, Irena Schumacher, Andrea Mecklenburg, Jana Reins, Dr. Andreas Finzer, Kerstin Vogel, Sabine Volk, Carola Würfel, Heidrun Hofmann-Henkel

Biometry Statistics:

Prof. Dr. med. Ulrich Mansmann (Institute for Medical Information Processing, Biometry, and Epidemiology, IBE)

Amani Al Tawil (Institute for Medical Information Processing, Biometry, and Epidemiology, IBE)

Prof. Dr. med. Konstantinos D. Rizas (LMU University Hospital Munich and Kantonsspital St. Gallen, St. Gallen, Switzerland)

Pharmacovigilance/Safety Management: ISAResearch Center - TUM University Hospital German Heart Center

Supplementary Tables

Supplementary Table 1: Demographic, clinical, and angiographic characteristics stratified by randomization group in the per-protocol set.

**Characteristics**	**Experimental group**	**Control group**	**Total**
N	278	298	576
**Demographic characteristics**			
Age – years (Q1 – Q3)	78 (70 – 83)	78 (68 – 83)	78 (69 – 83)
Female sex – N/total N (%)	77 / 278 (27.7%)	67 / 298 (22.5%)	144 / 576 (25.0%)
Diabetes mellitus – N/total N (%)	104 / 278 (37.4%)	95 / 298 (31.9%)	199 / 576 (34.5%)
Arterial hypertension – N/total N(%)	241 / 275 (87.6%)	251 / 297 (84.5%)	492 / 572 (86.0%)
Hypercholesterolemia – N/total N	171 / 273 (62.6%)	192 / 293 (65.5%)	363 / 566 (64.1%)
Active smoker – N/total N (%)	50 / 278 (18.0%)	49 / 298 (16.4)	99 / 576 (17.2%)
Family history of CAD – N/total N	69 / 233 (29.6%)	75 / 251 (29.9%)	144 / 484 (29.8%)
**Clinical characteristics**			
Previous MI – N/total N (%)	78 / 278 (28.1%)	77 / 293 (26.3%)	155 / 571 (27.1%)
Previous PCI – N/total N (%)	118 / 276 (42.8%)	126 / 295 (42.7%)	244 / 571 (42.7%)
Previous CABG – N/total N (%)	23 / 278 (8.3%)	22 / 298 (7.4%)	45 / 576 (7.8%)
Previous stroke – N/total N (%)	10 / 278 (3.6%)	21 / 298 (7.0%)	31 / 576 (5.4%)
Atrial fibrillation N/total N (%)			
- New diagnosis	72 / 271 (26.6%)	79 / 293 (27.0%)	151 / 564 (26.8%)
- Paroxysmal	122 / 271 (45.0%)	131 / 293 (44.7%)	253 / 564 (44.9%)
- Persistent	45 / 271 (16.6%)	59 / 293 (20.1%)	104 / 564 (18.4%)
- Long-standing persistent	3 / 271 (1.1%)	0 / 293 (0.0%)	3 / 564 (0.5%)
- Permanent	29 / 271 (10.7%)	24 / 293 (8.2%)	53 / 564 (9.4%)
MI type N/total (%)			
- STEMI	55 / 278 (19.8%)	59 / 298 (19.8%)	114 / 576 (19.8%)
- NSTEMI	223 / 278 (80.2%)	239 / 298 (80.2%)	462 / 576 (80.2%)
LVEF – N/total N (%)			
- ≥ 50%	134 / 270 (49.6%)	147 / 292 (50.3%)	281 / 562 (50.0%)
- 40 – 49%	69 / 270 (25.6%)	77 / 292 (26.4%)	146 / 562 (26.0%)
- 30 – 39%	43 / 270 (15.9%)	40 / 292 (13.7%)	83 / 562 (14.8%)
- < 30%	24 / 270 (8.9%)	28 / 292 (9.6%)	52 / 562 (9.2%)

NYHA, N/total N (%)<br>- I<br>- II<br>- III<br>- IV	 123 / 276 (44.6%)<br>50 / 276 (18.1%)<br>61 / 276 (22.1%)<br>42 / 276 (15.2%)	 141 / 298 (47.3%)<br>55 / 298 (18.5%)<br>73 / 298 (24.5%)<br>29 / 298 (9.7%)	 264 / 574 (46.0%)<br>105 / 574 (18.3%)<br>134 / 574 (23.3%)<br>34 / 574 (12.4%)
CCS, N/total N (%)<br>- 0<br>- I<br>- II<br>- III<br>- IV	 97 / 275 (35.3%)<br>10 / 275 (3.6%)<br>28 / 275 (10.2%)<br>59 / 275 (21.5%)<br>81 / 275 (29.4%)	 88 / 295 (29.8%)<br>15 / 295 (5.1%)<br>37 / 295 (12.5%)<br>40 / 295 (13.6%)<br>115 / 295 (39.0%)	 185 / 570 (32.5%)<br>25 / 570 (4.4%)<br>65 / 570 (11.4%)<br>99 / 570 (17.4%)<br>196 / 570 (34.4%)
Bleeding predisposition – N/total N (%)	13 / 276 (4.7)	17 / 295 (5.8%)	30 / 571 (5.3%)
CHA_2DS_2-VA Score – Score (Q1 – Q3)	4 (3 – 5)	4 (3 – 4)	4 (3 – 5)
HAS-BLED Score – Score (Q1 – Q3)	3 (3 – 3)	3 (2 – 3)	3 (3 – 3)
PAOD – N/total N (%)	30 / 277 (10.8%)	30 / 297 (10.1%)	60 / 574 (10.5%)
Chronic Renal Failure – N/total N (%)	67 / 278 (24.1%)	81 / 298 (27.2%)	148 / 576 (25.7%)
Diseased vessels, N/total N (%)<br>- 1<br>- 2<br>- 3	 71 / 278 (25.5%)<br>73 / 278 (26.3%)<br>134 / 278 (48.2%)	 74 / 298 (24.8%)<br>82 / 298 (27.5%)<br>142 / 298 (47.7%)	 145 / 576 (25.2%)<br>155 / 576 (26.9%)<br>276 / 576 (47.9%)
**Interventional characteristics**			
Access site, N/total N (%)<br>- Radial<br>- Femoral<br>- Other	 201 / 278 (72.3%)<br>74 / 278 (26.6%)<br>3 / 278 (1.1%)	 228 / 298(76.3%)<br>67 / 298 (22.7%)<br>3 / 298 (1.0%)	 429 / 576 (74.8%)<br>141 / 576 (24.3%)<br>6 / 576 (1.0%)
Culprit lesion, N/total N (%) *<br>- LM<br>- LAD<br>- LCx<br>- RCA<br>- Bypass graft	 13 / 278 (4.7%)<br>114 / 278 (41.0%)<br>73 / 278 (26.3%)<br>89 / 278 (32.0%)<br>4 / 278 (1.4%)	 14 / 298 (5.0%)<br>127 / 298 (42.6%)<br>79 / 298 (26.5%)<br>91 / 298 (30.5%)<br>2 / 298 (0.7%)	 27 / 576 (4.7%)<br>241 / 576 (41.8%)<br>152 / 576 (26.4%)<br>180 / 576 (31.2%)<br>6 / 576 (1.0%)
TIMI III flow after procedure – N/total N (%)	264 / 278 (95.0%)	282 / 298 (94.6%)	546 / 576 (94.8%)
Ostial PCI – N/total N (%)	46 / 278 (16.5%)	55 / 298 (18.5%)	101 / 576 (17.5%)
Bifurcation PCI – N/total N (%)	42 / 278 (15.1%)	59 / 298 (19.8%)	101 / 576 (17.5%)
Multivessel PCI – N/total N (%)	50 / 278 (18.0%)	57 / 298 (19.1%)	107 / 576 (18.6%)
Number of stents, median (Q1 – Q3)	2 (1 – 2)	1 (1 – 2)	1 (1 – 2)
Length of stents, mm (Q1 – Q3)	28 (18 – 41)	28 (18 – 44)	28 (18 – 43)

Medication			
ASA on admission	54 / 275 (19.6%)	69 / 295 (23.4%)	123 / 570 (21.6%)
P2Y12-inhibitors on admission	30 / 277 (10.8%)	46 / 295 (15.6%)	76 / 570 (13.3%)
OAC on admission	169 / 276 (61.2%)	183 / 296 (61.8%)	352 / 572 (61.5%)

***** Some patients presented with more than one culprit lesions.

Only available data have been used for calculation of proportions.

ASA: Aspirin; CAD: coronary artery disease; CCS: Canadian Cardiovascular Society Angina score; CHA_2DS_2-VA score: congestive heart failure, hypertension, age ≥75 (doubled), diabetes, stroke (doubled), vascular disease, age 65 to 74; HAS-BLED score: hypertension, abnormal renal and/or liver function, stroke, bleeding history or predisposition, labile INR, elderly (age > 65 years), drugs; LAD: left anterior descending artery; LCX: left circumflex artery; LVEF: left-ventricular ejection fraction; LM: left main coronary artery; NYHA classification: New York Heart Association classification; NSTEMI: non-ST elevation myocardial infarction; MI: myocardial infarction; OAC: oral anticoagulation; PAOD: peripheral arterial occlusive disease; PCI: percutaneous coronary intervention; Q1: lower quartile; Q3: upper quartile; RCA: right coronary artery; STEMI: ST-elevation myocardial infarction; TIMI: thrombolysis in myocardial infarction

Data and Safety Monitoring Board

The independent Data and Safety Monitoring Board (DSMB) consisted of three independent experts and met at regular intervals during the trial. DSMB meetings comprised open and closed sessions. During the open session, trial investigators could provide information regarding trial conduct, including site initiation, recruitment, and protocol amendments. The closed session was restricted to DSMB members and included review of accumulating safety and outcome data and formulation of a recommendation regarding continuation, modification, or termination of the trial.

The DSMB monitored adverse events and serious adverse events, as well as deaths, myocardial infarctions, stent thromboses, ischemic strokes, systemic thromboembolic events, and bleeding events of class ≥ 2, according to the Bleeding Academic Research Consortium criteria. For these reviews, the sponsor provided the DSMB with unfiltered, unprocessed and unadjudicated data.

No formal interim efficacy or safety analyses were planned. The DSMB was permitted to review and compare event frequencies between treatment groups; however, formal statistical hypothesis testing was not performed, as this would have constituted an interim analysis. DSMB recommendations were therefore based primarily on a qualitative assessment of the totality of the accumulating safety data.

The only prespecified quantitative stopping criterion concerned all-cause mortality. This criterion was met if the hazard ratio for mortality exceeded 1.3 in either direction, together with an absolute difference of >10 deaths between treatment groups. Independently of this criterion, the DSMB was authorized to recommend termination of the trial if other concerning safety signals emerged.

The first DSMB meeting was held on December 16, 2022, after which the DSMB recommended continuation of the trial without additional concerns.

The second DSMB meeting was held on December 14, 2023. The DSMB recommended continuation of the trial with particular attention to ischemic stroke events. Thereafter, in addition to serious adverse events the DSMB received monthly reports of updates on ischemic strokes.

The third DSMB meeting was held on September 19, 2025. At that time, the prespecified mortality stopping criterion had not been met: the hazard ratio for mortality was 1.3, whereas the absolute difference between treatment groups was five deaths (21 v. 16). The DSMB also considered the individual components of the efficacy endpoint. At that time, ischemic strokes (6 vs. 1), total strokes (ischemic and hemorrhagic, 7 vs. 1), and systemic embolic events (1 vs. 0) were numerically more frequent in the experimental group, whereas myocardial infarctions (13 vs. 14), stent thromboses (4 vs. 5), and urgent revascularizations (7 vs. 8) were only marginally less frequent. In addition, bleeding events were more frequent in the experimental group (103 vs. 76). Based on the totality of these accumulating data, the DSMB considered the likelihood of demonstrating superiority of the experimental intervention to be low and identified a concerning safety signal. The DSMB therefore recommended termination of the trial, stating: “Based on the low likelihood of superiority of the experimental intervention along with the safety signal with an increased number of deaths, strokes, and bleedings in the experimental group the DSMB recommends termination of the trial.”

The DSMB report referred to 604 randomized participant IDs. The discrepancy from the final randomized population of 602 participants resulted from two IDs that did not correspond to unique randomized participants: one resulted from an erroneous randomization at a study site and the other from duplicate randomization caused by an eCRF error. Both records were identified and removed during the data review meeting.

Following consultation with the Steering Committee, the sponsor (LMU University Hospital, Munich) accepted the DSMB recommendation and immediately discontinued further recruitment.

The DSMB charter and records of the DSMB recommendations are provided in the Supplementary Note 3.
