# Supplementary material for: Dual antithrombotic therapy using potent antiplatelet inhibitors in atrial fibrillation and acute coronary syndrome: a randomized controlled trial
Source: Nat Med. 2026 Aug 29;32(11):4036–46. doi: 10.1038/s41591-026-04629-7 (PMC13768177; doi:10.1038/s41591-026-04629-7)
Supplement: Supplementary file 2 [file 41591_2026_4629_MOESM2_ESM.pdf]

nature portfolio

Corresponding author(s): Konstantinos D. Rizas and Steffen Massberg

Last updated by author(s): Aug 5, 2026

Reporting Summary

Nature Portfolio wishes to improve the reproducibility of the work that we publish. This form provides structure for consistency and transparency in reporting. For further information on Nature Portfolio policies, see our Editorial Policies and the Editorial Policy Checklist.

Statistics

For all statistical analyses, confirm that the following items are present in the figure legend, table legend, main text, or Methods section.

n/a	Confirmed	
☐	☒	The exact sample size (*n*) for each experimental group/condition, given as a discrete number and unit of measurement
☒	☐	A statement on whether measurements were taken from distinct samples or whether the same sample was measured repeatedly
☐	☒	The statistical test(s) used AND whether they are one- or two-sided<br>*Only common tests should be described solely by name; describe more complex techniques in the Methods section.*
☐	☒	A description of all covariates tested
☐	☒	A description of any assumptions or corrections, such as tests of normality and adjustment for multiple comparisons
☐	☒	A full description of the statistical parameters including central tendency (e.g. means) or other basic estimates (e.g. regression coefficient) AND variation (e.g. standard deviation) or associated estimates of uncertainty (e.g. confidence intervals)
☐	☒	For null hypothesis testing, the test statistic (e.g. *F*, *t*, *r*) with confidence intervals, effect sizes, degrees of freedom and *P* value noted<br>*Give P values as exact values whenever suitable.*
☒	☐	For Bayesian analysis, information on the choice of priors and Markov chain Monte Carlo settings
☐	☒	For hierarchical and complex designs, identification of the appropriate level for tests and full reporting of outcomes
☒	☐	Estimates of effect sizes (e.g. Cohen's *d*, Pearson's *r*), indicating how they were calculated

Our web collection on statistics for biologists contains articles on many of the points above.

Software and code

Policy information about availability of computer code

Data collection	Patients were prospectively enrolled and randomized. Data was entered into a certified electronic Case Report Form (eCRF)
Data analysis	All statistical analyses were performed using CRAN R, version 4.6.0 and following packages: survival (v. 3.8.6), coxphf (v 1.13.4), ggplot2 (v 4.0.3) and survminer (v. 0.5.2). All scripts can be accessed via https://osf.io/mc46d/overview?view_only=1c85943857a844e19ee5d78c84f82c58

For manuscripts utilizing custom algorithms or software that are central to the research but not yet described in published literature, software must be made available to editors and reviewers. We strongly encourage code deposition in a community repository (e.g. GitHub). See the Nature Portfolio guidelines for submitting code & software for further information.

Data

Policy information about availability of data

All manuscripts must include a data availability statement. This statement should provide the following information, where applicable:

- Accession codes, unique identifiers, or web links for publicly available datasets
- A description of any restrictions on data availability
- For clinical datasets or third party data, please ensure that the statement adheres to our policy

Upon request, investigators can be given access to anonymized raw data for further scientific projects provided the positive evaluation of the steering committee, the local ethics committee of the Ludwig-Maximilians university Munich and the data protection officer of the LMU University hospital. Free availability of raw data

is not possible due to legal restrictions. Requests for data availability can be made to the corresponding author (Prof. Konstantinos Rizas, konstantinos.rizas@h-och.ch). The expected response time is 2 weeks, and the decision-making process takes 3 months.

Research involving human participants, their data, or biological material

Policy information about studies with human participants or human data. See also policy information about sex, gender (identity/presentation), and sexual orientation and race, ethnicity and racism.

Reporting on sex and gender	The participant's gender was determined by self-report. Pre-specified subgroup analyses stratified by sex are reported.
Reporting on race, ethnicity, or other socially relevant groupings	N/A - Race, ethnicity or other social variables were not routinely assessed and were not pre-defined as subgroup analyses of interest.
Population characteristics	The median age of the population was 78, IQR 14 years, 25.6% of patients were female.
Recruitment	Patients presenting with acute coronary syndrome and atrial fibrillation were screened for eligibility. Those meeting the inclusion criteria and not the exclusion criteria were informed about the trial, and those who provided informed consent were randomized using internet-based software.
Ethics oversight	The trial was approved by the local ethics committee of the Ludwig-Maximilians university Munich (number 21-0807 fed), at all participating centers and by the legal authorities (German Federal Institute for Drugs and Medical Devices - BfArM and Austrian Federal Office for Safety in Health Care- BASG). The study was performed in accordance with the Declaration of Helsinki and Good Clinical Practice principles. The trial was overseen by a steering committee and a data and safety monitoring board (DSMB).

Note that full information on the approval of the study protocol must also be provided in the manuscript.

Field-specific reporting

Please select the one below that is the best fit for your research. If you are not sure, read the appropriate sections before making your selection.

☒ Life sciences ☐ Behavioural & social sciences ☐ Ecological, evolutionary & environmental sciences

For a reference copy of the document with all sections, see nature.com/documents/nr-reporting-summary-flat.pdf

Life sciences study design

All studies must disclose on these points even when the disclosure is negative.

Sample size	The trial was designed to detect superiority of the experimental compared to the control treatment with respect to the efficacy endpoint and non-inferiority with respect to the safety endpoint using a hierarchical testing. The analysis of both endpoints was planned to take place using the Finkelstein and Schoenfeld win-loss-ratio (WLR) according to the defined ranking of endpoints in the outcomes section. For sample size calculation we used bootstrapping and 1,000 iterations. For the efficacy endpoint we found that a two-sided alpha of 0.05 and 6-week hazard-rates and standard errors of hazard rates according to the values provided in Supplementary Table 2, the power would be 80.0%, if comparison of WLR would be performed on a total sample size of 615 per group. To account for dependency between efficacy endpoints the sample size was increased by 10%, according to previous suggestions23, which led to a calculated sample size of 677 per group. For the safety endpoint we assumed an effect size of 0.95 and a non-inferiority delta of 30%. We found that with a total sample size of 695 per group, a two-group large-sample normal approximation test of proportions with a one-sided 2.5% significance level would have 80% power to reject the null hypothesis that the lower 97.5% confidence interval of the WLR compared between the experimental and control arm is lower than 0.77 (1/[1 + delta], with delta = 0.3) in favor of the alternative hypothesis, that the lower 97.5% confidence interval is greater than or equal to 0.77. For safety endpoints no dependency between the endpoints was considered, as the endpoints of the safety analysis are mutually exclusive. The sample size of the trial was given by the maximum of both sample sizes, i.e. 695 per group. Assuming an allocation ratio ncontrol/ninterventional =1.0 and a 6% drop-out rate, the sample study of the study was calculated to be N =1,474.
Data exclusions	Participants providing informed consent were included in the study. Efficacy endpoints were analyzed according to the intention-to-treat principle in the full analysis set. Safety endpoints were analyzed using the modified intention to treat principle in a per-protocol set. Per protocol set included randomized patients who received treatment according to the randomized treatment strategy and who had no major protocol deviation judged before database lock during the data review meeting.
Replication	We performed several sensitivity analyses against bias: Analyses of both efficacy and safety endpoints according to the intention-to-treat, per-protocol and as-treated principles. We also performed provisional power analysis simulating the original sample size. We confirm that all tests for replication of our results were successful.
Randomization	Randomization was internet-based and was performed with the use of blocks of block size 8 and was stratified by center.
Blinding	Investigators and participants were aware of the study group allocation. The Endpoint Adjudication Committee was blinded to the treatment allocation.

Reporting for specific materials, systems and methods

We require information from authors about some types of materials, experimental systems and methods used in many studies. Here, indicate whether each material, system or method listed is relevant to your study. If you are not sure if a list item applies to your research, read the appropriate section before selecting a response.

Materials & experimental systems

n/a	Involved in the study
☒	☐ Antibodies
☒	☐ Eukaryotic cell lines
☒	☐ Palaeontology and archaeology
☒	☐ Animals and other organisms
☐	☒ Clinical data
☒	☐ Dual use research of concern
☒	☐ Plants

Methods

n/a	Involved in the study
☒	☐ ChIP-seq
☒	☐ Flow cytometry
☒	☐ MRI-based neuroimaging

Clinical data

Policy information about clinical studies

All manuscripts should comply with the ICMJE guidelines for publication of clinical research and a completed CONSORT checklist must be included with all submissions.

Clinical trial registration: ClinicalTrials.gov identifier: NCT04981041

Study protocol: Version 1.0 (30-Mar-2021), Version 2.0 (21 Apr-2021), Version 3.0 (27 May-2021), Version 4.0 (27 Oct-2021), Version 5.0 (23 Dec-2021), Version 6.0 EU (20 Mar-2024), Version 7.0 EU (10-Jun-2024),Version 8.0 EU (05-Jan-2025). All information are available in the Supplementary Materials.

Data collection: Data collection was performed with site visits and telephone calls. Data collection was executed using a certified eCRF.

Outcomes: The primary efficacy endpoint of the study was defined as the composite of (i) all-cause mortality, (ii) definite or probable stent thrombosis, (iii) MI, (iv) ischemic stroke, (v) systemic thromboembolism, and (vi) urgent revascularization within 6 weeks after randomization. The composite safety endpoint was defined as the composite of (i) death, (ii) BARC 3c, (iii) BARC 3b, (iv) BARC 3a and (v) BARC 2 bleeding. All components of both primary endpoints were adjudicated blindly by an independent endpoint adjudication committee consisting of three experienced cardiologists. Secondary endpoints included the primary endpoints evaluated at 6 months, all individual components of the primary endpoints evaluated at 6 weeks and 6 months, unplanned hospitalization due to ACS or acute decompensated heart failure at 6 months, and all bleeding complications (including BARC 1 bleeding) at 6 weeks and 6 months.

Plants

Seed stocks: NA

Novel plant genotypes: NA

Authentication: NA
