# Supplementary material for: Dual antithrombotic therapy using potent antiplatelet inhibitors in atrial fibrillation and acute coronary syndrome: a randomized controlled trial
Source: Nat Med. 2026 Aug 29;32(11):4036–46. doi: 10.1038/s41591-026-04629-7 (PMC13768177; doi:10.1038/s41591-026-04629-7)
Supplement: Supplementary file 3 [file 41591_2026_4629_MOESM3_ESM.pdf]

Deutsches Herzzentrum München
des Freistaates Bayern
Klinik an der Technischen Universität München

Data Safety Monitoring Board Charter
# – Written Operating Procedures –

Study:	**Escalated single platelet inhibition for one month plus direct oral anticoagulation in patients with atrial fibrillation and acute coronary syndrome undergoing percutaneous coronary intervention (EPIDAURUS)**
Study Short Title:	EPIDAURUS
Protocol Code:	EPIDAURUS2020
Sponsor:	LMU University Hospital Munich<br>Univ.-Prof. Dr. med. Steffen Massberg (Sponsor Delegated Person)<br>Department of Cardiology<br>Marchioninistr. 15, 81377 Munich, Germany
Version / Date:	V1.0 / 23-JUL-2019
Replaced Version / Date:	Not applicable

Version History

Version No.:	Effective date	Changes performed
NA	NA	Initial Version

Approval:

Prepared by

23 JUL 2021
Date

Signature [Dr. med. Janisch, Marion, DHM, ISAResearch Center/ PM CRO]

Reviewed by

23 JUL 2021
Date

Signature [PD Dr. med. Rizas, Konstantinos, KUM, Cardiology / Coordinating Investigator]

Authorized by

23. Juli 2021
Date

Signature [Prof. Dr. med. Massberg, Steffen, KUM, Cardiology / Sponsor Delegated Person]

Table of Contents

1 Summary 4
2 DSMB Composition 4
3 DSMB Responsibilities 5
4 Meeting Schedule 5
5 Meeting Format and Documentation 6
6 Study Review Criteria and Stopping Rules 7
7 Interim Analysis 7
8 Data and Data Transfer 7
9 Sponsor's Response to DSMB Findings and Recommendations 8
10 Conflicts of Interest and Confidentiality 8
11 Amendment to this Charter 9
12 Signature List 9
13 Abbreviations 9
14 Attachments 10
14.1 Attachment 1: Data Review Timeline 10

1 Summary

This document summarizes all aspects of the Data Safety Monitoring Board (DSMB) organized by the sponsor of the trial and the ISAResearch Center (CRO) for the EPIDAURUS trial. The DSMB is the primary data and safety advisory board for this study. The DSMB reviews study data, assesses the differences in safety between treatment and control groups, monitors for excessive occurrence of adverse events and makes recommendations to the sponsor delegated person regarding safety issues, risks to research participants, as well as the continuing validity and scientific merit of the study. The Chairman of the DSMB notifies the sponsor regarding continuation of enrollment, modification of the protocol, temporary suspension or termination of the trial.

In addition to this charter, the DSMB members will receive the current study protocol and SMPCs of the investigational medicinal products as well as any subsequent amendments thereof from the ISAResearch Center (CRO).

2 DSMB Composition

The DSMB is composed of 3 members. They are:

1.	name	Univ.-Prof. Dr. med. Nikolaus Marx (Chairman)
	address	Pauwelsstraße 30, 52074 Aachen, Germany
	institution	Universitätsklinikum Aachen, Klinik für Kardiologie, Angiologie und Internistische Intensivmedizin (Med. Klinik I)
	telephone	+49 241 80-89300
	fax	+49 241 80-82545
	e-mail	nmarx@ukaachen.de
1.	name	Prof. Christine Meyer-Zürn
	address	Petersgraben 4, 4031 Basel, Switzerland
	institution	Universitätsspital Basel, Klinik für Kardiologie
	telephone	+41 61 328 52 64
	fax	not applicable
	e-mail	christine.meyerzuern@usb.ch
3.	name	PD Dr. Dr. med. Efstratios Charitos
	address	Venusberg-Campus 1, 53127 Bonn, Germany
	institution	Herzzentrum des Universitätsklinikums Bonn
	telephone	+49 228 28714092
	fax	not applicable
	e-mail	Efstratios.Charitos@ukbonn.de

All members have the required training, experience and expertise in their field of practice and in the conduct of clinical trials.

Each member of the DSMB is expected to serve for the full duration of the trial. In the unlikely event that a DSMB member is unable to continue participation on the DSMB, the sponsor will select a replacement adhering to the relevant criteria.

3 DSMB Responsibilities

The DSMB advises and supports the sponsor and the Coordinating Investigator with regard to patients' safety in this clinical trial. For this purpose, the DSMB regularly evaluates the results of safety analyses and its influence on the risk-benefit-assessment for the patients and the justifiability of further application of the study therapy. Moreover, the DSMB evaluates the validity and integrity of the data and thereby the study conduct.

At periodic time intervals during the course of the study (see Appendix 1, Date Review Timetable) the DSMB should discuss the previous course of the clinical trial under the following aspects:

a) Evaluate events for evidence of safety concerns
    - accumulation of adverse events and serious adverse events / unacceptable toxicity
    - critical efficacy endpoints, all-cause mortality, myocardial infarction, definite or probable stent thrombosis, ischaemic stroke and systemic thromboembolism, Bleeding type 2 or higher according to the Bleeding Academic Research Consortium (BARC) criteria
    - suspected unexpected serious adverse reaction (SUSAR) that may lead to a change in the risk-to-benefit assessment
    - other changes in the risk-to-benefit assessment

b) Evaluate the study progress with projections of recruitment and timelines for study milestones. This also includes evaluation of any significant trends of performance that may influence the safety or conduct of the study.

c) Advise the sponsor with regard to potential problem areas within the protocol regarding procedures, definitions or endpoints allowing timely notification of the regulatory authorities for protocol revisions.

Based on the data presented to the DSMB the DSMB recommends continuation, modification, temporary suspension or termination of the clinical trial after every regular meeting.

Reasons for the recommendation of study termination must be explained in detail. The decision for study termination should preferably be made by agreement between the sponsor delegated person and the DSMB. However, the final decision about study termination rests with the Steering Committee.

4 Meeting Schedule

Before start of enrollment, there will be a constituent meeting in order to:

- Discuss and finalize this DSMB charter
- Form an understanding of the study protocol
- Review the potential risks to research subjects
- Discussion of potential study modification and/or termination guidelines
- Review and approve the tables and listings to be used for reporting to the DSMB.

- Review the Data Review Timeline (see attachment 1)

The ISAResearch Center (CRO) prepares the agenda and organizes the DSMB meetings.

During enrollment phase, formal reviews will take place after the Control Visit (Day 42±7) follow-up of the first 250 patients have been completed. Subsequent meetings will be scheduled after completion of the 42 day follow-up of 1000 and 2000 patients.

Notwithstanding the above, the DSMB will meet at least every 12 months during the recruitment phase.

Ad-hoc meetings to discuss any emerging safety issue may be convened by any member of the DSMB or the sponsor delegated person at any time.

5 Meeting Format and Documentation

According to this agreement meetings will be organized by project management of the ISAResearch Center (CRO).

By default, meetings will be conducted as teleconferences or videoconferences. Face to face meetings may also take place.

A quorum of 2 members, including the Chairman, must be present to hold a DSMB meeting. Critical decisions of the DSMB should be made by unanimous vote. However, if this is not possible, majority vote will decide. In case that only 2 members are attending a DSMB meeting and there is disagreement between these 2 members the third member has to be consulted who has to vote as well.

The DSMB Meetings are divided into two parts. At the open part, in which e.g. enrollment data, individual AE data, baseline characteristics, overall data accuracy, compliance data and administrative data are discussed, personnel from the sponsor or investigators may be present. This is followed by a closed part for the review of critical information, e.g. unblinded patient data, data that may compromise the study integrity (e.g. comparative data), which is restricted to DSMB members only.

A closed session is only required if deemed necessary by the DSMB.

The results of the open session are documented in writing. Personnel from the ISAResearch Center (CRO) may assist in writing of this protocol. After approval and signature by the DSMB Chair open session protocols are stored in the trial master file (TMF) with the sponsor.

A report about the closed session is written by the DSMB Chair or if agreed between the DSMB members by one of the two other DSMB members. The report must be signed by the DSMB Chair and rests with the DSMB Chair until the end of the study. Afterwards, it will be stored in the TMF with the sponsor.

After every session, the DSMB makes a recommendation regarding study continuation. The sponsor delegated person is informed about this recommendation in writing. The recommendation shall be sent by the DSMB Chair to the sponsor delegated person within 10 business days after the meeting. If the findings of the DSMB include serious and potentially consequential recommendations that require immediate action, the DSMB Chair promptly notifies the sponsor delegated person within 3 business days after the meeting at the latest.

6 Study Review Criteria and Stopping Rules

The DSMB monitors:

- Adverse Events (AE), Serious Adverse Events (SAE) and Suspected Unexpected Serious Adverse Reactions (SUSAR)
- In addition, the following study endpoints are reported to the DSMB:
  - All-cause mortality
  - Myocardial infarction
  - Definite or probable stent thrombosis
  - Ischaemic stroke
  - Systemic thromboembolism
  - Bleeding BARC type 2 or higher
- Protocol deviations, compliance with IMP intake and follow-ups
- Recruitment

Information on SAEs is provided monthly to all DSMB members by the PV management of the ISAResearch Center (CRO). SUSARs are reported to the DSMB at the same time as they are reported to the regulatory authorities.

The assessment of events is based on absolute rates, differences in rates and the development of SAE/SUSAR over time.

As stopping rule is defined a difference in hazard rates of 30% or more (HR between experimental and control arm or between control and experimental arm > 1.3) with a concomitant difference in absolute deaths of > 10.

Termination or modification of the trial may be recommended for any other perceived safety concern based on clinical judgement, including, but not limited to, a higher than anticipated rate of any clinical endpoint or SUSAR.

7 Interim Analysis

There is no formal interim analysis planned according to the study protocol.

8 Data and Data Transfer

In order to provide the maximum amount of information to the DSMB, the analyses employ the most recent data available at that time point (recognizing the limitations thereof).

All clinical endpoints in this trial are referred to the event adjudication committee (EAC) for adjudication. The DSMB reports include adjudicated data whenever possible. If time does not allow for EAC review prior to a DSMB meeting, unadjudicated data are presented. Should the DSMB need additional information or a rationale for EAC decisions, the DSMB Chairman notifies the PV Team of the ISAResearch Center accordingly.

Data that were received by data management within 5 weeks before the scheduled DSMB meeting should have been cleaned by query management.

Approximately one week prior to the date of the DSMB meeting, prepared data in form of an extract of the study database and of the pharmacovigilance database are made available electronically for all members of the DSMB by PV management of the ISAResearch Center (CRO).

The DSMB tables provided by the ISAResearch Center (CRO) include:

- number of patients enrolled
- AEs, SAEs, SUSAR, clinical endpoints
- protocol deviations
- compliance with IMP intake and follow-up

After statistical analysis and evaluation of the data by the DSMB statistician, the other DSMB members receive the data from the DSMB statistician for further analysis and evaluation.

9 Sponsor's Response to DSMB Findings and Recommendations

If the DSMB recommends continuation of the study without modification, no formal response is required. However, if the recommendations request action such as a recommendation for termination of the study or modification of the protocol, the DSMB requests that the sponsor delegated person provides a formal written response stating whether the recommendations will be followed and the plan for addressing the issues.

In general, the considerations of the DSMB are not legally binding, but require professional consideration by the sponsor delegated person. The sponsor delegated person may need to consult with regulatory agencies or other consultants before finalizing the response to the DSMB.

Upon receipt, the DSMB considers the sponsor delegated person`s response and attempts to resolve relevant issues to avoid compromising study integrity or the ability of the sponsor delegated person to manage the study should the study continue.

The sponsor delegated person agrees to disseminate the final decision to respective regulatory agencies, the ethics committee and investigators within an appropriate time.

10 Conflicts of Interest and Confidentiality

All data provided to the DSMB and all deliberations of the DSMB are privileged and confidential. The DSMB agrees to use this information for no other purpose than to accomplish its responsibilities.

Members are also expected to not hold any interests that would conflict with the mission of the DSMB. The DSMB is independent from the sponsor, the investigators, and anyone involved in the clinical trial care of the study subjects. The DSMB members have no financial or any other conflict of interest related to the sponsor or the investigators.

Prior to their participation in the DSMB, all members must sign the DSMB Conflict of Interest Statement and Confidentiality Agreement.

11 Amendment to this Charter

The DSMB charter can be amended as needed during the course of the trial. All amendments are documented in the table of changes and saved with sequential version numbers and revision dates. Each amendment is reviewed and agreed upon by the sponsor delegated person, the coordinating investigator and the DSMB members.

12 Signature List

The following DSMB members agree to the contents of these written operating procedures by their signature.

[signature]	12.10.2021
Signature: Univ.-Prof. Dr. med. Nikolaus Marx (Chairman)	date (dd/Mmm/yyyy)
[signature]	1.11.2021
Signature: Prof. Christine Meyer-Zürn	date (dd/Mmm/yyyy)
[signature]	28.10.2021
Signature: PD Dr. Dr. med. Efstratios Charitos	date (dd/Mmm/yyyy)

13 Abbreviations

AE	Adverse Event
DHM	Deutsches Herzzentrum München, German Heart Center Munich
DSMB	Data Safety Monitoring Board
PI	Principal Investigator
PV	Pharmacovigilance
SAE	Serious Adverse Event
SUSAR	Suspected Unexpected Serious Adverse Reaction

14 Attachments

14.1 Attachment 1: Data Review Timeline

No.	Timeline	Data Review by	Type of Data
1	Constituent meeting, before the first patient has been enrolled	Entire DSMB	To train the committee members on the trial protocol; review and approval of this DSMB charter; establish stopping guidelines, timelines and modalities for data review
2	When the first 250 patients have completed their 42 day follow-up	Entire DSMB	Data summaries pre-approved by the DSMB
3	When the first 1000 patients have completed their 42 day follow-up	Entire DSMB	Data summaries pre-approved by the DSMB
4	When the first 2000 patients have completed their 42 day follow-up	Entire DSMB	Data summaries pre-approved by the DSMB
5	At least every 12 months	Entire DSMB	Data summaries pre-approved by the DSMB
	Monthly notifications	Entire DSMB	After enrollment of the first patient monthly notifications of all SAEs and enrollment status are sent to all DSMB members for the entire duration of the trial

If enrollment or study design are modified, the DSMB schedule may also be modified to accommodate such changes in order to best monitor patient safety. Any such modification of the DSMB charter during the course of the study is documented in an amendment to the Charter.

Recommendation DSMB Meeting

Study Information			
Sponsor:<br>Klinikum der Universität München	Study Short Title:<br>EPIDAURUS	Protocol-Code:<br>EPIDAURUS-2020	EudraCT/EUDAMED No.:<br>2020-004748-27

Meeting of the Data Safety Monitoring Board (DSMB) for the EPIDAURUS trial

16 Dec 2022

The DSMB gives the following recommendation:

☐ The clinical trial EPIDAURUS can be continued without modifications

☐ The clinical trial EPIDAURUS can be continued with the following modifications:

____

☐ The clinical trial has to be temporarily stopped or terminated (please provide detailed explanation)

____

Additional Comments (if applicable):

____

____

____

______________________________ ______________

Prof. Dr. med. N. Marx (DSMB Chair) Date (dd/Mmm/yyyy)

Meeting Minutes

**A. Study Information**			
Sponsor:<br>Klinikum der Universität München	Study Short Title:<br>EPIDAURUS	Protocol-Code:<br>EPIDAURUS-2020	EudraCT/EUDAMED No.:<br>2020-004748-27

**B. Meeting Details** *(add rows as needed)*				
Type of Meeting:	☐ Face-to-face meeting ☐ Phone ☒ Web conference			
Meeting Date and Time:	12 Dec 2023 12:00-13:00 Uhr			
	Name:	Dr Nikolaus Marx	Function:	Chair DSMB
	Name:	Dr Meyer-Zürn	Function:	DSMB member
	Name	Dr Charitos	Function:	DSMB member
	Name:		Function:	
	Name:		Function:	

**C. Protocol**
**Topic and Action Items**
The DSMB discusses the study and recommends continuing the study.<br>special attention should be paid on the recruitment given but the DSMB note that recruitment has increased since the last meeting.<br>In addition, attention should be paid on stroke events and the DSMB requests more detailed information on the 3 stroke cases in the exp group

**D. Protocol written by**		
**Name (print)**	**Signature**	**Date (DD-Mmm-YYYY)**
Nikolaus Marx	N. Marx	14-12-2023

EPIDAURUS DSMB Meeting 19.9.2025

Date presentation (status 16.9.2025):

- 604 individuals are recruited (41% of 1474 planned individuals)

- of the 604 participants, 507 reached end of trial

- the following endpoints were reached:

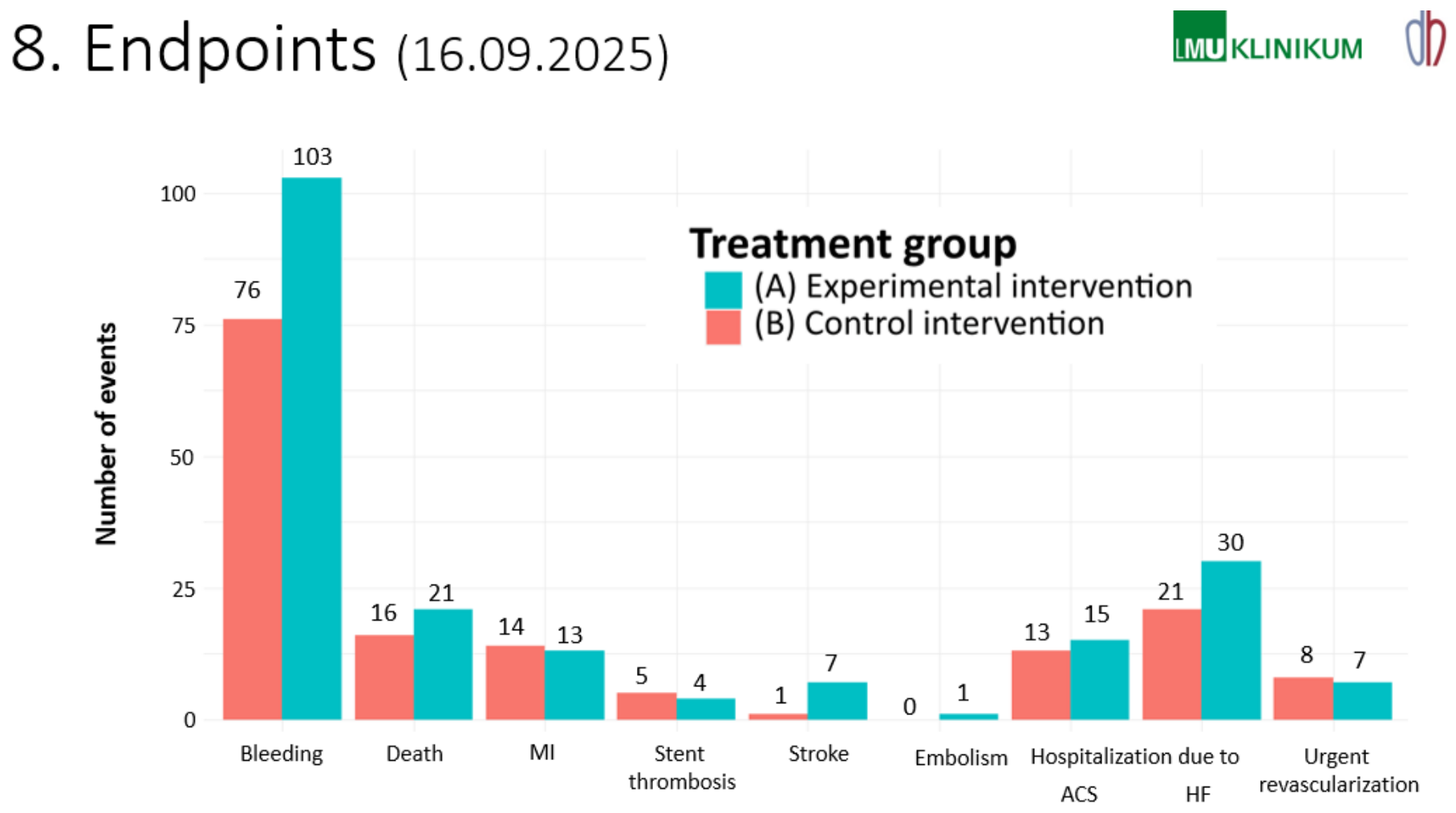

As stopping rule is defined a difference in hazard ratio of 30% or more (HR between experimental and control arm or between control and experimental arm > 1.3) with a concomitant difference in absolute deaths of > 10.

At the time of the DSMB Meeting (19.09.2025) the following event rates were reported to the committee:

Death in the Control group 16/ 301, Death in the Experimental group: 21 /303, absolute difference in deaths = 5

leading to a hazard Ratio (HR) at the time of the meeting of 1,3038

Conclusion: the strict stopping rule of a HR >1,3 AND a concomitant difference in absolute deaths of > 10 is not met given the difference in absolute deaths of 5.

Still, the DSMB recommends termination of the trial because of the following reasons:

- After reaching end of the trial in 34% of all participants there is a **high likelihood to not reach superiority of the experimental intervention vs control** given the higher rate of deaths (21 vs 16), ischemic strokes (6 vs 1), and systemic embolism (1 vs 0) and the only marginal lower numbers of MI (13 vs 14), stent thrombosis (4 vs 5), and urgent revascularisation (7 vs 8) (all components of the win ratio) in the experimental group

- The increased number of bleedings (103 vs 76) along with the increased numbers of deaths (21 vs 16) and strokes (7 vs 1) in the experimental vs the control group raise the concern of a **safety signal in the experimental group**

Recommendation:

Based on the low likelihood of superiority of the experimental intervention along with the safety signal with an increased number of deaths, strokes, and bleedings in the experimental group the DSMB recommends termination of the trial.

21.9.2025

Signature: Univ.-Prof. Dr. med. Nikolaus Marx (Chairman), date

21.9.2025

Signature: Prof. Dr. med. Christine Meyer-Zürn, date

21.09.2025

Signature: PD Dr. Dr. med. Efstratios Charitos, date
