# Supplementary material for: Dual antithrombotic therapy using potent antiplatelet inhibitors in atrial fibrillation and acute coronary syndrome: a randomized controlled trial
Source: Nat Med. 2026 Aug 29;32(11):4036–46. doi: 10.1038/s41591-026-04629-7 (PMC13768177; doi:10.1038/s41591-026-04629-7)
Supplement: Supplementary file 4 [file 41591_2026_4629_MOESM4_ESM.pdf]

# PROTOCOL

## Escalated single Platelet Inhibition for one month plus Direct oral anticoagulation in patients with Atrial fibrillation and acUte coRonary syndrome undergoing percutaneoUS coronary intervention

## EPIDAURUS

| | |
|---|---|
| **Protocol Code** | EPIDAURUS-2020 |
| **EudraCT No.** | EudraCT 2020-004748-27 |
| **Sponsor / Sponsor Delegated Person:** | LMU University Hospital Munich<br>Univ.-Prof. Dr. med. Steffen Massberg<br>Department of Cardiology<br>Marchioninistr. 15, 81377 Munich, Germany |
| **Coordinating Investigator (LKP)** | PD Dr. med. Konstantinos Rizas<br>LMU University Hospital Munich<br>Department of Cardiology<br>Marchioninistr. 15, 81377 Munich, Germany |
| **Version number / Date** | Version 4.0 (27 Oct-2021) |
| **Clinical Trial Phase:** | IV |

# Signatures:

## Declaration of the Sponsor

The present clinical trial protocol was subject to critical review. Its content is consistent with the current risk/benefit evaluation of the investigational medicinal product (IMP) as well as with moral, ethical and scientific principles of good clinical practice (GCP), the latest version of the Declaration of Helsinki, the local laws and regulations as well as applicable regulatory requirements.
With the signature below the person confirms to have read this clinical trial protocol and to agree that it contains all information required for study performance. Furthermore, the person agrees to conduct the study as set in this clinical trial protocol and to adhere to the sponsor's standard operation procedure (SOPs), if provided and as far as agreed. It has been understood that all documentation previously not published will be kept confidential. Furthermore the person agrees to take all necessary measures to ensure safety and confidentiality of the patient´s identities.

| | |
|---|---|
| Univ.-Prof. Dr. med. Steffen Massberg<br>LMU University Hospital Munich<br>Department of Cardiology<br>Author and Sponsor Delegated Person | 28.10.2021<br>Place, Date Signature |

# Declaration of the Author, the Coordinating Investigator and the Statistician

The present clinical trial protocol was subject to critical review. Its content is consistent with the current risk/benefit evaluation of the IMP as well as with moral, ethical and scientific principles of GCP, the latest version of the Declaration of Helsinki, the local laws and regulations as well as applicable regulatory requirements.
With their signature below the persons confirm to have read this clinical trial protocol and to agree that it contains all information required for study performance. Furthermore, the persons agree to conduct the study as set in this clinical trial protocol and to adhere to the sponsor's SOPs (as far as agreed and provided). It has been understood that all documentation previously not published will be kept confidential. Furthermore the persons agree to keep patient´s identities in strictest confidence and to take all required measures to ensure this confidentiality.

| | |
|---|---|
| PD Dr. med. Konstantinos Rizas<br>LMU University Hospital Munich<br>Department of Cardiology<br>Author & Coordinating Investigator (LKP) | 28.10.2021 München<br>[signature]<br>Place, Date, Signature |
| Prof. Dr. rer. nat. Ulrich Mansmann<br>Head of Institute for Medical Information Processing, Biometry, and Epidemiology, LMU Munich<br>Statistician | 28.10.2021 München<br>[signature]<br>Place, Date, Signature |

LUDWIG-MAXIMILIANS-UNIVERSITÄT
Prof. Dr. U. Mansmann
IBE - Institut für Medizinische Informationsverarbeitung, Biometrie und Epidemiologie
Marchioninistr. 15, 81377 München

# Declaration of the Coordinating Investigator

The present clinical trial protocol was subject to critical review. Its content is consistent with the current risk/benefit evaluation of the IMP as well as with moral, ethical and scientific principles of GCP, the latest version of the Declaration of Helsinki, the local laws and regulations as well as applicable regulatory requirements.
With their signature below the persons confirm to have read this clinical trial protocol and to agree that it contains all information required for study performance. Furthermore, the persons agree to conduct the study as set in this clinical trial protocol and to adhere to the sponsor's SOPs (as far as agreed and provided). It has been understood that all documentation previously not published will be kept confidential. Furthermore the persons agree to keep patient´s identities in strictest confidence and to take all required measures to ensure this confidentiality.

| | |
|---|---|
| Name and Institution of the Coordinating Investigator | ______________________<br>Place, Date, Signature |

# Investigator Agreement Page

By my signature below, I hereby attest that I have read, discussed and understood the background information concerning the investigational product. I have read and discussed this protocol and agree to carry out the trial as set out therein. I agree that the trial shall be carried out according to the ICH E6 (R2) standard and all applicable regulatory requirements. I accept my obligations relating to the principles that have their origin in the Declaration of Helsinki (2013) and specifically to Independent Ethics Committee (IEC), Informed Consent, and also my obligations to LMU University Hospital Munich, or contracted representatives, as far as safety reporting, providing data, allowing monitoring, auditing, inspection by domestic and foreign Regulatory Authorities and quality control visits are concerned.

Institution: ______________________________________________________________

Signatures

Principal Investigator: ____________________________________

Last name, first name, function / title

______________________ ____________________________________

Date [DD-Mmm-YYYY] Signature

Deputy Principal Investigator: ____________________________________

(if applicable) Last name, first name, function / title

______________________ ____________________________________

Date [DD-Mmm-YYYY] Signature

## Table of Contents

1 Protocol Synopsis ... 9
2 Schedule of Activities ... 15
3 List of Abbreviations ... 16
4 Administrative Structure ... 19
5 Introduction and Background ... 21
5.1 Background and Purpose ... 21
5.1.1 Background ... 21
5.1.2 Purpose ... 24
5.2 Dose Rationale ... 24
5.3 Overall Risk/ Benefit Assessment ... 25
6 Trial Objectives and Endpoints ... 26
6.1 Primary Endpoint(s) ... 26
6.2 Secondary Endpoints ... 26
6.3 Additional Event Evaluation ... 26
7 Clinical Trial Protocol ... 26
7.1 Trial Design ... 26
7.2 Trial Duration ... 27
8 Trial Population ... 27
8.1 Number of patients ... 28
8.2 Inclusion Criteria ... 28
8.3 Exclusion Criteria ... 28
8.4 Patient Recruitment and Screening ... 29
9 Treatment ... 29
9.1 Trial Treatment ... 29
9.2 Treatment Arms ... 32
9.2.1 Experimental intervention ... 32
9.2.2 Control intervention ... 32
9.3 Treatment Assignment and Randomization ... 32
9.4 Packaging, Labelling and Supply ... 32
9.5 Treatment of Patients ... 32
9.5.1 Patient Numbering ... 32
9.5.2 Treatment Administration ... 32
9.5.3 Compliance ... 33
9.5.4 Prior and Concomitant Medication ... 33
9.6 Trial Completion and Discontinuation ... 33
9.6.1 Trial Completion and Post-Trial Treatment ... 33

9.6.2 Lost to Follow-up ....................................................................................................33
9.6.3 Discontinuation of Patients from Treatment or Trial Participation ....................................33
9.6.4 Conditions for Terminating the Trial by the Sponsor ........................................................34
10 Trial Procedures ......................................................................................................35
10.1 Schedule of Assessments ..............................................................................................35
10.1.1 Screening / Baseline Visit ...........................................................................................35
10.1.2 Visit at D ......................................................................................................................36
10.1.3 Control Visit (Day 42 ± 7) ..............................................................................................37
10.1.4 Final Visit (Day 180 ± 10) ..............................................................................................37
11 Safety Monitoring ........................................................................................................37
11.1 Definitions .....................................................................................................................37
11.1.1 Adverse Event (AE) ......................................................................................................37
11.1.2 Adverse Reaction (AR) ..................................................................................................37
11.1.3 Serious Adverse Event (SAE) ..........................................................................................37
11.1.4 Suspected Unexpected Serious Adverse Reaction (SUSAR) ..........................................38
11.2 Assessment Criteria ........................................................................................................38
11.2.1 Adverse Event Assessment by the Investigator ..............................................................38
11.2.2 Assessment by the Sponsor ..........................................................................................39
11.3 Collection and Reporting of (Serious) Adverse Events .................................................39
11.3.1 Adverse Events .............................................................................................................39
11.3.2 Serious Adverse Events .................................................................................................40
11.4 Expedited reporting .........................................................................................................41
11.5 Reporting of Trial Treatment Errors including Misuse / Abuse .......................................41
11.6 Pregnancy Reporting .......................................................................................................41
11.7 Annual Safety Report .......................................................................................................42
12 Statistics .....................................................................................................................42
12.1 Sample Size Calculation ..................................................................................................42
12.2 Definition of Population Included in the Analysis ..........................................................42
12.3 Statistical Methods ..........................................................................................................42
12.4 Pre-specified subgroup analysis ......................................................................................43
12.5 Handling of Protocol Deviations and Missing Data .........................................................44
13 Substudy ......................................................................................................................44
14 Quality Assurance and Control ..................................................................................44
14.1 Routine Monitoring ..........................................................................................................44
14.2 Compliance with Protocol and Protocol Revisions ..........................................................44
14.3 Measures of Quality Assurance and Quality Control .......................................................45
14.3.1 Quality Control .............................................................................................................45
14.3.2 Quality Assurance ........................................................................................................45
15 Committees ..................................................................................................................46
15.1 Steering Committee ........................................................................................................46

15.2 Data Safety Monitoring Board ....................46
15.3 Event Adjudication Committee ....................46
16 Data Handling and Record Keeping ....................46
16.1 Case Report Forms ....................46
16.2 Source Data ....................47
16.3 Data Management ....................47
16.3.1 Data Handling and Quality Control ....................47
16.3.2 Data Coding ....................48
16.3.3 Database Lock ....................48
16.4 Data Protection and Confidentiality ....................48
16.5 Archiving and Data Retention ....................48
16.6 Access to Source Data/Documents / Audits and Inspections ....................49
17 Ethical, Regulatory and Administrative Aspects ....................49
17.1 Good Clinical Practice ....................49
17.2 Ethics Commission and Regulatory Authorities ....................49
17.3 Informed Consent ....................49
18 Financing ....................50
19 Insurance ....................50
20 Clinical Trial Report and Publication Policy ....................50
21 References ....................52
22 Appendices ....................54

# 1 Protocol Synopsis

| | |
|---|---|
| Title of Study: | **E**scalated single **P**latelet **I**nhibition for one month plus **D**irect oral anticoagulation in patients with **A**trial fibrillation and ac**U**te co**R**onary syndrome undergoing percutaneo**US** coronary intervention |
| Short Title: | EPIDAURUS |
| Protocol Code: | EPIDAURUS-2020 |
| Sponsor / Sponsor Delegated Person: | LMU University Hospital Munich<br>Univ.-Prof. Dr. med. Steffen Massberg<br>Department of Cardiology<br>Marchioninistr. 15, 81377 Munich, Germany |
| Coordinating Investigator (LKP): | PD Dr. med. Konstantinos Rizas<br>LMU University Hospital Munich<br>Department of Cardiology<br>Marchioninistr. 15, 81377 Munich, Germany |
| Trial Type: | Prospective, randomized, open-label, multinational European, multicentre, phase IV clinical trial |
| Design: | Experimental intervention:<br>Escalated regimen consisting of<br>Weeks 1 – 4 : Potent $P2Y_{12}$-inhibitor + non-vitamin K antagonist oral anticoagulant<br>Week 5 – month 6: Clopidogrel + non-vitamin K antagonist oral anticoagulant<br>Control intervention:<br>Standard regimen consisting of<br>In-hospital: Aspirin + Clopidogrel + non-vitamin K antagonist oral anticoagulant (up to 1 week)<br>Hospital-discharge or day 8 – month 6: Clopidogrel + non-vitamin K antagonist oral anticoagulant. In patients with high ischaemic risk the treating physician is allowed to prescribe Aspirin for a period of up to 4 weeks, according to current guidelines. |
| Hypothesis / Objectives: | To test whether an escalated antiplatelet therapy with a potent $P2Y_{12}$-inhibitor for one month can reduce ischaemic events without a significant increase in bleeding complications in patients with atrial fibrillation and ST elevation myocardial infarction (STEMI) or non-ST-segment elevation myocardial |

| | |
|---|---|
| | infarction (NSTEMI) undergoing successful completion of percutaneous coronary intervention. |
| Inclusion Criteria: | • Signed written informed consent<br>• Age ≥ 18 years<br>• Atrial fibrillation requiring oral anticoagulation<br>• STEMI or NSTEMI (biomarker positive acute coronary syndrome) and successful completion of PCI (randomization will take place within 24h after successful PCI) |
| Exclusion Criteria: | • Chronic renal insufficiency with glomerular filtration rate < 15 ml/min/1.73m$^2$<br>• History of ischaemic stroke or transient ischaemic attack (both contraindications for Prasugrel) and history of intracranial bleeding (contraindication for Ticagrelor)<br>• Contraindication for Clopidogrel or Aspirin<br>• Contraindication for Prasugrel and Ticagrelor<br>• Severe chronic liver disease (Child-Pugh C)<br>• Indication for oral anticoagulation with Vitamin K antagonists<br>• Moderate to severe mitral stenosis or mechanical heart valve<br>• Any bleeding BARC type ≥ 2 within the last 4 weeks before index procedure<br>• Pregnancy or lactation<br>• Inability to cooperate with the protocol requirements<br>• Life expectancy < 6 months<br>• Participation in another investigational drug study<br>• Previous enrolment in this study<br>• For women of childbearing potential no negative pregnancy test and no agree to use a reliable method of birth control during the study<br>• Previous treatment with GP IIb/IIIa inhibitors within the last 12 hours<br>• A known genetic disorder involved in the metabolism of the study medication |

| | |
|---|---|
| Primary Endpoints: | Primary efficacy endpoint:<br>Major ischaemic events defined as the composite of all-cause mortality, myocardial infarction, definite or probable stent thrombosis, ischaemic stroke or systemic thromboembolism (superiority test) at 6 weeks after randomization<br>Primary safety endpoint:<br>Bleeding type 2 or higher according to the Bleeding Academic Research Consortium (BARC) criteria (non-inferiority test) at 6 weeks after randomization |
| Secondary Endpoints: | Secondary endpoints:<br>• All individual components of the primary endpoint (all-cause mortality, myocardial infarction, definite or probable stent thrombosis, ischaemic stroke, systemic thromboembolism) at 6 weeks after randomization<br>• Cardiovascular mortality at 6 weeks after randomization<br>• Bleeding (BARC type ≥ 2) at 6 weeks after randomization<br>• Urgent revascularization at 6 weeks after randomization<br>• All-cause mortality at 6 months after randomization<br>• Unplanned hospitalization due to acute heart failure or acute coronary syndrome at 6 months after randomization<br>• Ischaemic stroke at 6 months after randomization |
| Randomization: | A randomized parallel group design to control for confounding is used. Patients will be randomized within 24h after successful completion of PCI (defined as TIMI flow grade 2 or more; transradial approach preferred). The randomization will be 1:1 for each treatment arm with stratification by centre. The block size is chosen as 8. |
| Study Visits: | Screening/Baseline Visit:<br>- Informed Consent<br>- Assessment of in- and exclusion criteria<br>- Demographic data, general and cardiovascular medical history, current diseases<br>- Clinical parameters<br>- Laboratory parameters<br>- Concomitant medication |

| | |
|---|---|
| | - Randomization<br>Discharge Visit:<br>- Laboratory parameters: CK max, troponin max<br>- Assessment of adverse events and occurrence of endpoints<br>- Concomitant medication<br>Control Visit (Day 42 ± 7):<br>- Assessment of adverse events and occurrence of endpoints<br>- Assessment of symptoms: angina pectoris (CCS classification), dyspnoea (NYHA classification), syncope<br>- Concomitant medication<br>- Laboratory: Laboratory parameters that were assessed after discharge of index hospital stay (if applicable)<br>Final Visit (Day 180 ± 10):<br>- Assessment of adverse events and occurrence of endpoints<br>- Concomitant medication<br>- Smoking status |
| Sample Size / Number of patients / Planned No. of Sites: | To be assessed for eligibility (n = 2500)<br>To be allocated to trial (n = 2334)<br>To be analysed (n = 2304)<br>Assumptions for sample-size calculation (hierarchical testing)<br>• Efficacy endpoint (ischaemic events) at 6 weeks:<br>Superiority testing, 2-sided alpha 5%, power 80%, 6-weeks event-rate in the control group 7%, 6-weeks event-rate in the intervention group 4.3%, corresponding to an odds-ratio of 0.6.<br>Number per group: 1147<br>• Safety endpoint (bleeding BARC ≥ 2) at 6 weeks:<br>Non-inferiority testing, when the sample size in each group is 1152, a two-group large-sample normal approximation test of proportions with a one-sided 2.5% significance level will have 80% power to reject the null hypothesis that the test and the standard are not equivalent (6-weeks event-rate in the control group 13.1%, odds-ratio between treatment and control-group 0.95, difference in proportions, $\pi_1 - \pi_0$, is -0,033 or farther |

| | |
|---|---|
| | from zero in the same direction) in favour of the alternative hypothesis that the proportions in the two groups are equivalent.<br><br>Number per group: 1152<br><br>The sample size of the trial is given by the maximum of both sample sizes: 1152 per group.<br><br>The computation assumed an allocation ratio $n_{control}/n_{interventional}$ = 1.0, an accrual time of 24 months, a follow-up time of 6 months and an annual 10% follow-up drop-out rate (6-week rate 1.25%, 2304 * 1.0125 = 2334). |
| Data Analysis: | Efficacy endpoint:<br><br>Superiority testing, 2-sided alpha 5%, power 80%, 6 weeks event-probability in the control group based on a post-hoc analysis of the ISAR-REACT 5 trial 7%, 6 weeks event-probability in the intervention group 4.3%, corresponding to an odds-ratio of 0.6.<br><br>Null hypothesis $H_0$: OR =1 (difference). For a two-sided alpha of 0.05, 6-week event probability in the control group of 0.070 and in the intervention group of 0.043 (OR = 0.6) the power is 80.0% if the chi-square test is performed on 1147 patients per group. The computation assumes an allocation ratio (n2/n1) = 1.0.<br><br>Safety endpoint:<br><br>Non-inferiority testing, when the sample size in each group is 1152, a two-group large-sample normal approximation test of proportions with a one-sided 2.5% significance level will have 80% power to reject the null hypothesis that the test and the standard are not equivalent (6-week event-rate in the control group 13.1%, odds-ratio between treatment and control-group 0.95, difference in proportions, $\pi_1 - \pi_0$, is -0,033 or farther from zero in the same direction) in favour of the alternative hypothesis that the proportions in the two groups are equivalent.<br><br>The sample size of the trial is given by the maximum of both sample sizes: 1152 per group.<br><br>Sample size calculations were performed using the nQuery platform for clinical trial design.<br><br>Compliance/Rate of loss to follow-up:<br><br>We assumed an annual 10% drop-out rate (6-week rate 1.25%) and increase in the total sample size to 2334 (=2304 * 1.0125). The computation assumed an allocation ratio |

| | |
|---|---|
| | $n_{control}/n_{experimental}$ = 1.0 and a constant accrual time of 24 months. |
| Duration of the Trial: | Time for preparation of the trial (months): 6<br>First patient in to last patient out (months): 30<br>Duration of the entire trial (months): 36<br>Recruitment period (months): 24<br>Trial duration per patient (months): 6<br>End of Trial: Last patient last visit |
| Follow-up: | Control visit: Day 42 (± 7 days)<br>Final visit: Day 180 (± 10 days) |

## 2 Schedule of Activities

| | Screening/ Baseline visit Day 0 | Discharge visit | Control visit Day 42 ± 7 | Final visit Day 180 ± 10 |
|---|---|---|---|---|
| Informed consent | X | | | |
| Inclusion- and exclusion criteria | X | | | |
| Demographic data | X | | | |
| Patient history | X | | | |
| Clinical parameters | X | | | |
| Laboratory[1] | X | | X[2] | |
| Concomitant medication | X | X | X | X |
| Randomization | X | | | |
| AEs und SAEs | X | X | X | X |
| Primary efficacy endpoint (all-cause mortality, MI, ST, ischaemic stroke, systemic thromboembolism) | | X | X | |
| Primary safety endpoint (BARC bleeding ≥ 2) | | X | X | |
| Cardiovascular mortality | | X | X | |
| MI | | X | X | |
| Ischaemic stroke | | X | X | X |
| Stent thrombosis | | X | X | |
| Systemic thromboembolism | | X | X | |
| Urgent revascularization | | X | X | |
| BARC bleeding ≥ 1 | | X | X | |
| All-cause mortality | | X | X | X |
| Unplanned hospitalization due to acute heart failure or acute coronary syndrome | | | X | X |

[1] Beyond clinical routine, platelet function testing will be performed. [2] Not obligatory

# 3 List of Abbreviations

| Term | Definition |
|---|---|
| ACS | Acute Coronary Syndrome |
| AE | Adverse Event |
| ACEI | Angiotensin-Converting-Enzyme Inhibitor |
| AF | Atrial Fibrillation |
| ATC | Anatomical Therapeutical Chemical |
| AR | Adverse Reaction |
| ARB | Angiotensin II Receptor Blocker |
| ARC | Academic Research Consortium |
| ARNI | Angiotensin-Receptor-Neprilysin-Inhibitor |
| BARC | Bleeding Academic Research Consortium |
| BNP | Brain Natriuretic Peptide |
| BUN | Blood Urea Nitrogen |
| CA | Competent Authority |
| CABG | Coronary Artery Bypass Grafting |
| CAPA | Corrective and Preventive Action |
| CAD | Coronary Artery Disease |
| CCB | Calcium Channel Blocker |
| CCS | Canadian Cardiovascular Society |
| cCT | Cerebral Computed Tomography |
| CI | Confidence Interval |
| CK | Creatine Kinase |
| CRF | Case Report Form |
| CRF | Chronic Renal Failure |
| CRO | Clinical Research Organization |
| CRP | C-reactive Protein |
| CRT | Cardiac Resynchronization Therapy |
| cTn | Cardiac Troponin |
| GOLD | Global Initiative for Chronic Obstructive Lung Disease |
| COPD | Chronic Obstructive Pulmonary Disease |
| CVD | Cerebrovascular Disease |
| DAPT | Dual Antiplatelet Therapy |
| DAT | Dual Antithrombotic Therapy |
| DC | Deceleration Capacity |
| DMP | Data Management Plan |
| DSMB | Data Safety Monitoring Board |
| DVP | Data Validation Plan |
| DZHK | Deutsches Zentrum für Herz-Kreislauf-Forschung e.V. |
| EDTA | (Ethane-1,2-diyldinitrilo)Tetraacetic Acid |
| ELISA | Enzyme-linked Immunosorbent Assay |
| GP IIb/IIIa | Glycoprotein IIb/IIIa |

| | |
|---|---|
| HbA1c | Haemoglobin A1c |
| HDL | High-density Lipoprotein |
| HR | Hazard Ratio |
| EAC | Event Adjudication Committee |
| EC | Ethics Committee |
| ECG | Electrocardiogram |
| EDC | Electronic Data Capture |
| eCRF | Electronic Case Report Form |
| ESC | European Society of Cardiology |
| FPFV | First patient First Visit |
| GCP | Good Clinical Practice |
| GOT | Glutamate-Oxaloacetate-Transaminase |
| GPT | Glutamate-Pyruvate-Transaminase |
| ICD | Implantable Cardioverter Defibrillator |
| ICH | International Conference on Harmonisation |
| ID | Identifier |
| IEC | Independent Ethics Committee |
| INR | International Normalized Ratio |
| IMP | Investigational Medicinal Product |
| ISTH | International Society on Thrombosis and Haemostasis |
| ITT | Intention to treat |
| LCA | Left Coronary Artery |
| LDH | Lactate Dehydrogenase |
| LDL | Low-density Lipoprotein |
| LVEF | Left Ventricular Ejection Fraction |
| LKP | Leiter der Klinischen Prüfung |
| MACE | Major Advers Cardiac Event |
| MI | Myocardial Infarction |
| MRI | Magnetic Resonance Imaging |
| NOAC | New Oral Anticoagulant |
| NSAID | Non-Steroidal Anti-Inflammatory Drug |
| NSTEMI | Non-ST-Elevation Myocardial Infarction |
| NT-proBNP | N-terminal pro Brain Natriuretic Peptide |
| NYHA | New York Heart Association |
| OAC | Oral Anticoagulation |
| OR | Odds Ratio |
| PAOD | Peripheral Arterial Occlusive Disease |
| PBC | Primary Biliary Cirrhosis |
| PCI | Percutaneous Coronary Intervention |
| PFT | Platelet Function Test |
| PRD | Periodic Repolarization Dynamics |
| PSC | Primary Sclerosing Cholangitis |
| SAE | Serious Adverse Event |

| | |
|---|---|
| SAP | Statistical Analysis Plan |
| SD | Standard Deviation |
| SLGT2 | Sodium dependent Glucose co-transporter 2 |
| SmPC | Summary of Product Characteristics |
| ST | Stent Thrombosis |
| STEMI | ST-Elevation Myocardial Infarction |
| SUSAR | Suspected Unexpected Serious Adverse Reaction |
| SOP | Standard Operation Procedure |
| TAT | Triple Antithrombotic Therapy |
| TIMI | Thrombolysis In Myocardial Infarction |
| TLR | Target Lesion Revascularization |
| TMF | Trial Master File |
| TVR | Target Vessel Revascularization |
| URL | Upper Reference Limit |
| VKA | Vitamin K antagonist |

# 4 Administrative Structure

| | |
|---|---|
| Sponsor /<br>Sponsor Delegated Person | LMU University Hospital Munich<br>Univ.-Prof. Dr. med Steffen Massberg<br>Department of Cardiology<br>Marchioninistr. 15, 81377 Munich, Germany |
| Coordinating Investigator / LKP | PD Dr. med. Konstantinos Rizas<br>LMU University Hospital Munich<br>Department of Cardiology<br>Marchioninistr. 15, 81377 Munich, Germany |
| Steering Committee | Univ.-Prof. Dr. med. Steffen Massberg (Chair, LMU University Hospital Munich)<br>PD Dr. med. Konstantinos Rizas (LMU University Hospital Munich)<br>Prof. Dr. med. Ingo Eitel (University Heart Center Lübeck)<br>PD Dr. med. Thomas Stiermaier (University Heart Center Lübeck)<br>Prof. Dr. Adnan Kastrati (Deutsches Herzzentrum München)<br>Prof. Dr. med. Dirk Sibbing (Privatklinik Lauterbacher Mühle am Ostersee)<br>Prof. Dr. med. Paulus Kirchhof (Universitäres Herz- und Gefäßzentrum UKE Hamburg)<br>Prof. Dr. med. Christoph Stellbrink (Klinikum Bielefeld)<br>Univ.-Prof. Dr. med. Holger Thiele (Herzzentrum Leipzig)<br>Univ.-Prof. Dr. med. Ulf Landmesser (Campus Benjamin Franklin, Charité – Universitätsmedizin Berlin) |
| Sources of financial support | Daiichi-Sankyo Deutschland GmbH<br>Deutsches Zentrum für Herz-Kreislauf-Forschung e.V. (DZHK) |
| Coordinating Research Center | ISAResearch Center<br>Deutsches Herzzentrum München des Freistaates Bayern<br>Klinik an der Technischen Universität München<br>Lazarettstr. 36, 80636 Munich, Germany |

| | |
|---|---|
| Monitoring | ISAResearch Center<br>Deutsches Herzzentrum München des Freistaates Bayern<br>Klinik an der Technischen Universität München<br>Lazarettstr. 36, 80636 Munich, Germany |
| Biometry / Statistics | Prof. Dr. rer. nat. Ulrich Mansmann<br>Institute for Medical Information Processing, Biometry, and Epidemiology, LMU Munich<br>Marchioninistr. 15, 81377 Munich, Germany |
| Data Management | ISAResearch Center<br>Deutsches Herzzentrum München des Freistaates Bayern<br>Klinik an der Technischen Universität München<br>Lazarettstr. 36, 80636 Munich, Germany |
| Pharmacovigilance/ Safety Management | ISAResearch Center<br>Deutsches Herzzentrum München des Freistaates Bayern<br>Klinik an der Technischen Universität München<br>Lazarettstr. 36, 80636 Munich, Germany |

The participating sites with contact details are to be found in a list maintained by Project Management.

# 5 Introduction and Background

## 5.1 Background and Purpose

### 5.1.1 Background

The selection of the optimal antithrombotic therapy in patients with nonvalvular atrial fibrillation (AF) and acute coronary syndrome (ACS) undergoing percutaneous coronary intervention (PCI) is challenging. Dual antiplatelet therapy (DAPT) with Aspirin and a $P2Y_{12}$-inhibitor has been proven to reduce recurrent cardiovascular events in patients undergoing PCI, but is less effective in reducing the incidence of ischaemic stroke related to AF (1). On the other hand, oral anticoagulants (OAC) are the standard therapy to prevent stroke and systemic embolism in patients suffering from AF, but is not indicated as secondary prevention after PCI (2). Until recently, triple antithrombotic therapy (TAT) consisting of Aspirin plus Clopidogrel plus OAC was considered the treatment of choice. While efficiently preventing ischaemic events, TAT is associated with an increase in bleeding complications. Therefore, in the past years several randomized controlled trials (PIONEER AF-PCI, RE-DUAL PCI, ENTRUST-AF PCI, and AUGUSTUS) challenged TAT by comparing a TAT-regimen based on Vitamin K antagonists (VKA) to a dual antithrombotic therapy (DAT)-regimen based on new oral anticoagulants (NOACs) and $P2Y_{12}$-inhibitors, mainly Clopidogrel in patients with AF undergoing PCI (3-6).

In PIONEER AF-PCI a total of 2,124 patients with AF undergoing PCI were randomized in a 1:1:1 ratio to a standard TAT regimen with VKA and DAPT (control group) vs. a DAT group with low-dose Rivaroxaban (15mg) and a $P2Y_{12}$-inhibitor for 12 months (first intervention group), and finally a TAT regimen with very-low dose Rivaroxaban (2.5mg twice daily) plus DAPT, followed by Rivaroxaban 15mg at the time of $P2Y_{12}$-inhibitor discontinuation (second intervention group) (6). In this trial, 29% of the patients presented with biomarker positive myocardial infarction (MI) and 22% with unstable angina. Ninety-four percent of the patients were treated with Clopidogrel and only 6% of the patients were on treatment with a potent $P2Y_{12}$-inhibitor (Prasugrel or Ticagrelor). Compared to standard TAT with VKA, Rivaroxaban-based regimens were associated with a significant reduction in adverse bleeding events without a significant increase in major adverse cardiovascular events. However, the power for the exploratory analysis of ischaemic events was estimated to be 11.4%.

RE-DUAL PCI randomized 2,725 patients with AF undergoing PCI (50% presented with ACS) to two regimens with Dabigatran (110mg and 150mg) and a $P2Y_{12}$-inhibitor (Clopidogrel in 88%, Ticagrelor in 12%) vs. a standard TAT regimen with VKA, Clopidogrel and Aspirin for 1-3 months (5). During a mean follow-up period of 14 months there was a significant reduction in major and clinically relevant non-major bleeding events in the groups treated with Dabigatran compared to the control group, without a significant increase in thromboembolic events. There was a numeric increase of ischaemic events in patients receiving DAT compared to TAT. However, regarding ischaemic events the trial was only powered for a non-inferiority analysis with a non-inferiority margin of 1.38 (5).

ENTRUST-AF PCI was a phase 3b trial, which randomized 1,506 patients with AF undergoing PCI to a DAT-regimen based on Edoxaban and a TAT-regimen with VKA, $P2Y_{12}$-inhibitor and Aspirin (3). Among the included patients, 52% presented with an ACS and only 7% were treated with a potent $P2Y_{12}$-inhibitor. The NOAC-based DAT regimen was associated with a non-significant reduction in bleeding complications. In an exploratory analysis, which however was not sufficiently powered, there was no significant increase in ischaemic complications between the two treatment groups, even though myocardial infarction and stent thromboses (ST) were seen more frequently in DAT compared to TAT.

In the recently published AUGUSTUS trial, 4,614 patients with coronary artery disease (CAD) and AF were randomized in a two-by-two factorial design to Apixaban vs. VKA and to Aspirin (TAT regimen) vs. placebo (DAT regimen) for 6 months (4) Only 37% of the patients presented with an ACS treated with PCI. About 24% of the patients presented with an ACS, which was treated medically. Only 331 (7%) patients were treated with a potent $P2Y_{12}$-inhibitor. At 6 months, the primary outcome of major or clinically relevant non-major bleeding events was significantly lower in patients treated with Apixaban compared to patients treated with VKA and was 89% higher in patients on a TAT regimen compared to patients on a DAT regimen. In a post-hoc analysis, which only included patients treated with PCI, there was a 42%-decrease in the rate of stent thromboses in patients treated with a TAT regimen compared to DAT (HR 0.58, 95% CI 0.28–1.22) (7) which however, did not reach the level of statistical significance. Eighty percent of all stent thromboses occurred within the first month after the index PCI (8).

The study design, baseline characteristics and endpoints of the PIONEER AF-PCI, RE-DUAL PCI, ENTRUST-AF PCI, and AUGUSTUS trials are summarized in Table 1. Based on the results of the above-mentioned randomized trials, which consistently showed a significant decrease in bleeding complications without detecting a significant increase in ischaemic events, current guidelines were revised and now recommend an in-hospital TAT followed by DAT based on NOAC in patients with an indication for oral anticoagulation undergoing PCI (2,9) However, all of the above trials were powered to show a superiority of a NOAC-based DAT to the VKA-based TAT with regard to bleeding events. In contrast, all analyses regarding ischaemic events were exploratory and not sufficiently powered. Moreover, the patients at high-risk of ischaemic complications with biomarker positive ACS undergoing PCI were underrepresented in all studies. In a recent meta-analysis of all four trials Gargiulo et al. could show that the decrease in bleeding complications with DAT compared to TAT comes at the expense of a significantly higher risk of stent thromboses (HR 1.59, 95% CI 1.01 - 2.50, p = 0.04) and a borderline increase in the risk of MI (HR 1.22, 95% CI 0.99 - 1.52, p = 0.07) (10). As presented in the post-hoc analysis of the AUGUSTUS trial these ischaemic events primarily occured within the first month after the index PCI (8).

Activation of blood platelets plays a key role during the early phase of an ACS. Approximately 30-40% of patients show low response to Clopidogrel and are not adequately protected against ischaemic events, in particular when presenting with ACS (11). Consequently, clinical outcomes of patients with ACS undergoing PCI without AF have been significantly improved with the use of potent $P2Y_{12}$ - receptor inhibitors Prasugrel or Ticagrelor. It is therefore reasonable to assume that a more potent platelet inhibition within the first month after PCI might reduce the rate of ischaemic complications observed in AF patients with ACS undergoing PCI, when receiving DAT. On the other hand, a subsequent de-escalation to a less potent platelet inhibition one month after PCI might prevent an increase in bleeding complications. This hypothesis is supported by a landmark analysis of the TRITON-TIMI-38 trial (12), which showed that the beneficial effect of Prasugrel compared to Clopidogrel with regard to ischaemic events was mainly exerted within the first days after the index-MI, while the bleeding complications progressively increased over time with non-significant differences in bleeding rates within the first month of treatment with Prasugrel vs. Clopidogrel.

In EPIDAURUS we will therefore test the hypothesis that DAT using NOAC plus an escalated antiplatelet therapy with a potent $P2Y_{12}$-inhibitor for one month followed by Clopidogrel reduces ischaemic events without a relevant increase in bleeding complications in AF patients with ACS undergoing PCI compared to standard in-hospital TAT followed by DAT with NOAC plus Clopidogrel.

**Table 1:** Summary of the study design, baseline characteristics and endpoints of PIONEER AF-PCI (6), RE-DUAL PCI (5), ENTRUST-AF PCI (3) and AUGUSTUS (4) trials.

| | PIONEER AF-PCI | RE-DUAL PCI | ENTRUST-AF PCI | AUGUSTUS |
|---|---|---|---|---|
| **NOAC** | Rivaroxaban | Dabigatran | Edoxaban | Apixaban |
| **Patients (N)** | 2.124 | 2.725 | 1.506 | 4.614 |
| **Groups** | 3 (open-label) | 3 (open-label) | 2 (open-label) | 2x2 (open/blinded) |
| **Age (Mean ± SD)** | 70 ± 9 | 70 ± 9 | 70 ± 7 | 71 ± 7 |
| **NSTEMI /STEMI (%)** | 29% | 50% | 52% | 51 % (37% PCI) |
| **Clopidogrel (%)** | 94% | 88% | 93% | 93% |
| **Powered for** | Superiority for ISTH bleeding (major/minor) | Non-inferiority for ISTH bleeding (major/minor) | Non-inferiority for ISTH bleeding (major/minor) | Superiority for ISTH bleeding (major/minor) |
| **Follow-up (months)** | 12 | 14 (mean) | 12 | 6 |
| **DAT vs. TAT (HR and 95% CI)** | | | | |
| **ISTH major bleeding** | 0.56 (0.36 – 0.89) | 0.57 (0.43 – 0.76) | 0.94 (0.64 – 1.40) | 0.60 (0.44 – 0.81) |
| **MACE** | 1.03 (0.84 – 1.25) | 1.03 (0.84 – 1.25) | 1.07 (0.73 – 1.58) | 1.13 (0.91 – 1.40) |
| **Stent thrombosis** | 1.25 (0.34 – 4.64) | 1.55 (0.69 – 3.46) | 1.34 (0.47 – 3.84) | 1.92 (0.92 – 3.95) |

CI: confidence interval; NOAC: new oral anticoagulants; HR: hazard ratio; ISTH: International Society on Thrombosis and Haemostasis; MACE: major adverse cardiac events; SD: standard deviation; NSTEMI: non-ST-segment elevation myocardial infarction; STEMI: ST-segment elevation myocardial infarction; DAT: dual antithrombotic therapy; TAT: triple antithrombotic therapy.

### 5.1.2 Purpose

The selection of the optimal antithrombotic therapy in patients with nonvalvular atrial fibrillation (AF) and acute coronary syndrome (ACS) undergoing percutaneous coronary intervention (PCI) is challenging. Until recently, triple antithrombotic therapy (TAT) consisting in Aspirin plus Clopidogrel plus OAC was considered the treatment of choice. While efficiently preventing ischaemic events, TAT is associated with an increase in bleeding complications. Therefore, in the past years several randomized controlled trials challenged TAT by comparing a triple antithrombotic therapy (TAT) regimen based on Vitamin K antagonists (VKA) to a dual antithrombotic regimen (DAT) based on non-vitamin K antagonist oral anticoagulants (NOACs) and $P2Y_{12}$-inhibitors, mainly Clopidogrel in patients with AF undergoing PCI.

However, approximately 30-40% of patients show low response to Clopidogrel and are not adequately protected against ischaemic events, in particular when presenting with ACS. This is supported by a recent meta-analysis (10) reporting that TAT compared to DAT is associated with lower rates of stent thrombosis within 30 days after PCI. It is therefore reasonable to assume that a more potent platelet inhibition within the first month after PCI might reduce the rate of ischaemic complications observed in AF patients undergoing PCI, when receiving DAT. Moreover, a subsequent de-escalation to a less potent platelet inhibition one month after PCI might prevent an increase in bleeding complications.

In EPIDAURUS we will therefore test the hypothesis that DAT using NOAC plus an escalated antiplatelet therapy with a potent $P2Y_{12}$-inhibitor for one month followed by Clopidogrel reduces ischaemic events without a relevant increase in bleeding complications in patients with AF and ACS undergoing PCI compared to standard DAT with NOAC plus Clopidogrel.

## 5.2 Dose Rationale

Treatment in the control group (standard arm) is based on recommendations by current guidelines, which recommend early cessation (<1 week) of TAT and continuation of DAT with NOAC and Clopidogrel in AF patients undergoing PCI (IIa, B recommendation in the current ESC guidelines) (9).

In the experimental (escalated) arm, an escalated antiplatelet therapy with a potent $P2Y_{12}$-inhibitor for one month followed by a de-escalation to Clopidogrel will be applied.

Following enrolment, patients will undergo a 1:1 randomization to a standard therapy (control arm) with an in-hospital TAT (Aspirin, Clopidogrel and NOAC - preferentially Edoxaban or Apixaban) (for a maximum of one week) and a subsequent DAT (NOAC - preferentially Edoxaban or Apixaban plus Clopidogrel) for 6 months or an intensified regimen (interventional arm) of NOAC (preferentially Edoxaban or Apixaban) plus a potent $P2Y_{12}$-inhibitor (Prasugrel (preferred) or Ticagrelor) for the first month (4 weeks) followed by NOAC (preferentially Edoxaban or Apixaban) and Clopidogrel for months 2-6. The default duration of Aspirin therapy in the control arm is in-hospital (up to 1 week). However, the treating physicians are allowed to extend the therapy with Aspirin in the control arm for a period of up to 4 weeks in patients with high ischaemic risk, according to current guidelines (2,9).

After randomization, Aspirin will be continued at a maintenance dose of 75-100mg (for Germany and Austria only 100 mg are applicable) for the in-hospital stay for a maximum of 7-days in the control group (11) and will be immediately discontinued in the intervention group. Switching between Clopidogrel and potent $P2Y_{12}$-inhibition or between $P2Y_{12}$-inhibition and Clopidogrel will be

performed according to current recommendations (13). Patients with a new-diagnosed AF will be allowed to participate in the study if they are randomized within 24 hours after PCI. After completion of the study a DAT with Aspirin or Clopidogrel and NOAC is suggested. No additional loading dose will be required for de-escalation to Clopidogrel (13).

## 5.3 Overall Risk/ Benefit Assessment

In the EPIDAURUS trial we assume that an escalated antiplatelet therapy with a potent $P2Y_{12}$-inhibitor for one month followed by Clopidogrel will reduce ischaemic events, defined as the composite of all-cause mortality, myocardial infarction, definite or probable stent thrombosis, ischaemic stroke and systemic thromboembolism at 6 weeks after randomization.

This hypothesis is supported by the results of several studies. First, activation of blood platelets is known to play a key role during the early phase of an ACS. Approximately 30-40% of patients show low response to Clopidogrel and are not adequately protected against ischaemic events, in particular when presenting with ACS (11). Second, clinical outcomes of patients with ACS undergoing PCI without AF have been significantly improved with the use of potent $P2Y_{12}$ receptor inhibitors Prasugrel or Ticagrelor. Third, in a recent meta-analysis of PIONEER AF-PCI, RE-DUAL PCI, ENTRUST-AF PCI, and AUGUSTUS trials Gargiulo et al. could show that a DAT consisting of NOAC and Clopidogrel compared to an intensified TAT is associated with a higher risk for stent thromboses and a borderline increase in the risk for MI (10). Finally, as presented in the post-hoc analysis of the AUGUSTUS trial these ischaemic events primarily occur within the first month after a PCI (8).

The risk of an intensified antithrombotic therapy depends on the underlying condition. For patients with a previous haemorrhagic stroke, a clinically overt bleeding BARC ≥ 2 within the last month and patients with chronic renal insufficiency, an escalated antiplatelet therapy with a potent $P2Y_{12}$-inhibitor might be associated with an increased rate of bleeding complications. For this reason, patients with previous haemorrhagic stroke, a clinically overt bleeding within the last 4 weeks before index procedure and known chronic renal insufficiency with glomerular filtration rate < 15 ml/min/1.73m$^2$ will be excluded from the study. In all other patients the bleeding risk under treatment with a potent $P2Y_{12}$-inhibitor is comparable to the bleeding risk under treatment with Clopidogrel. This is supported by a landmark analysis of the TRITON-TIMI-38 trial (12), which showed that the bleeding rates within the first month of treatment with Prasugrel vs. Clopidogrel did not differ significantly.

In summary, it is reasonable to assume that in patients with AF and MI undergoing PCI, benefit of reduced ischaemic events due to an intensified antithrombotic therapy with a potent $P2Y_{12}$-inhibitor for one month outweighs the risk of bleeding.

# 6 Trial Objectives and Endpoints

The primary study objective is to test whether an escalated antiplatelet therapy with a potent $P2Y_{12}$-inhibitor (Prasugrel or Ticagrelor) for 4 weeks can reduce ischaemic events without a significant increase in bleeding complications in patients with AF and ST-segment elevation myocardial infarction (STEMI) or non-ST-segment elevation myocardial infarction (NSTEMI) (biomarker positive acute coronary syndrome) undergoing PCI.

The effect of platelet function based on platelet function testing (PFT) on ischaemic and bleeding complications will be investigated in a pre-defined substudy.

## 6.1 Primary Endpoint(s)

Primary endpoints at 6 weeks after randomization (hierarchical testing)

- Efficacy: Major ischaemic events defined as the composite of all-cause mortality, myocardial infarction, definite or probable stent thrombosis, ischaemic stroke or systemic thromboembolism (superiority test)
- Safety: Bleeding type 2 or higher according to the Bleeding Academic Research Consortium (BARC) criteria (non-inferiority test)

## 6.2 Secondary Endpoints

- All individual components of the primary endpoint (all-cause mortality, myocardial infarction, definite or probable stent thrombosis, ischaemic stroke, systemic thromboembolism) at 6 weeks after randomization
- Cardiovascular mortality at 6 weeks after randomization
- Bleeding (BARC type ≥ 2) at 6 weeks after randomization
- Urgent revascularization at 6 weeks after randomization
- All-cause mortality at 6 months after randomization
- Unplanned hospitalization due to acute heart failure or acute coronary syndrome at 6 months after randomization
- Ischaemic stroke at 6 months after randomization

## 6.3 Additional Event Evaluation

- Any bleeding events (BARC type ≥ 1) at 6 weeks after randomization

# 7 Clinical Trial Protocol

## 7.1 Trial Design

This is a prospective, randomized, open-label, multinational European, multicentre, phase IV clinical trial designed to test whether an escalated antiplatelet therapy with a potent $P2Y_{12}$-inhibitor for one month can reduce ischaemic events without a significant increase in bleeding complications in patients with AF and STEMI or NSTEMI undergoing PCI.

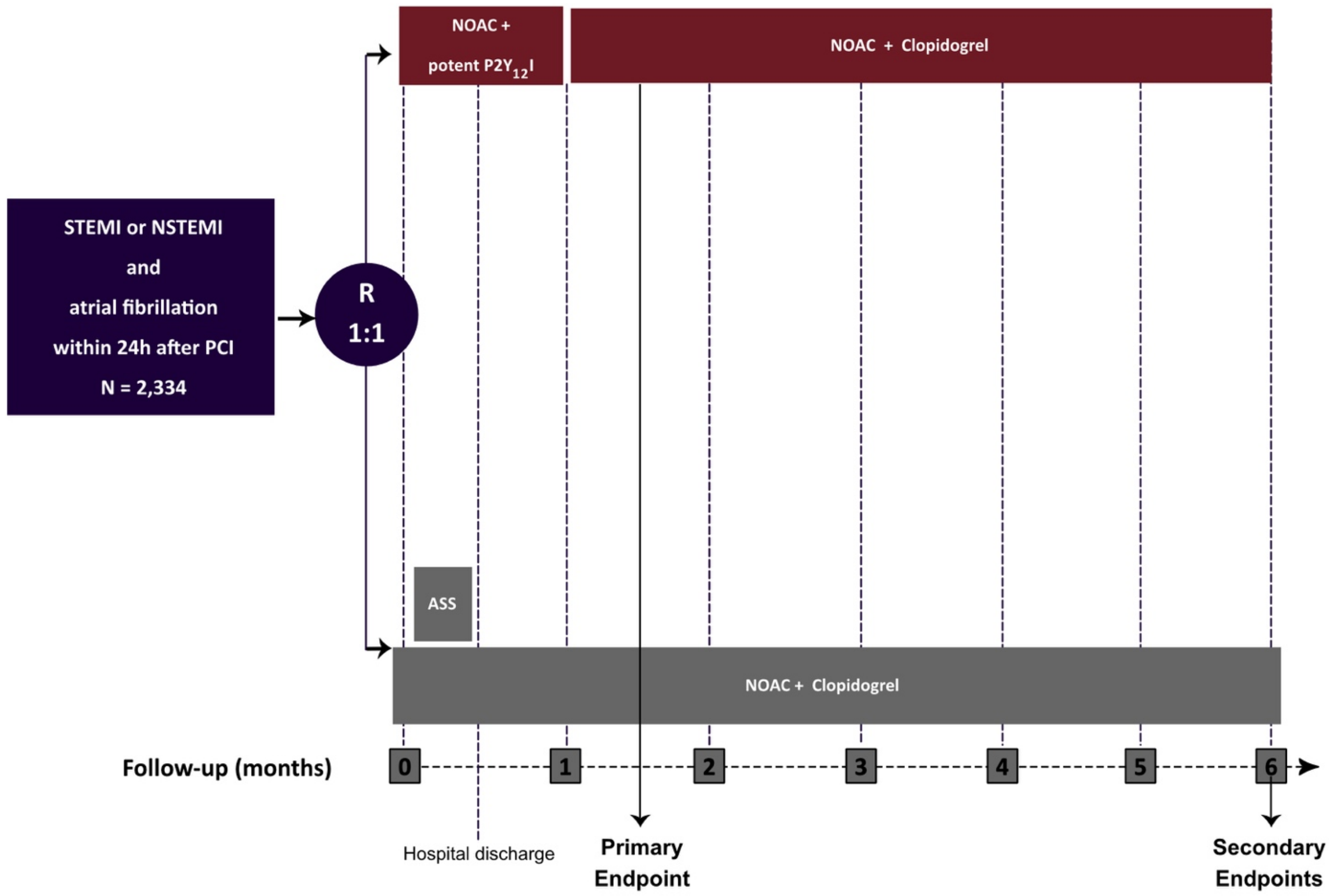


It is planned that the trial is performed in a minimum of 2 countries within the European Union. We anticipate that 50-60 sites will participate in this study.

## 7.2 Trial Duration

Planned start of recruitment: Q4/2021

Planned recruiting period: 24 months

Duration by patient: 6 months

End of Trial: Last patient last visit

# 8 Trial Population

Patients with ACS undergoing PCI and AF with an indication for oral anticoagulation with NOAC, after agreement to participate in the clinical trial will be stratified according to the study centre and randomized to a standard antiplatelet therapy with Clopidogrel or an escalated antiplatelet therapy with a potent $P2Y_{12}$-inhibitor.

It is imperative that the patients fully meet all inclusion criteria and none of the exclusion criteria.

## 8.1 Number of patients

A total number of 2334 patients should be allocated to the trial, a number of 2304 patients should be analysed.

## 8.2 Inclusion Criteria

- Written informed consent
- Age ≥ 18 years
- Atrial fibrillation requiring oral anticoagulation
- STEMI or NSTEMI (biomarker positive acute coronary syndrome) and successful completion of PCI (randomization will take place within 24h after successful PCI)

## 8.3 Exclusion Criteria

- Chronic renal insufficiency with glomerular filtration rate < 15 ml/min/1.73m$^2$
- History of ischaemic stroke or transient ischaemic attack (both contraindications for Prasugrel) and history of intracranial bleeding (contraindication for Ticagrelor)
- Contraindication for Clopidogrel or Aspirin
- Contraindication for Prasugrel and Ticagrelor
- Severe chronic liver disease (Child-Pugh C)
- Indication for oral anticoagulation with Vitamin K antagonists
- Moderate to severe mitral stenosis or mechanical heart valve
- Any bleeding BARC type ≥ 2 within the last 4 weeks before index procedure
- Pregnancy or lactation
- Inability to cooperate with the protocol requirements
- Life expectancy < 6 months
- Participation in another investigational drug study
- Previous enrolment in this study
- For women of childbearing potential no negative pregnancy test and no agree to use a reliable method of birth control during the study
- Previous treatment with GP IIb/IIIa inhibitors within the last 12 hours
- A known genetic disorder involved in the metabolism of the study medication

Women of childbearing potential: include any female who has experienced menarche and who has not undergone successful surgical sterilisation (hysterectomy, bilateral tubal ligation, or bilateral oophorectomy) or is not postmenopausal (amenorrhea ≥ 12 consecutive months)

Reliable methods of birth control: oral contraceptives, other hormonal contraceptives (vaginal products, skin patches, or implanted or injectable products), mechanical products such as an intrauterine device or barrier methods (diaphragm, condoms, spermicides) to prevent pregnancy, or are practicing abstinence or their partner is sterile (e.g. vasectomy).

## 8.4 Patient Recruitment and Screening

Patients presenting with STEMI or NSTEMI to the participating hospital and underwent a successful PCI (defined as TIMI flow grade 2 or more after intervention), who meet all inclusion and no exclusion criteria are eligible for enrolment. Screening is performed within 24 hours after completion of PCI.

Pre-screening lists will be obtained at the trial sites to document patients assessed for eligibility, but not enrolled in the trial.

# 9 Treatment

## 9.1 Trial Treatment

Following enrolment, patients will undergo a 1:1 randomization to a standard therapy (control arm) with an in-hospital TAT (Aspirin, Clopidogrel and NOAC - preferentially Edoxaban or Apixaban) (for a maximum of one week) and a subsequent DAT (NOAC - preferentially Edoxaban or Apixaban plus Clopidogrel) for 6 months or an intensified regimen (experimental arm) of NOAC (preferentially Edoxaban or Apixaban) plus a potent $P2Y_{12}$-inhibitor (Prasugrel (preferred) or Ticagrelor) for the first month (4 weeks) followed by NOAC (preferentially Edoxaban or Apixaban) and Clopidogrel for 5 months. The NOACs allowed in this trial are the anti-Xa-inhibitors Edoxaban, Apixaban and Rivaroxaban. The dose of the $P2Y_{12}$-inhibitors and NOACs is administered as recommended in the Summary of Product Characteristics (SmPC).

The default duration of Aspirin therapy in the control arm is in-hospital (up to 1 week). However, the treating physicians are allowed to extend the therapy with Aspirin in the control arm for a period of up to 4 weeks in patients with high ischaemic risk, according to current guidelines (2,9).

IMP1: Test product

| | |
|---|---|
| Drug name | Efient® |
| Name of manufacturer | Daiichi-Sankyo |
| Substance name (if applicable, give substance code) | Prasugrel (ATC-Code: B01AC22) |
| Name and dose of active ingredient per unit | Prasugrel as hydrochloride, 5 mg or 10 mg |
| Other ingredients | Tablet Core: microcrystalline cellulose, mannitol (E421), croscarmellose sodium, hypromellose (E464), magnesium stearate<br>Film-Coat: lactose monohydrate, hypromellose (E464), titanium dioxide (E171), triacetin (E1518), Iron oxide red (E172), {only Efient 10 mg} iron oxide yellow (E172), talc |
| Pharmaceutical form | Film coated tablet |
| Mode of administration | Oral |
| Batch number (if applicable) | NA |
| Storage conditions | Store in the original package in order to protect from air and moisture. |

Efient® is given as an example. The IMP is only defined by active substance Prasugrel. All other Prasugrel formulations with a marketing authorization in Germany or in the European Union could be used.

Or

IMP2: Test product

| | |
|---|---|
| Drug name | Brilique® |
| Name of manufacturer | AstraZeneca |
| Substance name (if applicable, give substance code) | Ticagrelor (ATC-Code: B01AC24) |
| Name and dose of active ingredient per unit | Ticagrelor 90 mg |
| Other ingredients | Tablet core: mannitol (E421), calcium hydrogen phosphate dihydrate, magnesium stearate (E470b), sodium starch glycolate type A, hydroxypropyl-cellulose (E463)<br>Tablet coating: talc, titanium dioxide (E171), Iron oxide yellow (E172), macrogol 400, hypromellose (E464) |
| Pharmaceutical form | Film coated tablet |
| Mode of administration | Oral |
| Batch number (if applicable) | NA |
| Storage conditions | None |

IMP3: Reference product

| | |
|---|---|
| Drug name | Plavix® |
| Name of manufacturer | Sanofi |
| Substance name (if applicable, give substance code) | Clopidogrel (ATC-Code: B01AC-04) |
| Name and dose of active ingredient per unit | Clopidogrel 75 mg as hydrogen sulfate |
| Other ingredients | Core: mannitol (E421), macrogol 6000, micro-crystalline cellulose, hydrogenated castor oil, low substituted hydroxypropylcellulose<br>Coating: hypromellose (E464), lactose monohydrate, triacetin (E1518), titanium dioxide (E171) |
| Pharmaceutical form | Film coated tablet |
| Mode of administration | oral |
| Batch number (if applicable) | NA |
| Storage conditions | In PVC/PVDC/aluminium blisters, store below 30°C. In all aluminium blisters, this medicinal product does not require any special storage conditions. |

Plavix® is given as an example. The IMP is only defined by active substance Clopidogrel. All other Clopidogrel formulations with a marketing authorization in Germany or in the European Union could be used.

And

IMP4: Reference product

Germany:

| | |
|---|---|
| Drug name | HerzASS-ratiopharm® 100 mg Tabletten |
| Name of manufacturer | ratiopharm GmbH |
| Substance name (if applicable, give substance code) | Acetylsalicylic Acid (ATC-Code: B01AC06) |
| Name and dose of active ingredient per unit | Acetylsalicylic Acid 100 mg |
| Other ingredients | Maize starch, micro-crystalline cellulose, cellulose powder |
| Pharmaceutical form | Tablet |
| Mode of administration | oral |
| Batch number (if applicable) | NA |
| Storage conditions | Do not store above 25°C, store in original packaging to protect the content from humidity |

HerzASS-ratiopharm® 100 mg is given as an example. The IMP is only defined by active substance Acetylsalicylic Acid. All other Acetylsalicylic Acid formulations with a marketing authorization in Germany or in the European Union could be used.

Austria:

| | |
|---|---|
| Drug name | HerzschutzASS "ratiopharm" 100 mg - Tabletten |
| Name of manufacturer | TEVA B.V. |
| Substance name (if applicable, give substance code) | Acetylsalicylic Acid (ATC-Code: B01AC06) |
| Name and dose of active ingredient per unit | Acetylsalicylic Acid 100 mg |
| Other ingredients | Maize starch, micro-crystalline cellulose, cellulose powder |
| Pharmaceutical form | Tablet |
| Mode of administration | oral |
| Batch number (if applicable) | NA |
| Storage conditions | Do not store above 30°C. Do not store above 25°C, store in original packaging to protect the content from humidity |

HerzschutzASS “ratiopharm” 100 mg is given as an example. The IMP is only defined by active substance Acetylsalicylic Acid. All other Acetylsalicylic Acid formulations with a marketing authorization in Austria or in the European Union could be used.

## 9.2 Treatment Arms

### 9.2.1 Experimental intervention

Escalated regimen consisting of

| | |
|---|---|
| Weeks 1 – 4: | Potent $P2Y_{12}$-inhibitor plus non-vitamin K antagonist oral anticoagulant |
| Week 5 – month 6: | Clopidogrel plus non-vitamin K antagonist oral anticoagulant |

### 9.2.2 Control intervention

Standard regimen consisting of

| | |
|---|---|
| In-hospital (up to 1 week): | Aspirin plus Clopidogrel plus non-vitamin K antagonist oral anticoagulant |
| Discharge or day 8 – month 6: | Clopidogrel plus non-vitamin K antagonist oral anticoagulant. In patients with high ischaemic risk the treating physician is allowed to prescribe Aspirin for a period of up to 4 weeks, according to current guidelines. |

## 9.3 Treatment Assignment and Randomization

A randomized parallel group design to control for confounding is used. Patients will be randomized within 24h after completion of successful PCI (transradial approach preferred). Participants will be randomly assigned to one of two treatment arms. Randomization will occur in a 1:1 ratio through use of a permuted-block randomization method to ensure a balanced assignment to each treatment arm. Randomization will be stratified by centre. The randomization list will be uploaded into the randomization module of the EDC system. Study investigators will have access to the randomization module via the eCRF and randomize participants using this central tool.

## 9.4 Packaging, Labelling and Supply

Commercially available IMPs (without special packaging or labelling) are used.

## 9.5 Treatment of Patients

### 9.5.1 Patient Numbering

Each patient is uniquely identified by a Patient ID which is composed by a 2-digit site number followed by a 3-digit subsequent number.

### 9.5.2 Treatment Administration

The patients will be instructed by their treating physicians regarding the intake of IMP after discharge. The IMP will be prescribed by the general practitioner or cardiologist according to the randomized treatment regimen.

If the $P2Y_{12}$-inhibitor loading at index PCI was performed with Clopidogrel or Prasugrel no additional loading with the randomized $P2Y_{12}$-inhibitor will be performed after randomization. If the $P2Y_{12}$-

inhibitor loading at index PCI was performed with Ticagrelor switching to an irreversible $P2Y_{12}$-inhibitor will be done according to current guidelines.

After 4 weeks the patients randomized in the experimental arm should be switched from Prasugrel or Ticagrelor to Clopidogrel by their treating general practitioner or cardiologist. The NOAC treatment remains unchanged. In the control group the patients remain on their treatment with Clopidogrel and NOAC. The IMPs are taken according to the instruction leaflet of the respective medication. If there is an indication, the treating physician is allowed to change the treatment to another anti-Xa-inhibitor, which however should be documented in the eCRF.

### 9.5.3 Compliance

Patients will be interviewed in detail about compliance to study medication at each visit. Patient's compliance will be assessed by the investigator and recorded in the eCRF. Any interruption or termination including the cause of the interruption and termination as well as the exact dates will be documented.

### 9.5.4 Prior and Concomitant Medication

Prior and concomitant medication can be changed and dose might be adjusted according to the decision of the treating physician.

## 9.6 Trial Completion and Discontinuation

### 9.6.1 Trial Completion and Post-Trial Treatment

After the end of the escalated treatment all patients will be treated with NOAC and Clopidogrel for months 2-6. After 6 months, a DAT with Aspirin or Clopidogrel and NOAC, in line with the current guidelines and according to the decision of the treating physician is suggested.

A patient will be considered to have completed the trial when the patient has completed the last visit planned in the protocol (final visit Day 180 ± 10 days).

After study completion patients will be treated at the discretion of the responsible physician. As a result the aspect of contraception after study completion depends on further treatment of the patient in line with the current guidelines and the respective SmPCs.

### 9.6.2 Lost to Follow-up

A patient will be classified as lost to follow-up only if, his/her vital status remains unknown despite multiple attempts to contact him/her in- or out of hospital via telephone, fax, email, or letter until the last scheduled follow-up of the last patient is completed.

### 9.6.3 Discontinuation of Patients from Treatment or Trial Participation

If a patient was withdrawn before completing the trial, the reason for withdrawal must be entered in the patient's file and the eCRF.

Patients will be withdrawn from the trial/treatment for the following reasons:

- withdrawal of consent by the subject for any reason at any time
- if continued trial participation is not in the best interest of the subject, as judged by the investigator (e.g. due to clinical adverse event, laboratory abnormality or inter-current illness)
- termination of the trial by regulatory authorities or ethics committees

- patients, who become prisoners, involuntarily incarcerated or compulsorily detained for treatment of either a psychiatric of physical illness

Patients who discontinue trial treatment prematurely should conduct the follow-up visits as far as possible.

Patients have the right to withdraw their consent at any time without providing a reason without prejudice to their future medical care by the physician or at the institution. The signed informed consent form must remain in the Investigator Site File and must be available for verification by monitors or authorities at any time. Withdrawal of full consent for a study means that the patient does not wish to receive further investigational treatment and does not wish or is unable to continue further study participation. Patient data up to withdrawal of consent will be included in the patient's study data, but no further information will be collected unless a separate consent has been given. Withdrawal of partial consent means that the patient does not wish to take protocol-specific product(s) any longer but is still willing to collaborate in providing further data by continuing on study. Should a patient request or decide to withdraw from the study, all efforts will be made to complete and report the observations as thoroughly as possible up to the end of withdrawal.

For any patient who has received IMP and withdrew prematurely from the study every effort should be made for attendance of a safety follow-up visit. The primary reason for withdrawal from the study should be documented. Patients will not be followed for any reason after consent has been withdrawn, unless a separate consent has been given for further data collection.

### 9.6.4 Conditions for Terminating the Trial by the Sponsor

The sponsor reserves the right to temporarily suspend or prematurely discontinue this trial either at a single site or at all sites at any time for reasons including safety or ethical issues, severe non-compliance, recurrent non-compliance, or unsatisfactory enrolment with respect to quality or quantity.

If the trial is prematurely terminated or suspended, the sponsor will promptly inform the Principal Investigators, the ECs and the regulatory authorities of the termination or suspension and the reason(s) for the termination or suspension.

In case of a premature termination of the trial, patients will be followed-up at least until all enrolled patients underwent their final visit.

# 10 Trial Procedures

A schedule of activities is provided in section 2.

## 10.1 Schedule of Assessments

### 10.1.1 Screening / Baseline Visit

It is the responsibility of the investigator, or a person delegated by the investigator to obtain written informed consent from each patient prior to participation in the trial, following adequate explanation of the aims, methods, anticipated benefits and potential hazards of the trial.

No clinical trial procedures will be conducted prior to taking consent from the participant. The consent will not denote the enrolment into the trial. Data obtained as part of the normal patient care can be used for screening/baseline visit if collected within 72h prior to randomization.

The following procedures are performed:

- Informed consent
- Assessment of in- and exclusion criteria
- Demographic data (age, gender, race), general and cardiovascular medical history and current diseases:
    - Symptoms: angina pectoris (CCS classification), dyspnoea (NYHA classification), syncope
    - Cardiovascular risk factors: Smoking status, arterial hypertension, diabetes mellitus, hypercholesterolaemia, family history of coronary artery disease
    - Cardiovascular history: Previous myocardial infarction, previous PCI, previous CABG, previous stroke, previous stent thrombosis, previous bleeding BARC ≥ 2 (within the last 4 weeks before index procedure), peripheral arterial occlusive disease (PAOD), cerebrovascular disease (CVD), atrial fibrillation, aortic stenosis, aortic insufficiency, mitral insufficiency, mitral stenosis, tricuspid insufficiency, pacemaker/ICD/CRT
    - Medical history: COPD (Gold), chronic renal failure (CRF) and stage, abnormal liver function, history of cancer, alcohol abuse
- Clinical parameters: Weight and height at admission, blood pressure and heart rate post PCI at screening, ECG at admission and post PCI at screening, ECG risk stratification (optional)
- Laboratory parameters (admission): Laboratory tests at admission depend on the routine tests performed in each study centre. We recommend following tests: Haemoglobin, erythrocytes, leukocytes, thrombocytes, creatinine, BUN, CK, troponin, LDH, CRP, BNP/NT-proBNP, HbA1c, total cholesterol, LDL, HDL, GOT, GPT, bilirubin, INR, platelet aggregation tests (substudy at pre-defined sites)
- Laboratory parameters (post PCI and prior to randomization): Laboratory tests post PCI depend on the routine tests performed in each study centre. We recommend following tests: Haemoglobin, CK, troponin.
- Pregnancy test for women of childbearing potential, contraception (if applicable)

- Concomitant medication will be recorded:
    - At admission: Aspirin, $P2Y_{12}$-inhibitors, OAC, beta-blocker, angiotensin-converting-enzyme inhibitors (ACEI), angiotensin II receptor blockers (ARB), aldosterone antagonist, angiotensin-receptor-neprilysin-inhibitor (ARNI), SGLT2 inhibitors, amiodarone, digitalis, calcium channel blockers (CCB), nitrates, statins, non-steroidal anti-inflammatory drugs (NSAIDs)
    - Peri-interventional: Aspirin, $P2Y_{12}$-inhibitors, heparin, GP IIb/IIIa inhibitors
    - At screening : Aspirin, $P2Y_{12}$-inhibitors, OAC, beta-blocker, ACEI/ARB, aldosterone antagonist, ARNI, SGLT2 inhibitors, amiodarone, digitalis, CCB, nitrates, statins
- Coronary angiography/PCI: CAD and vessels involved, PCI number of stents, PCI bifurcation, kissing balloon, PCI ostial, PCI LCA, PCI CABG, PCI planned and which vessels, planned bifurcation, planned ostial stenosis, planned LCA, planned CABG, success of PCI (TIMI grade 2 or more), left-ventricular ejection fraction (LVEF; angiographic or echocardiographic)
- Randomization within 24h after successful completion of PCI with TIMI flow grade 2 or more: All patients who meet the inclusion and none of the exclusion criteria are randomized 1:1 into two groups:

Group 1 (experimental): escalated antiplatelet therapy with a potent $P2Y_{12}$-inhibitor (Prasugrel or Ticagrelor):

Concomitant antiplatelet treatment with Aspirin will be discontinued in this group.

Group 2 (control): treatment with Clopidogrel:

Aspirin will be continued at a maintenance dose of 75-100mg/day (for Germany and Austria only 100 mg are applicable) for the in-hospital stay for a maximum of 7 days in the control group. According to the current guidelines, Aspirin is allowed to be continued for a maximum of 4 weeks in patients with high ischaemic risk.

In both groups:

If the patients are already on treatment with an anti-Xa-inhibitor (Edoxaban, Apixaban or Rivaroxaban) the anti-Xa-inhibitor will be continued.

In patients not on treatment with NOACs or in patients on treatment with VKA or anti-IIa-inhibitor, an anti-Xa-inhibitor will be initiated (preferentially Edoxaban).

### 10.1.2 Visit at Discharge

- Laboratory parameters: CK max, troponin max
- Assessment of adverse events and occurrence of endpoints
- Concomitant medication at discharge: Aspirin, $P2Y_{12}$-inhibitors, OAC, beta-blocker, ACEI/ARB, aldosterone antagonist, ARNI, SGLT2 inhibitors, amiodarone, digitalis, CCB, nitrates, statins

### 10.1.3 Control Visit (Day 42 ± 7)

The visit can be performed either in the hospital, by phone or letter (for patients not included in the substudy).

- Assessment of adverse events and occurrence of endpoints
- Symptoms: angina pectoris (CCS classification), dyspnoea (NYHA classification), syncope
- Concomitant medication: Aspirin, $P2Y_{12}$-inhibitors, OAC, beta-blocker, ACEI/ARB, aldosterone antagonist, ARNI, SGLT2 inhibitors, amiodarone, digitalis, CCB, nitrates, statins
- Laboratory: Laboratory parameters that were assessed after discharge of index hospital stay (if applicable)

### 10.1.4 Final Visit (Day 180 ± 10)

The visit can be performed either in the hospital, by phone or letter.

- Assessment of adverse events and occurrence of endpoints
- Concomitant medication: Aspirin, $P2Y_{12}$-inhibitors, OAC
- Smoking status

# 11 Safety Monitoring

## 11.1 Definitions

Definitions have been taken from the Regulation (EU) No 536/2014 of the European Parliament and of the council of 16 April 2014 on clinical trials on medicinal products for human use.

### 11.1.1 Adverse Event (AE)

An Adverse Event is any untoward medical occurrence in a subject to whom a medicinal product is administered and which does not necessarily have a causal relationship with this treatment.

An AE can therefore be any unfavourable and unintended sign (including an abnormal laboratory finding, for example), symptom, or disease temporally associated with the use of a medicinal product whether or not considered related to the medicinal product.

Pre-existing diseases or symptoms or abnormal lab values present before randomization should not be considered an AE unless the condition worsens or episodes increase in frequency during trial participation.

### 11.1.2 Adverse Reaction (AR)

In determining whether an adverse event is an adverse reaction, considerations shall be given to whether there is a reasonable possibility of establishing a causal relationship between the event and the investigational medicinal product based on an analysis of available evidence.

### 11.1.3 Serious Adverse Event (SAE)

A Serious Adverse Event is any untoward medical occurrence that at any dose

- results in death
- is life-threatening

- requires inpatient hospitalization or prolongation of existing hospitalization
- results in persistent or significant disability or incapacity
- results in a congenital anomaly or birth defect
- is qualified as another important medical event or condition (some medical events may jeopardize the patient or may require an intervention to prevent one of the above characteristics/consequences). Such events should also be considered as serious in accordance with the definition.

These characteristics/consequences have to be considered at the time of the event. For example, regarding a life-threatening event, this refers to an event in which the subject was at risk of death at the time of the event; it does not refer to an event which hypothetically might have caused death if it were more severe.

### 11.1.4 Suspected Unexpected Serious Adverse Reaction (SUSAR)

An Unexpected Serious Adverse Reaction is a serious adverse reaction, the nature, severity or outcome of which is not consistent with the reference safety information. The reference safety information for the IMPs is the effective SmPC of Efient®, Brilique®, Plavix® and HerzASS-ratiopharm® 100 mg (Germany) or HerzschutzASS “ratiopharm” 100 mg (Austria), respectively.

## 11.2 Assessment Criteria

### 11.2.1 Adverse Event Assessment by the Investigator

The investigator should evaluate all AEs according to the criteria and steps mentioned below.

#### 11.2.1.1 Assessment of Seriousness

For each AE the investigator determines its seriousness according to the definition provided in section 11.1.3. In case the AE fulfils one or several seriousness criteria, the SAE has to be recorded on the SAE page of the eCRF and reported to the sponsor and the designee responsible of safety evaluation immediately.

#### 11.2.1.2 Assessment of Intensity

All AEs have to be graded regarding their intensity.

| | |
|---|---|
| MILD | The patient is aware of the sign or symptom, but it does not interfere with his/her usual activities and/or it is of no clinical consequence. |
| MODERATE | The AE interferes with the usual activities of the patient or it is of some clinical consequence. |
| SEVERE | The AE interferes significantly with patient’s usual function, incapacitating with inability to work or carry out usual activity or the AE is of definite clinical consequence. |

#### 11.2.1.3 Assessment of Causality

The relationship (causality) of an AE to the investigational product(s) is evaluated according to the following classification:

| | |
|---|---|
| Not related | There is not a reasonable possibility that the AE is related to the IMP. Alternative aetiology, diagnosis or explanation for the event should be provided. |

| | |
|---|---|
| Related | There is a reasonable possibility that the AE is related to the IMP. This means that there are facts (evidence) or arguments to suggest a causal relationship. |

The following should be taken into consideration:

- a temporal relationship
- a pharmacologically or biologically plausible event
- positive de-challenge after stopping respectively re-challenge after restarting of IMP
- presence of confounding factors, such as concomitant medication, concurrent illness or relevant medical history

### 11.2.2 Assessment by the Sponsor

In order to fulfil the sponsor's responsibilities of ongoing safety evaluation and reporting obligations, the sponsor performs a sponsor assessment of each reported SAE concerning seriousness, causality and whether it is expected or not. The reference safety information for classification of expected or unexpected SAEs are the current versions of the Summaries of Product Characteristics (SmPC) of Efient®, Brilique®, Plavix® and HerzASS-ratiopharm® 100 mg (Germany) or HerzschutzASS "ratiopharm" 100 mg (Austria).

In addition, a risk-benefit evaluation is performed by the sponsor describing whether the SAE report implies a safety issue that

- might alter the risk-benefit assessment

- might be sufficient to consider changes in the conduct of the study.

## 11.3 Collection and Reporting of (Serious) Adverse Events

### 11.3.1 Adverse Events

All AEs which occur after randomization until the 6 months follow-up visit (final visit) must be collected by the investigator, recorded on the patient file and entered on the AE page of the eCRF.

The patient will be given the opportunity to report AEs spontaneously to the Investigator. At each study visit after randomization, the patient has to be asked by non-directive questioning about his health status and the occurrence of events, e.g., "Did you notice anything unusual about your health (since your last visit)?" In order to prevent reporting bias, subjects should not be questioned regarding the specific occurrence of one or more AEs. Nevertheless the investigator must ensure that endpoints are captured.

Abnormal laboratory values or test results constitute adverse events only if they fulfil at least one of the following criteria *(to be adapted accordingly)*:

- they induce clinical signs or symptoms,
- they are considered clinically significant,
- they require therapy,
- they lead to a change in IMP dosing or discontinuation of the IMP

Clinically significant abnormal laboratory values or test results must be identified through a review of values outside of normal ranges, significant changes from baseline or the previous visit, or values which are considered to be non-typical in patients with the underlying disease.

If known, the diagnosis of the underlying illness or disorder should be recorded by the investigator, rather than its individual symptoms. All signs, symptoms, and laboratory test findings related to the underlying diagnosis should be collectively recorded as a single diagnosis. The following information should be captured for all AEs: onset, stop date (duration), intensity, seriousness, relationship to investigational medicinal product, action taken, treatment required and outcome. If treatment for the AE was administered, it should be recorded on the concomitant medication page of the eCRF.

AEs should be followed up until resolution, stabilization or deemed irreversible.

### 11.3.2 Serious Adverse Events

#### 11.3.2.1 Content of SAE Reports and Reporting

If an AE fulfils one or more of the seriousness criteria an SAE report has to be completed in addition to the documentation on the AE page of the eCRF.

All SAEs must be reported by the investigator to the sponsor immediately (latest within 24 hours) after becoming aware of the event by entering the event in the respective SAE section of the eCRF. In some instances when the eCRF is not available the SAE should be reported by confirmed facsimile transmission (fax). If only limited information is initially available, follow-up reports are required. If an ongoing SAE changes in its intensity or relationship to the investigational product, a follow-up SAE report should be sent immediately to the sponsor. As follow-up information becomes available it should be sent immediately using the same procedure used for transmitting the initial SAE report. All SAEs should be followed up until resolution, stabilization or deemed irreversible.

The SAE report must contain at least the following criteria for a valid report:

- Patient identification (patient ID)
- SAE (diagnosis/symptom/description)
- Suspect IMP
- Reporting source (name, date and signature of the reporting investigator)

The following information should be provided on the SAE report in addition:

- Start date and time (time only during hospitalization for index procedure)
- Seriousness criteria
- IMP administration
- Action taken concerning IMP, de- / re-challenge
- Causality of SAE to IMP
- Other potential causes for SAE occurrence
- Intensity of the SAE
- SAE treatment
- Outcome and end date of the SAE
- Concomitant diseases and concomitant medication
- Laboratory values and supporting information (e.g. discharge letters, results of examinations)

A detailed guideline regarding completion of SAE pages in the eCRF and SAE reporting will be provided to the investigators.

#### 11.3.2.2 Exclusion from Reporting of SAEs

- the following hospitalizations are not considered SAEs: a visit to the emergency room or other hospital department < 24 hours, that does not result in admission (unless considered “important medical event” or “life threatening” event)
- routine health assessment requiring admission for baseline/trending of health status (e.g. routine colonoscopy)
- medical admission for purpose other than remedying ill health state and was planned prior to signing informed consent. Appropriate documentation is required in these cases.
- admission for social reasons encountered for another life circumstance that carries no bearing on health status and requires no medical/surgical intervention (e.g. lack of housing, economic inadequacy, care-giver respite, family circumstances, administrative)
- scheduled hospitalizations for diagnostic or administrative procedures not related to study procedures
- hospitalization due to logistical or geographical reasons (patient’s home too remote for outpatient treatment)

#### 11.3.2.3 Period of SAE Reporting

All SAEs which occur after randomization until the 6 month follow-up visit must be reported on the SAE Report Form provided in the eCRF to the sponsor.

## 11.4 Expedited reporting

If a Serious Adverse Event is assessed as a Suspected Unexpected Serious Adverse Reaction (SUSAR), the sponsor or his designee has to submit the event to Eudravigilance and the Competent Authorities. The leading Ethics Committees will be informed as well as the participating investigators.

## 11.5 Reporting of Trial Treatment Errors including Misuse / Abuse

The occurrence of treatment errors of the IMP including misuse and abuse has to be documented and reported as AE/SAE.

## 11.6 Pregnancy Reporting

In case of a pregnancy at the time of investigational product exposure or during follow-up period, the sponsor or his designee has to be notified by the investigator. The reporting obligation of the investigator starts immediately after coming to know of the patient being pregnant. The pregnancy has to be reported by the investigator on the respective Pregnancy Report Form. The investigator actively follows up on the further course of the pregnancy until delivery and on the condition of the new born.

If the investigator obtains knowledge of adverse events of the pregnant patient, the mother or the new born child, which fulfil the serious criteria specified in the protocol, the protocol specific documentation and reporting procedures apply. The sponsor evaluates the case of pregnancy regarding possible consequences for the pregnant patient, for other patients and/or the progress of the trial.

## 11.7 Annual Safety Report

The sponsor provides an Annual Safety Report including appendices such as line listing of Serious Adverse Reactions and cumulative summary tabulation of SAEs. The Annual Safety Report is submitted to the Competent Authorities and the Leading Ethics Committees.

# 12 Statistics

## 12.1 Sample Size Calculation

Assumptions for sample-size calculation (hierarchical testing)

- Primary efficacy endpoint (ischaemic events) at 6 weeks

  Superiority testing, 2-sided alpha 5%, power 80%, 6-weeks event-rate in the control group 7% (ISAR-REACT 5 study, data not published), 6-weeks event-rate in the intervention group 4.3%, corresponding to an odds-ratio of 0.6 (7,14).

- Number per group: 1147Primary safety endpoint (bleeding BARC ≥ 2) at 6 weeks

  Non-inferiority testing, when the sample size in each group is 1,152, a two-group large-sample normal approximation test of proportions with a one-sided 2.5% significance level will have 80% power to reject the null hypothesis that the test and the standard are not equivalent (6-weeks event-rate in the control group 13.1% (ISAR-REACT 5 study, data not published), odds-ratio between experimental and control group 0.95, difference in proportions, $\pi_1$ - $\pi_0$, is -0,033 or farther from zero in the same direction) in favour of the alternative hypothesis that the proportions in the two groups are equivalent.

  Number per group: 1152

The sample size of the trial is given by the maximum of both sample sizes: 1152 per group.

The computation assumed an allocation ratio $n_{control}/n_{experimental}$ = 1.0, an accrual time of 24 months, a follow-up time of 6 months and an annual 10% follow-up drop-out rate (6-week rate 1.25%, 2304 * 1.0125 = 2334).

## 12.2 Definition of Population Included in the Analysis

The analysis of the primary and secondary endpoints will be performed according to the intention to treat (ITT) principle, i.e., in all patients that have been randomized irrespective of their protocol adherence or continued participation in the study.

The safety endpoint of bleeding complications will be analysed in a modified intention to treat (mITT) population, i.e. in all randomized patients that received the randomly assigned study medication with endpoints occurring within 7 days after drug discontinuation.

## 12.3 Statistical Methods

This is a non-blinded, randomized, multicentre trial. We use random effect (to adjust for centres) logistic regression (to adjust for treatment and strata) to analyse the data. We will use hierarchical testing to assess the primary endpoints (efficacy and safety). We will also use Kaplan-Maier estimates to visualize time-to specific events (ischaemic events and bleeding events). A similar approach will be used for secondary endpoints. The exact elaboration of the analysis takes place in a statistical analysis plan (SAP), which will be available before the first evaluation.

As described, this is a study with several endpoints. This leads to two hierarchical checks.

Efficacy endpoint:

Superiority testing, 2-sided alpha 5%, power 80%, 6 weeks event-probability in the control group based on a post-hoc analysis of the ISAR-REACT 5 trial 7% (data not published), 6 weeks event-probability in the intervention group 4.3%, corresponding to an odds-ratio of 0.67 (7,14).

Null hypothesis H0: OR =1 (difference). For a two-sided alpha of 0.05, 6-week event probability in the control group of 0.070 and in the intervention group of 0.043 (OR = 0.6) the power is 80.0% if the chi-square test is performed on 1147 patients per group. The computation assumes an allocation ratio (n2/n1) = 1.0.

Safety endpoint:

Non-inferiority testing, when the sample size in each group is 1152, a two-group large-sample normal approximation test of proportions with a one-sided 2.5% significance level will have 80% power to reject the null hypothesis that the test and the standard are not equivalent (6-week event-rate in the control group 13.1% (based on unpublished data of the ISAR-REACT 5 trial), odds-ratio between treatment and control-group 0.95, difference in proportions, $\pi_1 - \pi_0$, is -0,033 or farther from zero in the same direction) in favour of the alternative hypothesis that the proportions in the two groups are equivalent.

## 12.4 Pre-specified subgroup analysis

Furthermore, the following subgroups will be examined:

- Age (Cut-off ≥/< 65)
- Gender
- STEMI or NSTEMI
- AF (paroxysmal or not)
- Apixaban, Edoxaban, Rivaroxaban
- Ticagrelor, Prasugrel
- On treatment with $P2Y_{12}$-inhibitor
- On treatment with Aspirin (≤ 7 days or > 7 days)
- On treatment with OAC
- Multivessel disease yes or no
- Bifurcation, ostial, 3-vessel PCI
- Peripheral arterial occlusive disease (PAOD) or cerebrovascular disease (CVD)
- CHA2DS2-VASc ≥ 3
- HAS-BLED ≥ 3
- Previous stroke
- Previous MI
- NYHA > II

- BNP cut-off median
- eGFR < 60 mL/min/1.73m$^2$
- Max CK > 1000 U/L during the first 48h after the index myocardial infarction
- Increased PRD or decreased DC

## 12.5 Handling of Protocol Deviations and Missing Data

Missing values in baseline, laboratory and clinical characteristics will be replaced by imputation procedures.

All analyses should be performed in an intention to treat basis. The safety endpoint of bleeding complications will be analysed in a modified intention to treat (mITT) population, i.e. in all randomized patients that received the randomly assigned study medication with endpoints occurring within 7 days after drug discontinuation.

In addition, we will perform per-protocol (as treated) analyses as sensitivity analyses for all primary and secondary endpoints.

# 13 Substudy

Platelet function test substudy:

In a predefined substudy of EPIDAURUS (EPIDAURUS-PFT), the effect of platelet function on the primary and secondary endpoints will be examined. This substudy will clarify the debated role of platelet reactivity as a potential determinant for cardiovascular outcome. Platelet function testing will be applied in the centres, where PFT-assays are available and the centres agree to participate in the substudy. Samples will be drawn after randomization (not less than 4 hours after loading and not later than 2 hours after randomization) and 24h after randomization. In addition, a platelet function testing is recommended but is not obligatory at the 6-week clinical visit. All commercially available assays are allowed to be used in the substudy. At the end of the study the effect of PFT-testing on the primary and secondary endpoints will be examined using multivariable cox-regression analysis.

# 14 Quality Assurance and Control

## 14.1 Routine Monitoring

Monitors will visit the site to check completeness of patient records, accuracy of data capture / data entry, adherence to the protocol and to Good Clinical Practice and progress of enrolment.

The extent and frequency of monitoring and percentage of source data verification performed is defined in the Monitoring Plan.

The investigator agrees to cooperate with the monitor to ensure that any problems detected in the course of these monitoring visits are addressed and resolved. The investigator allows the monitor to have access to trial related original data and documents relevant for monitoring of the trial.

## 14.2 Compliance with Protocol and Protocol Revisions

The investigator should conduct the trial in compliance with the protocol agreed to by sponsor, the Regulatory Authorities and for which an approval by the ECs was given.

Any significant protocol deviation will be documented and explained by the investigator or the person designated by the investigator in the eCRF and reported to the sponsor or his designee and will be included in the clinical trial report. Corrective and preventive actions (CAPA) may be developed by the site and/or the sponsor or his designee, implemented and followed up as appropriate.

The study shall be conducted as described in this approved protocol. All revisions to the protocol must be discussed with, and be prepared by sponsor. The investigator should not implement any deviation or change to the protocol without prior review and documented approval/favourable opinion from the IEC of an amendment, except where necessary to eliminate an immediate hazard(s) to study subjects. Any deviation must be documented in the eCRF.

If a deviation or change to a protocol is implemented to eliminate an immediate hazard(s) prior to obtaining EC's favourable opinion, as soon as possible the deviation or change will be submitted to:

- Sponsor
- ECs for review and approval/favourable opinion

The favourable opinion signed by the chairperson or designee of the ECs must be sent to the sponsor. If an amendment substantially alters the study design or increases the potential risk to the subject:

(1) the informed consent form must be revised and submitted to the ECs for review and favourable opinion;

(2) the revised informed consent form must be used to obtain consent from patients currently enrolled in the study if they are affected by the amendment; and

(3) the new informed consent form must be used to obtain consent from new patients prior to enrolment.

## 14.3 Measures of Quality Assurance and Quality Control

### 14.3.1 Quality Control

Quality Control will be applied to each stage of data handling to ensure that all data are reliable and have been processed correctly, e.g. by source data verification by monitors and data management procedures. A comprehensive Quality Management System with written SOPs and procedures is in place covering all processes and procedures to ensure a compliant conduct of the trial and that data are generated, recorded and reported in compliance with the protocol, GCP, and the applicable regulatory requirement(s).

### 14.3.2 Quality Assurance

An independent Quality Assurance is established at the Deutsches Herzzentrum München (Quality Management Representative Clinical Research). For this clinical trial an audit plan is developed and implemented according to respective SOPs comprising audits of service provider and investigator sites. In addition, regular systems audits are conducted at the ISAResearch Center, Deutsches Herzzentrum München as a service provider, to ensure independent review of trial related processes and procedures including follow-up and closure of CAPAs.

The sponsor ensures documented oversight of all contracted trial-related duties and functions including all trial-related duties and functions that are subcontracted to another party by the sponsor's contracted CRO.

Direct access to all trial related sites, source data, documents and reports for the purpose of auditing by the sponsor and inspection by domestic or foreign regulatory authorities is ensured.

# 15 Committees

## 15.1 Steering Committee

The steering committee is responsible for overseeing the good execution and administrative progress of the protocol. The committee meets regularly to monitor patient accrual, non-compliance with the protocol at individual centres, to act upon recommendations of the Data Safety Monitoring Board, and to determine the policy regarding individual publications arising from data generated from the performance of the trial.

## 15.2 Data Safety Monitoring Board

The Data Safety Monitoring Board (DSMB) is responsible for assessing the progress, safety data, integrity and critical efficacy endpoints of the trial. The main task of the DSMB is to monitor the safety of the participants of the clinical trial. Therefore the DSMB is provided with the AEs and SAEs that occurred during the conduct of the trial. Based on its review the DSMB provides the sponsor with recommendations regarding trial modification, continuation or termination. All final decisions, however, regarding trial termination or modification rest with the Steering Committee.

The sponsor decides on the selection of the DSMB members. The DSMB members are independent from the sponsor and competing interests. Detailed processes are described in the DSMB Charter.

## 15.3 Event Adjudication Committee

The Event Adjudication Committee (EAC) will adjudicate suspected endpoints in order to provide a standardized, systematic, independent and unbiased assessment. The members will review the events reported by the investigators as endpoints to determine whether they meet the specified criteria of endpoint definition. The additional endpoint bleeding events BARC type ≥ 1 at 6 weeks are not subject of EAC adjudication. During event adjudication, members of the committee are blinded to the randomized treatment received by the patient. More details are outlined in the EAC charter.

The Principal Investigator provides all source documents relevant for the adjudication to the sponsor, e.g. medical history, laboratory test results, treatment and diagnostic reports, admission / discharge reports, autopsy reports, if applicable. All patient related data are transmitted as strictly pseudonymised data.

# 16 Data Handling and Record Keeping

## 16.1 Case Report Forms

Site investigators or authorized study personnel enter the required data for this study into pass-word protected, web-based electronic case report forms (eCRFs). The eCRF contains the patients` data and examination results that are needed to present the study results and to perform protocol-specified data analyses. It must be ensured that the eCRFs only contain pseudonymised data. Any change or correction to eCRF will be documented in the audit trail.

Detailed instructions for the use of the eCRFs are provided in the eCRF User Guide. Inconsistencies in eCRFs are addressed to the investigator by electronic queries and that the site shall solve in a

timely manner, adapt the patient data accordingly and that the changes are approved by electronic signature.

All personnel with access to the electronic data capture (EDC) system are supported by a user helpdesk staffed with trained personnel to answer questions and ensure access is maintained such that data entry can proceed in a timely manner.

Investigators ensure that all eCRF forms are filled in a timely fashion as stated below and according to GCP-ICH guidelines. The investigator also ensures the accuracy, completeness, legibility, and timeliness of the data reported to the sponsor in the eCRFs and in all required reports. Data reported in the eCRF should be consistent with the source documents or the discrepancies should be explained. The entered data of the electronic database have to be approved by electronic signature of the investigator.

## 16.2 Source Data

Source documents are original records in which raw data are first recorded. These may be: hospital/clinic/general practitioner records, charts, diaries, x-rays, laboratory results, ECG, and other printouts, pharmacy records, care records, completed psychometric scales, daily record cards, quality of life questionnaires, etc. All source documents must be accurate, clear, unambiguous, permanent and capable of being audited. They should be made using a permanent form of recording (e.g. ink, typing, printing, optical disc).

Hospital/clinic/medical files that are computer generated and stored on magnetic support media may be required to be printed and signed and dated by the investigator.

All information on CRFs must be traceable to these source documents in the patient's file. The investigator must also keep the original Informed Consent Form signed by the patient (a second originally signed copy is given to the patient).

The site must implement processes to ensure availability of all required source documentation. A source document list will be used at the site to identify the source data for data points collected and the monitor will work with the site to complete this.

The investigator must give the monitor access to all relevant source documents to confirm their consistency with the CRF entries.

## 16.3 Data Management

### 16.3.1 Data Handling and Quality Control

The integrity of the database is the basis of the final statistical analysis. According to the study's data validation plan (DVP) automatic edit checks will be implemented into the eCRF in order to detect data discrepancies. The monitor, pharmacovigilance management and the data management are allowed to create manual queries in the eCRF. Both automated and manual queries are essential to guarantee data quality. The sites take responsibility to clarify, confirm or change questionable data accordingly. All query answers provided by the sites are reviewed closely by the monitor, pharmacovigilance management or the data management in order to decide whether a query may be considered closed or not and if a re-query is required.

The EDC system contains a system-generated audit trail that captures any changes made to a data field, including who made the change, why the change was made and the date and time it was made. This information is available both at the investigator's site and at the sponsor's. Data entries made

in the EDC screens are supported by source documents maintained for all subjects enrolled in this study.

Data of screening failures will be recorded at least as source data, as far as the reason for the premature discontinuation is identifiable. For screening failures only in- and exclusion criteria and the trial termination page have to be completed in the eCRF.

All personnel with access to the EDC system are supported by a user helpdesk staffed with trained personnel to answer questions and ensure access is maintained such that data entry can proceed in a timely manner.

Prior to the documentation of the first patient record (FPFV) in the trial data base a finalized and a fully approved Data Management Plan (DMP) has to be in place. This plan will define all study specific data management processes in detail and has to be updated and newly approved once changes in data management processes during the conduct of the study should take effect.

All other details are described in the Data Management Plan.

### 16.3.2 Data Coding

For data coding (e.g. AEs), internationally recognized and accepted dictionaries (e.g. MedDRA Version 24.0 or higher) will be used.

### 16.3.3 Database Lock

The data base lock will only take place in accordance with the applicable SOP for data base lock to ensure that:

- all study data have been entered accordingly
- decisions have been agreed upon on how individual protocol violations are to be dealt with
- written authorisation from the sponsor has been granted

## 16.4 Data Protection and Confidentiality

The investigator must assure that patients´ identities are protected from access by unauthorized parties. No information in source documents about the identity (e.g. name, date of birth, address, and case numbers of the patients) will be disclosed. The investigator keeps a patient identification log relating codes to the names of the patients. Any party with direct access to source data / source documents should take all reasonable precautions within the constraints of the applicable regulatory requirements to maintain confidentiality of patients' identities and sponsor's proprietary information.

## 16.5 Archiving and Data Retention

All records are to be retained by the Principal Investigator for a minimum of 15 years after completion of the trial, i.e. according to the applicable regulations and guidelines. The Principal Investigator will contact the sponsor for authorization prior to destruction of any trial records or in the event of accidental loss or destruction of any trial records.

The sponsor must retain all essential documents (Trial Master File) including the Case Report Form for the duration of at least 25 years after the end of the trial.

## 16.6 Access to Source Data/Documents / Audits and Inspections

The Principal Investigator permits trial-related audits by auditors mandated by the sponsor or his designee and inspections by domestic or foreign regulatory authorities after reasonable notice. The main purposes of an audit or inspection are to confirm that the rights and well-being of the patients enrolled have been protected, and that all data relevant for the evaluation of the investigational product have been processed and reported in compliance with the planned arrangements, ICH GCP and applicable regulatory requirements. The investigator will provide direct access to all trial documents, CRFs, source records and source data. If a regulatory inspection is announced, the Principal Investigator must immediately inform the sponsor and promptly forwards copies of inspection reports to the sponsor.

Representatives of the sponsor must be allowed to visit all trial sites periodically to assess the data quality and integrity.

# 17 Ethical, Regulatory and Administrative Aspects

## 17.1 Good Clinical Practice

The clinical trial will be performed in compliance with the protocol, the Declaration of Helsinki (2013), ICH E6 (R2), Directive 2001/20/EC and Regulation (EU) No 536/2014, as applicable, and applicable local laws and regulations.

Personnel involved in conducting this clinical trial is qualified by education, training, and experience to perform their respective task(s).

## 17.2 Ethics Commission and Regulatory Authorities

It is the responsibility of the sponsor to obtain approval for the trial protocol from the Competent Authorities (CA) and the ECs according to local regulatory requirements.

During the clinical trial any amendment or modification to the clinical trial protocol must be submitted to the Competent Authorities and ECs. The CA/EC have to be informed of any event likely to affect the safety of the patients or the conduct of the trial and/or has an impact on the risk-benefit relation of the clinical trial. All updates of the SmPCs will be sent to the CA/EC.

At the end of the trial the sponsor will notify the CA/EC about trial completion.

In addition, the local regulatory authorities have to be notified about the trial and the participating German sites before the initiation as well as any amendments and modifications throughout the conduct of the trial.

## 17.3 Informed Consent

Prior to enrolment in the trial, the investigator will inform the patient orally and in writing about scope and purpose, and possible risks / benefits of the trial as well as his/her rights and duties in a lay language. Patients must be informed that their records will be subject to review by monitors, sponsor and regulatory agency representatives. Patients must be assured that they may withdraw from the trial at any time for any reason and receive alternative conventional therapy as indicated.

By signing and dating the consent form, the patient attests that the information in the consent form and any other written information was accurately explained and apparently understood, and that the patient freely gave his/her consent. In addition, the Investigator who obtains the consent must sign

and date the consent form. Failure to obtain such a signed form renders the subject ineligible for trial.

An originally signed copy of the form must be kept on the patient's file; a second originally signed copy will be handed over to the trial participant. This is documented in the patient's file.

# 18 Financing

The clinical trial is financed by the Daiichi-Sankyo Deutschland GmbH and Deutsches Zentrum für Herz-Kreislauf-Forschung e.V. (DZHK).

# 19 Insurance

The sponsor provides an insurance for the patients for the total duration of the trial for compensation for any damage suffered by the patient resulting from participation in the clinical trial. The investigator provides the patient with the term and conditions and the certificate of insurance. In case of injury or disability deriving from participation in the study, the patient is requested to inform without delay the insurance company and the treating physician responsible for the trial.

Germany:

HDI-Gerling Industrie Versicherung AG

Niederlassung München

Ganghoferstr. 37 - 39

80339 München, Deutschland

Austria:

AXA XL

Tuchlauben 3

1010 Wien, Österreich

# 20 Clinical Trial Report and Publication Policy

Prior to start of recruitment the trial must be registered in the publicly accessible database "clinicaltrials.gov".

A clinical trial report should be written within 12 months (after completion of the trial (last patient last visit)) and submitted to the relevant Ethics Committees and Regulatory Authorities in accordance with local requirements.

Furthermore, publications in peer-reviewed medical journals are planned. The information generated by this trial is the property of the sponsor. It is agreed that the results of the trial will not be submitted for presentation, abstract, poster exhibition or publication until the sponsor has reviewed and commented on such a presentation or publication manuscript. The sponsor does not hold the overall right to veto publications and presentations. The sponsor of the study is committed to the unrestricted and widespread dissemination of the results of the study. Upon completion of the study, an abstract reporting the primary results will be prepared by the Steering Committee and presented at an annual scientific cardiology meeting. A manuscript will similarly be prepared for publication.

The publication of the principal results from any single-centre experience within the trial is not allowed before both the preparation and publication of the primary results.

Following analysis and presentation of the primary endpoint results, active participation of all committee members, investigators from high enrolling sites, and core laboratory personnel will be solicited for data analysis, abstract and manuscript preparation.

## 21 References


1. Valgimigli M, Bueno H et al. 2017 ESC focused update on dual antiplatelet therapy in coronary artery disease developed in collaboration with EACTS: The Task Force for dual antiplatelet therapy in coronary artery disease of the European Society of Cardiology (ESC) and of the European Association for Cardio-Thoracic Surgery (EACTS). European Heart Journal 2017;39:213-260.
2. Neumann F-J, Sousa-Uva M et al. 2018 ESC/EACTS Guidelines on myocardial revascularization. European Heart Journal 2018;40:87-165.
3. Vranckx P, Valgimigli M et al. Edoxaban-based versus vitamin K antagonist-based antithrombotic regimen after successful coronary stenting in patients with atrial fibrillation (ENTRUST-AF PCI): a randomised, open-label, phase 3b trial. Lancet 2019;394:1335-1343.
4. Lopes RD, Heizer G et al. Antithrombotic Therapy after Acute Coronary Syndrome or PCI in Atrial Fibrillation. N Engl J Med 2019;380:1509-1524.
5. Cannon CP, Bhatt DL et al. Dual Antithrombotic Therapy with Dabigatran after PCI in Atrial Fibrillation. N Engl J Med 2017;377:1513-1524.
6. Gibson CM, Mehran R et al. Prevention of Bleeding in Patients with Atrial Fibrillation Undergoing PCI. N Engl J Med 2016;375:2423-2434.
7. Windecker S, Lopes RD et al. Antithrombotic Therapy in Patients With Atrial Fibrillation and Acute Coronary Syndrome Treated Medically or With Percutaneous Coronary Intervention or Undergoing Elective Percutaneous Coronary Intervention. Circulation 2019;140:1921-1932.
8. Lopes RD, Leonardi S et al. Stent Thrombosis in Patients With Atrial Fibrillation Undergoing Coronary Stenting in the AUGUSTUS Trial. Circulation 2020;141:781-783.
9. Collet JP, Thiele H et al. 2020 ESC Guidelines for the management of acute coronary syndromes in patients presenting without persistent ST-segment elevation. Eur Heart J 2020.
10. Gargiulo G, Goette A et al. Safety and efficacy outcomes of double vs. triple antithrombotic therapy in patients with atrial fibrillation following percutaneous coronary intervention: a systematic review and meta-analysis of non-vitamin K antagonist oral anticoagulant-based randomized clinical trials. Eur Heart J 2019;40:3757-3767.
11. Sibbing D, Aradi D et al. Guided de-escalation of antiplatelet treatment in patients with acute coronary syndrome undergoing percutaneous coronary intervention (TROPICAL-ACS): a randomised, open-label, multicentre trial. Lancet 2017;390:1747-1757.
12. Antman EM, Wiviott SD et al. Early and late benefits of prasugrel in patients with acute coronary syndromes undergoing percutaneous coronary intervention: a TRITON-TIMI 38 (TRial to Assess Improvement in Therapeutic Outcomes by Optimizing Platelet InhibitioN with Prasugrel-Thrombolysis In Myocardial Infarction) analysis. J Am Coll Cardiol 2008;51:2028-33.
13. Angiolillo DJ, Rollini F et al. International Expert Consensus on Switching Platelet P2Y(12) Receptor-Inhibiting Therapies. Circulation 2017;136:1955-1975.
14. Wiviott SD, Trenk D et al. Prasugrel compared with high loading- and maintenance-dose clopidogrel in patients with planned percutaneous coronary intervention: the Prasugrel in Comparison to Clopidogrel for Inhibition of Platelet Activation and Aggregation-Thrombolysis in Myocardial Infarction 44 trial. Circulation 2007;116:2923-32.
15. Mehran R, Rao SV et al. Standardized bleeding definitions for cardiovascular clinical trials: a consensus report from the Bleeding Academic Research Consortium. Circulation 2011;123:2736-47.

16. Garcia-Garcia HM, McFadden EP et al. Standardized End Point Definitions for Coronary Intervention Trials: The Academic Research Consortium-2 Consensus Document. Circulation 2018;137:2635-2650.
17. Thygesen K, Alpert JS et al. Fourth Universal Definition of Myocardial Infarction (2018). Circulation 2018;138:e618-e651.
18. Hicks KA, Mahaffey KW et al. 2017 Cardiovascular and Stroke Endpoint Definitions for Clinical Trials. Circulation 2018;137:961-972.
19. Vranckx P, Lewalter T et al. Evaluation of the safety and efficacy of an edoxaban-based antithrombotic regimen in patients with atrial fibrillation following successful percutaneous coronary intervention (PCI) with stent placement: Rationale and design of the ENTRUST-AF PCI trial. American heart journal 2018;196:105-112.
20. Ibanez B, James S et al. 2017 ESC Guidelines for the management of acute myocardial infarction in patients presenting with ST-segment elevation: The Task Force for the management of acute myocardial infarction in patients presenting with ST-segment elevation of the European Society of Cardiology (ESC). European Heart Journal 2017;39:119-177.
21. Hindricks G, Potpara T et al. 2020 ESC Guidelines for the diagnosis and management of atrial fibrillation developed in collaboration with the European Association for Cardio-Thoracic Surgery (EACTS). Eur Heart J 2021;42:373-498.
22. Bonow RO, Carabello BA et al. ACC/AHA 2006 guidelines for the management of patients with valvular heart disease: a report of the American College of Cardiology/American Heart Association Task Force on Practice Guidelines (writing committee to revise the 1998 Guidelines for the Management of Patients With Valvular Heart Disease): developed in collaboration with the Society of Cardiovascular Anesthesiologists: endorsed by the Society for Cardiovascular Angiography and Interventions and the Society of Thoracic Surgeons. Circulation 2006;114:e84-231.
23. Campeau L. Letter: Grading of angina pectoris. Circulation 1976;54:522-3.
24. Pisters R, Lane DA, Nieuwlaat R, de Vos CB, Crijns HJ, Lip GY. A novel user-friendly score (HAS-BLED) to assess 1-year risk of major bleeding in patients with atrial fibrillation: the Euro Heart Survey. Chest 2010;138:1093-100.
25. Fontaine R, Kim M, Kieny R. [Surgical treatment of peripheral circulation disorders]. Helvetica chirurgica acta 1954;21:499-533.

# 22 Appendices

## 22.1 Appendix 1: Endpoint Definitions

**BLEEDING:**

These include all bleeding complications occurring after randomization of the patient. Bleeding localization, amount of haemoglobin drop (if available) and the number of blood units transfused must be recorded in the Case Report Form. Bleeding Academic Research Consortium Definition for Bleeding (15).

Type 0: No bleeding

Type 1: Bleeding that is not actionable and does not cause the patient to seek unscheduled performance of studies, hospitalization, or treatment by a healthcare professional; may include episodes leading to self-discontinuation of medical therapy by the patient without consulting a healthcare professional

Type 2: Any overt, actionable sign of haemorrhage (e.g., more bleeding than would be expected for a clinical circumstance, including bleeding found by imaging alone) that does not fit the criteria for type 3, 4, or 5 but does meet at least one of the following criteria: 51 (1) requiring nonsurgical, medical intervention by a healthcare professional, (2) leading to hospitalization or increased level of care, or (3) prompting evaluation

Type 3:

Type 3a: Overt bleeding plus a haemoglobin drop of 3 to <5 g/dL* (provided haemoglobin drop is related to bleed)

Any transfusion with overt bleeding

Type 3b: Overt bleeding plus a haemoglobin drop ≥5 g/dL* (provided haemoglobin drop is related to bleed)

Cardiac tamponade

Bleeding requiring surgical intervention for control (excluding dental, nasal, skin or haemorrhoid)

Bleeding requiring intravenous vasoactive agents

Type 3c: Intracranial haemorrhage (does not include microbleeds or haemorrhagic transformation, does include intraspinal)

Subcategories confirmed by autopsy or imaging or lumbar puncture

Intraocular bleed compromising vision

Type 4: CABG-related bleeding:

Perioperative intracranial bleeding within 48 h

Reoperation after closure of sternotomy for the purpose of controlling bleeding

Transfusion of ≥5 U whole blood or packed red blood cells within a 48-h period†

Chest tube output ≥2L within a 24-h period

Type 5: Fatal bleeding

Type 5a Probable fatal bleeding; no autopsy or imaging confirmation but clinically suspicious

Type 5b Definite fatal bleeding; overt bleeding or autopsy or imaging confirmation

Platelet transfusions should be recorded and reported but are not included in these definitions until further information is obtained about the relationship to outcomes.

If a CABG-related bleed is not adjudicated as at least a type 3 severity event, it will be classified as not a bleeding event.

If a bleeding event occurs with a clear temporal relationship to CABG (ie, within a 48-h time frame) but does not meet type 4 severity criteria, it will be classified as not a bleeding event.

* Corrected for transfusion (1 U packed red blood cells or 1 U whole blood =1 g/dL haemoglobin).

† Cell saver products are not counted.

**DEATH** (ARC-2 criteria) (16)

Cardiovascular death:

Is defined as death resulting from cardiovascular causes. The following categories may be collected:

- Death caused by acute MI
- Death caused by sudden cardiac, including unwitnessed, death
- Death resulting from heart failure
- Death caused by stroke
- Death caused by cardiovascular procedures
- Death resulting from cardiovascular haemorrhage
- Death resulting from other cardiovascular cause

Non-cardiovascular death:

Is defined as any death that is not thought to be the result of a cardiovascular cause. The following categories may be collected:

- Death resulting from malignancy
- Death resulting from pulmonary causes
- Death caused by infection (includes sepsis)
- Death resulting from gastrointestinal causes
- Death resulting from accident/trauma
- Death caused by other noncardiovascular organ failure
- Death resulting from other noncardiovascular cause

Undetermined death:

Is defined as a death not attributable to any other category because of the absence of any relevant source documents. Such deaths will be clarified as cardiovascular for end point determination.

**MYOCARDIAL INFARCTION**

The definition of myocardial infarction used in this trial is adapted from the Fourth Universal Definition of Myocardial Infarction (17). The term acute myocardial infarction should be used when there is acute myocardial injury with clinical evidence of acute myocardial ischaemia and with detection of a rise and/or fall of cTn values with at least one value above the 99th percentile. High-sensitivity (hs)-cTn assays are recommended as preferred biomarkers.CK-MB (and CK) values will be assessed concurrently and used in the case that troponin values are not available.

**Myocardial injury**

The term myocardial injury should be used when there is evidence of elevated cardiac troponin values (cTn) with at least 1 value above the 99$^{th}$ percentile upper reference limit (URL). The myocardial injury is considered acute if there is a rise and/or fall of cTn values.

**Myocardial Infarction Type 1:**

Detection of a rise and/or fall of cTn values with at least 1 value above the 99th percentile URL and with at least 1 of the following:

- Symptoms of acute myocardial ischaemia
- New ischaemic ECG changes

- Development of pathological Q waves on ECG
- Imaging evidence of new loss of viable myocardium or new regional wall motion abnormality in a pattern consistent with an ischaemic etiology.
- Intracoronary thrombus detected on angiography or by autopsy*

Postmortem demonstration of an atherothrombus in the artery supplying the infarcted myocardium, or a macroscopically large circumscribed area of necrosis with or without intra-myocardial haemorrhage, meets the type 1 MI criteria regardless of cTn values.

**Myocardial Infarction Type 2:**

Detection of a rise and/or fall of cTn values with at least 1 value above the 99th percentile URL, and evidence of an imbalance between myocardial oxygen supply and demand unrelated to acute coronary atherothrombosis, requiring at least 1 of the following:

- Symptoms of acute myocardial ischaemia
- New ischaemic ECG changes
- Development of pathological Q waves
- Imaging evidence of new loss of viable myocardium or new regional wall motion abnormality in a pattern consistent with an ischaemic etiology

**Myocardial Infarction Type 3:**

Patients who suffer cardiac death, with symptoms suggestive of myocardial ischaemia accompanied by presumed new ischaemic ECG changes or ventricular fibrillation, but die before blood samples for biomarkers can be obtained, or before increases in cardiac biomarkers can be identified, or MI is detected by autopsy examination.

**Myocardial Infarction Type 4a (PCI-Related MI ≤48 after the index procedure):**

Coronary intervention-related MI is arbitrarily defined by an elevation of cTn values >5 times the 99th percentile URL in patients with normal baseline values. In patients with elevated preprocedure cTn in whom the cTn level are stable (≤20% variation) or falling, the postprocedure cTn must rise by >20%. However, the absolute postprocedural value must still be at least 5 times the 99th percentile URL. In addition, 1 of the following elements is required:

- New ischaemic ECG changes
- Development of new pathological Q waves*
- Imaging evidence of new loss of viable myocardium or new regional wall motion abnormalities in a pattern consistent with an ischaemic etiology

- Angiographic findings consistent with a procedural flow-limiting complication such as coronary Dissection, occlusion of a major epicardial artery or a side branch occlusion/thrombus, disruption of collateral flow, or distal embolization†

*Isolated development of new pathological Q waves meets the type 4a MI criteria if cTn values are elevated and rising but <5 times the 99th percentile URL.

†Postmortem demonstration of a procedure-related thrombus in the culprit artery, or a macroscopically large circumscribed area of necrosis with or without intra-myocardial haemorrhage meets the type 4a MI criteria.

**Myocardial Infarction Type 4b (Stent thrombosis associated with PCI):**

Is documented by angiography or autopsy using the same criteria utilized for type 1 MI. It is important to indicate the time of the occurrence of the stent/scaffold thrombosis in relation to the timing of the PCI procedure. The following temporal categories are suggested:

- Acute 0-24 hours
- Subacute >24 hours to 30 days
- Late >30 days to 1 year
- Very late >1 year after stent/scaffold implantation

**Myocardial Infarction Type 4c (Restenosis associated with PCI):**

Defined as focal or diffuse restenosis, or a complex lesion associated with a rise and/or fall of cTn values above the 99th percentile URL applying, the same criteria utilized for type 1MI.

**Myocardial Infarction Type 5 (CABG-Related MI ≤48 after the index procedure):**

CABG-related MI is arbitrarily defined as elevation of cTn values > 10 times the 99th percentile URL in patients with normal baseline cTn values. In patients with elevated preprocedure CTn in whom cTn levels are stable (≤20% variation) or falling, the postprocedure cTn must rise by >20%. However, the absolute postprocedural value still must be > 10 times the 99th percentile URL. In addition, 1 of the following elements is required:

- Development of new pathological Q waves*
- Angiographic documented new graft occlusion or new native coronary artery occlusion
- Imaging evidence of new loss of viable myocardium or new regional wall motion abnormality in a pattern consistent with an ischaemic etiology.

*Isolated development of new pathological Q waves meets the type 5 MI criteria if cTn values are elevated and rising but <10 times the 99th percentile URL.

**Q-wave vs. Non-Q-wave Myocardial infarction:**

Q-wave MI will be diagnosed if new pathologic Q-waves (≥25% of the height of the partner R wave and/or ≥0.04 sec in duration) in ≥2 contiguous ECG leads occur. All other MIs not fulfilling the above mentioned criteria will be considered non-Q-wave MI.

## STENT THROMBOSIS

Stent thrombosis will be classified according to the Academic Research Consortium -2 (16):

**Definite Stent/Scaffold Thrombosis:**

Is considered to have occurred by either angiographic or pathological confirmation.

The angiographic confirmation includes the presence of a thrombus that originates in the stent/scaffold or in the segment 5 mm proximal or distal to the stent or in a side branch originating from the stented segment and the presence of at least 1 of the following criteria:

- Acute onset of ischemic symptoms at rest
- New ECG changes suggestive of acute ischaemia
- Typical rise and fall in cardiac biomarkers

The pathological confirmation of stent/scaffold thrombosis includes:

- Evidence of recent thrombus within the stent determined at autopsy or
- Examination of tissue retrieved following thrombectomy (visual/histology)

**Probable Stent/Scaffold Thrombosis:**

Regardless of the time after the index procedure, any myocardial infarction that is related to documented acute ischaemia in the territory of the implanted stent/scaffold without angiographic confirmation of stent thrombosis and in the absence of any other obvious cause.‡

‡ When the stented/scaffolded segment is in the left circumflex coronary artery or in the presence of preexisting electrocardiographic abnormalities (eg, left bundle branch block, paced rhythms), definitive evidence of localization may be absent and Clinical Events Committee adjudication is based on review of all available evidence.

**Silent stent occlusion:**

The incidental angiographic documentation of stent occlusion in the absence of clinical signs or symptoms is not considered stent thrombosis.

Timing of Stent thrombosis:

| | |
|---|---|
| Acute | 0§-24 h |
| Subacute | >24 h-30 d |
| Late | >30 d-1y |
| Very late | >1 y |

§ Defined as the moment the patient is undraped and taken off the catheterization table.

**STROKE**

Stroke is defined as an acute episode of focal or global neurological dysfunction of at least 24 hours caused by brain, spinal cord, or retinal vascular injury as a result of haemorrhage or infarction.

Diagnosis of stroke should be confirmed by cCT or MRI or pathology (18).

**Classification:**

Ischaemic stroke:

Ischaemic stroke is defined as an acute episode of focal cerebral, spinal, or retinal dysfunction caused by infarction of central nervous system tissue. Haemorrhage may be a consequence of ischaemic stroke. In this situation, the stroke is an ischemic stroke with haemorrhagic transformation and not a haemorrhagic stroke.

Haemorrhagic stroke:

Haemorrhagic stroke is defined as an acute episode of focal or global cerebral or spinal dysfunction caused by intraparenchymal, intraventricular, or subarachnoid haemorrhage.

Undetermined stroke:

Undetermined stroke is defined as an acute episode of focal or global neurological dysfunction caused by presumed brain, spinal cord, or retinal vascular injury as a result of haemorrhage or infarction but with insufficient information to allow categorization as either ischaemic or haemorrhagic.

**Modified Rankin Scale - Stroke severity assessment scale**

| | |
|---|---|
| Scale 0 | No symptoms at all |
| Scale 1 | No significant disability despite symptoms: able to carry out all usual duties and activities |
| Scale 2 | Slight disability: unable to carry out all previous activities but able to look after own affairs without assistance |
| Scale 3 | Moderate disability: requiring some help, but able to walk without assistance |
| Scale 4 | Moderately severe disability: unable to walk without assistance, and unable to attend to own bodily needs without assistance |
| Scale 5 | Severe disability: bedridden, incontinent and requiring constant nursing care and attention |
| Scale 6 | Death |

## SYSTEMIC THROMBOEMBOLISM

A systemic embolic event is defined as an arterial embolism resulting in clinical ischemia, excluding the central nervous system, coronary and pulmonary arterial circulation (19).

## URGENT REVASCULARIZATION

Any PCI or bypass surgery performed for recurrent ischaemia that in the investigator's opinion cannot be delayed for more than 24 hours and is defined by the investigator as a non-elective.

**Target Lesion Revascularization:**

Target lesion revascularization is defined as a repeat percutaneous intervention of the target lesion (including 5 mm proximal and distal to the stent/scaffold) or bypass surgery of the target vessel performed for restenosis or other complication of the target lesion.

**Target Vessel Revascularization:**

Target vessel revascularization is defined as any repeat percutaneous intervention or surgical bypass of any segment of the target vessel including the target lesion. The target vessel consists of target lesion(s) and any additional lesions in the main epicardial coronary artery or branches containing the target lesion.

## NYHA CLASSIFICATION OF HEART FAILURE

| | |
|---|---|
| Class I | No limitation of activities; patients suffer no symptoms from ordinary activities. |
| Class II | Slight, mild limitation of activity; patients are comfortable with rest or with mild exertion. |
| Class III | Marked limitation of activity; patients are comfortable only at rest. |
| Class IV | Patients who should be at complete rest, confined to bed or chair; any physical activity brings on discomfort and symptoms occur at rest. |

## ACUTE CORONARY SYNDROME (9,20)

### ST-segment elevation myocardial infarction (STEMI)

Is defined in patients with persistent chest discomfort or other symptoms suggestive of ischemia and persistent (>20 min) ST-segment elevation in at least two contiguous ECG leads.

### Non-ST-segment elevation myocardial infarction (NSTEMI)

Is defined in patients with acute chest discomfort or chest pain-equivalent symptoms and increased cardiac troponins above the $99^{th}$ percentile of healthy individuals (conventional or high-sensitive assays) but no persistent ST-segment elevation ECG changes.

### Unstable Angina

Is defined in patients with myocardial ischaemia at rest or on minimal exertion in the absence of acute cardiomyocyte injury/necrosis (cardiac troponins within the $99^{th}$ percentile of healthy individuals) but without persistent ST-segment elevation on ECG.

### Biomarker positive acute coronary syndrome

STEMI or NSTEMI

## 22.2 Appendix 2: Definition of Clinical and Angiographic Characteristics

### ATRIAL FIBRILLATION

Is defined as a supraventricular tachyarrhythmia with uncoordinated atrial electrical activation and consequently ineffective atrial contraction (21). Electrocardiographic characteristics of AF include:

- Irregularly irregular R-R intervals (when atrioventricular conduction is not impaired),
- Absence of distinct repeating P waves, and
- Irregular atrial activations.

**Classification of AF**

| AF pattern | Definition |
|---|---|
| First diagnosed | AF not diagnosed before, irrespective of its duration or the presence/severity of AF-related symptoms. |
| Paroxysmal | AF that terminates spontaneously or with intervention within 7 days of onset. |
| Persistent | AF that is continuously sustained beyond 7 days, including episodes terminated by cardioversion (drugs or electrical cardioversion) after ≥7 days |
| Long-standing persistent | Continuous AF of >12 months' duration when decided to adopt a rhythm control strategy |
| Permanent | AF that is accepted by the patient and physician, and no further attempts to restore/maintain sinus rhythm will be undertaken. Permanent AF represents a therapeutic attitude of the patient and physician rather than an inherent pathophysiological attribute of AF, and the term should not be used in the context of a rhythm control strategy with antiarrhythmic drug therapy or AF ablation. Should a rhythm control strategy be adopted, the arrhythmia would be re-classified as 'long-standing persistent AF'. |
| ,,valvular" AF (old term) | Means patients with moderate/severe mitral stenosis and those with mechanical prosthetic heart valves. |

## COMPLETION OF PCI

Defined by the local trial centre (including stop time as documented on the catheter laboratory report or the time point, when the patient leaves the catheter laboratory).

## MITRAL STENOSIS (22)

### CRITERIA FOR SEVERITY GRADING OF MITRAL STENOSIS

| Grading | Mitral valve area ($cm^2$) | Mean gradient at anormal heart rate (mmHg) |
|---|---|---|
| **Mild** | >1.5 cm2 | <5 |
| **Moderate** | 1.0–1.5 | 5–10 |
| **Severe** | ≤1.0 | >10 |

## SUCCESSFUL PCI

Defined as TIMI flow grade 2 or more.

## CANADIAN CARDIOVASCULAR SOCIETY (CCS) CLASSIFICATION OF ANGINA PECTORIS (23)

Class I: Ordinary physical activity (such as walking and climbing stairs) does not cause angina. Angina with strenuous or rapid or prolonged exertion at work or recreation.

Class II: Slight limitation of ordinary activity. Angina upon walking or climbing stairs rapidly, walking uphill, walking or stair climbing after meals, or in the cold, in wind or under emotional stress, or only during the few hours after awakening. Angina if walking more than two blocks on a level and climbing more than one flight of ordinary stairs at a normal pace and in normal conditions.

Class III: Marked limitation of ordinary physical activity. Walking one to two blocks on a level and climbing one flight of stairs in normal conditions and at a normal pace.

Class IV: Inability to carry on any physical activity without discomfort. Anginal syndrome may be present at rest.

## CARDIOGENIC SHOCK

Must fulfil one of the following criteria:

- Either systolic blood pressure < 90 mmHg for at least 30 minutes or
- Need for supportive measures to maintain a systolic blood pressure > 90 mmHg

Plus

- Either signs of impaired end-organ hypoperfusion (altered mental status, cold, clammy skin or extremities, oliguria with urine output <30mL/h or serum lactate >2.0 mmol/L) or
- Haemodynamic criteria of cardiac index < 2.2 L/min per square meter body surface area and a pulmonary capillary wedge pressure of at least 15 mmHg

## CHA2DS2Vasc SCORE

CHA2DS2-VASc score is used for stroke risk assessment. The maximum CHA2DS2-VASc score is 9.

| | Condition | Points |
|---|---|---|
| **C** | **Congestive heart failure** (signs/symptoms of heart failure or objective evidence of reduced left ventricular ejection fraction) | 1 |
| **H** | **Hypertension** (Resting blood pressure >140/90 mmHg on at least two occasions or current antihypertensive treatment) | 1 |
| **$A_2$** | **Age ≥75 years** | 2 |
| **D** | **Diabetes mellitus** (fasting glucose >125 mg/dL (7mmol/L) or treatment with oral hypoglycaemic agent and/or insulin) | 1 |
| **$S_2$** | **Prior stroke, transient ischaemic attack or thromboembolism** | 2 |
| **V** | **Vascular disease** (previous myocardial infarction, peripheral artery disease or aortic plaque) | 1 |
| **A** | **Age 65–74 years** | 1 |
| **Sc** | **Sex category (female)** | 1 |

**HAS-BLED SCORE**(24)

| HAS-BLED | Points |
|---|---|
| Hypertension i.e uncontrolled BP | 1 |
| Abnormal renal function | 1 |
| Abnormal liver function | 1 |
| Stroke | 1 |
| Bleeding tendency or predisposition | 1 |
| Labile INR | 1 |
| Age (e.g >65) | 1 |
| Drugs (e.g. concomitant Aspirin or NSAIDSs) | 1 |
| Alcohol abuse | 1 |

≤2 Points: low to moderate bleeding risk

>2 high bleeding risk

**CHILD-PUGH CLASS OF CHRONIC LIVER DISEASE**

The score employs five clinical measures of liver disease. Each measure is scored 1-3, with 3 indicating most severe derangement. In primary sclerosing cholangitis (PSC) and primary biliary cirrhosis (PBC), the bilirubin references are changed to reflect the fact that these diseases feature high conjugated bilirubin levels. The upper limit for 1 point is 68 µmol/l (4 mg/dl) and the upper limit for 2 points is 170 µmol/l (10 mg/dl).

| Measure | 1 point | 2 points | 3 points |
|---|---|---|---|
| Total bilirubin, µmol/l (mg/dl) | <34 (<2) | 34-50 (2-3) | >50 (>3) |
| Serum albumin, g/l | >35 | 28-35 | <28 |
| INR | <1.7 | 1.71-2.30 | > 2.30 |
| Ascites | None | Mild | Moderate to Severe |
| Hepatic encephalopathy | None | Grade I-II (or suppressed with medication) | Grade III-IV (or refractory) |

Interpretation:

Chronic liver disease is classified into Child-Pugh class A to C, employing the added score from above.

Class A: 5-6 Points

Class B: 7-9 Points

Class C: 10-15 Points

**DIABETES MELLITUS:**

Active treatment with insulin or an oral hypoglycaemic agent on admission

Patients diagnosed with diabetes who are on dietary therapy alone, documentation of an abnormal fasting blood glucose or glucose tolerance test based on the World Health Organization criteria is required.

**FAMILY HISTORY OF PREMATURE CAD**

Myocardial infarction, or sudden abrupt death without obvious cause, before the age of 55 in a first-degree blood male relative (parent, sibling, or children related by blood) or before the age of 65 in a first-degree blood female relative.

**HISTORY OF HYPERCHOLESTEROLEMIA**

Patients with any one of the following:

1. Prior total cholesterol > 200 mg/dl
2. Prior treatment with a lipid lowering agent for hypercholesterolemia

**ARTERIAL HYPERTENSION**

Arterial hypertension is considered to be present when a person's systolic blood pressure is 140 mmHg or greater, and/or their diastolic blood pressure is 90 mmHg or greater on 2 different occasions, or active treatment with antihypertensive drugs.

## MULTIVESSEL DISEASE

Presence of angiographically significant lesions (>50% lumen narrowing) in ≥ 2 major epicardial coronary arteries.

## SMOKING STATUS

| | |
|---|---|
| Smoker: | any cigarette smoking in the prior 6 months |
| Non-smoker: | no regularly cigarettes smoking at any time (according to the World Health Organization also former smoker that quit smoking for at least 10 years) |
| Former smoker: | those who had quit smoking at least 6 months before the index PCI. |

## PERIPHERAL ARTERIAL DISEASE (25)

### FONTAINE CLASSIFICATION OF PAD

| | |
|---|---|
| **Stage I** | Asymptomatic PAD |
| **Stage II** | Pain on exertion<br>• IIa: claudication at a walking distance >200 m<br>• IIb: claudication at a walking distance < 200 m |
| **Stage III** | Ischemic pain at rest |
| **Stage IV** | Necrosis, ulcers or gangrene of the limb |

## TIMI GRADE FLOW

A grading system for coronary flow:

| | |
|---|---|
| 0 | No perfusion: No ante grade flow beyond the point of occlusion. |
| 1 | Penetration without perfusion: The contrast material passes beyond the area of obstruction, but “hangs up” and fails to opacify the entire coronary bed distal to the obstruction for the duration of the cine run. |
| 2 | Partial reperfusion: The contrast material passes across the obstruction and opacifies the coronary bed distal to the obstruction. However, the rate of entry of contrast into the vessel distal to the obstruction and/or its rate of clearance from the distal bed are perceptibly slower than its entry into and/or clearance from comparable areas not |

perfused by the culprit vessel (e.g., the opposite coronary artery or coronary bed proximal to the obstruction).

3 Complete perfusion: Antegrade flow into the bed distal to the obstruction occurs as promptly as into the bed proximal to the obstruction and clearance of contrast material from the involved bed is as rapid as from an uninvolved bed in the same vessel or the opposite artery

## CHRONIC OBSTRUCTIVE PULMONARY DISEASE (COPD)

Severity of Airflow Limitation Assessment in Patients with COPD

| SEVERITY OF AIRFLOW LIMITATION | CLASSIFICATION | $FEV_1$* |
|---|---|---|
| MILD | GOLD I | ≥80% |
| MODERATE | GOLD II | 50%-79% |
| SEVERE | GOLD III | 30%-49% |
| VERY SEVERE | GOLD IV | <30% |

$FEV_1$ indicates forced expiratory volume in 1 second; FVC, forced vital capacity; GOLD, Global Initiative for Chronic Obstructive Lung Disease.

*Assessed in patients with $FEV_1$/FVC less than 0.70

## CHRONIC KIDNEY DISEASE

### GFR CATEGORIES IN CHRONIC KIDNEY DISEASE

| Category | GFR ml/min/1.73 m2 | Renal function |
|---|---|---|
| Grade 1 | ≥90 | Normal or high |
| Grade 2 | 60-89 | Mildly decreased |

| | | |
|---|---|---|
| **Grade 3a** | 45-59 | Mildly to moderately decreased |
| **Grade 3b** | 30-44 | Moderately to severely decreased |
| **Grade 4** | 15-29 | Severely decreased |
| **Grade 5** | <15 | Kidney failure |

**Transient Ischemic Neurological Attack:**

Transient ischemic attack (TIA) is defined as a transient episode of focal neurological dysfunction caused by brain, spinal cord, or retinal ischaemia, without acute infarction.

**Thrombocytopenia:**

Decrease in platelet count ≤100 x $10^9$ cells/L and a fall more than 25% from baseline and exclusion of pseudo thrombocytopenia. The severity of thrombocytopenia and the number of platelet units transfused must be recorded. Also, the date and time in which the nadir of platelet count develops must be recorded. A decrease in platelet count <20 x $10^9$ cells/L is considered profound thrombocytopenia.

Pseudo thrombocytopenia reflects erroneous reading by automated counters because of excessive platelet clumping in the presence of EDTA. A repeat platelet count should be obtained with sodium citrate as the anticoagulant, and/or a blood smear should be examined.

Heparin-induced thrombocytopenia is diagnosed in cases meeting the criteria of Pouplard and colleagues: heparin-dependent platelet-reactive antibodies in the C-serotonin release assay, or finding of increased levels of anti-heparin/PF4 antibodies by ELISA assay.

# PROTOCOL

## Escalated single Platelet Inhibition for one month plus Direct oral anticoagulation in patients with Atrial fibrillation and acUte coRonary syndrome undergoing percutaneoUS coronary intervention

## EPIDAURUS

| | |
|---|---|
| **Protocol Code** | EPIDAURUS-2020 |
| **EudraCT No.** | EudraCT 2020-004748-27 |
| **EU-CT No.** | 2024-512727-36-00 |
| **Sponsor / Sponsor Delegated Person:** | LMU University Hospital Munich<br>Univ.-Prof. Dr. med. Steffen Massberg<br>Department of Cardiology<br>Marchioninistr. 15, 81377 Munich, Germany |
| **Coordinating Investigator** | Prof. Dr. med. Konstantinos Rizas<br>LMU University Hospital Munich<br>Department of Cardiology<br>Marchioninistr. 15, 81377 Munich, Germany |
| **Principal Investigators** | Prof. Dr. med. Konstantinos Rizas,<br>Univ.-Prof. Dr.rer. nat. Ulrich Mansmann<br>Univ.-Prof. Dr. med. Steffen Massberg |
| **Version number / Date** | Version 8.0 EU (05-Jan-2025) |
| **Clinical Trial Phase:** | IV |

## Signatures:

# Declaration of the Sponsor

The present clinical trial protocol was subject to critical review. Its content is consistent with the current risk/benefit evaluation of the investigational medicinal product (IMP) as well as with moral, ethical and scientific principles of good clinical practice (GCP), the latest version of the Declaration of Helsinki, the local laws and regulations as well as applicable regulatory requirements.
With the signature below the person confirms to have read this clinical trial protocol and to agree that it contains all information required for study performance. Furthermore, the person agrees to conduct the study as set in this clinical trial protocol and to adhere to the sponsor's standard operation procedure (SOPs), if provided and as far as agreed. It has been understood that all documentation previously not published will be kept confidential. Furthermore the person agrees to take all necessary measures to ensure safety and confidentiality of the patient´s identities.

| | |
|---|---|
| Univ.-Prof. Dr. med. Steffen Massberg<br>LMU University Hospital Munich<br>Department of Cardiology<br>Author and Sponsor Delegated Person | 05 Feb, 2025<br>Place, Date Signature |

# Declaration of the Author, the Coordinating Investigator and the Statistician

The present clinical trial protocol was subject to critical review. Its content is consistent with the current risk/benefit evaluation of the IMP as well as with moral, ethical and scientific principles of GCP, the latest version of the Declaration of Helsinki, the local laws and regulations as well as applicable regulatory requirements.
With their signature below the persons confirm to have read this clinical trial protocol and to agree that it contains all information required for study performance. Furthermore, the persons agree to conduct the study as set in this clinical trial protocol and to adhere to the sponsor's SOPs (as far as agreed and provided). It has been understood that all documentation previously not published will be kept confidential. Furthermore the persons agree to keep patient´s identities in strictest confidence and to take all required measures to ensure this confidentiality.

| | |
|---|---|
| Prof. Dr. med. Konstantinos Rizas<br>LMU University Hospital Munich<br>Department of Cardiology<br>Author & Coordinating Investigator | München, 6-Feb 2025<br>Place, Date, Signature |
| Prof. Dr. rer. nat. Ulrich Mansmann<br>Head of Institute for Medical Information Processing, Biometry, and Epidemiology,<br>LMU Munich<br>Statistician | LUDWIG-MAXIMILIANS-UNIVERSITÄT<br>Prof. Dr. U. Mansmann<br>IBE - Institut für Medizinische Informations-verarbeitung, Biometrie und Epidemiologie<br>Marchioninistr. 15, 81377 München<br>13.03.2025<br>Place, Date, Signature |

# Declaration of the Coordinating Investigator

The present clinical trial protocol was subject to critical review. Its content is consistent with the current risk/benefit evaluation of the IMP as well as with moral, ethical and scientific principles of GCP, the latest version of the Declaration of Helsinki, the local laws and regulations as well as applicable regulatory requirements.
With their signature below the persons confirm to have read this clinical trial protocol and to agree that it contains all information required for study performance. Furthermore, the persons agree to conduct the study as set in this clinical trial protocol and to adhere to the sponsor's SOPs (as far as agreed and provided). It has been understood that all documentation previously not published will be kept confidential. Furthermore the persons agree to keep patient´s identities in strictest confidence and to take all required measures to ensure this confidentiality.

| | |
|---|---|
| Name and Institution of the Coordinating Investigator | Prof. Dr. med. Konstantinos Rizas<br>Leitung Campus Innenstadt<br>LMU Klinikum<br>Medizinische Klinik und Poliklinik I<br>Ziemssenstraße 5, 80336 München<br>5-Feb-2025<br>Place, Date, Signature |

# Investigator Agreement Page

By my signature below, I hereby attest that I have read, discussed and understood the background information concerning the investigational product. I have read and discussed this protocol and agree to carry out the trial as set out therein. I agree that the trial shall be carried out according to the current ICH E6 standard and all applicable regulatory requirements. I accept my obligations relating to the principles that have their origin in the Declaration of Helsinki (2013) and specifically to Independent Ethics Committee (IEC), Informed Consent, and also my obligations to LMU University Hospital Munich, or contracted representatives, as far as safety reporting, providing data, allowing monitoring, auditing, inspection by domestic and foreign Regulatory Authorities and quality control visits are concerned.

Institution: ______________________________________________

Signatures

Principal Investigator: ______________________________

Last name, first name, function / title

______________________ ______________________________

Date [DD-Mmm-YYYY] Signature

Deputy Principal Investigator: ______________________________

(if applicable)

Last name, first name, function / title

______________________ ______________________________

Date [DD-Mmm-YYYY] Signature

# Table of Contents


1 Protocol Synopsis ........ 9
2 Schedule of Activities ........ 15
3 List of Abbreviations ........ 16
4 Administrative Structure ........ 19
5 Introduction and Background ........ 21
5.1 Background and Purpose ........ 21
5.1.1 Background ........ 21
5.1.2 Purpose ........ 25
5.2 Dose Rationale ........ 25
5.3 Overall Risk/ Benefit Assessment ........ 26
6 Trial Objectives and Endpoints ........ 27
6.1 Primary Endpoint(s) ........ 27
6.2 Secondary Endpoints ........ 27
6.3 Additional Event Evaluation ........ 27
7 Clinical Trial Protocol ........ 28
7.1 Trial Design ........ 28
7.2 Trial Duration ........ 28
8 Trial Population ........ 29
8.1 Number of patients ........ 29
8.2 Inclusion Criteria ........ 29
8.3 Exclusion Criteria ........ 29
8.4 Patient Recruitment and Screening ........ 30
9 Treatment ........ 30
9.1 Trial Treatment ........ 30
9.2 Treatment Arms ........ 33
9.2.1 Experimental intervention ........ 33
9.2.2 Control intervention ........ 33
9.3 Treatment Assignment and Randomization ........ 33
9.4 Packaging, Labelling and Supply ........ 34
9.5 Treatment of Patients ........ 34
9.5.1 Patient Numbering ........ 34
9.5.2 Treatment Administration ........ 34
9.5.3 Compliance ........ 34
9.5.4 Prior and Concomitant Medication ........ 34
9.6 Trial Completion and Discontinuation ........ 35
9.6.1 Trial Completion and Post-Trial Treatment ........ 35

9.6.2 Lost to Follow-up ....... 35
9.6.3 Discontinuation of Patients from Treatment or Trial Participation ....... 35
9.6.4 Conditions for Terminating the Trial by the Sponsor ....... 36
10 Trial Procedures ....... 36
10.1 Screening / Baseline Visit ....... 36
10.2 Visit at Discharge ....... 38
10.3 Control Visit (Day 42 ± 7) ....... 38
10.4 Final Visit (Day 180 ± 10) ....... 38
11 Safety Monitoring ....... 39
11.1 Definitions ....... 39
11.1.1 Adverse Event (AE) ....... 39
11.1.2 Adverse Reaction (AR) ....... 39
11.1.3 Serious Adverse Event (SAE) ....... 39
11.1.4 Suspected Unexpected Serious Adverse Reaction (SUSAR) ....... 39
11.2 Assessment Criteria ....... 40
11.2.1 Adverse Event Assessment by the Investigator ....... 40
11.2.2 Assessment by the Sponsor ....... 40
11.3 Collection and Reporting of (Serious) Adverse Events ....... 41
11.3.1 Adverse Events ....... 41
11.3.2 Serious Adverse Events ....... 42
11.4 Expedited Reporting ....... 43
11.5 Reporting of Trial Treatment Errors including Misuse / Abuse ....... 43
11.6 Pregnancy Reporting ....... 43
11.7 Annual Safety Report ....... 44
12 Statistics ....... 44
12.1 Sample Size Calculation ....... 44
12.2 Definition of Population Included in the Analysis ....... 45
12.3 Statistical Methods ....... 46
12.4 Pre-specified subgroup analysis ....... 46
12.5 Handling of Protocol Deviations and Missing Data ....... 47
13 Substudy ....... 47
14 Quality Assurance and Control ....... 48
14.1 Routine Monitoring ....... 48
14.2 Compliance with Protocol and Protocol Revisions ....... 48
14.3 Measures of Quality Assurance and Quality Control ....... 49
14.3.1 Quality Control ....... 49
14.3.2 Quality Assurance ....... 49
15 Committees ....... 49
15.1 Steering Committee ....... 49
15.2 Data Safety Monitoring Board ....... 49

15.3 Event Adjudication Committee ....... 50
16 Data Handling and Record Keeping ....... 50
16.1 Case Report Forms ....... 50
16.2 Source Data ....... 50
16.3 Data Management ....... 51
16.3.1 Data Handling and Quality Control ....... 51
16.3.2 Data Coding ....... 51
16.3.3 Database Lock ....... 52
16.4 Data Protection and Confidentiality ....... 52
16.5 Archiving and Data Retention ....... 52
16.6 Access to Source Data/Documents / Audits and Inspections ....... 52
17 Ethical, Regulatory and Administrative Aspects ....... 53
17.1 Good Clinical Practice ....... 53
17.2 Ethics Commission and Regulatory Authorities ....... 53
17.3 Informed Consent ....... 53
17.4 Further Reporting Obligations ....... 53
17.4.1 Serious Breaches ....... 53
17.4.2 Other Reporting Obligations relevant for Subject Safety ....... 54
17.4.3 Urgent Safety Measures ....... 54
17.4.4 Start, End, Temporary Halt, and Early Termination of a Clinical Trial ....... 54
18 Financing ....... 54
19 Insurance ....... 54
20 Submission of the Results and Publication Policy ....... 55
21 References ....... 56
22 Appendices ....... 58

# 1 Protocol Synopsis

| | |
|---|---|
| Title of Study: | **E**scalated single **P**latelet **I**nhibition for one month plus **D**irect oral anticoagulation in patients with **A**trial fibrillation and ac**U**te co**R**onary syndrome undergoing percutaneo**US** coronary intervention |
| Short Title: | EPIDAURUS |
| Protocol Code: | EPIDAURUS-2020 |
| Sponsor / Sponsor Delegated Person: | LMU University Hospital Munich<br>Univ.-Prof. Dr. med. Steffen Massberg<br>Department of Cardiology<br>Marchioninistr. 15, 81377 Munich, Germany |
| Coordinating Investigator: | Prof. Dr. med. Konstantinos Rizas<br>LMU University Hospital Munich<br>Department of Cardiology<br>Marchioninistr. 15, 81377 Munich, Germany |
| Principal Investigators | Prof. Dr. med. Konstantinos Rizas,<br>LMU University Hospital Munich, Department of Cardiology<br>Marchioninistr. 15, 81377 Munich, Germany<br><br>Univ.-Prof. Dr.rer. nat. Ulrich Mansmann,<br>Institute for Medical Information Processing, Biometry, and Epidemiology, Marchioninistr. 15, 81377 Munich, Germany<br><br>Univ.-Prof. Dr. med. Steffen Massberg<br>LMU University Hospital Munich, Department of Cardiology<br>Marchioninistr. 15, 81377 Munich, Germany |
| Trial Type: | Prospective, randomized, open-label, multinational European, multicentre, phase IV clinical trial |
| Design: | <u>Experimental intervention:</u><br>Escalated regimen consisting of<br>Weeks 1 – 4 : Potent $P2Y_{12}$-inhibitor + non-vitamin K antagonist oral anticoagulant<br>Week 5 – month 6: Clopidogrel + non-vitamin K antagonist oral anticoagulant<br><u>Control intervention:</u><br>Standard regimen consisting of |

| | |
|---|---|
| | In-hospital: Aspirin + Clopidogrel + non-vitamin K antagonist oral anticoagulant (up to 1 week)<br>Hospital-discharge or day 8 – month 6: Clopidogrel + non-vitamin K antagonist oral anticoagulant. In patients with high ischaemic risk the treating physician is allowed to prescribe Aspirin for a period of up to 4 weeks, according to current guidelines. |
| Hypothesis / Objectives: | To test whether an escalated antiplatelet therapy with a potent $P2Y_{12}$-inhibitor for one month can reduce ischaemic events without a significant increase in bleeding complications in patients with atrial fibrillation and ST elevation myocardial infarction (STEMI) or non-ST-segment elevation myocardial infarction (NSTEMI) undergoing successful completion of percutaneous coronary intervention. |
| Inclusion Criteria: | • Signed written informed consent<br>• Age ≥ 18 years<br>• Atrial fibrillation requiring oral anticoagulation<br>• STEMI or NSTEMI (biomarker positive acute coronary syndrome)<br>• Successful completion of PCI (defined as TIMI flow grade 2 or more; randomization will take place within 5 days (recommended within 24h) after successful PCI and before hospital discharge) |
| Exclusion Criteria: | • Chronic renal insufficiency with glomerular filtration rate < 15 ml/min/1.73m$^2$<br>• History of ischaemic stroke or transient ischaemic attack (both contraindications for Prasugrel) and history of intracranial bleeding (contraindication for Ticagrelor)<br>• Contraindication for Clopidogrel or Aspirin<br>• Contraindication for Prasugrel and Ticagrelor<br>• Severe chronic liver disease (Child-Pugh C)<br>• Indication for oral anticoagulation with Vitamin K antagonists<br>• Moderate to severe mitral stenosis or mechanical heart valve<br>• Any bleeding BARC type ≥ 2 within the last 4 weeks before index procedure<br>• Pregnancy or lactation |

| | |
|---|---|
| | • Inability to cooperate with the protocol requirements<br>• Life expectancy < 6 months<br>• Participation in another investigational drug study<br>• Previous enrolment in this study<br>• For women of childbearing potential no negative pregnancy test and no agree to use a reliable method of birth control during the study<br>• Previous treatment with GP IIb/IIIa inhibitors within the last 12 hours<br>• A known genetic disorder involved in the metabolism of the study medication<br>• Any other reason in the opinion of the investigator making the patient ineligible for participation in the trial |
| Primary Endpoints: | Primary efficacy endpoint:<br>Major ischaemic events defined as the composite of all-cause mortality, myocardial infarction, definite or probable stent thrombosis, ischaemic stroke, systemic thromboembolism, or urgent revascularization (superiority test) at 6 weeks after randomization<br>Primary safety endpoint:<br>All cause mortality, bleeding type 2 or higher according to the Bleeding Academic Research Consortium (BARC) criteria (non-inferiority test) at 6 weeks after randomization<br>Both primary endpoints will be assessed using the Finkelstein and Schoenfeld test (win/loss ratio) using a decreasing ranking for the above-mentioned components of the endpoints. |
| Secondary Endpoints: | Secondary endpoints:<br>• All individual components of the primary endpoint (all-cause mortality, myocardial infarction, definite or probable stent thrombosis, ischaemic stroke, systemic thromboembolism) at 6 weeks after randomization<br>• Cardiovascular mortality at 6 weeks after randomization<br>• Bleeding (BARC type ≥ 2) at 6 weeks after randomization<br>• Urgent revascularization at 6 months after randomization<br>• All-cause mortality at 6 months after randomization |

| | |
|---|---|
| | • Unplanned hospitalization due to acute heart failure or acute coronary syndrome at 6 months after randomization<br>• Ischaemic stroke at 6 months after randomization |
| Randomization: | A randomized parallel group design to control for confounding is used. Patients will be randomized within 5 days (recommended within 24h) after successful completion of PCI (defined as TIMI flow grade 2 or more; transradial approach preferred) and before hospital discharge. The randomization will be 1:1 for each treatment arm with stratification by centre. The block size is chosen as 8. |
| Study Visits: | Screening/Baseline Visit:<br>- Informed Consent<br>- Assessment of in- and exclusion criteria<br>- Demographic data, general and cardiovascular medical history, current diseases<br>- Clinical parameters<br>- Laboratory parameters<br>- Concomitant medication<br>- Randomization<br>Discharge Visit:<br>- Laboratory parameters: CK max, troponin max<br>- Assessment of adverse events and occurrence of endpoints<br>- Concomitant medication<br>Control Visit (Day 42 ± 7):<br>- Assessment of adverse events and occurrence of endpoints<br>- Assessment of symptoms: angina pectoris (CCS classification), dyspnoea (NYHA classification), syncope<br>- Concomitant medication<br>- Laboratory: Laboratory parameters that were assessed after discharge of index hospital stay (if applicable)<br>- Applicable for Austria only: after randomization monthly pregnancy tests for women of childbearing potential<br>Final Visit (Day 180 ± 10): |

| | |
|---|---|
| | - Assessment of adverse events and occurrence of endpoints<br>- Concomitant medication<br>- Smoking status<br>- Applicable for Austria only: after randomization monthly pregnancy tests for women of childbearing potential |
| Sample Size / Number of patients / Planned No. of Sites: | To be assessed for eligibility (n = 2000)<br><br>To be allocated to trial (n = 1474)<br><br>To be analysed (n = 1390)<br><br>Assumptions for sample-size calculation (hierarchical testing)<br><br>• Efficacy endpoint (ischaemic events) at 6 weeks:<br><br>Superiority testing, using the Finkelstein and Schoenfeld test (win-loss-ratio; WLR) and application of decreasing ranking for the components of the ischaemic endpoint: (i) death; (ii) definite or probable stent thrombosis; (iii) myocardial infarction; (iv) stroke; (v) systemic embolism; (vi) urgent revascularization. The hazard rates in the control group were calculated on the basis of the analysis of the 223 control-patients included in EPIDAURUS until November 4th, 2024. The assumed effect of intervention (cumulative odds-ratio 0.67), which was based on previous studies, remained unchanged. For the calculations we used a simulation approach with 10^6 iterations.<br><br>Null hypothesis H0: WLR =1. For a two-sided alpha of 0.05, 6-week hazard-rates and SE of hazard rates according to the values provided above the power is 80.0% if comparison of WLR is performed on 615 patients per group. To account for dependency between the different endpoints the sample size was increased by 10% leading to a sample size of **677** patients per group. The computation assumes an allocation ratio ($n_{control}/n_{interventional}$) = 1.0.<br><br>• Safety endpoint: Non-inferiority testing for mortality, bleeding BARC 3c, BARC 3b, BARC 3a and BARC ≥ 2) at 6 weeks tested using the Finkelstein and Schoenfeld test (WLR) and application of decreasing ranking for the components of the safety endpoint.<br><br>When the sample size in each group is **695**, a two-group large-sample normal approximation test of proportions with a one-sided 2.5% significance level will have 80% power to reject the null hypothesis that the lower 97.5% confidence interval of the WLR compared between the intervention and control arm is |

| | |
|---|---|
| | lower than 0.77 (1/(1 + delta), with delta = 0.3) in favour of the alternative hypothesis that the lower 97.5% confidence interval is greater than or equal to 0.77. A dependency between the endpoints was not considered, as the endpoints of the safety analysis are mutually exclusive.The hazard rates ın the control group were calculated on the basis of the analysis of the 223 control-patients included in EPIDAURUS until November 4th, 2024. The assumed effect of intervention, which was based on previous studies, remained unchanged. For the calculations we used a simulation approach with 10^6 iterations.<br><br>The sample size of the trial is given by the maximum of both sample sizes: **695** per group.<br><br>Assuming an allocation ratio $n_{control}/n_{interventional}$ = 1.0 and a 6% drop-out rate, based on an analysis of available data, the sample study of the study was calculated to be **1474**. |
| Data Analysis: | The efficacy composite endpoint includes (i) death, (ii) definite or probable stent thrombosis, (iii) myocardial infarction, (iv) stroke, (v) systemic embolism, and (vi) urgent revascularization. The safety composite endpoint includes (i) death, (ii) BARC 3c bleeding, (iii) BARC 3b bleeding, (iv) BARC 3a bleeding, and (v) BARC 2 bleeding. The endpoints will be tested using a hierarchical design with superiority testing of the first endpoint and non-inferiority testing of the second endpoint, if superiority of the first endpoint is met. Both composite endpoints will be analyzed using Finkelstein and Schoenfeld test (win-loss-ratio; [WLR] using a decreasing ranking.<br><br>We will calculate the WLR using an unmatched approach with application of bootstrapping (boot.n = 1.000 iterations) for calculation of SE and 95% confidence intervals. The results will be presented using forrest plots. We will also use Kaplan-Maier estimates to visualize time-to specific events (ischaemic events and bleeding events). A similar approach will be used for secondary endpoints. |
| Duration of the Trial: | Time for preparation of the trial (months): 6<br><br>First patient in to last patient out (months): 78<br><br>Duration of the entire trial (months): 84<br><br>Recruitment period (months): 72<br><br>Trial duration per patient (months): 6<br><br>End of Trial: Last patient last visit |
| Follow-up: | Control visit: Day 42 (± 7 days)<br><br>Final visit: Day 180 (± 10 days) |

## 2 Schedule of Activities

| | Screening/ Baseline visit Day 0 | Discharge visit | Control visit Day 42 ± 7 | Final visit Day 180 ± 10 |
|---|---|---|---|---|
| Informed consent | X | | | |
| Inclusion- and exclusion criteria | X | | | |
| Demographic data | X | | | |
| Patient history | X | | | |
| Clinical parameters | X | | | |
| Laboratory[1] | X | | X[2] | |
| Concomitant medication | X | X | X | X |
| Randomization | X | | | |
| AEs und SAEs | X | X | X | X |
| Primary efficacy endpoint (all-cause mortality, MI, ST, ischaemic stroke, systemic thromboembolism) | | X | X | |
| Primary safety endpoint (BARC bleeding ≥ 2) | | X | X | |
| Cardiovascular mortality | | X | X | |
| Myocardial Infarction | | X | X | |
| Ischaemic stroke | | X | X | X |
| Stent thrombosis | | X | X | |
| Systemic thromboembolism | | X | X | |
| Urgent revascularization | | X | X | |
| BARC bleeding ≥ 1 | | X | X | |
| All-cause mortality | | X | X | X |
| Unplanned hospitalization due to acute heart failure or acute coronary syndrome | | | X | X |
| Applicable for Austria only: after randomization monthly pregnancy tests for women of childbearing potential | | | X | X |

[1] Beyond clinical routine, platelet function testing will be performed. [2] Not obligatory

# 3 List of Abbreviations

| Term | Definition |
|---|---|
| ACS | Acute Coronary Syndrome |
| AE | Adverse Event |
| ACEI | Angiotensin-Converting-Enzyme Inhibitor |
| AF | Atrial Fibrillation |
| ATC | Anatomical Therapeutical Chemical |
| AR | Adverse Reaction |
| ARB | Angiotensin II Receptor Blocker |
| ARC | Academic Research Consortium |
| ARNI | Angiotensin-Receptor-Neprilysin-Inhibitor |
| BARC | Bleeding Academic Research Consortium |
| BNP | Brain Natriuretic Peptide |
| BUN | Blood Urea Nitrogen |
| CA | Competent Authority |
| CABG | Coronary Artery Bypass Grafting |
| CAPA | Corrective and Preventive Action |
| CAD | Coronary Artery Disease |
| CCB | Calcium Channel Blocker |
| CCS | Canadian Cardiovascular Society |
| cCT | Cerebral Computed Tomography |
| CI | Confidence Interval |
| CK | Creatine Kinase |
| CRF | Case Report Form |
| CRF | Chronic Renal Failure |
| CRO | Clinical Research Organization |
| CRP | C-reactive Protein |
| CRT | Cardiac Resynchronization Therapy |
| CTIS | Clinical Trial Information System |
| cTn | Cardiac Troponin |
| GOLD | Global Initiative for Chronic Obstructive Lung Disease |
| COPD | Chronic Obstructive Pulmonary Disease |
| CVD | Cerebrovascular Disease |
| DAPT | Dual Antiplatelet Therapy |
| DAT | Dual Antithrombotic Therapy |
| DC | Deceleration Capacity |
| DMP | Data Management Plan |
| DSMB | Data Safety Monitoring Board |
| DVP | Data Validation Plan |
| EDTA | (Ethane-1,2-diyldinitrilo)Tetraacetic Acid |
| ELISA | Enzyme-linked Immunosorbent Assay |
| GP IIb/IIIa | Glycoprotein IIb/IIIa |

| | |
|---|---|
| HbA1c | Haemoglobin A1c |
| HDL | High-density Lipoprotein |
| HR | Hazard Ratio |
| EAC | Event Adjudication Committee |
| EC | Ethics Committee |
| ECG | Electrocardiogram |
| EDC | Electronic Data Capture |
| eCRF | Electronic Case Report Form |
| ESC | European Society of Cardiology |
| FPFV | First patient First Visit |
| GCP | Good Clinical Practice |
| GOT | Glutamate-Oxaloacetate-Transaminase |
| GPT | Glutamate-Pyruvate-Transaminase |
| ICD | Implantable Cardioverter Defibrillator |
| ICH | International Conference on Harmonisation |
| ID | Identifier |
| IEC | Independent Ethics Committee |
| INR | International Normalized Ratio |
| IMP | Investigational Medicinal Product |
| ISTH | International Society on Thrombosis and Haemostasis |
| ITT | Intention to treat |
| LCA | Left Coronary Artery |
| LDH | Lactate Dehydrogenase |
| LDL | Low-density Lipoprotein |
| LVEF | Left Ventricular Ejection Fraction |
| MACE | Major Adverse Cardiac Event |
| MI | Myocardial Infarction |
| MRI | Magnetic Resonance Imaging |
| NOAC | New Oral Anticoagulant |
| NSAID | Non-Steroidal Anti-Inflammatory Drug |
| NSTEMI | Non-ST-Elevation Myocardial Infarction |
| NT-proBNP | N-terminal pro Brain Natriuretic Peptide |
| NYHA | New York Heart Association |
| OAC | Oral Anticoagulation |
| OR | Odds Ratio |
| PAOD | Peripheral Arterial Occlusive Disease |
| PBC | Primary Biliary Cirrhosis |
| PCI | Percutaneous Coronary Intervention |
| PFT | Platelet Function Test |
| PRD | Periodic Repolarization Dynamics |
| PSC | Primary Sclerosing Cholangitis |
| SAE | Serious Adverse Event |
| SAP | Statistical Analysis Plan |

| | |
|---|---|
| SD | Standard Deviation |
| SLGT2 | Sodium dependent Glucose co-transporter 2 |
| SmPC | Summary of Product Characteristics |
| ST | Stent Thrombosis |
| STEMI | ST-Elevation Myocardial Infarction |
| SUSAR | Suspected Unexpected Serious Adverse Reaction |
| SOP | Standard Operation Procedure |
| TAT | Triple Antithrombotic Therapy |
| TIMI | Thrombolysis In Myocardial Infarction |
| TLR | Target Lesion Revascularization |
| TMF | Trial Master File |
| TVR | Target Vessel Revascularization |
| URL | Upper Reference Limit |
| VKA | Vitamin K antagonist |

# 4 Administrative Structure

| | |
|---|---|
| Sponsor /<br>Sponsor Delegated Person | LMU University Hospital Munich<br>Univ.-Prof. Dr. med Steffen Massberg<br>Department of Cardiology<br>Marchioninistr. 15, 81377 Munich, Germany |
| Coordinating Investigator | Prof. Dr. med. Konstantinos Rizas<br>LMU University Hospital Munich<br>Department of Cardiology<br>Marchioninistr. 15, 81377 Munich, Germany |
| Steering Committee | Univ.-Prof. Dr. med. Steffen Massberg (Chair, LMU University Hospital Munich)<br>Prof. Dr. med. Konstantinos Rizas (LMU University Hospital Munich)<br>Prof. Dr. med. Ingo Eitel (University Heart Center Lübeck)<br>PD Dr. med. Thomas Stiermaier (University Heart Center Lübeck)<br>Prof. Dr. Adnan Kastrati (Deutsches Herzzentrum München)<br>Prof. Dr. med. Dirk Sibbing (Privatklinik Lauterbacher Mühle am Ostersee)<br>Prof. Dr. med. Paulus Kirchhof (Universitäres Herz- und Gefäßzentrum UKE Hamburg)<br>Prof. Dr. med. Christoph Stellbrink (Klinikum Bielefeld)<br>Univ.-Prof. Dr. med. Holger Thiele (Herzzentrum Leipzig)<br>Univ.-Prof. Dr. med. Ulf Landmesser (Campus Benjamin Franklin, Charité – Universitätsmedizin Berlin)<br>PD Dr. med. Marc Kollum (Hegau-Bodensee-Klinikum Singen) |
| Sources of financial support | Daiichi-Sankyo Deutschland GmbH |
| Coordinating Research Center | ISAResearch Center<br>Deutsches Herzzentrum München des Freistaates Bayern<br>Klinik an der Technischen Universität München<br>Lazarettstr. 36, 80636 Munich, Germany |

| | |
|---|---|
| Monitoring | ISAResearch Center<br>Deutsches Herzzentrum München des Freistaates Bayern<br>Klinik an der Technischen Universität München<br>Lazarettstr. 36, 80636 Munich, Germany |
| Biometry / Statistics | Prof. Dr. rer. nat. Ulrich Mansmann<br>Institute for Medical Information Processing, Biometry, and Epidemiology, LMU Munich<br>Marchioninistr. 15, 81377 Munich, Germany |
| Data Management | ISAResearch Center<br>Deutsches Herzzentrum München des Freistaates Bayern<br>Klinik an der Technischen Universität München<br>Lazarettstr. 36, 80636 Munich, Germany |
| Pharmacovigilance/ Safety Management | ISAResearch Center<br>Deutsches Herzzentrum München des Freistaates Bayern<br>Klinik an der Technischen Universität München<br>Lazarettstr. 36, 80636 Munich, Germany |

The participating sites with contact details are to be found in a list maintained by Project Management.

# 5 Introduction and Background

## 5.1 Background and Purpose

### 5.1.1 Background

The selection of the optimal antithrombotic therapy in patients with nonvalvular atrial fibrillation (AF) and acute coronary syndrome (ACS) undergoing percutaneous coronary intervention (PCI) is challenging. Dual antiplatelet therapy (DAPT) with Aspirin and a $P2Y_{12}$-inhibitor has been proven to reduce recurrent cardiovascular events in patients undergoing PCI, but is less effective in reducing the incidence of ischaemic stroke related to AF (1). On the other hand, oral anticoagulants (OAC) are the standard therapy to prevent stroke and systemic embolism in patients suffering from AF, but is not indicated as secondary prevention after PCI (2). Until recently, triple antithrombotic therapy (TAT) consisting of Aspirin plus Clopidogrel plus OAC was considered the treatment of choice. While efficiently preventing ischaemic events, TAT is associated with an increase in bleeding complications. Therefore, in the past years several randomized controlled trials (PIONEER AF-PCI, RE-DUAL PCI, ENTRUST-AF PCI, and AUGUSTUS) challenged TAT by comparing a TAT-regimen based on Vitamin K antagonists (VKA) to a dual antithrombotic therapy (DAT)-regimen based on new oral anticoagulants (NOACs) and $P2Y_{12}$-inhibitors, mainly Clopidogrel in patients with AF undergoing PCI (3-6).

In PIONEER AF-PCI a total of 2,124 patients with AF undergoing PCI were randomized in a 1:1:1 ratio to a standard TAT regimen with VKA and DAPT (control group) vs. a DAT group with low-dose Rivaroxaban (15mg) and a $P2Y_{12}$-inhibitor for 12 months (first intervention group), and finally a TAT regimen with very-low dose Rivaroxaban (2.5mg twice daily) plus DAPT, followed by Rivaroxaban 15mg at the time of $P2Y_{12}$-inhibitor discontinuation (second intervention group) (6). In this trial, 30% of the patients presented with biomarker positive myocardial infarction (MI). Ninety-four percent of the patients were treated with Clopidogrel and only 6% of the patients were on treatment with a potent $P2Y_{12}$-inhibitor (Prasugrel or Ticagrelor). Compared to standard TAT with VKA, Rivaroxaban-based regimens were associated with a significant reduction in adverse bleeding events without a significant increase in major adverse cardiovascular events. However, the power for the exploratory analysis of ischaemic events was estimated to be 11.4% (7).

RE-DUAL PCI randomized 2,725 patients with AF undergoing PCI (54% presented with ACS) to two regimens with Dabigatran (110mg and 150mg) and a $P2Y_{12}$-inhibitor (Clopidogrel in 88%, Ticagrelor in 12%) vs. a standard TAT regimen with VKA, Clopidogrel and Aspirin for 1-3 months (5). During a mean follow-up period of 14 months there was a significant reduction in major and clinically relevant non-major bleeding events in the groups treated with Dabigatran compared to the control group, without a significant increase in thromboembolic events. There was a numeric increase of ischaemic events in patients receiving DAT compared to TAT. However, regarding ischaemic events the trial was only powered for a non-inferiority analysis with a non-inferiority margin of 1.38 (5).

ENTRUST-AF PCI was a phase 3b trial, which randomized 1,506 patients with AF undergoing PCI to a DAT-regimen based on Edoxaban and a TAT-regimen with VKA, $P2Y_{12}$-inhibitor and Aspirin (3). Among the included patients, 52% presented with an ACS and only 7% were treated with a potent $P2Y_{12}$-inhibitor. The NOAC-based DAT regimen was associated with a non-significant reduction in bleeding complications. In an exploratory analysis, which however was not sufficiently powered, there was no significant increase in ischaemic complications between the two treatment groups, even though myocardial infarction and stent thromboses (ST) were seen more frequently in DAT compared to TAT.

In the recently published AUGUSTUS trial, 4,614 patients with coronary artery disease (CAD) and AF were randomized in a two-by-two factorial design to Apixaban vs. VKA and to Aspirin (TAT regimen) vs. placebo (DAT regimen) for 6 months (4) Only 37% of the patients presented with an ACS treated with PCI. About 24% of the patients presented with an ACS, which was treated medically. Only 331 (7%) patients were treated with a potent $P2Y_{12}$-inhibitor. At 6 months, the primary outcome of major or clinically relevant non-major bleeding events was significantly lower in patients treated with Apixaban compared to patients treated with VKA and was 89% higher in patients on a TAT regimen compared to patients on a DAT regimen. In a post-hoc analysis, which only included patients treated with PCI, there was a 42%-decrease in the rate of stent thromboses in patients treated with a TAT regimen compared to DAT (HR 0.58, 95% CI 0.28–1.22) (8) which however, did not reach the level of statistical significance. Eighty percent of all stent thromboses occurred within the first month after the index PCI (9).

The study design, baseline characteristics and endpoints of the PIONEER AF-PCI, RE-DUAL PCI, ENTRUST-AF PCI, and AUGUSTUS trials are summarized in Table 1. Based on the results of the above-mentioned randomized trials, which consistently showed a significant decrease in bleeding complications without detecting a significant increase in ischaemic events, current guidelines were revised and now recommend an in-hospital TAT followed by DAT based on NOAC in patients with an indication for oral anticoagulation undergoing PCI (2,10) However, all of the above trials were powered to show a superiority of a NOAC-based DAT to the VKA-based TAT with regard to bleeding events. In contrast, all analyses regarding ischaemic events were exploratory and not sufficiently powered. Moreover, the patients at high-risk of ischaemic complications with biomarker positive ACS undergoing PCI were underrepresented in all studies. In a recent meta-analysis of all four trials Gargiulo et al. could show that the decrease in bleeding complications with DAT compared to TAT comes at the expense of a significantly higher risk of stent thromboses (HR 1.59, 95% CI 1.01 - 2.50, p = 0.04) and a borderline increase in the risk of MI (HR 1.22, 95% CI 0.99 - 1.52, p = 0.07) (11). As presented in the post-hoc analysis of the AUGUSTUS trial these ischaemic events primarily occurred within the first month after the index PCI (9).

Activation of blood platelets plays a key role during the early phase of an ACS. Approximately 30-40% of patients show low response to Clopidogrel and are not adequately protected against ischaemic events, in particular when presenting with ACS (12). Consequently, clinical outcomes of patients with ACS undergoing PCI without AF have been significantly improved with the use of potent $P2Y_{12}$ - receptor inhibitors Prasugrel or Ticagrelor. It is therefore reasonable to assume that a more potent platelet inhibition within the first month after PCI might reduce the rate of ischaemic complications observed in AF patients with ACS undergoing PCI, when receiving DAT. On the other hand, a subsequent de-escalation to a less potent platelet inhibition one month after PCI might prevent an increase in bleeding complications. This hypothesis is supported by a landmark analysis of the TRITON-TIMI-38 trial (13), which showed that the beneficial effect of Prasugrel compared to Clopidogrel with regard to ischaemic events was mainly exerted within the first days after the index-MI, while the bleeding complications progressively increased over time with non-significant differences in bleeding rates within the first month of treatment with Prasugrel vs. Clopidogrel.

In a recently published retrospective analysis (14), including 2,259 patients with ACS and AF treated with NOAC and $P2Y_{12}$-inhibitors, treatment with Prasugrel or Ticagrelor compared to Clopidogrel led to a significant decrease of major adverse cardiac events, without a significant increase in bleeding complications after 12 months of follow-up.

In EPIDAURUS we will therefore test the hypothesis that DAT using NOAC plus an escalated antiplatelet therapy with a potent $P2Y_{12}$-inhibitor for one month followed by Clopidogrel reduces

ischaemic events without a relevant increase in bleeding complications in AF patients with ACS undergoing PCI compared to standard in-hospital TAT followed by DAT with NOAC plus Clopidogrel. The lack of evidence, as well as the clinical and scientific importance for a randomized trial comparing the combination of a potent $P2Y_{12}$-inhibitor or Clopidogrel with NOAC in patients with atrial fibrillation is underscored in the recent ESC guidelines of the year 2023 for the management of ACS (15). EPIDAURUS is the only ongoing randomized trial testing the combination of direct factor Xa inhibitors with a potent $P2Y_{12}$ -inhibitor or Clopidogrel in patients with ACS and AF. One other ongoing randomized trial, initiated in Poland (ADONIS-PCI clinicaltrials.gov NCT04695106) will test the combination of direct thrombin-inhibitor Dabigatran with Ticagrelor vs. Dabigatran and Clopidogrel. The recently published retrospective analysis by Godtfredsen et al. supports the clinical hypothesis of the EPIDAURUS trial.

**Table 1:** Summary of the study design, baseline characteristics and endpoints of PIONEER AF-PCI (6), RE-DUAL PCI (5), ENTRUST-AF PCI (3) and AUGUSTUS (4) trials.

| | PIONEER AF-PCI | RE-DUAL PCI | ENTRUST-AF PCI | AUGUSTUS |
|---|---|---|---|---|
| **NOAC** | Rivaroxaban | Dabigatran | Edoxaban | Apixaban |
| **Patients (N)** | 2.124 | 2.725 | 1.506 | 4.614 |
| **Groups** | 3 (open-label) | 3 (open-label) | 2 (open-label) | 2x2 (open/blinded) |
| **Age (Mean ± SD)** | 70 ± 9 | 70 ± 9 | 70 ± 7 | 71 ± 7 |
| **Stable Angina (%)** | 22.2% | 45.8% | 45.6% | 38.8% |
| **Unstable Angina (%)** | 47.7% | 17.9% | 15.6% | |
| **NSTEMI /STEMI (%)** | 30.1% | 36.3% | 39% | 61.2 % (37% PCI) |
| **Clopidogrel (%)** | 94% | 88% | 93% | 93% |
| **Powered for** | Superiority for ISTH bleeding (major/minor) | Non-inferiority for ISTH bleeding (major/minor) | Non-inferiority for ISTH bleeding (major/minor) | Superiority for ISTH bleeding (major/minor) |
| **Follow-up (months)** | 12 | 14 (mean) | 12 | 6 |
| **DAT vs. TAT (HR and 95% CI)** | | | | |
| **ISTH major bleeding** | 0.56 (0.36 – 0.89) | 0.57 (0.43 – 0.76) | 0.94 (0.64 – 1.40) | 0.60 (0.44 – 0.81) |
| **MACE** | 1.03 (0.84 – 1.25) | 1.03 (0.84 – 1.25) | 1.07 (0.73 – 1.58) | 1.13 (0.91 – 1.40) |
| **Stent thrombosis** | 1.25 (0.34 – 4.64) | 1.55 (0.69 – 3.46) | 1.34 (0.47 – 3.84) | 1.92 (0.92 – 3.95) |

CI: confidence interval; NOAC: new oral anticoagulants; HR: hazard ratio; ISTH: International Society on Thrombosis and Haemostasis; MACE: major adverse cardiac events; SD: standard deviation; NSTEMI: non-ST-segment elevation myocardial infarction; STEMI: ST-segment elevation myocardial infarction; DAT: dual antithrombotic therapy; TAT: triple antithrombotic therapy.

### 5.1.2 Purpose

The selection of the optimal antithrombotic therapy in patients with nonvalvular atrial fibrillation (AF) and acute coronary syndrome (ACS) undergoing percutaneous coronary intervention (PCI) is challenging. Until recently, triple antithrombotic therapy (TAT) consisting in Aspirin plus Clopidogrel plus OAC was considered the treatment of choice. While efficiently preventing ischaemic events, TAT is associated with an increase in bleeding complications. Therefore, in the past years several randomized controlled trials challenged TAT by comparing a triple antithrombotic therapy (TAT) regimen based on Vitamin K antagonists (VKA) to a dual antithrombotic regimen (DAT) based on non-vitamin K antagonist oral anticoagulants (NOACs) and $P2Y_{12}$-inhibitors, mainly Clopidogrel in patients with AF undergoing PCI.

However, approximately 30-40% of patients show low response to Clopidogrel and are not adequately protected against ischaemic events, in particular when presenting with ACS. This is supported by a recent meta-analysis (11) reporting that TAT compared to DAT is associated with lower rates of stent thrombosis within 30 days after PCI. It is therefore reasonable to assume that a more potent platelet inhibition within the first month after PCI might reduce the rate of ischaemic complications observed in AF patients undergoing PCI, when receiving DAT. Moreover, a subsequent de-escalation to a less potent platelet inhibition one month after PCI might prevent an increase in bleeding complications.

In EPIDAURUS we will therefore test the hypothesis that DAT using NOAC plus an escalated antiplatelet therapy with a potent $P2Y_{12}$-inhibitor for one month followed by Clopidogrel reduces ischaemic events without a relevant increase in bleeding complications in patients with AF and ACS undergoing PCI compared to standard DAT with NOAC plus Clopidogrel.

## 5.2 Dose Rationale

Treatment in the control group (standard arm) is based on recommendations by current guidelines, which recommend early cessation (<1 week) of TAT and continuation of DAT with NOAC and Clopidogrel in AF patients undergoing PCI (IIa, B recommendation in the current ESC guidelines) (10).

In the experimental (escalated) arm, an escalated antiplatelet therapy with a potent $P2Y_{12}$-inhibitor for one month followed by a de-escalation to Clopidogrel will be applied.

Following enrolment, patients will undergo a 1:1 randomization to a standard therapy (control arm) with an in-hospital TAT (Aspirin, Clopidogrel and NOAC - preferentially Edoxaban or Apixaban) (for a maximum of one week) and a subsequent DAT (NOAC - preferentially Edoxaban or Apixaban plus Clopidogrel) for 6 months or an intensified regimen (interventional arm) of NOAC (preferentially Edoxaban or Apixaban) plus a potent $P2Y_{12}$-inhibitor (Prasugrel (preferred) or Ticagrelor) for the first month (4 weeks) followed by NOAC (preferentially Edoxaban or Apixaban) and Clopidogrel for months 2-6. The default duration of Aspirin therapy in the control arm is in-hospital (up to 1 week). However, the treating physicians are allowed to extend the therapy with Aspirin in the control arm for a period of up to 4 weeks in patients with high ischaemic risk, according to current guidelines (2,10).

After randomization, Aspirin will be continued at a maintenance dose of 75-100mg (for Germany and Austria only 100 mg are applicable) for the in-hospital stay for a maximum of 7-days in the control group (12) and will be immediately discontinued in the intervention group. Switching between Clopidogrel and potent $P2Y_{12}$-inhibition or between potent $P2Y_{12}$-inhibition and Clopidogrel will be

performed according to current recommendations (16). Patients with a new-diagnosed AF will be allowed to participate in the study if they are randomized within 5 days (recommended within 24h) after PCI and before discharge. After completion of the study a DAT with Aspirin or Clopidogrel and NOAC is suggested. No additional loading dose will be required for de-escalation to Clopidogrel (16).

## 5.3 Overall Risk/ Benefit Assessment

In the EPIDAURUS trial we assume that an escalated antiplatelet therapy with a potent $P2Y_{12}$-inhibitor for one month followed by Clopidogrel will reduce ischaemic events, defined as the composite of all-cause mortality, myocardial infarction, definite or probable stent thrombosis, ischaemic stroke and systemic thromboembolism at 6 weeks after randomization.

This hypothesis is supported by the results of several studies. First, activation of blood platelets is known to play a key role during the early phase of an ACS. Approximately 30-40% of patients show low response to Clopidogrel and are not adequately protected against ischaemic events, in particular when presenting with ACS (12). Second, clinical outcomes of patients with ACS undergoing PCI without AF have been significantly improved with the use of potent $P2Y_{12}$ receptor inhibitors Prasugrel or Ticagrelor. Third, in a recent meta-analysis of PIONEER AF-PCI, RE-DUAL PCI, ENTRUST-AF PCI, and AUGUSTUS trials Gargiulo et al. could show that a DAT consisting of NOAC and Clopidogrel compared to an intensified TAT is associated with a higher risk for stent thromboses and a borderline increase in the risk for MI (11). Finally, as presented in the post-hoc analysis of the AUGUSTUS trial these ischaemic events primarily occur within the first month after a PCI (9).

The risk of an intensified antithrombotic therapy depends on the underlying condition. For patients with a previous haemorrhagic stroke, a clinically overt bleeding BARC ≥ 2 within the last month and patients with chronic renal insufficiency, an escalated antiplatelet therapy with a potent $P2Y_{12}$-inhibitor might be associated with an increased rate of bleeding complications. For this reason, patients with previous haemorrhagic stroke, a clinically overt bleeding within the last 4 weeks before index procedure and known chronic renal insufficiency with glomerular filtration rate $< 15$ ml/min/1.73m$^2$ will be excluded from the study. In all other patients the bleeding risk under treatment with a potent $P2Y_{12}$-inhibitor is comparable to the bleeding risk under treatment with Clopidogrel. This is supported by a landmark analysis of the TRITON-TIMI-38 trial (13), which showed that the bleeding rates within the first month of treatment with Prasugrel vs. Clopidogrel did not differ significantly.

In summary, it is reasonable to assume that in patients with AF and MI undergoing PCI, benefit of reduced ischaemic events due to an intensified antithrombotic therapy with a potent $P2Y_{12}$-inhibitor for one month outweighs the risk of bleeding.

# 6 Trial Objectives and Endpoints

The primary study objective is to test whether an escalated antiplatelet therapy with a potent $P2Y_{12}$-inhibitor (Prasugrel or Ticagrelor) for 4 weeks can reduce ischaemic events without a significant increase in bleeding complications in patients with AF and ST-segment elevation myocardial infarction (STEMI) or non-ST-segment elevation myocardial infarction (NSTEMI) (biomarker positive acute coronary syndrome) undergoing PCI.

The effect of platelet function based on platelet function testing (PFT) on ischaemic and bleeding complications will be investigated in a pre-defined substudy.

## 6.1 Primary Endpoint(s)

Primary endpoints at 6 weeks after randomization (hierarchical testing)

- Efficacy: Major ischaemic events defined as the composite of all-cause mortality, myocardial infarction, definite or probable stent thrombosis, ischaemic stroke, systemic thromboembolism or urgent revascularization (superiority test)
- Safety: All cause mortality, bleeding type 2 or higher according to the Bleeding Academic Research Consortium (BARC) criteria (non-inferiority test)

## 6.2 Secondary Endpoints

- All individual components of the primary endpoint (all-cause mortality, myocardial infarction, definite or probable stent thrombosis, ischaemic stroke, systemic thromboembolism) at 6 weeks after randomization
- Cardiovascular mortality at 6 weeks after randomization
- Bleeding (BARC type ≥ 2) at 6 weeks after randomization
- Urgent revascularization at 6 weeks after randomization
- All-cause mortality at 6 months after randomization
- Unplanned hospitalization due to acute heart failure or acute coronary syndrome at 6 months after randomization
- Ischaemic stroke at 6 months after randomization

## 6.3 Additional Event Evaluation

- Any bleeding events (BARC type ≥ 1) at 6 weeks after randomization

# 7 Clinical Trial Protocol

## 7.1 Trial Design

This is a prospective, randomized, open-label, multinational European, multicentre, phase IV clinical trial designed to test whether an escalated antiplatelet therapy with a potent $P2Y_{12}$-inhibitor for one month can reduce ischaemic events without a significant increase in bleeding complications in patients with AF and STEMI or NSTEMI undergoing PCI.

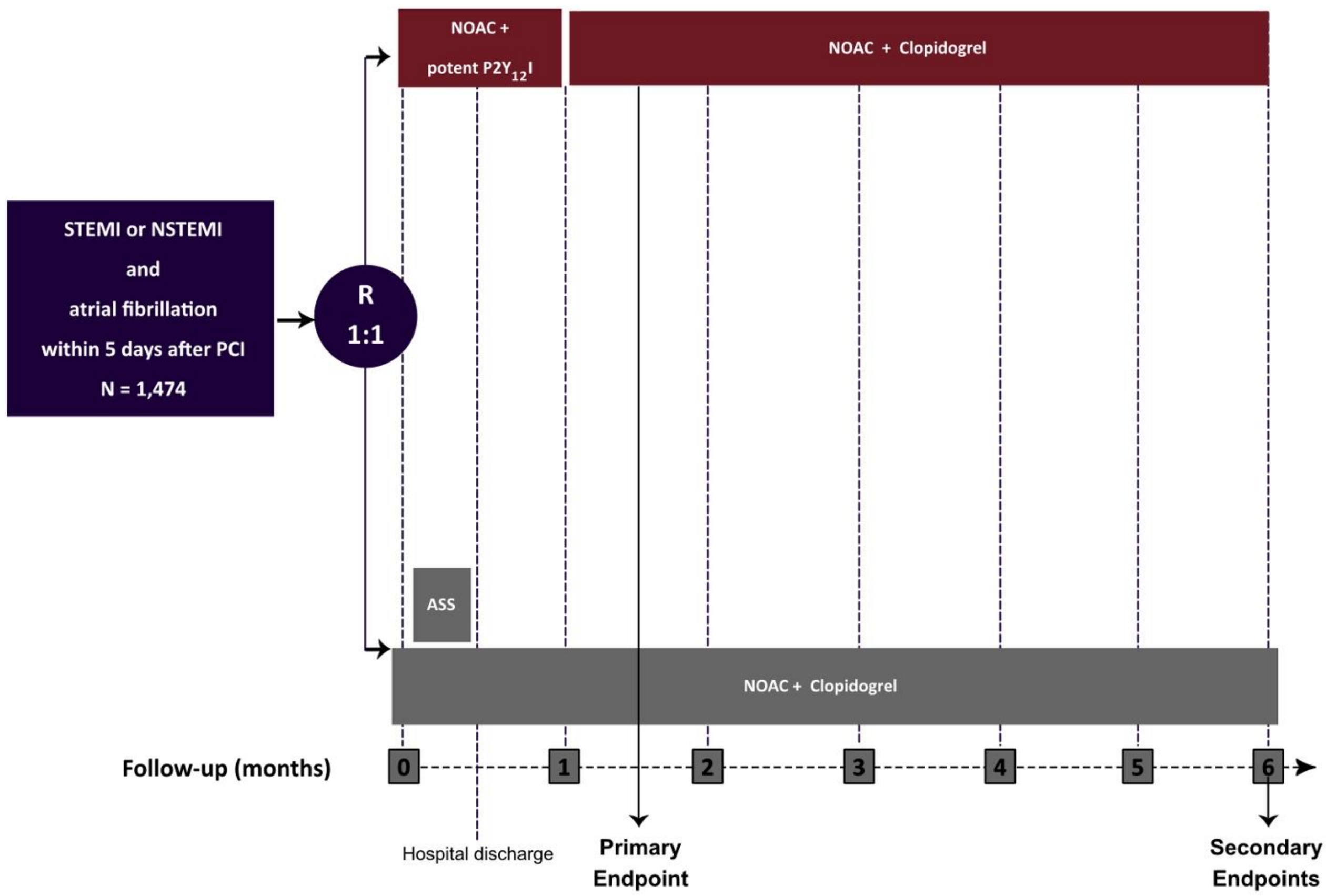


It is planned that the trial is performed in a minimum of 2 countries within the European Union and one country that is not a member state of the European Union. We anticipate that 50-60 sites will participate in this study.

## 7.2 Trial Duration

Planned start of recruitment: Q4/2021

Planned recruiting period: 66 months

Duration by patient: 6 months

End of Trial: Last patient last visit

# 8 Trial Population

Patients with ACS undergoing PCI and AF with an indication for oral anticoagulation with NOAC, after agreement to participate in the clinical trial will be stratified according to the study centre and randomized to a standard antiplatelet therapy with Clopidogrel or an escalated antiplatelet therapy with a potent $P2Y_{12}$-inhibitor.

It is imperative that the patients fully meet all inclusion criteria and none of the exclusion criteria.

## 8.1 Number of patients

A total number of 1474 patients should be allocated to the trial, a number of 1390 patients should be analysed.

## 8.2 Inclusion Criteria

- Written informed consent
- Age ≥ 18 years
- Atrial fibrillation requiring oral anticoagulation
- STEMI or NSTEMI (biomarker positive acute coronary syndrome)
- Successful completion of PCI (defined as TIMI grade 2 or more; randomization will take place within 5 days (recommended within 24h) after successful PCI and before hospital discharge)

## 8.3 Exclusion Criteria

- Chronic renal insufficiency with glomerular filtration rate < 15 ml/min/1.73m$^2$
- History of ischaemic stroke or transient ischaemic attack (both contraindications for Prasugrel) and history of intracranial bleeding (contraindication for Ticagrelor)
- Contraindication for Clopidogrel or Aspirin
- Contraindication for Prasugrel and Ticagrelor
- Severe chronic liver disease (Child-Pugh C)
- Indication for oral anticoagulation with Vitamin K antagonists
- Moderate to severe mitral stenosis or mechanical heart valve
- Any bleeding BARC type ≥ 2 within the last 4 weeks before index procedure
- Pregnancy or lactation
- Inability to cooperate with the protocol requirements
- Life expectancy < 6 months
- Participation in another investigational drug study
- Previous enrolment in this study

- For women of childbearing potential no negative pregnancy test and no agree to use a reliable method of birth control during the study
- Previous treatment with GP IIb/IIIa inhibitors within the last 12 hours
- A known genetic disorder involved in the metabolism of the study medication
- Any other reason in the opinion of the investigator making the patient ineligible for participation in the trial

Women of childbearing potential: include any female who has experienced menarche and who has not undergone successful surgical sterilisation (hysterectomy, bilateral tubal ligation, or bilateral oophorectomy) or is not postmenopausal (amenorrhea ≥ 12 consecutive months)

Reliable methods of birth control: oral contraceptives, other hormonal contraceptives (vaginal products, skin patches, or implanted or injectable products), mechanical products such as an intrauterine device or barrier methods (diaphragm, condoms, spermicides) to prevent pregnancy, or are practicing abstinence or their partner is sterile (e.g. vasectomy).

## 8.4 Patient Recruitment and Screening

Patients presenting with STEMI or NSTEMI to the participating hospital and underwent a successful PCI (defined as TIMI flow grade 2 or more after intervention), who meet all inclusion and no exclusion criteria are eligible for enrolment. Screening is performed within 5 days (recommended within 24h) after completion of PCI and before hospital discharge.

Pre-screening lists will be obtained at the trial sites to document patients assessed for eligibility, but not enrolled in the trial.

# 9 Treatment

## 9.1 Trial Treatment

Following enrolment, patients will undergo a 1:1 randomization to a standard therapy (control arm) with an in-hospital TAT (Aspirin, Clopidogrel and NOAC - preferentially Edoxaban or Apixaban) (for a maximum of one week) and a subsequent DAT (NOAC - preferentially Edoxaban or Apixaban plus Clopidogrel) for 6 months or an intensified regimen (experimental arm) of NOAC (preferentially Edoxaban or Apixaban) plus a potent $P2Y_{12}$-inhibitor (Prasugrel (preferred) or Ticagrelor) for the first month (4 weeks) followed by NOAC (preferentially Edoxaban or Apixaban) and Clopidogrel for 5 months. The NOACs allowed in this trial are the anti-Xa-inhibitors Edoxaban, Apixaban and Rivaroxaban. The dose of the $P2Y_{12}$-inhibitors and NOACs is administered as recommended in the Summary of Product Characteristics (SmPC).

The default duration of Aspirin therapy in the control arm is in-hospital (up to 1 week). However, the treating physicians are allowed to extend the therapy with Aspirin in the control arm for a period of up to 4 weeks in patients with high ischaemic risk, according to current guidelines (2,10).

IMP1: Test product

| | |
|---|---|
| Drug name | Efient® |
| Name of manufacturer | Daiichi-Sankyo |
| Substance name (if applicable, give substance code) | Prasugrel (ATC-Code: B01AC22) |
| Name and dose of active ingredient per unit | Prasugrel as hydrochloride, 5 mg or 10 mg |
| Other ingredients | Tablet Core: microcrystalline cellulose, mannitol (E421), croscarmellose sodium, hypromellose (E464), magnesium stearate<br>Film-Coat: lactose monohydrate, hypromellose (E464), titanium dioxide (E171), triacetin (E1518), Iron oxide red (E172), {only Efient 10 mg}(7) iron oxide yellow (E172), talc |
| Pharmaceutical form | Film coated tablet |
| Mode of administration | Oral |
| Batch number (if applicable) | NA |
| Storage conditions | Store in the original package in order to protect from air and moisture. |

Efient® is given as an example. The IMP is only defined by active substance Prasugrel. All other Prasugrel formulations with a marketing authorization in Germany or in the European Union could be used.

Or

IMP2: Test product

| | |
|---|---|
| Drug name | Brilique® |
| Name of manufacturer | AstraZeneca |
| Substance name (if applicable, give substance code) | Ticagrelor (ATC-Code: B01AC24) |
| Name and dose of active ingredient per unit | Ticagrelor 90 mg |
| Other ingredients | Tablet core: mannitol (E421), calcium hydrogen phosphate dihydrate, magnesium stearate (E470b),<br>sodium starch glycolate type A, hydroxypropyl-cellulose (E463)<br>Tablet coating: talc, titanium dioxide (E171), Iron oxide yellow (E172), macrogol 400, hypromellose (E464) |
| Pharmaceutical form | Film coated tablet |
| Mode of administration | Oral |
| Batch number (if applicable) | NA |
| Storage conditions | None |

IMP3: Reference product

| | |
|---|---|
| Drug name | Plavix® |
| Name of manufacturer | Sanofi |
| Substance name (if applicable, give substance code) | Clopidogrel (ATC-Code: B01AC-04) |
| Name and dose of active ingredient per unit | Clopidogrel 75 mg as hydrogen sulfate |
| Other ingredients | Core: mannitol (E421), macrogol 6000, micro-crystalline cellulose, hydrogenated castor oil,<br>low substituted hydroxypropylcellulose<br>Coating: hypromellose (E464), lactose monohydrate, triacetin (E1518),<br>titanium dioxide (E171) |
| Pharmaceutical form | Film coated tablet |
| Mode of administration | oral |
| Batch number (if applicable) | NA |
| Storage conditions | In PVC/PVDC/aluminium blisters, store below 30°C. In all aluminium blisters, this medicinal product does not require any special storage conditions. |

Plavix® is given as an example. The IMP is only defined by active substance Clopidogrel. All other Clopidogrel formulations with a marketing authorization in Germany or in the European Union could be used.

And

IMP4: Reference product

Germany:

| | |
|---|---|
| Drug name | HerzASS-ratiopharm® 100 mg Tabletten |
| Name of manufacturer | ratiopharm GmbH |
| Substance name (if applicable, give substance code) | Acetylsalicylic Acid (ATC-Code: B01AC06) |
| Name and dose of active ingredient per unit | Acetylsalicylic Acid 100 mg |
| Other ingredients | Maize starch, micro-crystalline cellulose, cellulose powder |
| Pharmaceutical form | Tablet |
| Mode of administration | oral |
| Batch number (if applicable) | NA |
| Storage conditions | Do not store above 25°C, store in original packaging to protect the content from humidity |

HerzASS-ratiopharm® 100 mg is given as an example. The IMP is only defined by active substance Acetylsalicylic Acid. All other Acetylsalicylic Acid formulations with a marketing authorization in Germany or in the European Union could be used.

Austria:

| | |
|---|---|
| Drug name | HerzschutzASS “ratiopharm” 100 mg - Tabletten |
| Name of manufacturer | TEVA B.V. |
| Substance name (if applicable, give substance code) | Acetylsalicylic Acid (ATC-Code: B01AC06) |
| Name and dose of active ingredient per unit | Acetylsalicylic Acid 100 mg |
| Other ingredients | Maize starch, micro-crystalline cellulose, cellulose powder |
| Pharmaceutical form | Tablet |
| Mode of administration | oral |
| Batch number (if applicable) | NA |
| Storage conditions | Do not store above 30°C. Do not store above 25°C, store in original packaging to protect the content from humidity |

HerzschutzASS “ratiopharm” 100 mg is given as an example. The IMP is only defined by active substance Acetylsalicylic Acid. All other Acetylsalicylic Acid formulations with a marketing authorization in Austria or in the European Union could be used.

## 9.2 Treatment Arms

### 9.2.1 Experimental intervention

Escalated regimen consisting of

Weeks 1 – 4: Potent $P2Y_{12}$-inhibitor plus non-vitamin K antagonist oral anticoagulant

Week 5 – month 6: Clopidogrel plus non-vitamin K antagonist oral anticoagulant

### 9.2.2 Control intervention

Standard regimen consisting of

In-hospital (up to 1 week): Aspirin plus Clopidogrel plus non-vitamin K antagonist oral anticoagulant

Discharge or day 8 – month 6: Clopidogrel plus non-vitamin K antagonist oral anticoagulant. In patients with high ischaemic risk the treating physician is allowed to prescribe Aspirin for a period of up to 4 weeks, according to current guidelines.

## 9.3 Treatment Assignment and Randomization

A randomized parallel group design to control for confounding is used. Patients will be randomized within 5 days (recommended within 24h) after completion of successful PCI (defined as TIMI flow grade 2 or more, transradial approach preferred) and before hospital discharge. Participants will be randomly assigned to one of two treatment arms. Randomization will occur in a 1:1 ratio through use of a permuted-block randomization method to ensure a balanced assignment to each treatment arm.

Randomization will be stratified by centre. The randomization list will be uploaded into the randomization module of the EDC system. Study investigators will have access to the randomization module via the eCRF and randomize participants using this central tool.

## 9.4 Packaging, Labelling and Supply

Commercially available IMPs (without special packaging or labelling) are used.

## 9.5 Treatment of Patients

### 9.5.1 Patient Numbering

Each patient is uniquely identified by a Patient ID which is composed by a 2-digit site number followed by a 3-digit subsequent number.

### 9.5.2 Treatment Administration

The patients will be instructed by their treating physicians regarding the intake of IMP after discharge. The IMP will be prescribed by the general practitioner or cardiologist according to the randomized treatment regimen. After 4 weeks the patients randomized in the experimental arm should be switched from Prasugrel or Ticagrelor to Clopidogrel by their treating general practitioner or cardiologist. A patient contact by phone is recommended to ensure timely switch of medication according to the protocol. The NOAC treatment remains unchanged. In the control group the patients remain on their treatment with Clopidogrel and NOAC. The IMPs are taken according to the instruction leaflet of the respective medication. If there is an indication, the treating physician is allowed to change the treatment to another anti-Xa-inhibitor, which however should be documented in the eCRF.

If the patient is switched from Clopidogrel to Ticagrelor after randomization a loading-dose of 180mg Ticagrelor is recommended according to current guidelines (15) and the international expert consensus recommendation (16). If the patient is switched from Ticagrelor to Clopidogrel after randomization or at the end of the 4-weeks treatment period a loading prescription of 600mg Clopidogrel is recommended according to current guidelines (15) and the international expert consensus recommendation (16). For switching between Clopidogrel and Prasugrel and vice versa, no loading is recommended regardless of the timepoint of switching.

### 9.5.3 Compliance

Patients will be interviewed in detail about compliance to study medication at each visit. Patient's compliance will be assessed by the investigator and recorded in the eCRF. Any interruption or termination including the cause of the interruption and termination as well as the exact dates will be documented.

### 9.5.4 Prior and Concomitant Medication

Prior and concomitant medication can be changed and dose might be adjusted according to the decision of the treating physician.

## 9.6 Trial Completion and Discontinuation

### 9.6.1 Trial Completion and Post-Trial Treatment

After the end of the escalated treatment all patients will be treated with NOAC and Clopidogrel for months 2-6. After 6 months, a DAT with Aspirin or Clopidogrel and NOAC, in line with the current guidelines and according to the decision of the treating physician is suggested.

A patient will be considered to have completed the trial when the patient has completed the last visit planned in the protocol (final visit Day 180 ± 10 days).

After study completion patients will be treated at the discretion of the responsible physician. As a result the aspect of contraception after study completion depends on further treatment of the patient in line with the current guidelines and the respective SmPCs.

### 9.6.2 Lost to Follow-up

A patient will be classified as lost to follow-up only if, his/her vital status remains unknown despite multiple attempts to contact him/her in- or out of hospital via telephone, fax, email, or letter until the last scheduled follow-up of the last patient is completed.

### 9.6.3 Discontinuation of Patients from Treatment or Trial Participation

If a patient was withdrawn before completing the trial, the reason for withdrawal must be entered in the patient's file and the eCRF.

Patients will be withdrawn from the trial/treatment for the following reasons:

- withdrawal of consent by the subject for any reason at any time
- if continued trial participation is not in the best interest of the subject, as judged by the investigator (e.g. due to clinical adverse event, laboratory abnormality or inter-current illness)
- termination of the trial by regulatory authorities or ethics committees
- patients, who become prisoners, involuntarily incarcerated or compulsorily detained for treatment of either a psychiatric of physical illness

Patients who discontinue trial treatment prematurely should conduct the follow-up visits as far as possible.

Patients have the right to withdraw their consent at any time without providing a reason without prejudice to their future medical care by the physician or at the institution. The signed informed consent form must remain in the Investigator Site File and must be available for verification by monitors or authorities at any time. Withdrawal of full consent for a study means that the patient does not wish to receive further investigational treatment and does not wish or is unable to continue further study participation. Patient data up to withdrawal of consent will be included in the patient's study data, but no further information will be collected unless a separate consent has been given. Withdrawal of partial consent means that the patient does not wish to take protocol-specific product(s) any longer but is still willing to collaborate in providing further data by continuing on study. Should a patient request or decide to withdraw from the study, all efforts will be made to complete and report the observations as thoroughly as possible up to the end of withdrawal.

For any patient who has received IMP and withdrew prematurely from the study every effort should be made for attendance of a safety follow-up visit. The primary reason for withdrawal from the study should be documented. Patients will not be followed for any reason after consent has been withdrawn, unless a separate consent has been given for further data collection.

### 9.6.4 Conditions for Terminating the Trial by the Sponsor

The sponsor reserves the right to temporarily suspend or prematurely discontinue this trial either at a single site or at all sites at any time for reasons including safety or ethical issues, severe non-compliance, recurrent non-compliance, or unsatisfactory enrolment with respect to quality or quantity.

If the trial is prematurely terminated or suspended, the sponsor will promptly inform the Principal Investigators and the Member States concerned of the termination or suspension and the reason(s) for the termination or suspension.

In case of a premature termination of the trial, patients will be followed-up at least until all enrolled patients underwent their final visit.

# 10 Trial Procedures

A schedule of activities is provided in section 2.

## 10.1 Screening / Baseline Visit

It is the responsibility of the investigator, or a person delegated by the investigator to obtain written informed consent from each patient prior to participation in the trial, following adequate explanation of the aims, methods, anticipated benefits and potential hazards of the trial.

No clinical trial procedures will be conducted prior to taking consent from the participant. Randomization will denote the enrolment into the trial. Data obtained as part of the normal patient care can be used for screening/baseline visit if collected within 72h prior to randomization.

The following procedures are performed:

- Informed consent
- Assessment of in- and exclusion criteria
- Demographic data (age, gender, race), general and cardiovascular medical history and current diseases:
    - Symptoms: angina pectoris (CCS classification), dyspnoea (NYHA classification), syncope
    - Cardiovascular risk factors: Smoking status, arterial hypertension, diabetes mellitus, hypercholesterolaemia, family history of coronary artery disease
    - Cardiovascular history: Previous myocardial infarction, previous PCI, previous CABG, previous stroke, previous stent thrombosis, previous bleeding BARC ≥ 2 (within the last 4 weeks before index procedure), peripheral arterial occlusive disease (PAOD), cerebrovascular disease (CVD), atrial fibrillation, aortic stenosis, aortic insufficiency, mitral insufficiency, mitral stenosis, tricuspid insufficiency, pacemaker/ICD/CRT

- Medical history: COPD (Gold), chronic renal failure (CRF) and stage, abnormal liver function, history of cancer, alcohol abuse

- Clinical parameters: Weight and height at admission, blood pressure and heart rate post PCI at screening, ECG at admission and post PCI at screening, ECG risk stratification (optional)
- Laboratory parameters (admission): Laboratory tests at admission depend on the routine tests performed in each study centre. We recommend following tests: Haemoglobin, erythrocytes, leukocytes, thrombocytes, creatinine, BUN, CK, troponin, LDH, CRP, BNP/NT-proBNP, HbA1c, total cholesterol, LDL, HDL, GOT, GPT, bilirubin, INR, platelet aggregation tests (substudy at pre-defined sites)
- Laboratory parameters (post PCI and prior to randomization): Laboratory tests post PCI depend on the routine tests performed in each study centre. We recommend following tests: Haemoglobin, CK, troponin.
- Pregnancy test for women of childbearing potential, contraception (if applicable)
- Concomitant medication will be recorded:

  - At admission: Aspirin, $P2Y_{12}$-inhibitors, OAC, beta-blocker, angiotensin-converting-enzyme inhibitors (ACEI), angiotensin II receptor blockers (ARB), aldosterone antagonist, angiotensin-receptor-neprilysin-inhibitor (ARNI), SGLT2 inhibitors, amiodarone, digitalis, calcium channel blockers (CCB), nitrates, statins, non-steroidal anti-inflammatory drugs (NSAIDs)
  - Peri-interventional (including emergency medication): Aspirin, $P2Y_{12}$-inhibitors, heparin, GP IIb/IIIa inhibitors
  - At screening (planned medication after PCI and before randomization): Aspirin, $P2Y_{12}$-inhibitors, OAC, beta-blocker, ACEI/ARB, aldosterone antagonist, ARNI, SGLT2 inhibitors, amiodarone, digitalis, CCB, nitrates, statins
- Coronary angiography/PCI: CAD and vessels involved, PCI number of stents, PCI bifurcation, kissing balloon, PCI ostial, PCI LCA, PCI CABG, PCI planned and which vessels, planned bifurcation, planned ostial stenosis, planned LCA, planned CABG, success of PCI (TIMI grade 2 or more), left-ventricular ejection fraction (LVEF; angiographic or echocardiographic)
- Randomization within 5 days (recommended within 24h) after successful completion of PCI (defined as TIMI flow grade 2 or more) and before hospital discharge: All patients who meet the inclusion and none of the exclusion criteria are randomized 1:1 into two groups:

Group 1 (experimental): escalated antiplatelet therapy with a potent $P2Y_{12}$-inhibitor (Prasugrel or Ticagrelor):

Concomitant antiplatelet treatment with Aspirin will be discontinued in this group.

Group 2 (control): treatment with Clopidogrel:

Aspirin will be continued at a maintenance dose of 75-100mg/day (for Germany and Austria only 100 mg are applicable) for the in-hospital stay for a maximum of 7 days in the control group. According to the current guidelines, Aspirin is allowed to be continued for a maximum of 4 weeks in patients with high ischaemic risk.

In both groups:

If the patients are already on treatment with an anti-Xa-inhibitor (Edoxaban, Apixaban or Rivaroxaban) the anti-Xa-inhibitor will be continued.

In patients not on treatment with NOACs or in patients on treatment with VKA or anti-IIa-inhibitor, an anti-Xa-inhibitor will be initiated (preferentially Edoxaban).

## 10.2 Visit at Discharge

- Laboratory parameters: CK max, troponin max
- Assessment of adverse events and occurrence of endpoints
- Concomitant medication at discharge: Aspirin, $P2Y_{12}$-inhibitors, OAC, beta-blocker, ACEI/ARB, aldosterone antagonist, ARNI, SGLT2 inhibitors, amiodarone, digitalis, CCB, nitrates, statins

Four weeks after randomization a patient contact by phone for patients randomized into the experimental arm is recommended to ensure timely switch of medication according to the protocol.

## 10.3 Control Visit (Day 42 ± 7)

The visit can be performed either in the hospital, by phone or letter (for patients not included in the substudy).

- Assessment of adverse events and occurrence of endpoints
- Symptoms: angina pectoris (CCS classification), dyspnoea (NYHA classification), syncope
- Concomitant medication: Aspirin, $P2Y_{12}$-inhibitors, OAC, beta-blocker, ACEI/ARB, aldosterone antagonist, ARNI, SGLT2 inhibitors, amiodarone, digitalis, CCB, nitrates, statins
- Laboratory: Laboratory parameters that were assessed after discharge of index hospital stay (if applicable)
- Applicable for Austria only: Pregnancy test: after randomization monthly pregnancy tests for women of childbearing potential

## 10.4 Final Visit (Day 180 ± 10)

The visit can be performed either in the hospital, by phone or letter.

- Assessment of adverse events and occurrence of endpoints
- Concomitant medication: Aspirin, $P2Y_{12}$-inhibitors, OAC
- Smoking status
- Applicable for Austria only: Pregnancy test: after randomization monthly pregnancy tests for women of childbearing potential

# 11 Safety Monitoring

## 11.1 Definitions

Definitions have been taken from the Regulation (EU) No 536/2014 of the European Parliament and of the council of 16 April 2014 on clinical trials on medicinal products for human use.

### 11.1.1 Adverse Event (AE)

An Adverse Event is any untoward medical occurrence in a subject to whom a medicinal product is administered and which does not necessarily have a causal relationship with this treatment.

An AE can therefore be any unfavourable and unintended sign (including an abnormal laboratory finding, for example), symptom, or disease temporally associated with the use of a medicinal product whether or not considered related to the medicinal product.

Pre-existing diseases or symptoms or abnormal lab values present before randomization should not be considered an AE unless the condition worsens or episodes increase in frequency during trial participation.

### 11.1.2 Adverse Reaction (AR)

In determining whether an adverse event is an adverse reaction, considerations shall be given to whether there is a reasonable possibility of establishing a causal relationship between the event and the investigational medicinal product based on an analysis of available evidence.

### 11.1.3 Serious Adverse Event (SAE)

A Serious Adverse Event is any untoward medical occurrence that at any dose

- results in death
- is life-threatening
- requires inpatient hospitalization or prolongation of existing hospitalization
- results in persistent or significant disability or incapacity
- results in a congenital anomaly or birth defect
- is qualified as another important medical event or condition (some medical events may jeopardize the patient or may require an intervention to prevent one of the above characteristics/consequences). Such events should also be considered as serious in accordance with the definition.

These characteristics/consequences have to be considered at the time of the event. For example, regarding a life-threatening event, this refers to an event in which the subject was at risk of death at the time of the event; it does not refer to an event which hypothetically might have caused death if it were more severe.

### 11.1.4 Suspected Unexpected Serious Adverse Reaction (SUSAR)

An Unexpected Serious Adverse Reaction is a serious adverse reaction, the nature, severity or outcome of which is not consistent with the reference safety information. The reference safety

information for the IMPs is the effective SmPC of Efient®, Brilique®, Plavix® and HerzASS-ratiopharm® 100 mg (Germany) or HerzschutzASS "ratiopharm" 100 mg (Austria), respectively.

## 11.2 Assessment Criteria

### 11.2.1 Adverse Event Assessment by the Investigator

The investigator should evaluate all AEs according to the criteria and steps mentioned below.

#### 11.2.1.1 Assessment of Seriousness

For each AE the investigator determines its seriousness according to the definition provided in section 11.1.3. In case the AE fulfils one or several seriousness criteria, the SAE has to be recorded on the SAE page of the eCRF and reported to the sponsor and the designee responsible of safety evaluation immediately.

#### 11.2.1.2 Assessment of Intensity

All AEs reported in the eCRF have to be graded regarding their intensity.

| | |
|---|---|
| MILD | The patient is aware of the sign or symptom, but it does not interfere with his/her usual activities and/or it is of no clinical consequence. |
| MODERATE | The AE interferes with the usual activities of the patient or it is of some clinical consequence. |
| SEVERE | The AE interferes significantly with patient's usual function, incapacitating with inability to work or carry out usual activity or the AE is of definite clinical consequence. |

#### 11.2.1.3 Assessment of Causality

The relationship (causality) of an AE to the investigational product(s) is evaluated according to the following classification:

| | |
|---|---|
| Not related | There is not a reasonable possibility that the AE is related to the IMP. Alternative aetiology, diagnosis or explanation for the event should be provided. |
| Related | There is a reasonable possibility that the AE is related to the IMP. This means that there are facts (evidence) or arguments to suggest a causal relationship. |

The following should be taken into consideration:

- a temporal relationship
- a pharmacologically or biologically plausible event
- positive de-challenge after stopping respectively re-challenge after restarting of IMP
- presence of confounding factors, such as concomitant medication, concurrent illness or relevant medical history

### 11.2.2 Assessment by the Sponsor

In order to fulfil the sponsor's responsibilities of ongoing safety evaluation and reporting obligations, the sponsor performs a sponsor assessment of each reported SAE concerning seriousness, causality and whether it is expected or not. The reference safety information for classification of expected or unexpected SAEs are the current versions of the Summaries of Product Characteristics

(SmPC) of Efient®, Brilique®, Plavix® and HerzASS-ratiopharm® 100 mg (Germany) or HerzschutzASS “ratiopharm” 100 mg (Austria).

In addition, a risk-benefit evaluation is performed by the sponsor describing whether the SAE report implies a safety issue that

- might alter the risk-benefit assessment
- might be sufficient to consider changes in the conduct of the study.

## 11.3 Collection and Reporting of (Serious) Adverse Events

### 11.3.1 Adverse Events

All AEs which occur after randomization until the 6 months follow-up visit (final visit) must be collected by the investigator and recorded on the patient file.

Recording in the eCRF is only required for events meeting the following criteria:

1. Serious adverse events (Definition see section 11.1.3)
2. Important medical events (see SAE definition in section 11.1.3)
3. Medication error/overdose (intentional or unintentional): at least to be reported as protocol deviation (see section 11.5)
4. Adverse events that led to study drug discontinuation
5. Pregnancy and lactation exposure and outcomes
6. Adverse events of special interest: i.e. all endpoint relevant non-serious adverse events as well as bleedings BARC ≥ 1

This selective approach of safety data collection according to the ICH guideline E19 on a selective approach to safety data collection (17) is justified because the safety profile of all IMPs (Aspirin, Clopidogrel, Prasugrel and Ticagrelor) is sufficiently characterized and all prerequisites listed in paragraph 2.2 of the referenced guideline including mechanistic, clinical safety, clinical pharmacology and non-clinical toxicology factors are met. Moreover, the EPIDAURUS trial shows several similarities to approval and post-approval studies for potent $P2Y_{12}$-inhibitors and there is a plenty of qualitative post-approval surveillance data available. All these factors support a selective safety data collection in the EPIDAURUS trial.

The patient will be given the opportunity to report AEs spontaneously to the Investigator. At each study visit after randomization, the patient has to be asked by non-directive questioning about his health status and the occurrence of events, e.g., “Did you notice anything unusual about your health (since your last visit)?” In order to prevent reporting bias, subjects should not be questioned regarding the specific occurrence of one or more AEs. Nevertheless the investigator must ensure that endpoints are captured.

Abnormal laboratory values or test results have to be reported as adverse event only if they lead to discontinuation of the IMP.

Clinically significant abnormal laboratory values or test results must be identified through a review of values outside of normal ranges, significant changes from baseline or the previous visit, or values which are considered to be non-typical in patients with the underlying disease.

If known, the diagnosis of the underlying illness or disorder should be recorded by the investigator, rather than its individual symptoms. All signs, symptoms, and laboratory test findings related to the underlying diagnosis should be collectively recorded as a single diagnosis. The following information should be captured for all reported AEs: onset, stop date (duration), intensity, seriousness, relationship to investigational medicinal product, action taken, treatment required and outcome.

AEs should be followed up until resolution, stabilization or deemed irreversible.

### 11.3.2 Serious Adverse Events

#### 11.3.2.1 Content of SAE Reports and Reporting

If an AE fulfils one or more of the seriousness criteria an SAE report has to be completed in addition to the documentation on the AE page of the eCRF.

All SAEs must be reported by the investigator to the sponsor immediately (latest within 24 hours) after becoming aware of the event by entering the event in the respective SAE section of the eCRF. In some instances when the eCRF is not available the SAE should be reported by confirmed facsimile transmission (fax). If only limited information is initially available, follow-up reports are required. If an ongoing SAE changes in its intensity or relationship to the investigational product, a follow-up SAE report should be sent immediately to the sponsor. As follow-up information becomes available it should be sent immediately using the same procedure used for transmitting the initial SAE report. All SAEs should be followed up until resolution, stabilization or deemed irreversible.

The SAE report must contain at least the following criteria for a valid report:

- Patient identification (patient ID)
- SAE (diagnosis/symptom/description)
- Suspect IMP
- Reporting source (name, date and signature of the reporting investigator)

The following information should be provided on the SAE report in addition:

- Start date and time (time only during hospitalization for index procedure)
- Seriousness criteria
- IMP administration
- Action taken concerning IMP, de- / re-challenge
- Causality of SAE to IMP
- Other potential causes for SAE occurrence
- Intensity of the SAE
- SAE treatment
- Outcome and end date of the SAE
- Concomitant diseases and concomitant medication
- Laboratory values and supporting information (e.g. discharge letters, results of examinations)

A detailed guideline regarding completion of SAE pages in the eCRF and SAE reporting will be provided to the investigators.

#### 11.3.2.2 Exclusion from Reporting of SAEs

The following hospitalizations are not considered SAEs:

- a visit to the emergency room or other hospital department < 24 hours, that does not result in admission (unless considered “important medical event” or “life threatening” event)
- routine health assessment requiring admission for baseline/trending of health status (e.g. routine colonoscopy)
- medical admission for purpose other than remedying ill health state and was planned prior to randomization. Appropriate documentation is required in these cases.
- admission for social reasons encountered for another life circumstance that carries no bearing on health status and requires no medical/surgical intervention (e.g. lack of housing, economic inadequacy, care-giver respite, family circumstances, administrative)
- scheduled hospitalizations for diagnostic or administrative procedures not related to study procedures
- hospitalization due to logistical or geographical reasons (patient’s home too remote for outpatient treatment)

#### 11.3.2.3 Period of SAE Reporting

All SAEs which occur after randomization until the 6 month follow-up visit must be reported on the SAE Report Form provided in the eCRF to the sponsor.

## 11.4 Expedited Reporting

If a Serious Adverse Event is assessed as a Suspected Unexpected Serious Adverse Reaction (SUSAR), the sponsor or his designee has to submit the event to Eudravigilance. The participating investigators will be informed as well.

## 11.5 Reporting of Trial Treatment Errors including Misuse / Abuse

The occurrence of treatment errors of the IMP including misuse and abuse has to be documented as protocol deviation and reported as AE/SAE in case the definition of an AE/SAE and requirements for safety reporting described in sections 11.1 to 11.3 are met.

## 11.6 Pregnancy Reporting

In case of a pregnancy at the time of investigational product exposure or during follow-up period, the sponsor or his designee has to be notified by the investigator. The reporting obligation of the investigator starts immediately after coming to know of the patient being pregnant. The pregnancy has to be reported by the investigator on the respective Pregnancy Report Form. The investigator actively follows up on the further course of the pregnancy until delivery and on the condition of the new born.

If the investigator obtains knowledge of adverse events of the pregnant patient, the mother or the new born child, which fulfil the serious criteria specified in the protocol, the protocol specific documentation and reporting procedures apply. The sponsor evaluates the case of pregnancy regarding possible consequences for the pregnant patient, for other patients and/or the progress of the trial.

## 11.7 Annual Safety Report

The sponsor provides an Annual Safety Report including appendices such as line listing of Serious Adverse Reactions and cumulative summary tabulation of SAEs. The Annual Safety Report is submitted to the Member States concerned.

# 12 Statistics

## 12.1 Sample Size Calculation

The efficacy composite endpoint includes (i) death, (ii) definite or probable stent thrombosis, (iii) myocardial infarction, (iv) stroke, (v) systemic embolism, and (vi) urgent revascularization. The safety composite endpoint includes (i) death, (ii) BARC 3c bleeding, (iii) BARC 3b bleeding, (iv) BARC 3a bleeding, and (v) BARC 2 bleeding. The endpoints will be tested using a hierarchical design with superiority testing of the first endpoint and non-inferiority testing of the second endpoint, if superiority of the first endpoint is met. Both composite endpoints will be analyzed using Finkelstein and Schoenfeld test (win-loss-ratio; [WLR] (18) using a decreasing ranking. For sample size calculation we assumed a normal distribution of hazards and applied a simulation approach using the hazard-rates and standard errors (SE) provided in Table 2. The event-rates, hazard-rates, SE of hazard rates and drop-out rates of the control group were derived from an analysis of the 223 control patients included in EPIDAURUS until November 4th, 2024. In order to avoid power-loss no direct comparison between control and intervention groups was performed. The assumed effect of intervention on outcome remained unchanged (cumulative odds ratio 0.67 for the ischaemic endpoints and 0.95 for the bleeding endpoints) compared to previous protocol versions and was derived from unpublished data of the ISAR REACT 5 trial. The assumed effect size is in-line with a recently published retrospective analysis comparing escalated vs. standard therapy in patients with AF and ACS (14).

Assumptions for sample-size calculation (hierarchical testing)

- Primary efficacy endpoint (ischaemic events) at 6 weeks

  Superiority testing, 2-sided alpha 5%, power 80%, 6 weeks hazard-rates in the control group on the basis of the analysis of the control patients enrolled in EPIDAURUS until November, 4th 2024 (Table 2). Hazard-rates in the interventional group are derived from a post-hoc analysis of the ISAR-REACT 5 trial and a recently published retrospective analysis comparing escalated vs. standard therapy in patients with AF and ACS (14) (8,19).

  Null hypothesis H0: WLR =1. For a two-sided alpha of 0.05, 6-week hazard-rates and SE of hazard rates according to the values provided in Table 2 the power is 80.0% if comparison of WLR is performed on 615 patients per group. The computation assumes an allocation ratio ($n_{control}/n_{interventional}$) = 1.0. To account for dependency between the different endpoints the sample size was increased by 10% (20)[, leading to a sample size of **677** patients per group. The computation assumes an allocation ratio (ncontrol/ninterventional) = 1.0.

  Number per group: **677**

- <u>Primary safety endpoint (bleeding BARC ≥ 2) at 6 weeks</u>

  Non-inferiority testing for mortality, bleeding BARC 3c, BARC 3b, BARC 3a and BARC ≥ 2) at 6 weeks tested using the Finkelstein and Schoenfeld test (WLR) and application of decreasing ranking for the components of the safety endpoint. When the sample size in each group is **695**, a two-group large-sample normal approximation test of proportions with a one-sided 2.5% significance level will have 80% power to reject the null hypothesis that the lower 97.5% confidence interval of the WLR compared between the intervention and control arm is lower than 0.77 (1/(1 + delta), with delta = 0.3) in favour of the alternative hypothesis that the lower 97.5% confidence interval is greater than or equal to 0.77. A dependency between the endpoints was not considered, as the endpoints of the safety analysis are mutually exclusive.

The sample size of the trial is given by the maximum of both sample sizes: 695 per group. Assuming an allocation ratio ncontrol/ninterventional = 1.0 and a 6% drop-out rate (derived from an analysis of available data), the sample study of the study was calculated to be 1474. (1390 * 1.06 = **1474**).

**Table 2:** 6-week event-rates, hazard-rates and their corresponding standard errors (SE), which were used for sample-size calculation.

| | Control group | | | Intervention group | | | HR |
|---|---|---|---|---|---|---|---|
| **Endpoint** | **Event rate** | **Hazard rate** | **SE of hazard rate** | **Event rate** | **Hazard rate** | **SE of hazard rate** | |
| **Death** | 4.35% | 4.44% | 1.57% | 3.28% | 3.33% | 1.18% | 0.75 |
| **Stent thrombosis** | 1.17% | 1.18% | 0.84% | 0.34% | 0.35% | 0.25% | 0.30 |
| **Myocardial infarction** | 3.79% | 3.87% | 1.46% | 1.91% | 1.93% | 0.73% | 0.50 |
| **Ischaemic stroke** | 1.00% | 1.01% | 0.50% | 0.70% | 0.70% | 0.35% | 0.70 |
| **Systemic embolism** | 1.00% | 1.01% | 0.50% | 0.70% | 0.70% | 0.40% | 0.70 |
| **Urgent revascularization** | 2.77% | 2.81% | 1.26% | 1.39% | 1.40% | 0.63% | 0.50 |
| **BARC 2 bleeding** | 7.85% | 8.18% | 2.13% | 7.48% | 7.77% | 2.02% | 0.95 |
| **BARC 3a bleeding** | 3.22% | 3.28% | 1.34% | 3.06% | 3.11% | 1.28% | 0.95 |
| **BARC 3b bleeding** | 1.07% | 1.07% | 0.77% | 1.01% | 1.02% | 0.73% | 0.95 |
| **BARC 3c bleeding** | 1.00% | 1.01% | 0.50% | 0.95% | 0.95% | 0.47% | 0.95 |

## 12.2 Definition of Population Included in the Analysis

The analysis of the primary and secondary endpoints will be performed according to the intention to treat (ITT) principle, i.e., in all patients that have been randomized irrespective of their protocol adherence or continued participation in the study.

The safety endpoint of bleeding complications will be analysed in a modified intention to treat (mITT) population, i.e. in all randomized patients that received the randomly assigned study medication with endpoints occurring within 7 days after drug discontinuation.

## 12.3 Statistical Methods

This is a non-blinded, randomized, multicentre trial. We will use hierarchical testing to assess the primary endpoints (efficacy and safety). The efficacy composite endpoint includes (i) death, (ii) definite or probable stent thrombosis, (iii) myocardial infarction, (iv) stroke, (v) systemic embolism, and (vi) urgent revascularization. The safety composite endpoint includes (i) death, (ii) BARC 3c bleeding, (iii) BARC 3b bleeding, (iv) BARC 3a bleeding, and (v) BARC 2 bleeding. The endpoints will be tested using a hierarchical design with superiority testing of the first endpoint and non-inferiority testing of the second endpoint, if superiority of the first endpoint is met. Both composite endpoints will be analyzed using Finkelstein and Schoenfeld test (win-loss-ratio; [WLR] (18) using a decreasing ranking.

We will calculate the WLR using an unmatched approach with application of bootstrapping (boot.n = 1.000 iterations) for calculation of SE and 95% confidence intervals (21). The results will be presented using forrest plots.

We will also use Kaplan-Maier estimates to visualize time-to specific events (ischaemic events and bleeding events). A similar approach will be used for secondary endpoints. The exact elaboration of the analysis takes place in a statistical analysis plan (SAP), which will be available before the first evaluation.

As described, this is a study with several endpoints. This leads to two hierarchical checks.

Efficacy endpoint:

Superiority testing, 2-sided alpha 5%, power 80%, 6 weeks event-probability in the control group based on a post-hoc analysis of the ISAR-REACT 5 trial 7% (data not published), 6 weeks event-probability in the intervention group 4.3%, corresponding to an odds-ratio of 0.67 (8,19).

Null hypothesis H0: OR =1 (difference). For a two-sided alpha of 0.05, 6-week event probability in the control group of 0.070 and in the intervention group of 0.043 (OR = 0.6) the power is 80.0% if the chi-square test is performed on 1147 patients per group. The computation assumes an allocation ratio (n2/n1) = 1.0.

Safety endpoint:

Non-inferiority testing, when the sample size in each group is 1152, a two-group large-sample normal approximation test of proportions with a one-sided 2.5% significance level will have 80% power to reject the null hypothesis that the test and the standard are not equivalent (6-week event-rate in the control group 13.1% (based on unpublished data of the ISAR-REACT 5 trial), odds-ratio between treatment and control-group 0.95, difference in proportions, $\pi_1$ - $\pi_0$, is -0,033 or farther from zero in the same direction) in favour of the alternative hypothesis that the proportions in the two groups are equivalent.

## 12.4 Pre-specified subgroup analysis

Furthermore, the following subgroups will be examined:

- Age (Cut-off ≥/< 65)
- Gender
- STEMI or NSTEMI
- AF (paroxysmal or not)

- Apixaban, Edoxaban, Rivaroxaban
- Ticagrelor, Prasugrel
- On treatment with $P2Y_{12}$-inhibitor
- On treatment with Aspirin (≤ 7 days or > 7 days)
- On treatment with OAC
- Multivessel disease yes or no
- Bifurcation, ostial, 3-vessel PCI
- Peripheral arterial occlusive disease (PAOD) or cerebrovascular disease (CVD)
- CHA2DS2-VASc ≥ 3
- HAS-BLED ≥ 3
- Previous stroke
- Previous MI
- NYHA > II
- BNP cut-off median
- eGFR < 60 mL/min/1.73$m^2$
- Max CK > 1000 U/L from admission up to 48 h after completion of PCI
- Increased PRD or decreased DC. PRD will be calculated from 20-Min ECGs recorded in Frank-leads configuration or from normal 10-second 12-lead ECGs. DC will be estimated from 20-Min ECG recordings.
-

## 12.5 Handling of Protocol Deviations and Missing Data

Missing values in baseline, laboratory and clinical characteristics will be replaced by imputation procedures.

All analyses should be performed in an intention to treat basis. The safety endpoint of bleeding complications will be analysed in a modified intention to treat (mITT) population, i.e. in all randomized patients that received the randomly assigned study medication with endpoints occurring within 7 days after drug discontinuation.

In addition, we will perform per-protocol (as treated) analyses as sensitivity analyses for all primary and secondary endpoints.

# 13 Substudy

Platelet function test substudy:

In a predefined substudy of EPIDAURUS (EPIDAURUS-PFT), the effect of platelet function on the primary and secondary endpoints will be examined. This substudy will clarify the debated role of platelet reactivity as a potential determinant for cardiovascular outcome. Platelet function testing will be applied in the centres, where PFT-assays are available and performed within clinical routine and

the centres agree to participate in the substudy. Available platelet function testing results starting from 2 hours after loading will be recorded in the eCRF. In addition, a platelet function testing is recommended but is not obligatory at the 6-week clinical visit. All commercially available assays are allowed to be used in the substudy. At the end of the study the effect of PFT-testing on the primary and secondary endpoints will be examined using multivariable cox-regression analysis.

# 14 Quality Assurance and Control

## 14.1 Routine Monitoring

Monitors will visit the site to check completeness of patient records, accuracy of data capture / data entry, adherence to the protocol and to Good Clinical Practice and progress of enrolment.

The extent and frequency of monitoring and percentage of source data verification performed is defined in the Monitoring Plan.

The investigator agrees to cooperate with the monitor to ensure that any problems detected in the course of these monitoring visits are addressed and resolved. The investigator allows the monitor to have access to trial related original data and documents relevant for monitoring of the trial.

## 14.2 Compliance with Protocol and Protocol Revisions

The investigator should conduct the trial in compliance with the protocol agreed to by sponsor and approved by the Member States concerned

Any significant protocol deviation will be documented and explained by the investigator or the person designated by the investigator in the eCRF and reported to the sponsor or his designee and will be included in the submission of results. Corrective and preventive actions (CAPA) may be developed by the site and/or the sponsor or his designee, implemented and followed up as appropriate.

The study shall be conducted as described in this approved protocol. All revisions to the protocol must be discussed with, and be prepared by sponsor. The investigator should not implement any deviation or change to the protocol without prior review and documented approval of an amendment by the Member States concerned, except where necessary to eliminate an immediate hazard(s) to study subjects. Any deviation must be documented in the eCRF.

If a deviation or change to a protocol is implemented to eliminate an immediate hazard(s) prior to obtaining approval by the Member States concerned, as soon as possible the deviation or change will be submitted to:

- Sponsor
- the Member States concerned for review and approval

If an amendment substantially alters the study design or increases the potential risk to the subject:

(1) the informed consent form must be revised and submitted to the Member States concerned for review and approval;

(2) the revised informed consent form must be used to obtain consent from patients currently enrolled in the study if they are affected by the amendment; and

(3) the new informed consent form must be used to obtain consent from new patients prior to enrolment.

## 14.3 Measures of Quality Assurance and Quality Control

### 14.3.1 Quality Control

Quality Control will be applied to each stage of data handling to ensure that all data are reliable and have been processed correctly, e.g. by source data verification by monitors and data management procedures. A comprehensive Quality Management System with written SOPs and procedures is in place covering all processes and procedures to ensure a compliant conduct of the trial and that data are generated, recorded and reported in compliance with the protocol, GCP, and the applicable regulatory requirement(s).

### 14.3.2 Quality Assurance

An independent Quality Assurance is established at the Deutsches Herzzentrum München (Quality Management Representative Clinical Research). For this clinical trial an audit plan is developed and implemented according to respective SOPs comprising audits of service provider and investigator sites. In addition, regular systems audits are conducted at the ISAResearch Center, Deutsches Herzzentrum München as a service provider, to ensure independent review of trial related processes and procedures including follow-up and closure of CAPAs.

The sponsor ensures documented oversight of all contracted trial-related duties and functions including all trial-related duties and functions that are subcontracted to another party by the sponsor's contracted CRO.

Direct access to all trial related sites, source data, documents and reports for the purpose of auditing by the sponsor and inspection by domestic or foreign regulatory authorities is ensured.

# 15 Committees

## 15.1 Steering Committee

The steering committee is responsible for overseeing the good execution and administrative progress of the protocol. The committee meets regularly to monitor patient accrual, non-compliance with the protocol at individual centres, to act upon recommendations of the Data Safety Monitoring Board, and to determine the policy regarding individual publications arising from data generated from the performance of the trial.

## 15.2 Data Safety Monitoring Board

The Data Safety Monitoring Board (DSMB) is responsible for assessing the progress, safety data, integrity and critical efficacy endpoints of the trial. The main task of the DSMB is to monitor the safety of the participants of the clinical trial. Therefore the DSMB is provided with the AEs and SAEs that were reported during the conduct of the trial. Based on its review the DSMB provides the sponsor with recommendations regarding trial modification, continuation or termination. All final decisions, however, regarding trial termination or modification rest with the Steering Committee.

The sponsor decides on the selection of the DSMB members. The DSMB members are independent from the sponsor and competing interests. Detailed processes are described in the DSMB Charter.

## 15.3 Event Adjudication Committee

The Event Adjudication Committee (EAC) will adjudicate suspected endpoints in order to provide a standardized, systematic, independent and unbiased assessment. The members will review the events reported by the investigators as endpoints to determine whether they meet the specified criteria of endpoint definition. The additional endpoint bleeding events BARC type ≥ 1 at 6 weeks are not subject of EAC adjudication. During event adjudication, members of the committee are blinded to the randomized treatment received by the patient. More details are outlined in the EAC charter.

The Principal Investigator provides all source documents relevant for the adjudication to the sponsor, e.g. medical history, laboratory test results, treatment and diagnostic reports, admission / discharge reports, autopsy reports, if applicable. All patient related data are transmitted as strictly pseudonymised data.

# 16 Data Handling and Record Keeping

## 16.1 Case Report Forms

Site investigators or authorized study personnel enter the required data for this study into pass-word protected, web-based electronic case report forms (eCRFs). The eCRF contains the patients` data and examination results that are needed to present the study results and to perform protocol-specified data analyses. It must be ensured that the eCRFs only contain pseudonymised data. Any change or correction to eCRF will be documented in the audit trail.

Detailed instructions for the use of the eCRFs are provided in the eCRF User Guide. Inconsistencies in eCRFs are addressed to the investigator by electronic queries and that the site shall solve in a timely manner, adapt the patient data accordingly and that the changes are approved by electronic signature.

All personnel with access to the electronic data capture (EDC) system are supported by a user helpdesk staffed with trained personnel to answer questions and ensure access is maintained such that data entry can proceed in a timely manner.

Investigators ensure that all eCRF forms are filled in a timely fashion as stated below and according to GCP-ICH guidelines. The investigator also ensures the accuracy, completeness, legibility, and timeliness of the data reported to the sponsor in the eCRFs and in all required reports. Data reported in the eCRF should be consistent with the source documents or the discrepancies should be explained. The entered data of the electronic database have to be approved by electronic signature of the investigator.

## 16.2 Source Data

Source documents are original records in which raw data are first recorded. These may be: hospital/clinic/general practitioner records, charts, diaries, x-rays, laboratory results, ECG, and other printouts, pharmacy records, care records, completed psychometric scales, daily record cards, quality of life questionnaires, etc. All source documents must be accurate, clear, unambiguous, permanent and capable of being audited. They should be made using a permanent form of recording (e.g. ink, typing, printing, optical disc).

Hospital/clinic/medical files that are computer generated and stored on magnetic support media may be required to be printed and signed and dated by the investigator.

All information on CRFs must be traceable to these source documents in the patient's file. The investigator must also keep the original Informed Consent Form signed by the patient (a second copy is given to the patient).

The site must implement processes to ensure availability of all required source documentation. A source document list will be used at the site to identify the source data for data points collected and the monitor will work with the site to complete this.

The investigator must give the monitor access to all relevant source documents to confirm their consistency with the CRF entries.

## 16.3 Data Management

### 16.3.1 Data Handling and Quality Control

The integrity of the database is the basis of the final statistical analysis. According to the study’s data validation plan (DVP) automatic edit checks will be implemented into the eCRF in order to detect data discrepancies. The monitor, pharmacovigilance management and the data management are allowed to create manual queries in the eCRF. Both automated and manual queries are essential to guarantee data quality. The sites take responsibility to clarify, confirm or change questionable data accordingly. All query answers provided by the sites are reviewed closely by the monitor, pharmacovigilance management or the data management in order to decide whether a query may be considered closed or not and if a re-query is required.

The EDC system contains a system-generated audit trail that captures any changes made to a data field, including who made the change, why the change was made and the date and time it was made. This information is available both at the investigator’s site and at the sponsor’s. Data entries made in the EDC screens are supported by source documents maintained for all subjects enrolled in this study.

Data of screening failures will be recorded at least as source data, as far as the reason for the premature discontinuation is identifiable. For screening failures only in- and exclusion criteria and the trial termination page have to be completed in the eCRF.

All personnel with access to the EDC system are supported by a user helpdesk staffed with trained personnel to answer questions and ensure access is maintained such that data entry can proceed in a timely manner.

Prior to the documentation of the first patient record (FPFV) in the trial data base a finalized and a fully approved Data Management Plan (DMP) has to be in place. This plan will define all study specific data management processes in detail and has to be updated and newly approved once changes in data management processes during the conduct of the study should take effect.

All other details are described in the Data Management Plan.

### 16.3.2 Data Coding

For data coding (e.g. AEs), internationally recognized and accepted dictionaries (e.g. MedDRA Version 24.0 or higher) will be used.

### 16.3.3 Database Lock

The data base lock will only take place in accordance with the applicable SOP for data base lock to ensure that:

- all study data have been entered accordingly
- decisions have been agreed upon on how individual protocol violations are to be dealt with
- written authorisation from the sponsor has been granted

## 16.4 Data Protection and Confidentiality

The investigator must assure that patients´ identities are protected from access by unauthorized parties. No information in source documents about the identity (e.g. name, date of birth, address, and case numbers of the patients) will be disclosed. The investigator keeps a patient identification log relating codes to the names of the patients. Any party with direct access to source data / source documents should take all reasonable precautions within the constraints of the applicable regulatory requirements to maintain confidentiality of patients' identities and sponsor's proprietary information.

## 16.5 Archiving and Data Retention

All records are to be retained by the Principal Investigator for a minimum of 25 years after completion of the trial, i.e. according to the applicable regulations and guidelines. The Principal Investigator will contact the sponsor for authorization prior to destruction of any trial records or in the event of accidental loss or destruction of any trial records.

The sponsor must retain all essential documents (Trial Master File) including the Case Report Form for the duration of at least 25 years after the end of the trial.

## 16.6 Access to Source Data/Documents / Audits and Inspections

The Principal Investigator permits trial-related audits by auditors mandated by the sponsor or his designee and inspections by domestic or foreign regulatory authorities after reasonable notice. The main purposes of an audit or inspection are to confirm that the rights and well-being of the patients enrolled have been protected, and that all data relevant for the evaluation of the investigational product have been processed and reported in compliance with the planned arrangements, ICH GCP and applicable regulatory requirements. The investigator will provide direct access to all trial documents, CRFs, source records and source data. If a regulatory inspection is announced, the Principal Investigator must immediately inform the sponsor and promptly forwards copies of inspection reports to the sponsor.

Representatives of the sponsor must be allowed to visit all trial sites periodically to assess the data quality and integrity.

# 17 Ethical, Regulatory and Administrative Aspects

## 17.1 Good Clinical Practice

The clinical trial is performed in compliance with the protocol, the Declaration of Helsinki (2013), ICH E6 (R2), Directive 2001/20/EC (applicable until transition into CTIS) and Regulation (EU) No 536/2014, as applicable, and applicable local laws and regulations.

Personnel involved in conducting this clinical trial is qualified by education, training, and experience to perform their respective task(s).

## 17.2 Ethics Commission and Regulatory Authorities

It is the responsibility of the sponsor to obtain approval for the trial protocol from the Member States concerned according to applicable regulatory requirements.

During the clinical trial any amendment or modification to the clinical trial protocol must be submitted to the Member States concerned. All updates of the SmPCs will be sent to the Member States concerned.

## 17.3 Informed Consent

Prior to enrolment in the trial, the investigator will inform the patient orally and in writing about scope and purpose, and possible risks / benefits of the trial as well as his/her rights and duties in a lay language. Patients must be informed that their records will be subject to review by monitors, sponsor and regulatory agency representatives. Patients must be assured that they may withdraw from the trial at any time for any reason and receive alternative conventional therapy as indicated.

By signing and dating the consent form, the patient attests that the information in the consent form and any other written information was accurately explained and apparently understood, and that the patient freely gave his/her consent. In addition, the Investigator who obtains the consent must sign and date the consent form. Failure to obtain such a signed form renders the subject ineligible for trial.

An originally signed copy of the form must be kept on the patient’s file; a second copy will be handed over to the trial participant. This is documented in the patient’s file.

## 17.4 Further Reporting Obligations

### 17.4.1 Serious Breaches

A ‘serious breach’ means a breach likely to affect to a significant degree the safety and rights of a subject or the reliability and robustness of the data generated in the clinical trial.

The investigator has to immediately report any events that might meet the definition of a serious breach to the sponsor.

The sponsor will notify the Member States concerned about a serious breach of the Regulation (EU) No 536/2014 or of the version of the protocol applicable at the time of the breach through the EU portal without undue delay but not later than seven days of becoming aware of that breach.

### 17.4.2 Other Reporting Obligations relevant for Subject Safety

Unexpected events: The sponsor will notify the Member States concerned through the EU portal of all unexpected events which affect the benefit-risk balance of the clinical trial, but are not suspected unexpected serious adverse reactions as referred to in Article 42 of Regulation (EU) No 536/2014. That notification will be made without undue delay but no later than 15 days from the date the sponsor became aware of this event.

The sponsor will submit to the Member States concerned, through the EU portal, all inspection reports of third country authorities concerning the clinical trial.

### 17.4.3 Urgent Safety Measures

Where an unexpected event is likely to seriously affect the benefit-risk balance, the sponsor and the investigator shall take appropriate urgent safety measures to protect the subjects.

The sponsor will notify the Member States concerned, through the EU portal, of the event and the measures taken. That notification will be made without undue delay but no later than seven days from the date the measures have been taken.

### 17.4.4 Start, End, Temporary Halt, and Early Termination of a Clinical Trial

Start, first visit of the first subject in each Member State, end of the recruitment, end of the clinical trial, temporary halt for reasons not affecting the benefit-risk balance, restart and early termination of the clinical trial for reasons not affecting the benefit-risk balance will be reported to the Member States concerned through the EU portal within 15 days (Regulation (EU) No 536/2014 Articles 36, 37, 38).

# 18 Financing

The clinical trial is financed by the Daiichi-Sankyo Deutschland GmbH.

# 19 Insurance

The sponsor provides an insurance for the patients for the total duration of the trial for compensation for any damage suffered by the patient resulting from participation in the clinical trial. The investigator provides the patient with the term and conditions and the certificate of insurance. In case of injury or disability deriving from participation in the study, the patient is requested to inform without delay the insurance company and the treating physician responsible for the trial.

Germany:

HDI-Gerling Industrie Versicherung AG

Niederlassung München

Ganghoferstr. 37 - 39

80339 München, Deutschland

Austria:

AXA XL

Tuchlauben 3

1010 Wien, Österreich

# 20 Submission of the Results and Publication Policy

Prior to start of recruitment the trial must be registered in the publicly accessible database "clinicaltrials.gov".

Irrespective of the outcome of the clinical trial, within one year from the end of the clinical trial in all Member States concerned, the sponsor will submit to the EU database a summary of the results of the clinical trial, including a summary understandable to laypersons.

Furthermore, publications in peer-reviewed medical journals are planned. The information generated by this trial is the property of the sponsor. It is agreed that the results of the trial will not be submitted for presentation, abstract, poster exhibition or publication until the sponsor has reviewed and commented on such a presentation or publication manuscript. The sponsor does not hold the overall right to veto publications and presentations. The sponsor of the study is committed to the unrestricted and widespread dissemination of the results of the study. Upon completion of the study, an abstract reporting the primary results will be prepared by the Steering Committee and presented at an annual scientific cardiology meeting. A manuscript will similarly be prepared for publication.

The publication of the principal results from any single-centre experience within the trial is not allowed before both the preparation and publication of the primary results.

Following analysis and presentation of the primary endpoint results, active participation of all committee members, investigators from high enrolling sites, and core laboratory personnel will be solicited for data analysis, abstract and manuscript preparation.

# 21 References

1. Valgimigli M, Bueno H, Byrne RA et al. 2017 ESC focused update on dual antiplatelet therapy in coronary artery disease developed in collaboration with EACTS: The Task Force for dual antiplatelet therapy in coronary artery disease of the European Society of Cardiology (ESC) and of the European Association for Cardio-Thoracic Surgery (EACTS). European Heart Journal 2017;39:213-260.
2. Neumann F-J, Sousa-Uva M, Ahlsson A et al. 2018 ESC/EACTS Guidelines on myocardial revascularization. European Heart Journal 2018;40:87-165.
3. Vranckx P, Valgimigli M, Eckardt L et al. Edoxaban-based versus vitamin K antagonist-based antithrombotic regimen after successful coronary stenting in patients with atrial fibrillation (ENTRUST-AF PCI): a randomised, open-label, phase 3b trial. Lancet 2019;394:1335-1343.
4. Lopes RD, Heizer G, Aronson R et al. Antithrombotic Therapy after Acute Coronary Syndrome or PCI in Atrial Fibrillation. N Engl J Med 2019;380:1509-1524.
5. Cannon CP, Bhatt DL, Oldgren J et al. Dual Antithrombotic Therapy with Dabigatran after PCI in Atrial Fibrillation. N Engl J Med 2017;377:1513-1524.
6. Gibson CM, Mehran R, Bode C et al. Prevention of Bleeding in Patients with Atrial Fibrillation Undergoing PCI. N Engl J Med 2016;375:2423-2434.
7. Sibbing D, Rizas K, Massberg S. Spoilt for choice – P2Y12-receptor inhibition in patients with atrial fibrillation undergoing percutaneous coronary intervention. JACC Interventions in press 2024.
8. Windecker S, Lopes RD, Massaro T et al. Antithrombotic Therapy in Patients With Atrial Fibrillation and Acute Coronary Syndrome Treated Medically or With Percutaneous Coronary Intervention or Undergoing Elective Percutaneous Coronary Intervention. Circulation 2019;140:1921-1932.
9. Lopes RD, Leonardi S, Wojdyla DM et al. Stent Thrombosis in Patients With Atrial Fibrillation Undergoing Coronary Stenting in the AUGUSTUS Trial. Circulation 2020;141:781-783.
10. Collet JP, Thiele H, Barbato E et al. 2020 ESC Guidelines for the management of acute coronary syndromes in patients presenting without persistent ST-segment elevation. Eur Heart J 2020.
11. Gargiulo G, Goette A, Tijssen J et al. Safety and efficacy outcomes of double vs. triple antithrombotic therapy in patients with atrial fibrillation following percutaneous coronary intervention: a systematic review and meta-analysis of non-vitamin K antagonist oral anticoagulant-based randomized clinical trials. Eur Heart J 2019;40:3757-3767.
12. Sibbing D, Aradi D, Jacobshagen C et al. Guided de-escalation of antiplatelet treatment in patients with acute coronary syndrome undergoing percutaneous coronary intervention (TROPICAL-ACS): a randomised, open-label, multicentre trial. Lancet 2017;390:1747-1757.
13. Antman EM, Wiviott SD, Murphy SA et al. Early and late benefits of prasugrel in patients with acute coronary syndromes undergoing percutaneous coronary intervention: a TRITON-TIMI 38 (TRial to Assess Improvement in Therapeutic Outcomes by Optimizing Platelet InhibitioN with Prasugrel-Thrombolysis In Myocardial Infarction) analysis. J Am Coll Cardiol 2008;51:2028-33.
14. Godtfredsen SJ, Kragholm KH, Kristensen AMD et al. Ticagrelor or prasugrel vs. clopidogrel in patients with atrial fibrillation undergoing percutaneous coronary intervention for myocardial infarction. Eur Heart J Open 2024;4:oead134.

15. Byrne RA, Rossello X, Coughlan JJ et al. 2023 ESC Guidelines for the management of acute coronary syndromes. Eur Heart J 2023;44:3720-3826.
16. Angiolillo DJ, Rollini F, Storey RF et al. International Expert Consensus on Switching Platelet P2Y(12) Receptor-Inhibiting Therapies. Circulation 2017;136:1955-1975.
17. Fontaine R, Kim M, Kieny R. [Surgical treatment of peripheral circulation disorders]. Helvetica chirurgica acta 1954;21:499-533.
18. Finkelstein DM, Schoenfeld DA. Combining mortality and longitudinal measures in clinical trials. Stat Med 1999;18:1341-54.
19. Wiviott SD, Trenk D, Frelinger AL et al. Prasugrel compared with high loading- and maintenance-dose clopidogrel in patients with planned percutaneous coronary intervention: the Prasugrel in Comparison to Clopidogrel for Inhibition of Platelet Activation and Aggregation-Thrombolysis in Myocardial Infarction 44 trial. Circulation 2007;116:2923-32.
20. Pocock SJ, Gregson J, Collier TJ, Ferreira JP, Stone GW. The win ratio in cardiology trials: lessons learnt, new developments, and wise future use. Eur Heart J 2024;45:4684-4699.
21. Pocock SJ, Ariti CA, Collier TJ, Wang D. The win ratio: a new approach to the analysis of composite endpoints in clinical trials based on clinical priorities. Eur Heart J 2012;33:176-82.
22. Mehran R, Rao SV, Bhatt DL et al. Standardized bleeding definitions for cardiovascular clinical trials: a consensus report from the Bleeding Academic Research Consortium. Circulation 2011;123:2736-47.
23. Garcia-Garcia HM, McFadden EP, Farb A et al. Standardized End Point Definitions for Coronary Intervention Trials: The Academic Research Consortium-2 Consensus Document. Circulation 2018;137:2635-2650.
24. Thygesen K, Alpert JS, Jaffe AS et al. Fourth Universal Definition of Myocardial Infarction (2018). Circulation 2018;138:e618-e651.
25. Hicks KA, Mahaffey KW, Mehran R et al. 2017 Cardiovascular and Stroke Endpoint Definitions for Clinical Trials. Circulation 2018;137:961-972.
26. Ibanez B, James S, Agewall S et al. 2017 ESC Guidelines for the management of acute myocardial infarction in patients presenting with ST-segment elevation: The Task Force for the management of acute myocardial infarction in patients presenting with ST-segment elevation of the European Society of Cardiology (ESC). European Heart Journal 2017;39:119-177.
27. Hindricks G, Potpara T, Dagres N et al. 2020 ESC Guidelines for the diagnosis and management of atrial fibrillation developed in collaboration with the European Association for Cardio-Thoracic Surgery (EACTS). Eur Heart J 2021;42:373-498.
28. Bonow RO, Carabello BA, Kanu C et al. ACC/AHA 2006 guidelines for the management of patients with valvular heart disease: a report of the American College of Cardiology/American Heart Association Task Force on Practice Guidelines (writing committee to revise the 1998 Guidelines for the Management of Patients With Valvular Heart Disease): developed in collaboration with the Society of Cardiovascular Anesthesiologists: endorsed by the Society for Cardiovascular Angiography and Interventions and the Society of Thoracic Surgeons. Circulation 2006;114:e84-231.
29. Campeau L. Letter: Grading of angina pectoris. Circulation 1976;54:522-3.
30. Pisters R, Lane DA, Nieuwlaat R, de Vos CB, Crijns HJ, Lip GY. A novel user-friendly score (HAS-BLED) to assess 1-year risk of major bleeding in patients with atrial fibrillation: the Euro Heart Survey. Chest 2010;138:1093-100.

# 22 Appendices

## 22.1 Appendix 1: Endpoint Definitions

**BLEEDING:**

These include all bleeding complications occurring after randomization of the patient. Bleeding localization, amount of haemoglobin drop (if available) and the number of blood units transfused must be recorded in the Case Report Form. Bleeding Academic Research Consortium Definition for Bleeding (22).

| | |
|---|---|
| Type 0: | No bleeding |
| Type 1: | Bleeding that is not actionable and does not cause the patient to seek unscheduled performance of studies, hospitalization, or treatment by a healthcare professional; may include episodes leading to self-discontinuation of medical therapy by the patient without consulting a healthcare professional |
| Type 2: | Any overt, actionable sign of haemorrhage (e.g., more bleeding than would be expected for a clinical circumstance, including bleeding found by imaging alone) that does not fit the criteria for type 3, 4, or 5 but does meet at least one of the following criteria: (1) requiring nonsurgical, medical intervention by a healthcare professional, (2) leading to hospitalization or increased level of care, or (3) prompting evaluation |
| Type 3: | |
| Type 3a: | Overt bleeding plus a haemoglobin drop of 3 to <5 g/dL* (provided haemoglobin drop is related to bleed) |
| | Any transfusion with overt bleeding |
| Type 3b: | Overt bleeding plus a haemoglobin drop ≥5 g/dL* (provided haemoglobin drop is related to bleed) |
| | Cardiac tamponade |
| | Bleeding requiring surgical intervention for control (excluding dental, nasal, skin or haemorrhoid) |
| | Bleeding requiring intravenous vasoactive agents |
| Type 3c: | Intracranial haemorrhage (does not include microbleeds or haemorrhagic transformation, does include intraspinal) |
| | Subcategories confirmed by autopsy or imaging or lumbar puncture |
| | Intraocular bleed compromising vision |
| Type 4: | CABG-related bleeding: |

Perioperative intracranial bleeding within 48 h

Reoperation after closure of sternotomy for the purpose of controlling bleeding

Transfusion of ≥5 U whole blood or packed red blood cells within a 48-h period†

Chest tube output ≥2L within a 24-h period

Type 5: Fatal bleeding

Type 5a Probable fatal bleeding; no autopsy or imaging confirmation but clinically suspicious

Type 5b Definite fatal bleeding; overt bleeding or autopsy or imaging confirmation

Platelet transfusions should be recorded and reported but are not included in these definitions until further information is obtained about the relationship to outcomes.

If a CABG-related bleed is not adjudicated as at least a type 3 severity event, it will be classified as not a bleeding event.

If a bleeding event occurs with a clear temporal relationship to CABG (ie, within a 48-h time frame) but does not meet type 4 severity criteria, it will be classified as not a bleeding event.

* Corrected for transfusion (1 U packed red blood cells or 1 U whole blood =1 g/dL haemoglobin).

† Cell saver products are not counted.

**DEATH** (ARC-2 criteria) (23)

Cardiovascular death:

Is defined as death resulting from cardiovascular causes. The following categories may be collected:

- Death caused by acute MI
- Death caused by sudden cardiac, including unwitnessed, death
- Death resulting from heart failure
- Death caused by stroke
- Death caused by cardiovascular procedures
- Death resulting from cardiovascular haemorrhage
- Death resulting from other cardiovascular cause

Non-cardiovascular death:

Is defined as any death that is not thought to be the result of a cardiovascular cause. The following categories may be collected:

- Death resulting from malignancy
- Death resulting from pulmonary causes
- Death caused by infection (includes sepsis)
- Death resulting from gastrointestinal causes
- Death resulting from accident/trauma
- Death caused by other noncardiovascular organ failure
- Death resulting from other noncardiovascular cause

Undetermined death:

Is defined as a death not attributable to any other category because of the absence of any relevant source documents. Such deaths will be clarified as cardiovascular for end point determination.

**MYOCARDIAL INFARCTION**

The definition of myocardial infarction used in this trial is adapted from the Fourth Universal Definition of Myocardial Infarction (24). The term acute myocardial infarction should be used when there is acute myocardial injury with clinical evidence of acute myocardial ischaemia and with detection of a rise and/or fall of cTn values with at least one value above the 99th percentile. High-sensitivity (hs)-cTn assays are recommended as preferred biomarkers.CK-MB (and CK) values will be assessed concurrently and used in the case that troponin values are not available.

**Myocardial injury**

The term myocardial injury should be used when there is evidence of elevated cardiac troponin values (cTn) with at least 1 value above the $99^{th}$ percentile upper reference limit (URL). The myocardial injury is considered acute if there is a rise and/or fall of cTn values.

**Myocardial Infarction Type 1:**

Detection of a rise and/or fall of cTn values with at least 1 value above the 99th percentile URL and with at least 1 of the following:

- Symptoms of acute myocardial ischaemia
- New ischaemic ECG changes

- Development of pathological Q waves on ECG
- Imaging evidence of new loss of viable myocardium or new regional wall motion abnormality in a pattern consistent with an ischaemic etiology.
- Intracoronary thrombus detected on angiography or by autopsy*

Postmortem demonstration of an atherothrombus in the artery supplying the infarcted myocardium, or a macroscopically large circumscribed area of necrosis with or without intra-myocardial haemorrhage, meets the type 1 MI criteria regardless of cTn values.

**Myocardial Infarction Type 2:**

Detection of a rise and/or fall of cTn values with at least 1 value above the 99th percentile URL, and evidence of an imbalance between myocardial oxygen supply and demand unrelated to acute coronary atherothrombosis, requiring at least 1 of the following:

- Symptoms of acute myocardial ischaemia
- New ischaemic ECG changes
- Development of pathological Q waves
- Imaging evidence of new loss of viable myocardium or new regional wall motion abnormality in a pattern consistent with an ischaemic etiology

**Myocardial Infarction Type 3:**

Patients who suffer cardiac death, with symptoms suggestive of myocardial ischaemia accompanied by presumed new ischaemic ECG changes or ventricular fibrillation, but die before blood samples for biomarkers can be obtained, or before increases in cardiac biomarkers can be identified, or MI is detected by autopsy examination.

**Myocardial Infarction Type 4a (PCI-Related MI ≤48 after the index procedure):**

Coronary intervention-related MI is arbitrarily defined by an elevation of cTn values >5 times the 99th percentile URL in patients with normal baseline values. In patients with elevated preprocedure cTn in whom the cTn level are stable (≤20% variation) or falling, the postprocedure cTn must rise by >20%. However, the absolute postprocedural value must still be at least 5 times the 99th percentile URL. In addition, 1 of the following elements is required:

- New ischaemic ECG changes
- Development of new pathological Q waves*
- Imaging evidence of new loss of viable myocardium or new regional wall motion abnormalities in a pattern consistent with an ischaemic etiology

- Angiographic findings consistent with a procedural flow-limiting complication such as coronary Dissection, occlusion of a major epicardial artery or a side branch occlusion/thrombus, disruption of collateral flow, or distal embolization†

*Isolated development of new pathological Q waves meets the type 4a MI criteria if cTn values are elevated and rising but <5 times the 99th percentile URL.

†Postmortem demonstration of a procedure-related thrombus in the culprit artery, or a macroscopically large circumscribed area of necrosis with or without intra-myocardial haemorrhage meets the type 4a MI criteria.

**Myocardial Infarction Type 4b (Stent thrombosis associated with PCI):**

Is documented by angiography or autopsy using the same criteria utilized for type 1 MI. It is important to indicate the time of the occurrence of the stent/scaffold thrombosis in relation to the timing of the PCI procedure. The following temporal categories are suggested:

- Acute 0-24 hours
- Subacute >24 hours to 30 days
- Late >30 days to 1 year
- Very late >1 year after stent/scaffold implantation

**Myocardial Infarction Type 4c (Restenosis associated with PCI):**

Defined as focal or diffuse restenosis, or a complex lesion associated with a rise and/or fall of cTn values above the 99th percentile URL applying, the same criteria utilized for type 1MI.

**Myocardial Infarction Type 5 (CABG-Related MI ≤48 after the index procedure):**

CABG-related MI is arbitrarily defined as elevation of cTn values > 10 times the 99th percentile URL in patients with normal baseline cTn values. In patients with elevated preprocedure CTn in whom cTn levels are stable (≤20% variation) or falling, the postprocedure cTn must rise by >20%. However, the absolute postprocedural value still must be > 10 times the 99th percentile URL. In addition, 1 of the following elements is required:

- Development of new pathological Q waves*
- Angiographic documented new graft occlusion or new native coronary artery occlusion
- Imaging evidence of new loss of viable myocardium or new regional wall motion abnormality in a pattern consistent with an ischaemic etiology.

*Isolated development of new pathological Q waves meets the type 5 MI criteria if cTn values are elevated and rising but <10 times the 99th percentile URL.

**Q-wave vs. Non-Q-wave Myocardial infarction:**

Q-wave MI will be diagnosed if new pathologic Q-waves (≥25% of the height of the partner R wave and/or ≥0.04 sec in duration) in ≥2 contiguous ECG leads occur. All other MIs not fulfilling the above mentioned criteria will be considered non-Q-wave MI.

## STENT THROMBOSIS

Stent thrombosis will be classified according to the Academic Research Consortium -2 (23):

**Definite Stent/Scaffold Thrombosis:**

Is considered to have occurred by either angiographic or pathological confirmation.

The angiographic confirmation includes the presence of a thrombus that originates in the stent/scaffold or in the segment 5 mm proximal or distal to the stent or in a side branch originating from the stented segment and the presence of at least 1 of the following criteria:

- Acute onset of ischemic symptoms at rest
- New ECG changes suggestive of acute ischaemia
- Typical rise and fall in cardiac biomarkers

The pathological confirmation of stent/scaffold thrombosis includes:

- Evidence of recent thrombus within the stent determined at autopsy or
- Examination of tissue retrieved following thrombectomy (visual/histology)

**Probable Stent/Scaffold Thrombosis:**

Regardless of the time after the index procedure, any myocardial infarction that is related to documented acute ischaemia in the territory of the implanted stent/scaffold without angiographic confirmation of stent thrombosis and in the absence of any other obvious cause.‡

‡ When the stented/scaffolded segment is in the left circumflex coronary artery or in the presence of preexisting electrocardiographic abnormalities (eg, left bundle branch block, paced rhythms), definitive evidence of localization may be absent and Clinical Events Committee adjudication is based on review of all available evidence.

**Silent stent occlusion:**

The incidental angiographic documentation of stent occlusion in the absence of clinical signs or symptoms is not considered stent thrombosis.

Timing of Stent thrombosis:

| | |
|---|---|
| Acute | 0§-24 h |
| Subacute | >24 h-30 d |
| Late | >30 d-1y |
| Very late | >1 y |

§ Defined as the moment the patient is undraped and taken off the catheterization table.

## STROKE

Stroke is defined as an acute episode of focal or global neurological dysfunction of at least 24 hours caused by brain, spinal cord, or retinal vascular injury as a result of haemorrhage or infarction.

Diagnosis of stroke should be confirmed by cCT or MRI or pathology (25).

**Classification:**

Ischaemic stroke:

Ischaemic stroke is defined as an acute episode of focal cerebral, spinal, or retinal dysfunction caused by infarction of central nervous system tissue. Haemorrhage may be a consequence of ischaemic stroke. In this situation, the stroke is an ischemic stroke with haemorrhagic transformation and not a haemorrhagic stroke.

Haemorrhagic stroke:

Haemorrhagic stroke is defined as an acute episode of focal or global cerebral or spinal dysfunction caused by intraparenchymal, intraventricular, or subarachnoid haemorrhage.

Undetermined stroke:

Undetermined stroke is defined as an acute episode of focal or global neurological dysfunction caused by presumed brain, spinal cord, or retinal vascular injury as a result of haemorrhage or infarction but with insufficient information to allow categorization as either ischaemic or haemorrhagic.

**Modified Rankin Scale - Stroke severity assessment scale**

| | |
|---|---|
| Scale 0 | No symptoms at all |
| Scale 1 | No significant disability despite symptoms: able to carry out all usual duties and activities |
| Scale 2 | Slight disability: unable to carry out all previous activities but able to look after own affairs without assistance |
| Scale 3 | Moderate disability: requiring some help, but able to walk without assistance |
| Scale 4 | Moderately severe disability: unable to walk without assistance, and unable to attend to own bodily needs without assistance |
| Scale 5 | Severe disability: bedridden, incontinent and requiring constant nursing care and attention |
| Scale 6 | Death |

## SYSTEMIC THROMBOEMBOLISM

A systemic embolic event is defined as an arterial embolism resulting in clinical ischemia, excluding the central nervous system, coronary and pulmonary arterial circulation (23).

## URGENT REVASCULARIZATION

Any PCI or bypass surgery performed for recurrent ischaemia that in the investigator's opinion cannot be delayed for more than 24 hours and is defined by the investigator as a non-elective.

**Target Lesion Revascularization:**

Target lesion revascularization is defined as a repeat percutaneous intervention of the target lesion (including 5 mm proximal and distal to the stent/scaffold) or bypass surgery of the target vessel performed for restenosis or other complication of the target lesion.

**Target Vessel Revascularization:**

Target vessel revascularization is defined as any repeat percutaneous intervention or surgical bypass of any segment of the target vessel including the target lesion. The target vessel consists of target lesion(s) and any additional lesions in the main epicardial coronary artery or branches containing the target lesion.

## NYHA CLASSIFICATION OF HEART FAILURE

| | |
|---|---|
| Class I | No limitation of activities; patients suffer no symptoms from ordinary activities. |
| Class II | Slight, mild limitation of activity; patients are comfortable with rest or with mild exertion. |
| Class III | Marked limitation of activity; patients are comfortable only at rest. |
| Class IV | Patients who should be at complete rest, confined to bed or chair; any physical activity brings on discomfort and symptoms occur at rest. |

## **ACUTE CORONARY SYNDROME** (10,26)

### ST-segment elevation myocardial infarction (STEMI)

Is defined in patients with persistent chest discomfort or other symptoms suggestive of ischemia and persistent (>20 min) ST-segment elevation in at least two contiguous ECG leads.

### Non-ST-segment elevation myocardial infarction (NSTEMI)

Is defined in patients with acute chest discomfort or chest pain-equivalent symptoms and increased cardiac troponins above the $99^{th}$ percentile of healthy individuals (conventional or high-sensitive assays) but no persistent ST-segment elevation ECG changes.

### Unstable Angina

Is defined in patients with myocardial ischaemia at rest or on minimal exertion in the absence of acute cardiomyocyte injury/necrosis (cardiac troponins within the $99^{th}$ percentile of healthy individuals) but without persistent ST-segment elevation on ECG.

### Biomarker positive acute coronary syndrome

STEMI or NSTEMI

## 22.2 Appendix 2: Definition of Clinical and Angiographic Characteristics

**ATRIAL FIBRILLATION**

Is defined as a supraventricular tachyarrhythmia with uncoordinated atrial electrical activation and consequently ineffective atrial contraction (27). Electrocardiographic characteristics of AF include:

- Irregularly irregular R-R intervals (when atrioventricular conduction is not impaired),
- Absence of distinct repeating P waves, and
- Irregular atrial activations.

**Classification of AF**

| AF pattern | Definition |
|---|---|
| First diagnosed | AF not diagnosed before, irrespective of its duration or the presence/severity of AF-related symptoms. |
| Paroxysmal | AF that terminates spontaneously or with intervention within 7 days of onset. |
| Persistent | AF that is continuously sustained beyond 7 days, including episodes terminated by cardioversion (drugs or electrical cardioversion) after ≥7 days |
| Long-standing persistent | Continuous AF of >12 months' duration when decided to adopt a rhythm control strategy |
| Permanent | AF that is accepted by the patient and physician, and no further attempts to restore/maintain sinus rhythm will be undertaken. Permanent AF represents a therapeutic attitude of the patient and physician rather than an inherent pathophysiological attribute of AF, and the term should not be used in the context of a rhythm control strategy with antiarrhythmic drug therapy or AF ablation. Should a rhythm control strategy be adopted, the arrhythmia would be re-classified as 'long-standing persistent AF'. |
| ,,valvular" AF (old term) | Means patients with moderate/severe mitral stenosis and those with mechanical prosthetic heart valves. |

## COMPLETION OF PCI

Defined by the local trial centre (including stop time as documented on the catheter laboratory report or the time point, when the patient leaves the catheter laboratory).

## MITRAL STENOSIS (28)

### CRITERIA FOR SEVERITY GRADING OF MITRAL STENOSIS

| Grading | Mitral valve area ($cm^2$) | Mean gradient at anormal heart rate (mmHg) |
|---|---|---|
| **Mild** | >1.5 cm2 | <5 |
| **Moderate** | 1.0–1.5 | 5–10 |
| **Severe** | ≤1.0 | >10 |

## SUCCESSFUL PCI

Defined as TIMI flow grade 2 or more.

## CANADIAN CARDIOVASCULAR SOCIETY (CCS) CLASSIFICATION OF ANGINA PECTORIS (29)

Class I: Ordinary physical activity (such as walking and climbing stairs) does not cause angina. Angina with strenuous or rapid or prolonged exertion at work or recreation.

Class II: Slight limitation of ordinary activity. Angina upon walking or climbing stairs rapidly, walking uphill, walking or stair climbing after meals, or in the cold, in wind or under emotional stress, or only during the few hours after awakening. Angina if walking more than two blocks on a level and climbing more than one flight of ordinary stairs at a normal pace and in normal conditions.

Class III: Marked limitation of ordinary physical activity. Walking one to two blocks on a level and climbing one flight of stairs in normal conditions and at a normal pace.

Class IV: Inability to carry on any physical activity without discomfort. Anginal syndrome may be present at rest.

## CARDIOGENIC SHOCK

Must fulfil one of the following criteria:

- Either systolic blood pressure < 90 mmHg for at least 30 minutes or
- Need for supportive measures to maintain a systolic blood pressure > 90 mmHg

Plus

- Either signs of impaired end-organ hypoperfusion (altered mental status, cold, clammy skin or extremities, oliguria with urine output <30mL/h or serum lactate >2.0 mmol/L) or
- Haemodynamic criteria of cardiac index < 2.2 L/min per square meter body surface area and a pulmonary capillary wedge pressure of at least 15 mmHg

## CHA2DS2Vasc SCORE

CHA2DS2-VASc score is used for stroke risk assessment. The maximum CHA2DS2-VASc score is 9.

| | Condition | Points |
|---|---|---|
| C | **Congestive heart failure** (signs/symptoms of heart failure or objective evidence of reduced left ventricular ejection fraction) | 1 |
| H | **Hypertension** (Resting blood pressure >140/90 mmHg on at least two occasions or current antihypertensive treatment) | 1 |
| $A_2$ | **Age ≥75 years** | 2 |
| D | **Diabetes mellitus** (fasting glucose >125 mg/dL (7mmol/L) or treatment with oral hypoglycaemic agent and/or insulin) | 1 |
| $S_2$ | **Prior stroke, transient ischaemic attack or thromboembolism** | 2 |
| V | **Vascular disease** (previous myocardial infarction, peripheral artery disease or aortic plaque) | 1 |
| A | **Age 65–74 years** | 1 |
| Sc | **Sex category (female)** | 1 |

**HAS-BLED SCORE** (30)

| HAS-BLED | Points |
|---|---|
| Hypertension i.e uncontrolled BP | 1 |
| Abnormal renal function | 1 |
| Abnormal liver function | 1 |
| Stroke | 1 |
| Bleeding tendency or predisposition | 1 |
| Labile INR | 1 |
| Age (e.g >65) | 1 |
| Drugs (e.g. concomitant Aspirin or NSAIDSs) | 1 |
| Alcohol abuse | 1 |

≤2 Points: low to moderate bleeding risk

>2 high bleeding risk

**CHILD-PUGH CLASS OF CHRONIC LIVER DISEASE**

The score employs five clinical measures of liver disease. Each measure is scored 1-3, with 3 indicating most severe derangement. In primary sclerosing cholangitis (PSC) and primary biliary cirrhosis (PBC), the bilirubin references are changed to reflect the fact that these diseases feature high conjugated bilirubin levels. The upper limit for 1 point is 68 µmol/l (4 mg/dl) and the upper limit for 2 points is 170 µmol/l (10 mg/dl).

| Measure | 1 point | 2 points | 3 points |
|---|---|---|---|
| Total bilirubin, µmol/l (mg/dl) | <34 (<2) | 34-50 (2-3) | >50 (>3) |
| Serum albumin, g/l | >35 | 28-35 | <28 |
| INR | <1.7 | 1.71-2.30 | > 2.30 |
| Ascites | None | Mild | Moderate to Severe |
| Hepatic encephalopathy | None | Grade I-II<br>(or suppressed with medication) | Grade III-IV<br>(or refractory) |

Interpretation:

Chronic liver disease is classified into Child-Pugh class A to C, employing the added score from above.

Class A: 5-6 Points

Class B: 7-9 Points

Class C: 10-15 Points

**DIABETES MELLITUS:**

Active treatment with insulin or an oral hypoglycaemic agent on admission

Patients diagnosed with diabetes who are on dietary therapy alone, documentation of an abnormal fasting blood glucose or glucose tolerance test based on the World Health Organization criteria is required.

**FAMILY HISTORY OF PREMATURE CAD**

Myocardial infarction, or sudden abrupt death without obvious cause, before the age of 55 in a first-degree blood male relative (parent, sibling, or children related by blood) or before the age of 65 in a first-degree blood female relative.

**HISTORY OF HYPERCHOLESTEROLEMIA**

Patients with any one of the following:

1. Prior total cholesterol > 200 mg/dl
2. Prior treatment with a lipid lowering agent for hypercholesterolemia

**ARTERIAL HYPERTENSION**

Arterial hypertension is considered to be present when a person's systolic blood pressure is 140 mmHg or greater, and/or their diastolic blood pressure is 90 mmHg or greater on 2 different occasions, or active treatment with antihypertensive drugs.

## MULTIVESSEL DISEASE

Presence of angiographically significant lesions (>50% lumen narrowing) in ≥ 2 major epicardial coronary arteries.

## SMOKING STATUS

| | |
|---|---|
| Smoker: | any cigarette smoking in the prior 6 months |
| Non-smoker: | no regularly cigarettes smoking at any time (according to the World Health Organization also former smoker that quit smoking for at least 10 years) |
| Former smoker: | those who had quit smoking at least 6 months ago |

## PERIPHERAL ARTERIAL DISEASE (17)

### FONTAINE CLASSIFICATION OF PAD

| | |
|---|---|
| **Stage I** | Asymptomatic PAD |
| **Stage II** | Pain on exertion<br>• IIa: claudication at a walking distance >200 m<br>• IIb: claudication at a walking distance < 200 m |
| **Stage III** | Ischemic pain at rest |
| **Stage IV** | Necrosis, ulcers or gangrene of the limb |

## TIMI GRADE FLOW

A grading system for coronary flow:

| | |
|---|---|
| 0 | No perfusion: No ante grade flow beyond the point of occlusion. |
| 1 | Penetration without perfusion: The contrast material passes beyond the area of obstruction, but “hangs up” and fails to opacify the entire coronary bed distal to the obstruction for the duration of the cine run. |
| 2 | Partial reperfusion: The contrast material passes across the obstruction and opacifies the coronary bed distal to the obstruction. However, the rate of entry of contrast into the vessel distal to the obstruction and/or its rate of clearance from the distal bed are perceptibly slower than its entry into and/or clearance from comparable areas not |

| | |
|---|---|
| | perfused by the culprit vessel (e.g., the opposite coronary artery or coronary bed proximal to the obstruction). |
| 3 | Complete perfusion: Antegrade flow into the bed distal to the obstruction occurs as promptly as into the bed proximal to the obstruction and clearance of contrast material from the involved bed is as rapid as from an uninvolved bed in the same vessel or the opposite artery |

## CHRONIC OBSTRUCTIVE PULMONARY DISEASE (COPD)

Severity of Airflow Limitation Assessment in Patients with COPD

| SEVERITY OF AIRFLOW LIMITATION | CLASSIFICATION | $FEV_1$* |
|---|---|---|
| MILD | GOLD I | ≥80% |
| MODERATE | GOLD II | 50%-79% |
| SEVERE | GOLD III | 30%-49% |
| VERY SEVERE | GOLD IV | <30% |

$FEV_1$ indicates forced expiratory volume in 1 second; FVC, forced vital capacity; GOLD, Global Initiative for Chronic Obstructive Lung Disease.

*Assessed in patients with $FEV_1$/FVC less than 0.70

## CHRONIC KIDNEY DISEASE

### GFR CATEGORIES IN CHRONIC KIDNEY DISEASE

| Category | GFR ml/min/1.73 m2 | Renal function |
|---|---|---|
| Grade 1 | ≥90 | Normal or high |
| Grade 2 | 60-89 | Mildly decreased |

| | | |
|---|---|---|
| **Grade 3a** | 45-59 | Mildly to moderately decreased |
| **Grade 3b** | 30-44 | Moderately to severely decreased |
| **Grade 4** | 15-29 | Severely decreased |
| **Grade 5** | <15 | Kidney failure |

**Transient Ischemic Neurological Attack:**

Transient ischemic attack (TIA) is defined as a transient episode of focal neurological dysfunction caused by brain, spinal cord, or retinal ischaemia, without acute infarction.

**Thrombocytopenia:**

Decrease in platelet count ≤100 x $10^9$ cells/L and a fall more than 25% from baseline and exclusion of pseudo thrombocytopenia. The severity of thrombocytopenia and the number of platelet units transfused must be recorded. Also, the date and time in which the nadir of platelet count develops must be recorded. A decrease in platelet count <20 x $10^9$ cells/L is considered profound thrombocytopenia.

Pseudo thrombocytopenia reflects erroneous reading by automated counters because of excessive platelet clumping in the presence of EDTA. A repeat platelet count should be obtained with sodium citrate as the anticoagulant, and/or a blood smear should be examined.

Heparin-induced thrombocytopenia is diagnosed in cases meeting the criteria of Pouplard and colleagues: heparin-dependent platelet-reactive antibodies in the C-serotonin release assay, or finding of increased levels of anti-heparin/PF4 antibodies by ELISA assay.

LMU KLINIKUM

# Table of Changes Version 1.0 to Version 2.0 (refers to the version with track changes)

(new text in red)

| Section, page (track change) | Description of Change |
|---|---|
| 5.2, page 24 | Old text:<br>After randomization, Aspirin will be continued at a maintenance dose of 75-100mg for the in-hospital stay for a maximum of 7-days in the control group and will be immediately discontinued in the intervention group.<br>New text:<br>After randomization, Aspirin will be continued at a maintenance dose of 75-100mg (for Germany and Austria only 100 mg are applicable) for the in-hospital stay for a maximum of 7-days in the control group and will be immediately discontinued in the intervention group.<br>Reason:<br>A dose of 75 mg is not relevant in Germany and Austria. The dose of 75 mg is according to the guidelines and may be used in other European countries, where the trial eventually will be conducted. |
| 9.1, page 30, IMP 1 | Old text:<br>All other Prasugrel formulations with a marketing authorization in the European Union could be used.<br>New text:<br>All other Prasugrel formulations with a marketing authorization in Germany or in the European Union could be used. |
| 9.1, page 31, IMP 3 | Old text:<br>All other Clopidogrel formulations with a marketing authorization in the European Union could be used.<br>New text:<br>All other Clopidogrel formulations with a marketing authorization in Germany or in the European Union could be used. |
| 9.1, page 31, IMP 4 | IMP 4 was added:<br>IMP4: Reference product<br>Germany:<br>Drug name: HerzASS-ratiopharm® 100 mg Tabletten |

| | |
|---|---|
| Name of manufacturer | ratiopharm GmbH |
| Substance name (if applicable, give substance code) | Acetylsalicylic Acid (ATC-Code: B01AC06) |
| Name and dose of active ingredient per unit | Acetylsalicylic Acid 100 mg |
| Other ingredients | Maize starch, micro-crystalline cellulose, cellulose powder |
| Pharmaceutical form | Tablet |
| Mode of administration | oral |
| Batch number (if applicable) | NA |
| Storage conditions | Do not store above 25°C, store in original packaging to protect the content from humidity |

HerzASS-ratiopharm® 100 mg is given as an example. The IMP is only defined by active substance Acetylsalicylic Acid. All other Acetylsalicylic Acid formulations with a marketing authorization in Germany or in the European Union could be used.

Austria:

| | |
|---|---|
| Drug name | HerzschutzASS “ratiopharm” 100 mg - Tabletten |
| Name of manufacturer | TEVA B.V. |
| Substance name (if applicable, give substance code) | Acetylsalicylic Acid (ATC-Code: B01AC06) |
| Name and dose of active ingredient per unit | Acetylsalicylic Acid 100 mg |
| Other ingredients | Maize starch, micro-crystalline cellulose, cellulose powder |
| Pharmaceutical form | Tablet |
| Mode of administration | oral |
| Batch number (if applicable) | NA |
| Storage conditions | Do not store above 30°C. Do not store above 25°C, store in original packaging to protect the content from humidity |

| | |
|---|---|
| | HerzschutzASS “ratiopharm” 100 mg is given as an example. The IMP is only defined by active substance Acetylsalicylic Acid. All other Acetylsalicylic Acid formulations with a marketing authorization in Austria or in the European Union could be used. |
| 10.1.1, page 36 | Old text:<br>After randomization, Aspirin will be continued at a maintenance dose of 75-100mg for the in-hospital stay for a maximum of 7-days in the control group and will be immediately discontinued in the intervention group.<br>New text:<br>After randomization, Aspirin will be continued at a maintenance dose of 75-100mg (for Germany and Austria only 100 mg are applicable) for the in-hospital stay for a maximum of 7-days in the control group and will be immediately discontinued in the intervention group.<br>Reason:<br>A dose of 75 mg is not relevant in Germany and Austria. The dose of 75 mg is according to the guidelines and may be used in other European countries, where the trial eventually will be conducted. |
| 11.1.4, page 38 | Old text:<br>The reference safety information for the IMPs is the effective SmPC of Efient®, Brilique® and Plavix®, respectively.<br>New text:<br>The reference safety information for the IMPs is the effective SmPC of Efient®, Brilique®, Plavix® and HerzASS-ratiopharm® 100 mg (Germany) or HerzschutzASS “ratiopharm” 100 mg (Austria), respectively. |
| 11.2.2, page 39 | Old text:<br>The reference safety information for classification of expected or unexpected SAEs are the current versions of the Summaries of Product Characteristics (SmPC) of Efient®, Brilique® and Plavix®.<br>New text:<br>The reference safety information for classification of expected or unexpected SAEs are the current versions of the Summaries of Product Characteristics (SmPC) of Efient®, Brilique®, Plavix® and HerzASS-ratiopharm® 100 mg (Germany) or HerzschutzASS “ratiopharm” 100 mg (Austria). |

| | |
|---|---|
| PD Dr. med. Konstantinos Rizas<br>LMU University Hospital Munich<br>Department of Cardiology<br>Author and Coordinating Investigator (LKP) | München, 22.4.2021<br>Place, Date, Signature |
| Univ.-Prof. Dr. med. Steffen Massberg<br>LMU University Hospital Munich<br>Department of Cardiology<br>Author and Sponsor Delegated Person | München 22.04.2021<br>Place, Date Signature |

## Table of Changes Version 2.0 to Version 3.0 (refers to the version with track changes)

(new text in red)

| Section, page (track change) | Description of Change |
|---|---|
| Synopsis, page 14 | New text:<br>End of Trial: Last patient last visit<br>Reason:<br>Requested by BfArM, was missing in V. 2.0 |
| 7.2, page 27 | New text:<br>End of Trial: Last patient last visit<br>Reason:<br>Requested by BfArM, was missing in V. 2.0 |
| 9.6.1, page 33 | New text:<br>As a result the aspect of contraception after study completion depends on further treatment of the patient in line with the current guidelines and the respective SmPCs.<br>Reason:<br>Requested by BfArM, was missing in V. 2.0 |

| | |
|---|---|
| PD Dr. med. Konstantinos Rizas<br>LMU University Hospital Munich<br>Department of Cardiology<br>Author and Coordinating Investigator (LKP) | München, 28.5.21<br>Place, Date, Signature |

| | |
|---|---|
| Univ.-Prof. Dr. med. Steffen Massberg<br>LMU University Hospital Munich<br>Department of Cardiology<br>Author and Sponsor Delegated Person | 7.6.2021<br>Place, Date Signature |

# Table of Changes Version 3.0 to Version 4.0 (refers to the version with track changes)

(new text in red)

| Section, page (track change) | Description of Change |
|---|---|
| Synopsis, page 9 | <u>New text</u>:<br>In-hospital: Aspirin + Clopidogrel + non-vitamin K antagonist oral anticoagulant (up to 1 week)<br><u>Reason</u>:<br>Requested by EC |
| Synopsis, page 9 | <u>New text</u>:<br>Hospital-discharge or day 8 – month 6: Clopidogrel + non-vitamin K antagonist oral anticoagulant. In patients with high ischaemic risk ~~for stent thrombosis~~ the treating physician is allowed to prescribe Aspirin for a period of up to 4 weeks, according to current guidelines.<br><u>Reason</u>:<br>Requested by EC |
| Synopsis, page 10 (Exclusion criteria) | <u>New text</u>:<br>• ~~Previous~~ History of ischaemic ~~haemorrhagic~~ stroke or transient ischaemic attack (both contraindications for Prasugrel) and history of intracranial bleeding (contraindication for Ticagrelor)<br>• Contraindication for Clopidogrel or Aspirin~~$P2Y_{12}$-inhibitor~~<br>• Contraindication for Prasugrel and Ticagrelor<br>• Severe chronic liver disease (Child-Pugh C)<br><u>Reason</u>:<br>Requested by EC |
| Synopsis, page 11 | <u>New text</u>:<br><u>Primary efficacy endpoint:</u><br>Major ischaemic events defined as the composite of all-cause mortality, myocardial infarction, definite or probable stent thrombosis, ischaemic stroke or~~and~~ systemic thromboembolism (superiority test) at 6 weeks after randomization<br><u>Reason:</u><br>"Or" describes more precisely how the primary endpoint is analysed |

| | |
|---|---|
| List of abbreviations, page 16 | New text:<br>DAT = Dual Antithrombotic Therapy<br>Reason:<br>Was missing in V 3.0 |
| Section 6.1, page 26 | New text:<br>Primary endpoints at 6 weeks after randomization (hierarchical testing)<br>• Efficacy: Major ischaemic events defined as the composite of all-cause mortality, myocardial infarction, definite or probable stent thrombosis, ischaemic stroke or~~and~~ systemic thromboembolism (superiority test)<br>Reason:<br>“Or” describes more precisely how the primary endpoint is analysed |
| Section 7.2, page 27 | New text:<br>Planned start of recruitment: Q4~~3~~/2021<br>Reason:<br>Planned start of recruitment corrected |
| Section 8.3, page 28 | New text:<br>• ~~Previous~~ History of ischaemic ~~haemorrhagic~~ stroke or transient ischaemic attack (both contraindications for Prasugrel) and history of intracranial bleeding (contraindication for Ticagrelor)<br>• Contraindication for Clopidogrel or Aspirin~~$P2Y_{12}$-inhibitor~~<br>• Contraindication for Prasugrel and Ticagrelor<br>• Severe chronic liver disease (Child-Pugh C)<br>Reason:<br>Requested by EC |
| Section 9.2.2, page 32 | New text:<br>Standard regimen consisting of<br>In-hospital (up to 1 week): Aspirin plus Clopidogrel plus non-vitamin K antagonist oral anticoagulant<br>Discharge or day 8 – month 6: Clopidogrel plus non-vitamin K antagonist oral anticoagulant. In patients with high ischaemic risk the treating physician is allowed to prescribe Aspirin for a period of up to 4 weeks, according to current guidelines.<br>Reason:<br>Requested by EC |

<table>
<tr><td>Section 10.1.1, page 35</td><td>
<u>New text</u>:

- Laboratory parameters (post PCI and prior to randomization): Laboratory tests post PCI depend on the routine tests performed in each study centre. We recommend following tests: Haemoglobin, CK, troponin.

<u>Reason</u>:

Baseline laboratory parameters (haemoglobin, CK, troponin) should be provided, if available
</td></tr>
<tr><td>Section 10.1.1, page 36</td><td>
<u>New text</u>:

<u>Group 2 (control): treatment with Clopidogrel:</u>

Aspirin will be continued at a maintenance dose of 75-100mg/day (for Germany and Austria only 100 mg are applicable) for the in-hospital stay for a maximum of 7 days in the control group. According to the current guidelines, Aspirin is allowed to be continued for a maximum of 4 weeks in patients with high ~~risk for stent~~ ischaemic risk ~~thrombosis~~.

<u>Reason</u>:

Inconsistency corrected
</td></tr>
<tr><td>Section 12.4, page 43</td><td>
<u>New text</u>:

<u>On treatment with Aspirin (≤ 7 days or > 7 days)</u>

<u>Reason</u>:

Requested by EC
</td></tr>
<tr><td>Section 22.1, page 62</td><td>
The classifications of NYHA CLASSIFICATION OF HEART FAILURE and ACUTE CORONARY SYNDROME were moved from section 2.2 into section 2.1 because these classifications are relevant for clinical endpoints.
</td></tr>
<tr><td>Section 22.2, page 70</td><td>
<u>New text</u>:

**CHRONIC OBSTRUCTIVE PULMONARY DISEASE (COPD)**

Severity of Airflow Limitation Assessment in Patients with COPD

<table>
<tr><th>SEVERITY OF AIRFLOW LIMITATION</th><th>CLASSIFICATION</th><th>$FEV_1$*</th></tr>
<tr><td>MILD</td><td>GOLD I</td><td>≥80%</td></tr>
<tr><td>MODERATE</td><td>GOLD II</td><td>50%-79%</td></tr>
<tr><td>SEVERE</td><td>GOLD III</td><td>30%-49%</td></tr>
<tr><td>VERY SEVERE</td><td>GOLD IV</td><td><30%</td></tr>
</table>
</td></tr>
</table>

| | |
|---|---|
| | FEV$_1$ indicates forced expiratory volume in 1 second; FVC, forced vital capacity; GOLD, Global Initiative for Chronic Obstructive Lung Disease.<br>*Assessed in patients with FEV$_1$/FVC less than 0.70<br>Reason:<br>Classification of COPD was missing in V 3.0 |

| | |
|---|---|
| PD Dr. med. Konstantinos Rizas<br>LMU University Hospital Munich<br>Department of Cardiology<br>Author and Coordinating Investigator (LKP) | München 28.10.21 [signature]<br>Place, Date, Signature |

| | |
|---|---|
| Univ.-Prof. Dr. med. Steffen Massberg<br>LMU University Hospital Munich<br>Department of Cardiology<br>Author and Sponsor Delegated Person | [signature] 28.10.2021 [signature]<br>Place, Date Signature |

# Table of Changes Version 4.0 to Version 5.0 (refers to the version with track changes)

(new text in red)

| Section, page (track change) | Description of Change |
|---|---|
| Page 16 | Deleted:<br>~~DZHK Deutsches Zentrum für Herz-Kreislauf-Forschung e.V.~~<br>Reason:<br>The DZHK refused the financial support of the trial |
| Page 19 | Deleted:<br>~~Deutsches Zentrum für Herz-Kreislauf-Forschung e.V. (DZHK)~~<br>Reason:<br>The DZHK refused the financial support of the trial |
| Page 39 | Deleted:<br>~~(to be adapted accordingly)~~<br>Reason:<br>Remained from the template |
| Page 18 | Deleted:<br>~~… and Deutsches Zentrum für Herz-Kreislauf-Forschung e.V. (DZHK).~~<br>Reason:<br>The DZHK refused the financial support of the trial |

| | |
|---|---|
| PD Dr. med. Konstantinos Rizas<br>LMU University Hospital Munich<br>Department of Cardiology<br>Author and Coordinating Investigator (LKP) | München, 27.12.2021<br>Place, Date, Signature |

| | |
|---|---|
| Univ.-Prof. Dr. med. Steffen Massberg<br>LMU University Hospital Munich<br>Department of Cardiology<br>Author and Sponsor Delegated Person | 27.12.2021<br>Place, Date Signature |

# Table of Changes - Justification

| Study Information | | | |
|---|---|---|---|
| Sponsor:<br>Klinikum der Universität München | Study Short Title:<br>EPIDAURUS | Protocol-Code:<br>EPIDAURUS-2020 | EudraCT/EUDAMED No.:<br>2020-004748-27 |

Additions or changes to the study protocol (from version 5.0 to version 6.0) are shown in red and **bold** and deletions are shown in ~~strike out mode~~. Page numbers refer to Protocol Version 6.0 EU (20.03.2024), tracking mode

| Section | Page(s) | Change(s) | Justification |
|---|---|---|---|
| Footer | 1-78 | Version ~~5.0 (23 Dec-2021)~~ **6.0 EU (20Mar2024)** | New protocol version |
| Front Page | 1 | Version ~~5.0 (23 Dec-2021)~~ **6.0 EU (20 Mar-2024)** | New protocol version |
| Front Page – Coordinating Investigator<br><br>Declaration of the Author & Coordinating Investigator<br><br>Synopsis – Coordinating Investigator | 1<br><br>3<br><br>9 | Coordinating Investigator (~~LKP~~)<br><br>~~PD~~ **Prof**. Dr. med. Konstantinos Rizas | Term LKP no longer valid under EU Clinical Trial Regulation;<br><br>New title of Coordinating Investigator |

| | | | |
|---|---|---|---|
| Administrative Structure – Coordinating Investigator /Steering Committee | 20 | | |
| Investigator Agreement Page | 5 | I agree that the trial shall be carried out according to the **current** ICH E6 ~~(R2)~~ standard and all applicable regulatory requirements. | Maintaining up-to-dateness during study period |
| 1 Synopsis – Inclusion Criteria<br><br>8.2 Inclusion Criteria | 10<br><br>30 | • STEMI or NSTEMI (biomarker positive acute coronary syndrome)<br>• ~~and s~~**S**uccessful completion of PCI (**defined as TIMI flow grade 2 or more;** randomization will take place within ~~24h~~ **5 days (recommended within 24h)** after successful PCI **and before hospital discharge**) | Definition of inclusion criterion „Successful PCI“ was added.<br><br>Prolongation of randomization time window |
| 1 Synopsis – Exclusion Criteria<br><br>8.3 Exclusion Criteria | 11<br><br>30 | • **Any other reason in the opinion of the investigator making the patient ineligible for participation in the trial** | One exclusion criterion was added. |
| 1 Synopsis – Randomization<br><br>9.3 Treatment Assignment and Randomization | 11<br><br>34 | Patients will be randomized within ~~24h~~ **5 days (recommended within 24h)** after successful completion of PCI ~~and before hospital discharge~~ (defined as TIMI flow grade 2 or more; transradial approach preferred) **and before hospital discharge**. | Prolongation of randomization time window |

<table>
<tr>
<td>1 Synopsis – Study Visits: Control Visit (Day 42 ± 7) + Final Visit (Day 180 ± 10)<br><br>10.3 Control Visit (Day 42 ± 7)<br><br>10.4 Final Visit (180 ± 10)</td>
<td>12<br><br>42<br><br>42</td>
<td>• Applicable for Austria only: after randomization monthly pregnancy tests for women of childbearing potential</td>
<td>One consolidated protocol for all EU countries</td>
</tr>
<tr>
<td>1 Synopsis – Duration of the Trial</td>
<td>14</td>
<td>First patient in to last patient out (months): <s>30</s> 54<br><br>Duration of the entire trial (months):<s>36</s> 60<br><br>Recruitment period (months): <s>24</s> 48</td>
<td>Prolongation of recruitment period</td>
</tr>
<tr>
<td>2 Schedule of Activities</td>
<td>15</td>
<td>
<table>
<tr><th></th><th>Screening/<br><br>Baseline visit<br>Day 0</th><th>Discharge visit</th><th>Control visit<br><br>Day 42 ± 7</th><th>Final visit<br><br>Day 180 ± 10</th></tr>
<tr><td>Applicable for Austria only: after randomization monthly pregnancy tests for women of childbearing potential</td><td></td><td></td><td>X</td><td>X</td></tr>
</table>
</td>
<td>One consolidated protocol for all EU countries</td>
</tr>
<tr>
<td>3 List of Abbreviations</td>
<td>17</td>
<td>CTIS: Clinical Trial Information System<br><br><s>LKP Leiter der Klinischen Prüfung</s></td>
<td>New submission procedure via CTIS;<br><br>Term LKP no longer valid under EU CTR</td>
</tr>
</table>

| | | | |
|---|---|---|---|
| 4 Administrative Structure – Steering Committee | 20 | **PD Dr. med. Marc Kollum (Hegau-Bodensee-Klinikum Singen)** | New Steering Commitee Member |
| 5.1.1 Background | 23<br><br><br>24 | **In a recently published retrospective analysis (13), including 2,259 patients with ACS and AF treated with NOAC and P2Y12-inhibitors, treatment with Prasugrel or Ticagrelor compared to Clopidogrel led to a significant decrease of major adverse cardiac events, without a significant increase in bleeding complications after 12 months of follow-up.**<br>[…]<br>**The lack of evidence, as well as the clinical and scientific importance for a randomized trial comparing the combination of a potent P2Y12-inhibitor or Clopidogrel with NOAC in patients with atrial fibrillation is underscored in the recent ESC guidelines of the year 2023 for the management of ACS. EPIDAURUS is the only ongoing randomized trial testing the combination of direct factor Xa inhibitors with a potent P2Y12 -inhibitor or Clopidogrel in patients with ACS and AF. Two other ongoing randomized trials, initiated in the Netherlands (clinicaltrials.gov NCT04688723) and Poland (clinicaltrials.gov NCT04695106) will test the combination of direct thrombin-inhibitor Dabigatran with Ticagrelor vs. Dabigatran and Clopidogrel. The recently published retrospective analysis by Godtfredsen et al. supports the clinical hypothesis of the EPIDAURUS trial.** | Update of scientific background |
| 5.2 Dose Rationale | 26-27 | Switching between Clopidogrel and potent $P2Y_{12}$-inhibition or between **potent** $P2Y_{12}$-inhibition and Clopidogrel will be performed according to current recommendations. Patients with a new-diagnosed AF will be allowed to participate in the study if they are randomized within ~~24 hours~~ **5 days (recommendation within 24h)** after PCI **and before discharge.** | More precise wording<br><br>Prolongation of randomization time window |
| 7.1 Trial Design | 29 | It is planned that the trial is performed in a minimum of 2 countries within the European Union and **one country that is not a member state of the European Union.** | Inclusion of one Albanian study site. |

| | | | |
|---|---|---|---|
| | | 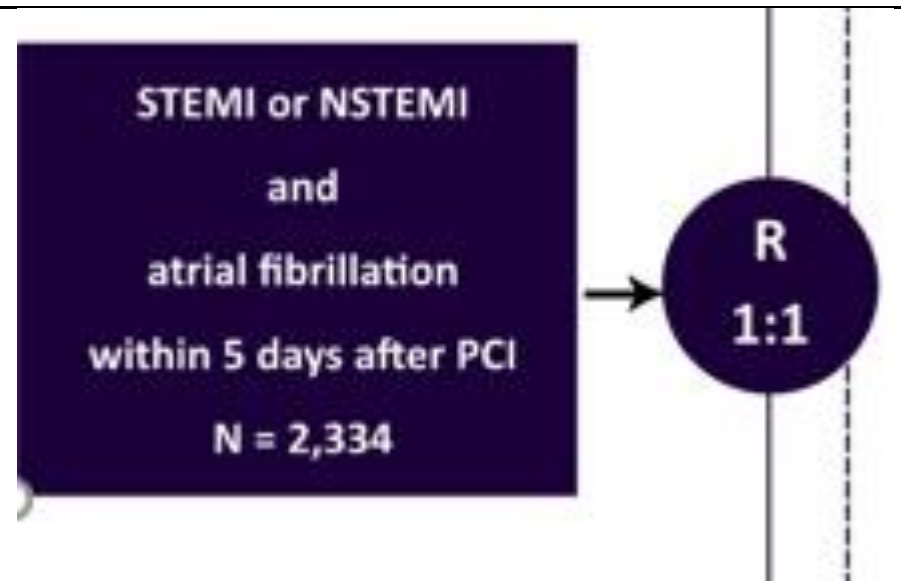 | Figure was updated (Prolongation of randomization time window) |
| 7.2 Trial Duration | 29 | Planned recruiting period: ~~24~~ **48** months | Prolongation of recruitment period |
| 8.4 Patient Recruitment and Screening | 31 | Screening is performed within ~~24 hours~~ **5 days (recommended within 24h)** after completion of PCI and **before hospital discharge**. | Prolongation of randomization time window |
| 9.5.2 Treatment Administration | 35 | The patients will be instructed by their treating physicians regarding the intake of IMP after discharge. The IMP will be prescribed by the general practitioner or cardiologist according to the randomized treatment regimen.<br><br>After 4 weeks the patients randomized in the experimental arm should be switched from Prasugrel or Ticagrelor to Clopidogrel by their treating general practitioner or cardiologist**. A patient contact by phone is recommended to ensure timely switch of medication according to the protocol.** The NOAC treatment remains unchanged. In the control group the patients remain on their treatment with Clopidogrel and NOAC. The IMPs are taken according to the instruction leaflet of the respective medication. If there is an indication, the treating physician is allowed to change the treatment to another anti-Xa-inhibitor, which however should be documented in the eCRF.<br><br>**If the patient is switched from Clopidogrel to Ticagrelor after randomization a loading-dose of 180mg Ticagrelor is recommended. If the patient is switched from Ticagrelor to Clopidogrel after randomization or at the end of the 4-weeks treatment period a loading prescription of 600mg Clopidogrel is recommended. For switching between Clopidogrel and Prasugrel and vice versa, no loading is recommended regardless of the timepoint of switching.** | Recommendation of an additional patient contact to ensure protocol adherence |

| | | | |
|---|---|---|---|
| | | ~~If the P2Y12-inhibitor loading at index PCI was performed with Clopidogrel or Prasugrel no additional loading with the randomized P2Y12-inhibitor will be performed after randomization. If the P2Y12-inhibitor loading at index PCI was performed with Ticagrelor switching to an irreversible P2Y12-inhibitor will be done according to current guidelines.~~ | More precise recommendations for loading |
| 9.6.4 Conditions for Terminating the Trial by the Sponsor | 37 | If the trial is prematurely terminated or suspended, the sponsor will promptly inform the Principal Investigators, ~~the EC's and the regulatory authorities~~ **and the Members States concerned** of the termination or suspension and the reason(s) for the termination or suspension. | New submission procedure via CTIS |
| 10 Trial Procedures | 36 | ~~10.1 Schedule of Assessments~~ | Bullet point deleted (subitem 10.1 not needed, no subitem 10.2) |
| 10.1 Screening / Baseline Visit | 38<br><br><br><br>40 | No clinical trial procedures will be conducted prior to taking consent from the participant. ~~The consent will not~~ **Randomization will** denote the enrolment into the trial.<br>[…]<br>Concomitant medication will be recorded:<br>• […]<br>• Peri-interventional **(including emergency medication)**: Aspirin, $P2Y_{12}$-inhibitors, heparin, GP IIb/IIIa inhibitors<br>• At screening **(planned medication after PCI and before randomization)**: Aspirin, $P2Y_{12}$-inhibitors, OAC, beta-blocker, ACEI/ARB, aldosterone antagonist, ARNI, SGLT2 inhibitors, amiodarone, digitalis, CCB, nitrates, statins<br>[…] | More precise wording<br><br><br><br>More precise descriptions to ensure correct documentation |

| | | | |
|---|---|---|---|
| | | Randomization within ~~24 hours~~ **5 days (recommended within 24h)** after successful completion of PCI **(defined as TIMI flow grade 2 or more) and before hospital discharge:** ~~with TIMI flow grade 2 or more~~: All patients who meet the inclusion and none of the exclusion criteria are randomized 1:1 into two groups:<br>[…] | Prolongation of randomization time window |
| 10.2 Visit at Discharge | 41 | **Four weeks after randomization a patient contact by phone for patients randomized into the experimental arm is recommended to ensure timely switch of medication according to the protocol.** | Recommendation of an additional patient contact to ensure protocol adherence |
| 11.2.1.2 Assessment of Intensity | 43 | All AEs **reported in the eCRF** have to be graded regarding their intensity. | Selective approach for safety reporting due to sufficiently characterized safety profile of IMPs |
| 11.3.1 Adverse Events | 44-45 | All AEs which occur after randomization until the 6 months follow-up visit (final visit) must be collected by the investigator~~,~~ **and** recorded on the patient file**.** ~~and entered on the AE page of the eCRF.~~<br>**Recording in the eCRF is only required for events meeting the following criteria:**<br>**1. Serious adverse events (Definition see section** Fehler! Verweisquelle konnte nicht gefunden werden.**)**<br>**2. Important medical events (see SAE definition in section** Fehler! Verweisquelle konnte nicht gefunden werden.**)**<br>**3. Medication error/overdose (intentional or unintentional): at least to be reported as protocol deviation (see section** Fehler! Verweisquelle konnte nicht gefunden werden.**)** | Selective approach for safety reporting due to sufficiently characterized safety profile of IMPs |

**4. Adverse events that led to study drug discontinuation**

**5. Pregnancy and lactation exposure and outcomes**

**6. Adverse events of special interest: i.e. all endpoint relevant non-serious adverse events as well as bleedings BARC ≥ 1**

**This selective approach of safety data collection according to the ICH guideline E19 on a selective approach to safety data collection is justified because the safety profile of all IMPs (Aspirin, Clopidogrel, Prasugrel and Ticagrelor) is sufficiently characterized and all prerequisites listed in paragraph 2.2 of the referenced guideline including mechanistic, clinical safety, clinical pharmacology and non-clinical toxicology factors are met. Moreover, the EPIDAURUS trial shows several similarities to approval and post-approval studies for potent $P2Y_{12}$-inhibitors and there is a plenty of qualitative post-approval surveillance data available. All these factors support a selective safety data collection in the EPIDAURUS trial.**

[…]

Abnormal laboratory values or test **results have to be reported as adverse event only if they lead to discontinuation of the IMP.** ~~constitute adverse events only if they fulfil at least one of the following criteria:~~

- ~~they induce clinical signs or symptoms~~
- ~~they are considered clinically significant~~
- ~~they require therapy~~

[…]

If known, the diagnosis of the underlying illness or disorder should be recorded by the investigator, rather than its individual symptoms. All signs, symptoms, and laboratory test findings related to the underlying diagnosis should be collectively recorded as a single diagnosis. The following information should be captured for all **reported** AEs: onset, stop date (duration), intensity, seriousness, relationship to

| | | | |
|---|---|---|---|
| | | investigational medicinal product, action taken, treatment required and outcome. ~~If treatment for the AE was administered, it should be recorded on the concomitant medication page of the eCRF.~~ | |
| 11.3.2.2 Exclusion from Reporting of SAE's | 47 | • medical admission for purpose other than remedying ill health state and was planned prior to ~~signing informed consent~~ **randomization**. Appropriate documentation is required in these cases. | Randomization denotes enrolment into the trial |
| 11.4 Expedited Reporting | 47 | If a Serious Adverse Event is assessed as a Suspected Unexpected Serious Adverse Reaction (SUSAR), the sponsor or his designee has to submit the event to Eudravigilance~~ and the Competent Authorities~~. ~~The leading Ethics Committees will be informed as well as t~~**The participating investigators will be informed as well.** | New submission procedure via CTIS |
| 11.5 Reporting of Trial Treatment Errors including Misuse / Abuse | 47 | The occurrence of treatment errors of the IMP including misuse and abuse has to be documented **as protocol deviation** and reported as AE/SAE **in case the definition of an AE/SAE and requirements for safety reporting described in sections** Fehler! Verweisquelle konnte nicht gefunden werden. **to REF _Ref158631713 \r \h * MERGEFORMAT** Fehler! Verweisquelle konnte nicht gefunden werden. **are met.** | Selective approach for safety reporting due to sufficiently characterized safety profile of IMPs |
| 11.7 Annual Safety Report | 48 | The Annual Safety Report is submitted to **the Member States concerned.** ~~Competent Authorities and the Leading Ethics Committees.~~ | New submission procedure via CTIS |
| 12.4 Pre-specified subgroup analysis | 50 | • Max CK > 1000 U/L **from admission up to 48 h after completion of PCI** ~~during the first 48h after the index myocardial infarction~~<br>• Increased PRD or decreased DC. **PRD will be calculated from 20-Min ECGs recorded in Frank-leads configuration or from normal 10-second 12-lead ECGs. DC will be estimated from 20-Min ECG recordings.** | More precise description |
| 13 Substudy | 50 | Platelet function testing will be applied in the centres, where PFT-assays are **available and performed within clinical routine** and the centres agree to participate in the substudy. **Available platelet function** | |

| | | | |
|---|---|---|---|
| | | **testing results starting from 2 hours after loading will be recorded in the eCRF.** ~~Samples will be drawn after randomization (not less than 4 hours after loading and not later than 2 hours after randomization) and 24h after randomization.~~ | More precise description |
| 14.2 Compliance with Protocol and Protocol Revisions | 51 | The investigator should conduct the trial in compliance with the protocol agreed to by sponsor **and approved by the Member States concerned.** ~~, the Regulatory Authorities and for which an approval by the ECs was given.~~<br><br>Any significant protocol deviation will be documented and explained by the investigator or the person designated by the investigator in the eCRF and reported to the sponsor or his designee and will be included in the **submission of results** ~~clinical trial report~~. Corrective and preventive actions (CAPA) may be developed by the site and/or the sponsor or his designee, implemented and followed up as appropriate.<br><br>The study shall be conducted as described in this approved protocol. All revisions to the protocol must be discussed with, and be prepared by sponsor. The investigator should not implement any deviation or change to the protocol without prior review and documented approval~~/favourable opinion from the IEC~~ of an amendment **by the Member States concerned**, except where necessary to eliminate an immediate hazard(s) to study subjects. Any deviation must be documented in the eCRF.<br><br>If a deviation or change to a protocol is implemented to eliminate an immediate hazard(s) prior to obtaining ~~EC's favourable opinion~~ **approval by the Member States concerned**, as soon as possible the deviation or change will be submitted to:<br><br>• Sponsor<br><br>• ~~ECs~~ **the Member States concerned** for review and approval~~/favourable opinion~~<br><br>~~The favourable opinion signed by the chairperson or designee of the ECs must be sent to the sponsor.~~<br>If an amendment substantially alters the study design or increases the potential risk to the subject:<br><br>(1) the informed consent form must be revised and submitted to the ~~ECs~~ **Member States concerned** for review and **approval** ~~favourable opinion~~;<br><br>[…] | New submission procedure via CTIS |
| 15.2 Data Safety Monitoring Board | 52 | Therefore the DSMB is provided with the AEs and SAEs that **were reported** ~~occurred~~ during the conduct of the trial. | Only <u>reported</u> events can be assessed |

| | | | |
|---|---|---|---|
| 16.2 Source Data | 53 | The investigator must also keep the original Informed Consent Form signed by the patient (a second ~~originally signed~~ copy is given to the patient). | Handing out an ICF-copy to study participants is sufficient according to GCP |
| 16.5 Archiving and Data Retention | 55 | All records are to be retained by the Principal Investigator for a minimum of **25** ~~15~~ years after completion of the trial, i.e. according to the applicable regulations and guidelines. | Following current regulations and guidelines |
| 17.1 Good Clinical Practice | 56 | The clinical trial **is** ~~will be~~ performed in compliance with the protocol, the Declaration of Helsinki (2013), ICH E6 (R2), Directive 2001/20/EC **(applicable until transition into CTIS)** and Regulation (EU) No 536/2014, as applicable, and applicable local laws and regulations.<br><br>Personnel involved in conducting this clinical trial is qualified by education, training, and experience to perform their respective task(s). | New submission procedure via CTIS |
| 17.2 Ethics Commission and Regulatory Authorities | 56 | It is the responsibility of the sponsor to obtain approval for the trial protocol from the **Member States concerned** ~~Competent Authorities (CA) and the ECs~~ according to **applicable** ~~local~~ regulatory requirements.<br><br>During the clinical trial any amendment or modification to the clinical trial protocol must be submitted to the **Member States concerned** ~~Competent Authorities and ECs~~. ~~The CA/EC have to be informed of any event likely to affect the safety of the patients or the conduct of the trial and/or has an impact on the risk-benefit relation of the clinical trial.~~ All updates of the SmPCs will be sent to the **Member States concerned** ~~CA/EC~~.<br><br>~~At the end of the trial the sponsor will notify the CA/EC about trial completion.~~<br><br>~~In addition, the local regulatory authorities have to be notified about the trial and the participating German sites before the initiation as well as any amendments and modifications throughout the conduct of the trial.~~ | New submission procedure via CTIS |
| 17.3 Informed Consent | 57 | An originally signed copy of the form must be kept on the patient's file; a second ~~originally signed~~ copy will be handed over to the trial participant. | Handing out an ICF-copy to |

| | | | |
|---|---|---|---|
| | | | study participants is sufficient according to GCP |
| 17.4 Further Reporting Obligations | 57 | **17.4 Further Reporting Obligations**<br>**17.4.1 Serious Breaches**<br>**A 'serious breach' means a breach likely to affect to a significant degree the safety and rights of a subject or the reliability and robustness of the data generated in the clinical trial.**<br>**The investigator has to immediately report any events that might meet the definition of a serious breach to the sponsor.**<br>**The sponsor will notify the Member States concerned about a serious breach of the Regulation (EU) No 536/2014 or of the version of the protocol applicable at the time of the breach through the EU portal without undue delay but not later than seven days of becoming aware of that breach.**<br>**17.4.2 Other Reporting Obligations relevant for Subject Safety**<br>**Unexpected events: The sponsor will notify the Member States concerned through the EU portal of all unexpected events which affect the benefit-risk balance of the clinical trial, but are not suspected unexpected serious adverse reactions as referred to in Article 42 of Regulation (EU) No 536/2014. That notification will be made without undue delay but no later than 15 days from the date the sponsor became aware of this event.**<br>**The sponsor will submit to the Member States concerned, through the EU portal, all inspection reports of third country authorities concerning the clinical trial.**<br>**17.4.3 Urgent Safety Measures**<br>**Where an unexpected event is likely to seriously affect the benefit-risk balance, the sponsor and the investigator shall take appropriate urgent safety measures to protect the subjects.**<br>**The sponsor will notify the Member States concerned, through the EU portal, of the event and the measures taken. That notification will be made without undue delay but no later than seven days from the date the measures have been taken.** | Further reporting obligations according to Regulation (EU) No 536/2014 |

| | | | |
|---|---|---|---|
| | | **17.4.4 Start, End, Temporary Halt, and Early Termination of a Clinical Trial**<br><br>**Start, first visit of the first subject in each Member State, end of the recruitment, end of the clinical trial, temporary halt for reasons not affecting the benefit-risk balance, restart and early termination of the clinical trial for reasons not affecting the benefit-risk balance will be reported to the Member States concerned through the EU portal within 15 days (Regulation (EU) No 536/2014 Articles 36, 37, 38).** | |
| 20 Submission of the Results and Publication Policy | 58 | **20 ~~Clinical Trial Report~~ Submission of the Results and Publication Policy**<br><br>Prior to start of recruitment the trial must be registered in the publicly accessible database “clinicaltrials.gov”.<br><br>**Irrespective of the outcome of the clinical trial, within one year from the end of the clinical trial in all Member States concerned, the sponsor will submit to the EU database a summary of the results of the clinical trial, including a summary understandable to laypersons.**<br><br>~~A clinical trial report should be written within 12 months (after completion of the trial (last patient last visit)) and submitted to the relevant Ethics Committees and Regulatory Authorities in accordance with local requirements.~~ | New submission procedure via CTIS according to Regulation (EU) No 536/2014 |
| 22.1 Appendix 1: Endpoint Definitions | 62 | **<u>BLEEDING</u>:**<br><br>[…]<br><br>Type 2: Any overt, actionable sign of haemorrhage (e.g., more bleeding than would be expected for a clinical circumstance, including bleeding found by imaging alone) that does not fit the criteria for type 3, 4, or 5 but does meet at least one of the following criteria: ~~51~~ (1) requiring nonsurgical, medical intervention by a healthcare professional, (2) leading to hospitalization or increased level of care, or (3) prompting evaluation | By mistake in V5.0 |
| 22.2 Appendix 2: Definition of Clinical and Angiographic Characteristics | 76 | **SMOKING STATUS**<br><br>Smoker: any cigarette smoking in the prior 6 months<br>Non-smoker: no regularly cigarettes smoking at any time (according to the World Health Organization also former smoker that quit smoking for at least 10 years) | Smoking status is assessed twice (at screening and at final visit) |

| | | | |
|---|---|---|---|
| | | Former smoker: those who had quit smoking at least 6 months ago ~~before the index PCI~~ | |

02 Apr 2024

**Date** (DD-Mmm.-YYYY): **Signature /** Prof. Dr. med. Steffen Massberg (Sponsor Delegated Person)

Prof. Dr. med. Konstantinos Rizas
Leitung Campus Innenstadt
LMU Klinikum
Medizinische Klinik und Poliklinik I
Ziemssenstraße 5 · 80336 München

2-4-2024

**Date** (DD-Mmm.-YYYY): **Signature /** Prof. Dr. med. Konstantinos Rizas (Coordinating Investigator)

# Table of Changes - Justification

| **Study Information** | | | |
|---|---|---|---|
| Sponsor:<br>Klinikum der Universität München | Study Short Title:<br>EPIDAURUS | Protocol-Code:<br>EPIDAURUS-2020 | EudraCT/EUDAMED No.:<br>2020-004748-27 |

Additions or changes to the study protocol (from version 6.0 to version 7.0) are shown in red and **bold** and deletions are shown in ~~strike out mode~~. Page numbers refer to Protocol Version 7.0 EU (10Jun2024), tracking mode

| **Section** | **Page(s)** | **Change(s)** | **Justification** |
|---|---|---|---|
| Footer | 1-76 | Version ~~6.0 (20Mar2024 )~~ **7.0 EU (10Jun2024)** | New protocol version |
| Front Page | 1 | Version ~~5.0 (23 Dec 2021)~~ **7.0 EU (10Jun2024)** | New protocol version |
| 5.1.1 Background | 24<br><br>26 | In this trial, ~~30%~~ **29%** of the patients presented with biomarker positive myocardial infarction (MI) ~~and 22% with unstable angina~~. Ninety-four percent of the patients were treated with Clopidogrel and only 6% of the patients were on treatment with a potent $P2Y_{12}$-inhibitor (Prasugrel or Ticagrelor). Compared to standard TAT with VKA, Rivaroxaban-based regimens were associated with a significant reduction in adverse bleeding events without a significant increase in major adverse cardiovascular events. However, the power for the exploratory analysis of ischaemic events was estimated to be 11.4%. **(7)**<br><br>~~Two~~ **One** other ongoing randomized trial, initiated in ~~the Netherlands (clinicaltrials.gov NCT04688723) and~~ Poland (**ADONIS-PCI** clinicaltrials.gov NCT04695106) will test the combination of direct thrombin-inhibitor Dabigatran with Ticagrelor vs. Dabigatran and Clopidogrel. The recently published retrospective analysis by Godtfredsen et al. supports the clinical hypothesis of the EPIDAURUS trial. | Update of scientific background<br><br>Addition of reference |

| | | | |
|---|---|---|---|
| Table 1 | 27 | Stable Angina (%) 22.2% 45.8% 45.6% 38.8%<br>Unstable Angina (%) 47.7% 17.9% 15.6%<br>**NSTEMI /STEMI (%)** ~~29~~30.1% ~~50~~36.3% ~~52~~39% ~~51~~ 61.2 % (37% PCI) | Update of table |
| 9.5.2 Treatment Administration | 38 | If the patient is switched from Clopidogrel to Ticagrelor after randomization a loading-dose of 180mg Ticagrelor is recommended **according to current guidelines (15) and the international expert consensus recommendation (16).** If the patient is switched from Ticagrelor to Clopidogrel after randomization or at the end of the 4-weeks treatment period a loading prescription of 600mg Clopidogrel is recommended **according to current guidelines (15) and the international expert consensus recommendation (16).** For switching between Clopidogrel and Prasugrel and vice versa, no loading is recommended regardless of the timepoint of switching. | Addition of references |
| 21 References | 55 | 7. Sibbing D, Rizas K, Massberg S. Spoilt for choice – P2Y12-receptor inhibition in patients with atrial fibrillation undergoing percutaneous coronary intervention. JACC Interventions in press 2024. | Addition of reference 7 |

10 June 2024

**Date** (DD-Mmm.-YYYY): **Signature /** Prof. Dr. med. Steffen Massberg (Sponsor Delegated Person)

10.6.24

**Date** (DD-Mmm.-YYYY): **Signature /** Prof. Dr. med. Konstantinos Rizas (Coordinating Investigator)

LMU KLINIKUM

Deutsches Herzzentrum München
des Freistaates Bayern
Klinik an der Technischen Universität München

# Table of Changes - Justification

| Study Information | | | |
|---|---|---|---|
| Sponsor:<br>Klinikum der Universität München | Study Short Title:<br>EPIDAURUS | Protocol-Code:<br>EPIDAURUS-2020 | EudraCT/EUDAMED No.:<br>2020-004748-27 |

Additions or changes to the study protocol (from version 7.0 to version 8.0) are shown in red and **bold** and deletions are shown in ~~strike out mode~~. Page numbers refer to Protocol Version 8.0 EU (05Jan2025), tracking mode

| Section | Page(s) | Change(s) | | Justification |
|---|---|---|---|---|
| Footer | 1-79 | Version ~~7.0 (10Jun2024)~~ **8.0 EU (05Jan2025)** | | New protocol version |
| Front Page | 1 | Version ~~7.0 (10Jun2024)~~ **8.0 EU (05Jan2025)**<br>Principal Investigators: Prof. Dr. med. Konstantinos Rizas,<br>Univ.-Prof. Dr.rer. nat. Ulrich Mansmann<br>Univ.-Prof. Dr. med. Steffen Massberg | | New protocol version<br>Naming of princpal investigators |
| Synopsis | 12 | Principal Investigators | Prof. Dr. med. Konstantinos Rizas,<br>LMU University Hospital Munich, Department of Cardiology Marchioninistr. 15, 81377 Munich, Germany<br><br>Univ.-Prof. Dr.rer. nat. Ulrich Mansmann,<br>Institute for Medical Information Processing, Biometry, and Epidemiology, Marchioninistr. 15, 81377 Munich, Germany<br><br>Univ.-Prof. Dr. med. Steffen Massberg | Adding principal investigators |

| | | | | |
|---|---|---|---|---|
| | | | LMU University Hospital Munich, Department of Cardiology<br>Marchioninistr. 15, 81377 Munich, Germany | |
| Synopsis | 14 | Primary Endpoints: | Primary efficacy endpoint:<br>Major ischaemic events defined as the composite of all-cause mortality, myocardial infarction, definite or probable stent thrombosis, ischaemic stroke ~~or~~, systemic thromboembolism, or urgent revascularization (superiority test) at 6 weeks after randomization<br>Primary safety endpoint:<br>~~Bleeding~~ All cause mortality, bleeding type 2 or higher according to the Bleeding Academic Research Consortium (BARC) criteria (non-inferiority test) at 6 weeks after randomization<br>Both primary endpoints will be assessed using the Finkelstein and Schoenfeld test (win/loss ratio) using a decreasing ranking for the above-mentioned components of the endpoints. | Urgent revascularization (previously secondary endpoint) has been considered as the last element of the composite endpoint with the lowest hierarchical priority.<br>A win-loss ratio should always include mortality as the endpoint with the highest priority. |
| | | Secondary Endpoints: | • Urgent revascularization at 6 ~~weeks~~months after randomization | We considered a new secondary endpoint |

| | | | | |
|---|---|---|---|---|
| Synopsis | 16/17 | Sample Size / Number of patients / Planned No. of Sites: | To be assessed for eligibility (n = ~~2500~~2000)<br>To be allocated to trial (n = ~~2334~~1474)<br>To be analysed (n = ~~2304~~1390)<br>Assumptions for sample-size calculation (hierarchical testing)<br>• Efficacy endpoint (ischaemic events) at 6 weeks:<br>~~Superiority testing, 2-sided alpha 5%, power 80%, 6-weeks event-rate in the control group 7%, 6-weeks event-rate in the intervention group 4.3%, corresponding to an odds-ratio of 0.6.~~<br>~~Number per group: 1147~~<br>Superiority testing, using the Finkelstein and Schoenfeld test (win-loss-ratio; WLR) and application of decreasing ranking for the components of the ischaemic endpoint: (i) death; (ii) definite or probable stent thrombosis; (iii) myocardial infarction; (iv) stroke; (v) systemic embolism; (vi) urgent revascularization. The hazard rates in the control group were calculated on the basis of the analysis of the 223 control-patients included in EPIDAURUS until November 4th, 2024. The assumed effect of intervention (cumulative odds-ratio 0.67), which was based on previous studies, remained unchanged. For the calculations we used a simulation approach with 10^6 iterations.<br>Null hypothesis H0: WLR =1. For a two-sided alpha of 0.05, 6-week hazard-rates and SE of hazard rates according to the values provided above the power is 80.0% if comparison of WLR is performed on 615 patients per group. To account for dependency between the different endpoints the sample size was increased by 10% leading to a sample size of **677** patients per group. The computation assumes an allocation ratio ($n_{control}/n_{interventional}$) = 1.0.<br>• Safety endpoint ~~(bleeding BARC ≥ 2) at 6 weeks:~~ | Using win-loss ratio we could reduce the overall sample size of the trial. The details of the statistical analysis are described in this section. |

| | | | | |
|---|---|---|---|---|
| | | | • : Non-inferiority testing~~, when~~ for mortality, bleeding BARC 3c, BARC 3b, BARC 3a and BARC ≥ 2) at 6 weeks tested using the Finkelstein and Schoenfeld test (WLR) and application of decreasing ranking for the components of the safety endpoint.<br><br>When the sample size in each group is ~~1152~~**695**, a two-group large-sample normal approximation test of proportions with a one-sided 2.5% significance level will have 80% power to reject the null hypothesis that the ~~test and the standard are not equivalent (6-weeks event-rate in the control group 13.1%, odds-ratio between treatment and control-group 0.95, difference in proportions, $\pi_1 - \pi_0$, is -0,033 or farther from zero in the same direction) in favour of the alternative hypothesis that the proportions in the two groups are equivalent~~lower 97.5% confidence interval of the  WLR compered between the intervention and control arm ~~compered between the intervention and control arm~~ is lower than 0.77 (1/(1 + delta), with delta = 0.3) in favour of the alternative hypothesis that the lower 97.5% confidence interval is greater than or equal to 0.77. A dependency between the endpoints was not considered, as the endpoints of the safety analysis are mutually exclusive.The hazard rates ın the control group were calculated on the basis of the analysis of the 223 control-patients included in EPIDAURUS until November 4th, 2024. The assumed effect of intervention, which was based on previous studies, remained unchanged. For the calculations we used a simulation approach with 10^6 iterations.<br><br>~~Number per group: 1152~~<br><br>The sample size of the trial is given by the maximum of both sample sizes: ~~1152~~**695** per group.<br><br>~~The computation assumed~~Assuming an allocation ratio $n_{control}/n_{interventional}$ = 1.0~~, an accrual time of 24 months, a follow-up time of 6 months~~ and ~~an annual 10% follow-up~~a 6% drop-out rate ~~(6-week~~ | |

| | | | | |
|---|---|---|---|---|
| | | | ~~rate 1.25%, 2304 * 1.0125 = 2334).~~, based on an analysis of available data, the sample study of the study was calculated to be **1474**. | |

| | | | | |
|---|---|---|---|---|
| Synopsis | 17/18 | Data Analysis: | ~~Efficacy endpoint:~~<br>~~Superiority testing, 2-sided alpha 5%, power 80%, 6 weeks event-probability in the control group based on a post-hoc analysis of the ISAR-REACT 5 trial 7%, 6 weeks event-probability in the intervention group 4.3%, corresponding to an odds-ratio of 0.6.~~<br>~~Null hypothesis $H_0$: OR =1 (difference). For a two-sided alpha of 0.05, 6-week event probability in the control group of 0.070 and in the intervention group of 0.043 (OR = 0.6) the power is 80.0% if the chi-square test is performed on 1147 patients per group. The computation assumes an allocation ratio (n2/n1) = 1.0.~~<br>~~Safety endpoint:~~<br>~~Non-inferiority testing, when the sample size in each group is 1152, a two-group large-sample normal approximation test of proportions with a one-sided 2.5% significance level will have 80% power to reject the null hypothesis that the test and the standard are not equivalent (6-week event-rate in the control group 13.1%, odds-ratio between treatment and control-group 0.95, difference in proportions, $\pi_1 - \pi_0$, is -0,033 or farther from zero in the same direction) in favour of the alternative hypothesis that the proportions in the two groups are equivalent.~~<br>~~The sample size of the trial is given by the maximum of both sample sizes: 1152 per group.~~<br>~~Sample size calculations were performed using the nQuery platform for clinical trial design.~~ | The details of the statistical analysis are described in this section. |

| | | | | |
|---|---|---|---|---|
| | | | ~~Compliance/Rate of loss to follow-up:~~<br><br>~~We assumed an annual 10% drop-out rate (6-week rate 1.25%) and increase in the total sample size to 2334 (=2304 * 1.0125). The computation assumed an allocation ratio $n_{control}/n_{experimental}$ = 1.0 and a constant accrual time of 24 months.~~The efficacy composite endpoint includes (i) death, (ii) definite or probable stent thrombosis, (iii) myocardial infarction, (iv) stroke, (v) systemic embolism, and (vi) urgent revascularization. The safety composite endpoint includes (i) death, (ii) BARC 3c bleeding, (iii) BARC 3b bleeding, (iv) BARC 3a bleeding, and (v) BARC 2 bleeding. The endpoints will be tested using a hierarchical design with superiority testing of the first endpoint and non-inferiority testing of the second endpoint, if superiority of the first endpoint is met. Both composite endpoints will be analyzed using Finkelstein and Schoenfeld test (win-loss-ratio; [WLR] using a decreasing ranking.<br><br>We will calculate the WLR using an unmatched approach with application of bootstrapping (boot.n = 1.000 iterations) for calculation of SE and 95% confidence intervals. The results will be presented using forrest plots. We will also use Kaplan-Maier estimates to visualize time-to specific events (ischaemic events and bleeding events). A similar approach will be used for secondary endpoints. | |
| Synopsis | 18/19 | Duration of the Trial: | First patient in to last patient out (months): ~~54~~78<br><br>Duration of the entire trial (months): ~~60~~84<br><br>Recruitment period (months): ~~48~~72 | Prolongation of recruitment period |

| | | | |
|---|---|---|---|
| 6.1 Primary endpoints | 32 | Primary endpoints at 6 weeks after randomization (hierarchical testing)<br>• Efficacy: Major ischaemic events defined as the composite of all-cause mortality, myocardial infarction, definite or probable stent thrombosis, ischaemic stroke, systemic thromboembolism or urgent revascularization (superiority test)<br>• Safety: All cause mortality, bleeding type 2 or higher according to the Bleeding Academic Research Consortium (BARC) criteria (non-inferiority test) | A win-loss ratio should always include mortality as the endpoint with the highest priority. |
| 7.2 Trial Duration | 33 | Planned recruiting period: ~~48~~66 months | Prolongation of recruitment period |
| 8.1 Number of patients | 34 | A total number of ~~2334~~1474 patients should be allocated to the trial, a number of ~~2304~~1390 patients should be analysed. | Recalculation of sample size |

| | | | |
|---|---|---|---|
| 12. Sample Size Calculation | 48-50 | The efficacy composite endpoint includes (i) death, (ii) definite or probable stent thrombosis, (iii) myocardial infarction, (iv) stroke, (v) systemic embolism, and (vi) urgent revascularization. The safety composite endpoint includes (i) death, (ii) BARC 3c bleeding, (iii) BARC 3b bleeding, (iv) BARC 3a bleeding, and (v) BARC 2 bleeding. The endpoints will be tested using a hierarchical design with superiority testing of the first endpoint and non-inferiority testing of the second endpoint, if superiority of the first endpoint is met. Both composite endpoints will be analyzed using Finkelstein and Schoenfeld test (win-loss-ratio; [WLR] (18) using a decreasing ranking. For sample size calculation we assumed a normal distribution of hazards and applied a simulation approach using the hazard-rates and standard errors (SE) provided in Table 2. The event-rates, hazard-rates, SE of hazard rates and drop-out rates of the control group were derived from an analysis of the 223 control patients included in EPIDAURUS until November 4th, 2024. In order to avoid power-loss no direct comparison between control and intervention groups was performed. The assumed effect of intervention on outcome remained unchanged (cumulative odds ratio 0.67 for the ischaemic endpoints and 0.95 for the bleeding endpoints) compared to previous protocol versions and was derived from unpublished data of the ISAR REACT 5 trial. The assumed effect size is in-line with a recently published retrospective analysis comparing escalated vs. standard therapy in patients with AF and ACS (14).<br>Assumptions for sample-size calculation (hierarchical testing) | This is a detailed description of sample size calculation using win-loss ratio. The table summarizes the assumed 6-week event rates and hazard rates for all events. |

- Primary efficacy endpoint (ischaemic events) at 6 weeks

  Superiority testing, 2-sided alpha 5%, power 80%, 6~~-~~ weeks ~~event-rate~~hazard-rates in the control group ~~7% (~~on the basis of the analysis of the control patients enrolled in EPIDAURUS until November, 4th 2024 (Table 2). Hazard-rates in the interventional group are derived from a post-hoc analysis of the ISAR-REACT 5 ~~study, data not~~trial and a recently published~~),~~ retrospective analysis comparing escalated vs. standard therapy in patients with AF and ACS (14) (8,19).

  Null hypothesis H0: WLR =1. For a two-sided alpha of 0.05, 6-~~weeks event-rate in the intervention group 4.3%, corresponding~~week hazard-rates and SE of hazard rates according to the values provided in Table 2 the power is 80.0% if comparison of WLR is performed on 615 patients per group. The computation assumes an ~~odds-~~allocation ratio ~~of 0.6~~ ($n_{control}/n_{interventional}$) = 1.0. To account for dependency between the different endpoints the sample size was increased by 10% (~~8,18~~20)~~.~~ [, leading to a sample size of **677** patients per group. The computation assumes an allocation ratio (ncontrol/ninterventional) = 1.0.

  Number per group: ~~1147~~**677**

- Primary safety endpoint (bleeding BARC ≥ 2) at 6 weeks

  Non-inferiority testing~~, when~~ for mortality, bleeding BARC 3c, BARC 3b, BARC 3a and BARC ≥ 2) at 6 weeks tested using the Finkelstein and Schoenfeld test (WLR) and application of decreasing ranking for the components of the safety endpoint. When the sample size in each group is ~~1,152~~**695**, a two-group large-sample normal approximation test of proportions with a one-sided 2.5% significance level will have 80% power to reject the null hypothesis that the ~~test and the standard are not equivalent (6-weeks event-rate in the control group 13.1% (ISAR-REACT~~ lower 97.5~~ study, data not published), odds-ratio between experimental and control group 0.95, difference in proportions, $\pi_1 - \pi_0$,~~% confidence interval of the WLR compered between the intervention and control arm is ~~-~~lower than 0.77 (1/(1 + delta), with delta = 0~~,033 or farther from zero in the same direction~~.3) in favour of the alternative hypothesis that the ~~proportions in~~lower 97.5% confidence interval is greater than or equal to 0.77. A dependency between the endpoints was not considered, as the endpoints of the ~~two groups are equivalent.~~safety analysis are mutually exclusive. ~~Number per group: 1152~~

The sample size of the trial is given by the maximum of both sample sizes: ~~1152~~695 per group.

~~The computation assumed~~ Assuming an allocation ratio ncontrol/~~$n_{experimental}$~~ninterventional = 1.0~~, an accrual time of 24 months, a follow-up time of 6 months~~ and ~~an annual 10% follow-up~~a 6% drop-out rate (~~6-week rate~~derived from an analysis of available data), the sample study of the study was calculated to be 1474. (1390 * 1.~~25%, 2304 * 1.0125 = 2334).~~06 = **1474**).

**Table 2:** 6-week event-rates, hazard-rates and their corresponding standard errors (SE), which were used for sample-size calculation.

| | Control group | | | Intervention group | | | HR |
|---|---|---|---|---|---|---|---|
| **Endpoint** | **Event rate** | **Hazard rate** | **SE of hazard rate** | **Event rate** | **Hazard rate** | **SE of hazard rate** | |
| **Death** | 4.35% | 4.44% | 1.57% | 3.28% | 3.33% | 1.18% | 0.75 |
| **Stent thrombosis** | 1.17% | 1.18% | 0.84% | 0.34% | 0.35% | 0.25% | 0.30 |
| **Myocardial infarction** | 3.79% | 3.87% | 1.46% | 1.91% | 1.93% | 0.73% | 0.50 |
| **Ischaemic stroke** | 1.00% | 1.01% | 0.50% | 0.70% | 0.70% | 0.35% | 0.70 |
| **Systemic embolism** | 1.00% | 1.01% | 0.50% | 0.70% | 0.70% | 0.40% | 0.70 |
| **Urgent revascularization** | 2.77% | 2.81% | 1.26% | 1.39% | 1.40% | 0.63% | 0.50 |
| **BARC 2 bleeding** | 7.85% | 8.18% | 2.13% | 7.48% | 7.77% | 2.02% | 0.95 |
| **BARC 3a bleeding** | 3.22% | 3.28% | 1.34% | 3.06% | 3.11% | 1.28% | 0.95 |
| **BARC 3b bleeding** | 1.07% | 1.07% | 0.77% | 1.01% | 1.02% | 0.73% | 0.95 |
| **BARC 3c bleeding** | 1.00% | 1.01% | 0.50% | 0.95% | 0.95% | 0.47% | 0.95 |

| | | | |
|---|---|---|---|
| 12.3 Statistical Methods | 50/51 | This is a non-blinded, randomized, multicentre trial. ~~We use random effect (to adjust for centres) logistic regression (to adjust for treatment and strata) to analyse the data. We will use hierarchical testing to assess the primary endpoints (efficacy and safety).~~ We will use hierarchical testing to assess the primary endpoints (efficacy and safety). The efficacy composite endpoint includes (i) death, (ii) definite or probable stent | Description of the method used to analyze the combined endpoint of the |

| | | | |
|---|---|---|---|
| | | thrombosis, (iii) myocardial infarction, (iv) stroke, (v) systemic embolism, and (vi) urgent revascularization. The safety composite endpoint includes (i) death, (ii) BARC 3c bleeding, (iii) BARC 3b bleeding, (iv) BARC 3a bleeding, and (v) BARC 2 bleeding. The endpoints will be tested using a hierarchical design with superiority testing of the first endpoint and non-inferiority testing of the second endpoint, if superiority of the first endpoint is met. Both composite endpoints will be analyzed using Finkelstein and Schoenfeld test (win-loss-ratio; [WLR] (18) using a decreasing ranking.<br><br>We will calculate the WLR using an unmatched approach with application of bootstrapping (boot.n = 1.000 iterations) for calculation of SE and 95% confidence intervals (21). The results will be presented using forrest plots.<br><br>[…] | study (win-loss ratio) |
| 21 References | 62 | 20. Pocock SJ, Gregson J, Collier TJ, Ferreira JP, Stone GW. The win ratio in cardiology trials: lessons learnt, new developments, and wise future use. Eur Heart J 2024;45:4684-4699.<br>21. Pocock SJ, Ariti CA, Collier TJ, Wang D. The win ratio: a new approach to the analysis of composite endpoints in clinical trials based on clinical priorities. Eur Heart J 2012;33:176-82. | Addition of reference 20 and 21 |

LMU KLINIKUM

Deutsches Herzzentrum München
des Freistaates Bayern
Klinik an der Technischen Universität München

Prof. Dr. med. Steffen Massberg
Direktor der Klinik
LMU Klinikum
Medizinische Klinik und Poliklinik I
Ziemssenstraße 5 · 80336 München

19 Mar 2025

**Date** (DD-Mmm.-YYYY): **Signature** / Prof. Dr. med. Steffen Massberg (Sponsor Delegated Person)

Prof. Dr. med. Konstantinos Rizas
Leitung Campus Innenstadt
LMU Klinikum
Medizinische Klinik und Poliklinik I
Ziemssenstraße 5 · 80336 München

19 Mar 2025

**Date** (DD-Mmm.-YYYY): **Signature** / Prof. Dr. med. Konstantinos Rizas (Coordinating Investigator)
