# Supplementary material for: Dual antithrombotic therapy using potent antiplatelet inhibitors in atrial fibrillation and acute coronary syndrome: a randomized controlled trial
Source: Nat Med. 2026 Aug 29;32(11):4036–46. doi: 10.1038/s41591-026-04629-7 (PMC13768177; doi:10.1038/s41591-026-04629-7)
Supplement: Supplementary file 5 [file 41591_2026_4629_MOESM5_ESM.pdf]

Statistical Analysis Plan

STUDY TITLE: Escalated single Platelet Inhibition for one month plus Direct oral anticoagulation in patients with Atrial fibrillation and acUte coRonary syndrome undergoing percutaneoUS coronary intervention (EPIDAURUS)[1]

EPIDAURUS

Trial Registration Number	Protocol Version	SAP Version
EudraCT 2020-004748-27	Version 5.0 (23 Dec-2021)	1.0, Date: Dec 1, 2022

SAP Author	SAP Reviewer
Amani Al Tawil, MSc<br>Prof. Dr. med. Konstantinos Rizas	Prof. Dr Ulrich Mansmann

COORDINATING INVESTIGATOR

PD Dr. med. Konstantinos Rizas
LMU University Hospital Munich
Department of Cardiology
Marchioninistr. 15, 81377 Munich, Germany

TRIAL STATISTICIAN

Prof. Dr Ulrich Mansmann
Amani Al Tawil, MSc
IBE - Institut für Medizinische Informationsverarbeitung, Biometrie und Epidemiologie
Ludwig-Maximilians-Universität München
Marchioninistr. 15, 81377 München

[1] This document has been written based on the information contained in the study protocol Version 5.0 dated 23 Dec-2021

Approval of SAP and Signature Page

We the undersigned have carefully read this statistical analysis plan (SAP) Version 1.0 and hereby agree to the described methods and proceedings.

U. Mansmann

01-Dec-2022

Prof. Dr. Ulrich Mansmann
Responsible Biometrician

Date

01-Dec-2022

PD Dr. med. Konstantinos Rizas
Coordinating Investigator

Date

Table of Contents

List of Tables 4
Abbreviation and Definition 5
1 Introduction 7
1.1 Preface 7
1.2 Background and Rationale 7
1.3 Purpose of the Analysis 9
1.4 Study Objectives 9
1.4.1 Primary Objective and Outcome Measure 9
1.4.2 Secondary Objectives and Outcome Measures 10
1.4.3 Additional Event Evaluation 10
2 Study Methods 10
2.1 Trial Design 10
2.2 Inclusion and Exclusion Criteria 11
2.3 Subject Information and Recruitment Status 12
2.4 Randomization 12
2.5 Trial Procedures 13
2.6 Sample Size 15
2.7 Ethical Aspects: Good Clinical Practice 16
3 General Considerations 17
3.1 Timing of Final Analysis 17
4 Analysis Sets 17
4.1 Definition of Analysis Sets 17
4.2 Violations and Deviations 18
4.2.1 Deviation from the Protocol 18
4.2.2 Protocol Violations 18
5 Primary and Secondary Variables 19
5.1 Primary Variable 19
5.2 Secondary Variables 19
5.2.1 Component of the primary endpoint: all-cause mortality at 6 weeks after randomization 19
5.2.2 Component of the primary endpoint: myocardial infarction at 6 weeks after randomization 20
5.2.3 Component of the primary endpoint: definite or probable stent thrombosis at 6 weeks after randomization 20
5.2.4 Component of the primary endpoint: ischaemic stroke at 6 weeks after randomization 20
5.2.5 Component of the primary endpoint: systemic thromboembolism at 6 weeks after randomization 20
5.2.6 Cardiovascular mortality at 6 weeks after randomization 21
5.2.7 Bleeding (BARC type ≥ 2) at 6 weeks after randomization (superiority testing) 21
5.2.8 Urgent revascularization at 6 weeks after randomization 21
5.2.9 All-cause mortality at 6 months after randomization 21
5.2.10 Unplanned hospitalization due to acute heart failure or acute coronary syndrome at 6 months after randomization 21
5.2.11 Ischaemic stroke at 6 months after randomization 21
5.3 Additional Event Evaluation 22
6 Analysis Methods 22
6.1 General Principles 22

6.1.1 Consort Diagram ... 22
6.1.2 Stratification of Analyses ... 22
6.1.3 Incomplete Dates ... 23
6.1.4 Estimand for primary Outcomes ... 23
6.1.5 Hypothesis ... 23
6.1.6 Presentation of event analysis ... 24
6.2 Analysis Method ... 24
6.2.1 By-patient Presentations ... 24
6.2.2 Baseline Value ... 24
6.2.3 Descriptive Summaries of Continuous Variables ... 28
6.2.4 Descriptive summaries of categorical variables ... 28
6.2.5 Eligibility ... 28
6.2.6 Withdrawal/Follow-up ... 28
6.2.7 Analysis of the primary efficacy Variable ... 29
6.2.8 Secondary Endpoints ... 30
6.2.9 Subgroup Analysis ... 31
6.2.10 Sensitivity analysis of the primary efficacy and safety endpoint ... 32
6.2.11 Additional Analysis ... 32
6.2.12 Analysis of Safety ... 33
7 Statistical Software ... 33
References ... 33

List of Tables

Table 1. Schedule of Events ... 16
Table 2: Definition of population analysis sets ... 18
Table 3: characteristics of patients randomized to the experimental arm versus control arm ... 25

Abbreviation and Definition

ACEI	Angiotensin Converting Enzyme Inhibitor
ACS	Acute Coronary Syndrome
AF	Atrial Fibrillation
ARB	Angiotensin II Receptor Blocker
ARNI	Angiotensin Receptor Neprilysin Inhibitor
BARC	Bleeding Academic Research Consortium
BNP	Brain Natriuretic Peptide
BUN	Blood Urea Nitrogen
CABG	Coronary Artery Bypass Graft
CAD	Coronary Artery Disease
CCB	Calcium Channel Blockers
CCS	Canadian Cardiovascular Society
CK	Creatine Kinase
COPD	Chronic Obstructive Pulmonary Disease
CRF	Chronic Renal Failure
CRP	C-Reactive Protein
CRT	Cardiac Resynchronization Therapy
CSR	Clinical Study Report
CVD	Cerebrovascular Disease
DAT	dual antithrombotic therapy
DAPT	Dual Antiplatelet Therapy
DM	Data Management
DRM	Data Review Meeting
EAC	Event Adjudication Committee
EC	Ethics Committee
eCRF	Electronic Case Report Form
EDC	Electronic Data Capture
EU	European Union
FAS	Full Analysis Set
GFR	Glomerular Filtration Rate
GOT	Aspartate Aminotransferase
GPT	Alanine Aminotransferase
HbA1C	Hemoglobin A1C
HDL	High Density Lipoprotein
HF	Heart Failure
HR	Hazard Ratio
IBE	Institute for Medical Information Processing Biometry and Epidemiology
ICD	Implantable Cardioverter Defibrillator
ICH	International Conference on Harmonization
ICH E3	International Conference on Harmonization-Structure and Content of Clinical Study Reports
ICH E6	International Conference on Harmonization-Guideline For Good Clinical Practice
ICH E9	International Conference on Harmonization-Statistical Principles for Clinical Trials

ICH E9(R1)	International Conference on Harmonization-Addendum on Estimands and Sensitivity Analysis in Clinical Trials
INR	International Normalized Ratio
LCA	Left Coronary Artery
LDH	Troponin, Lactate Dehydrogenase
LDL	Low-Density Lipoprotein
LVEF	Left-Ventricular Ejection Fraction
MI	Myocardial Infarction
NOAC	New Oral Anticoagulant
NSAIDS	Non-Steroidal Anti-Inflammatory Drugs
NSTEMI	Non-St-Segment Elevation Myocardial Infarction
NT-proBNP	N-Terminal Fragment Of The BNP-Prohormone
NYHA	New York Heart Association
OAC	Oral Anticoagulants
PAOD	Peripheral Arterial Occlusive Disease
PCI	Percutaneous Coronary Intervention
PP	Per-Protocol
PV	Protocol Violations
SAP	Statistical Analysis Plan
SGLT2I	Sodium-Glucose-Cotransporter 2 Inhibitors,
SOP	Standard Operating Procedure
STEMI	St-Segment Elevation Myocardial Infarction
TAT	Triple Antithrombotic Therapy
VKA	Vitamin K Antagonists
WoC	Withdrawal of Consent

1 Introduction

1.1 Preface

This statistical analysis plan (SAP) describes the study design and statistical analyses for study protocol EPIDAURUS, entitled "Escalated single Platelet Inhibition for one month plus Direct oral anticoagulation in patients with Atrial fibrillation and acUte coRonary syndrome undergoing percutaneoUS coronary intervention".

This SAP has been prepared to support a clinical study report (CSR) that will be included in future regulatory submissions. CSR analyses, as directed by this document, may also be used for other purposes such as publication of study results. Changes from analyses planned in the study protocol will be accounted for in this document. Analysis specifications in this document supersede suggestions for or descriptions of analyses contained in other documents.

By writing this SAP we aim to ensure the credibility of the study findings by pre-specifying the statistical approaches to the analysis of the study data before hard locking the database of the EPIDAURUS trial data. This SAP is presented to avoid post hoc decisions that may influence the interpretation of the results and the statistical analyses of the final data[1], as well as preventing outcome bias and selective reporting. This SAP is a technical extension of the EPIDAURUS study protocol (V 5.0, Date: December 23, 2021). **Web appendix 1** shows the trial protocol; This SAP follows the principles of the International Conference on Harmonization (ICH) guidelines E3, E6, E9 and E9(R1). This SAP is a comprehensive and detailed description of the strategy, rationale and statistical techniques that will be used to assess whether an escalated antiplatelet therapy with a potent P2Y12-inhibitor for one month can reduce ischemic events without a significant increase in bleeding complications in patients with Atrial Fibrillation (AF) and ST-Segment Elevation Myocardial Infarction (STEMI) or Non-ST-Segment Elevation Myocardial Infarction (NSTEMI)

Statistical analyses will be performed using R-Studio and CRAN-R. Validation of R-programming will be performed to establish a high degree of assurance that our analysis process will consistently produce a product meeting the predetermined specifications and quality attributes. For this, we will randomly identify a list of variable measures and check the consistency and quality of outputs.

1.2 Background and Rationale

The selection of the optimal antithrombotic therapy in patients with nonvalvular AF and acute coronary syndrome (ACS) undergoing percutaneous coronary intervention (PCI) is challenging. Dual antiplatelet therapy (DAPT) with Aspirin and a P2Y12-inhibitor has been proven to reduce recurrent cardiovascular events in patients undergoing PCI, but is less effective in reducing the incidence of ischaemic stroke related to AF[2]. On the other hand, oral anticoagulants (OAC) are the standard therapy to prevent stroke and systemic embolism in patients suffering from AF, but are not indicated as secondary prevention after PCI[3]. Until recently, triple antithrombotic therapy (TAT) consisting of Aspirin plus Clopidogrel plus OAC was considered the treatment of choice. While efficiently preventing ischaemic events, TAT is associated with an increase in bleeding complications. In the past years several randomized controlled trials (PIONEER AF-PCI, RE-DUAL

PCI, ENTRUST-AF PCI, and AUGUSTUS) challenged TAT by comparing a TAT-regimen based on Vitamin K antagonists (VKA) to a dual antithrombotic therapy (DAT)-regimen based on new oral anticoagulants (NOACs) and P2Y12-inhibitors, mainly Clopidogrel in patients with AF undergoing PCI[4–7].

Based on the results of the PIONEER AF-PCI, RE-DUAL PCI, ENTRUST-AF PCI, and AUGUSTUS trials, which consistently showed a significant decrease in bleeding complications without detecting a significant increase in ischaemic events, current guidelines were revised and now recommend an in-hospital TAT followed by DAT based on NOAC in patients with an indication for oral anticoagulation undergoing PCI[8]. However, all the above trials were powered to show a superiority of a NOAC-based DAT to the VKA-based TAT with regard to bleeding events. In contrast, all analyses regarding ischaemic events were exploratory and not sufficiently powered. Moreover, the patients at high-risk of ischaemic complications with biomarker positive ACS (STEMI or NSTEMI) undergoing PCI were underrepresented in all studies.

In a recent meta-analysis of all four trials, Gargiulo et al. could show that the decrease in bleeding complications with DAT compared to TAT comes at the expense of a significantly higher risk of stent thromboses (Hazard Ratio (HR) 1.59, 95% CI 1.01 - 2.50, $p = 0.04$) and a borderline increase in the risk of myocardial infarction (MI) (HR 1.22, 95% CI 0.99 - 1.52, $p = 0.07$) [9]. As presented in the post-hoc analysis of the AUGUSTUS trial, these ischaemic events primarily occurred within the first month after the index PCI[10].

Activation of blood platelets plays a key role during the early phase of an ACS. Approximately 30-40% of patients show low response to Clopidogrel and are not adequately protected against ischaemic events, in particular when presenting with ACS[11]. Consequently, clinical outcomes of patients with ACS undergoing PCI without AF have been significantly improved with the use of the potent P2Y12 - receptor inhibitors Prasugrel or Ticagrelor.

It is therefore reasonable to assume that a more potent platelet inhibition within the first month after PCI might reduce the rate of ischaemic complications observed in AF patients with ACS undergoing PCI, when receiving DAT. On the other hand, a subsequent de-escalation to a less potent platelet inhibition one month after PCI might prevent an increase in bleeding complications. This hypothesis is supported by a landmark analysis of the TRITON-TIMI-38 trial[12] which showed that the beneficial effect of Prasugrel compared to Clopidogrel with regard to ischaemic events was mainly exerted within the first days after the index-MI, while the bleeding complications progressively increased over time with non-significant differences in bleeding rates within the first month of treatment with Prasugrel vs. Clopidogrel.

In EPIDAURUS we will therefore test the hypothesis that DAT using NOAC plus an escalated antiplatelet therapy with a potent P2Y12-inhibitor for one month followed by Clopidogrel reduces ischaemic events without a relevant increase in bleeding complications in AF patients with STEMI or NSTEMI undergoing PCI compared to standard in-hospital TAT followed by DAT with NOAC plus Clopidogrel.

1.3 Purpose of the Analysis

The statistical analyses described in this SAP will test whether an escalated antiplatelet therapy with a potent P2Y12-inhibitor for one month can reduce ischaemic events without a significant increase in bleeding complications in patients with AF and STEMI or NSTEMI undergoing successful completion of percutaneous coronary intervention. The statistical analyses described in this SAP will be included in the final clinical study report or a peer-reviewed publication.

1.4 Study Objectives

[ICH E3; 8]

1.4.1 Primary Objective and Outcome Measure

Study hypothesis

We hypothesize that an escalated antiplatelet therapy with a potent P2Y12-inhibitor for one month can reduce ischaemic events without a significant increase in bleeding complications in patients with AF and STEMI or NSTEMI undergoing successful completion of PCI versus the standard regimen

The null hypotheses H_0

Null hypothesis for primary efficacy endpoint: There is no difference in the number of major ischaemic events between the two study interventional arms.
Null hypothesis for primary safety endpoint: The experimental and the control arm are not equivalent in terms of bleeding type 2 or higher according to the Bleeding Academic Research Consortium (BARC) criteria.

Primary Objective	Primary Outcome Measure
The primary objective of the EPIDAURUS trial is to test whether an escalated antiplatelet therapy with a potent P2Y12-inhibitor for one month can reduce ischaemic events without a significant increase in bleeding complications in patients with AF and STEMI or NSTEMI	Primary Efficacy Endpoint:<br>Incidence of major ischaemic events defined as the composite of all-cause mortality, MI, definite or probable stent thrombosis, ischaemic stroke or systemic thromboembolism (superiority test) at 6 weeks after randomization.<br><br>Primary Safety Endpoint:<br>Bleeding type 2 or higher according to the BARC criteria (non-inferiority test) at 6 weeks after randomization

1.4.2 Secondary Objectives and Outcome Measures

Secondary Efficacy Objectives	Secondary Outcome Measures
Incidence of:<br>• The individual components of the primary endpoint (all-cause mortality, MI, definite or probable stent thrombosis, ischaemic stroke, systemic thromboembolism) at 6 weeks after randomization<br>• The individual components of the primary endpoint (all-cause mortality, MI, definite or probable stent thrombosis, ischaemic stroke, systemic thromboembolism and urgent revascularization) at 6 months after randomization<br>• Cardiovascular mortality at 6 weeks and 6 months after randomization<br>• Urgent Revascularization at 6 weeks and 6 months after randomization.<br>• Unplanned hospitalization due to acute heart failure (HF) or ACS at 6 months after randomization	Incidence of:<br>• All-cause mortality at 6 weeks and 6 months after randomization<br>• Bleeding BARC type ≥ 3 at 6 weeks and 6 months after randomization.<br>• Bleeding BARC type ≥ 2 at 6 weeks and 6 months after randomization

1.4.3 Additional Event Evaluation

Any bleeding events (BARC type ≥ 1) at 6 weeks and 6 months after randomization

2 Study Methods

2.1 Trial Design

[ICH E3; 9]

The EPIDAURUS study is a phase IV, multinational European, multicenter, prospective, randomized, open-label, two-arm, parallel-group clinical trial designed to test whether an escalated antiplatelet therapy with a potent P2Y12-inhibitor for one month can reduce ischaemic events without a significant increase in bleeding complications in patients with AF and STEMI or NSTEMI undergoing PCI. It is planned that the trial is performed in a minimum of 2 countries within the European Union (EU). We anticipate that 50-60 sites will participate in this study

This study uses a fixed sample design with a target sample size of 2334 randomized patients. Closeout of the trial will commence after all randomized individuals have completed a minimum follow up of 6 months required to test the different hypotheses.

Patients with ACS and AF undergoing PCI with an indication for oral anticoagulation with NOAC, after agreement to participate in the clinical trial will be 1:1 randomized to either a standard antiplatelet therapy with Clopidogrel (control arm) or an escalated antiplatelet therapy with a potent P2Y12-inhibitor (experimental arm) through the use of a permuted-block randomization method to ensure a balanced assignment to each treatment arm. Randomization will be stratified by centre.

EPIDAURUS is a trial of the clinical efficacy of an escalated antiplatelet therapy after a minimum of six months' follow-up period and comprises two arms:

1- The experimental arm consists of intensified regimen of NOAC plus a potent P2Y12-inhibitor for the first month followed by NOAC and Clopidogrel for 5 months.
2- The control arm of the study consists of a standard therapy with an in-hospital TAT (Aspirin, Clopidogrel and NOAC (for a maximum of one week) and a subsequent DAT (NOAC plus Clopidogrel) for 6 months

2.2 Inclusion and Exclusion Criteria

[ICH E3;9.3]

This section is intended to describe details about all the subjects in the study. It is distinct from the Analysis Population in section **4.1**. This section is intended to describe the intended characteristics of ***all*** the subjects in the study. If a subject appears to be eligible for the trial according to the below-detailed criteria, the investigator will inform the subject about the trial and ask the subject for his/her written consent. It is a requirement that written consent is obtained before any trial-specific procedures. The investigator will then record the details of the eligible subjects on the trial-specific lists provided.

Inclusion Criteria: *[ICH E3; 9.3.1]*
Subjects must meet ALL of the following inclusion criteria to be eligible for enrolment into the trial:

- Written informed consent
- Age ≥ 18 years
- AF requiring oral anticoagulation
- STEMI or NSTEMI (biomarker positive acute coronary syndrome) and successful completion of PCI (randomization will take place within 24h after successful PCI)

Exclusion Criteria: *[ICH E3; 9.3.2]*

Subjects presenting with any of the following exclusion criteria may not be included in the trial:

- Chronic renal insufficiency with glomerular filtration rate (GFR) < 15 ml/min/1.73m2
- History of ischaemic stroke or transient ischaemic attack (both contraindications for Prasugrel) and history of intracranial bleeding (contraindication for Ticagrelor)
- Contraindication for Clopidogrel or Aspirin
- Contraindication for Prasugrel and Ticagrelor
- Severe chronic liver disease (Child-Pugh C)
- Indication for oral anticoagulation with VKA
- Moderate to severe mitral stenosis or mechanical heart valve
- Any bleeding BARC type ≥ 2 within the last 4 weeks before index procedure
- Pregnancy or lactation
- Inability to cooperate with the protocol requirements
- Life expectancy < 6 months
- Participation in another investigational drug study
- Previous enrolment in this study
- For women of childbearing potential, no negative pregnancy test and no agree to use a reliable method of birth control during the study
- Previous treatment with GP IIb/IIIa inhibitors within the last 12 hours
- A known genetic disorder involved in the metabolism of the study medication

2.3 Subject Information and Recruitment Status

Prior to enrolment in the trial, the investigator will inform the patient orally and in writing about scope and purpose, and possible risks / benefits of the trial as well as his/her rights and duties in a lay language. Patients must be informed that their records will be subject to review by monitors, sponsor and regulatory agency representatives. Patients must be assured that they may withdraw from the trial at any time for any reason and without prejudice to their future medical care by the physician or at the institution

Patients presenting with STEMI or NSTEMI to the participating hospital after successful completion of the PCI (defined as TIMI flow grade 2 or more after intervention), who meet all inclusion and no exclusion criteria are eligible for enrolment. Screening is performed within 24 hours after completion of PCI. Pre-screening lists will be obtained at the trial sites to document patients assessed for eligibility, but not enrolled in the trial.

2.4 Randomization

[ICH E3; 9.4.3. ICH E9; 2.3.2)

A randomized parallel group design to control for confounding is used. Patients will be randomized within 24h after completion of successful PCI (transradial approach preferred). Participants will be randomly assigned to one of two treatment arms. Randomization will occur in a 1:1 ratio through use of a permuted-block randomization method (block size chosen as 8) to ensure a balanced assignment to each treatment arm. Randomization will be stratified by centre. The randomization list will be uploaded into the randomization module of the EDC system. Study investigators will have access to

the randomization module via the eCRF and randomize participants using this central tool. The study investigators, sponsors and statisticians have no access to the randomization list

2.5 Trial Procedures

[ICH E3; 9.5.1. ICH E9; 2.2.2]

Screening / Baseline Visit

It is the responsibility of the investigator, or a person delegated by the investigator to obtain written informed consent from each patient prior to participation in the trial, following adequate explanation of the aims, methods, anticipated benefits and potential hazards of the trial.

No clinical trial procedures will be conducted prior to taking consent from the participant. The consent will not denote the enrolment into the trial. Data obtained as part of the normal patient care can be used for screening/baseline visit if collected within 72h prior to randomization.

The following procedures are performed:

- Informed consent
- Assessment of in- and exclusion criteria
- Demographic data (age, gender, race), general and cardiovascular medical history and current diseases:
  - Symptoms: angina pectoris (CCS classification), dyspnoea (NYHA classification), syncope
  - Cardiovascular risk factors: Smoking status, arterial hypertension, diabetes mellitus, hypercholesterolaemia, family history of coronary artery disease
  - Cardiovascular history: Previous myocardial infarction, previous PCI, previous coronary artery bypass graft (CABG) surgery, previous stroke, previous stent thrombosis, previous bleeding BARC ≥ 2 (within the last 4 weeks before index procedure), peripheral arterial occlusive disease (PAOD), cerebrovascular disease (CVD), atrial fibrillation, aortic stenosis, aortic insufficiency, mitral insufficiency, mitral stenosis, tricuspid insufficiency, pacemaker/implantable cardioverter defibrillator (ICD) or /cardiac resynchronization therapy (CRT).
  - Medical history: chronic obstructive pulmonary disease (COPD), chronic renal failure (CRF) and stage, abnormal liver function, history of cancer, alcohol abuse
- Clinical parameters: Weight and height at admission, blood pressure and heart rate post PCI at screening, ECG at admission and post-PCI at screening, ECG autonomic risk stratification (optional)
- Laboratory parameters (admission): Laboratory tests at admission depend on the routine tests performed in each study centre. We recommend following tests: Haemoglobin, erythrocytes, leukocytes, thrombocytes, creatinine, blood-urea-nitrogen (BUN), creatine kinase (CK), troponin, lactate dehydrogenase (LDH), C-reactive protein (CRP), brain-natriuretic peptide (BNP) and /N-terminal fragment of the BNP-prohormone (NT-proBNP), Hemoglobin A1C (HbA1c), total cholesterol, low-density lipoprotein (LDL), high-density lipoprotein (HDL), aspartate aminotransferase (GOT), alanine aminotransferase (GPT), bilirubin, international normalized ratio (INR), platelet aggregation tests (substudy at pre-defined sites)

- Laboratory parameters (post PCI and prior to randomization): Laboratory tests post PCI depend on the routine tests performed in each study centre. We recommend following tests: Haemoglobin, CK, troponin.
- Pregnancy test for women of childbearing potential, contraception (if applicable)
- Concomitant medication will be recorded:
    - At admission: Aspirin, P2Y12-inhibitors, OAC, beta-blocker, angiotensin-converting-enzyme inhibitors (ACEI), angiotensin II receptor blockers (ARB), aldosterone antagonist, angiotensin-receptor-neprilysin-inhibitor (ARNI), sodium-glucose-cotransporter 2 (SGLT2) inhibitors, amiodarone, digitalis, calcium channel blockers (CCB), nitrates, statins, non-steroidal anti-inflammatory drugs (NSAIDs)
    - Peri-interventional: Aspirin, P2Y12-inhibitors, heparin, GP IIb/IIIa inhibitors
    - At screening: Aspirin, P2Y12-inhibitors, OAC, beta-blocker, ACEI/ARB, aldosterone antagonist, ARNI, SGLT2 inhibitors, amiodarone, digitalis, CCB, nitrates, statins
- Coronary angiography/PCI: coronary artery disease (CAD) and vessels involved, PCI number of stents, PCI bifurcation, kissing balloon, PCI ostial, PCI left coronary artery (LCA), PCI CABG, PCI planned and which vessels, planned bifurcation, planned ostial stenosis, planned LCA, planned CABG, success of PCI (TIMI grade 2 or more), left-ventricular ejection fraction (LVEF; angiographic or echocardiographic)
- Randomization within 24h after successful completion of PCI with TIMI flow grade 2 or more: All patients who meet the inclusion and none of the exclusion criteria are randomized 1:1 into two groups:

Group 1 (experimental): escalated antiplatelet therapy with a potent P2Y12-inhibitor (Prasugrel or Ticagrelor):
Concomitant antiplatelet treatment with Aspirin will be discontinued in this group.

Group 2 (control): treatment with Clopidogrel:
Aspirin will be continued at a maintenance dose of 75-100mg/day (for Germany and Austria only 100 mg are applicable) for the in-hospital stay for a maximum of 7 days in the control group. According to the current guidelines, Aspirin is allowed to be continued for a maximum of 4 weeks in patients with high ischaemic risk.

In both groups:
If the patients are already on treatment with an anti-Xa-inhibitor (Edoxaban, Apixaban or Rivaroxaban) the anti-Xa-inhibitor will be continued.
In patients not on treatment with NOACs or in patients on treatment with VKA or anti-IIa-inhibitor, an anti-Xa-inhibitor will be initiated (preferentially Edoxaban).

Visit at Discharge

- Laboratory parameters: CK max, troponin max
- Assessment of adverse events and occurrence of endpoints
- Concomitant medication at discharge: Aspirin, P2Y12-inhibitors, OAC, beta-blocker, ACEI/ARB, aldosterone antagonist, ARNI, SGLT2 inhibitors, amiodarone, digitalis, CCB, nitrates, statins

Control Visit (Day 42 ± 7)
The visit can be performed either in the hospital, by phone or letter (for patients not included in the sub study).

- Assessment of adverse events and occurrence of endpoints
- Symptoms: angina pectoris (CCS classification), dyspnoea (NYHA classification), syncope
- Concomitant medication: Aspirin, P2Y12-inhibitors, OAC, beta-blocker, ACEI/ARB, aldosterone antagonist, ARNI, SGLT2 inhibitors, amiodarone, digitalis, CCB, nitrates, statins
- Laboratory: Laboratory parameters that were assessed after discharge of index hospital stay (if applicable)

Final Visit (Day 180 ± 10)
The visit can be performed either in the hospital, by phone or letter.

- Assessment of adverse events and occurrence of endpoints
- Concomitant medication: Aspirin, P2Y12-inhibitors, OAC
- Smoking status

The procedures to be performed throughout the study are outlined in the schedule of events in

Table 1.

2.6 Sample Size

[ICH E3; 9.7.2. ICH E9; 3.5]

Assumptions for sample-size calculation (hierarchical testing):

- Primary efficacy endpoint (ischaemic events) at 6 weeks:
  Superiority testing, 2-sided alpha 5%, power 80%, 6-weeks event-rate in the control group 7% (ISAR-REACT 5 study, data not published), 6-weeks event-rate in the intervention group 4.3%, corresponding to an odds-ratio of 0.6 (7,14).
  Number per group: 1147

- Primary safety endpoint (bleeding BARC ≥ 2) at 6 weeks:
  Non-inferiority testing, a two-group large-sample normal approximation test of proportions with a one-sided 2.5% significance level will have 80% power to reject the null hypothesis that the test and the standard are not equivalent (6-weeks event-rate in the control group 13.1% (ISAR-REACT 5 study, data not published), odds-ratio between experimental and control group 0.95, difference in proportions, $\pi_1 - \pi_0$, is -0,033 or farther from zero in the same direction) in favor of the alternative hypothesis that the proportions in the two groups are equivalent.
  Number per group: 1152

The sample size of the trial is given by the maximum of both sample sizes: 1152 per group.

The computation assumed an allocation ratio $\frac{ncontrol}{nexperimental} = 1$, an accrual time of 24 months, a follow-up time of 6 months and an annual 10% follow-up drop-out rate (6-week rate 1.25%, 2,304 * 1.0125 = 2,334).

Table 1. Schedule of Events

	Screening/ Baseline visit Day 0	Discharge visit	Control visit Day 42 ± 7	Final visit Day 180 ± 10
Informed Consent	X			
In-/Exclusion Criteria	X			
Demographic data	X			
Patient history	X			
Clinical parameters	X			
Laboratory[1]	X		X[2]	
Taking of blood sample for bio banking	X		X	
Concomitant medication	X	X		X
Randomization	X			
AEs und SAEs	X	X	X	X
Primary efficacy endpoint		X	X	
Primary safety endpoint		X	X	
Cardiovascular mortality		X	X	
MI		X	X	
Ischemic stroke		X	X	X
Stent thrombosis		X	X	
Systemic thromboembolism		X	X	
Urgent revascularization		X	X	
BARC bleeding ≥ 1		X	X	
All-cause mortality		X	X	X
Unplanned hospitalization due to acute heart failure or acute coronary syndrome			X	X

1 Beyond clinical routine, platelet function testing will be performed.
2 Not obligatory

2.7 Ethical Aspects: Good Clinical Practice

The clinical trial will be performed in compliance with the protocol, the Declaration of Helsinki (2013), ICH E6 (R2), Directive 2001/20/EC and Regulation (EU) No 536/2014, as applicable, and applicable local laws and regulations. Personnel involved in conducting this clinical trial is qualified by education, training, and experience to perform their respective task(s).

3 General Considerations

3.1 Timing of Final Analysis

All outcomes of the EPIDAURUS trial will be analyzed collectively after the Data Review Meeting (DRM).

All final analyses will be performed on the derived database. The following prerequisites before analysis have to be fulfilled:

- DRM
- Data cleaning and resolution of all queries concerning CRF
- Finalization and approval of this SAP document.

All these processes before database locking must take place to comply with the requirements documented in the IBE-SOP DM07, DM08 and DM11. Following data integrity checks the database will be locked before the statistical analyses specified in the SAP will be performed.

4 Analysis Sets

(ICH E3; 9.7.1. ICH E9; 5.2)

4.1 Definition of Analysis Sets

The primary analysis (efficacy) will be based on the intention to treat (ITT) principle comprising all randomized subjects[1]. In this efficacy analysis, a full analysis set (FAS) which is as close as possible to the ITT will be used in the primary analysis to avoid over-optimistic estimates of efficacy resulting from a per-protocol (PP) analysis. The safety endpoints will be analyzed according to modified intention to treat principle (mITT) applied in a per-protocol set (PPS), as shown in **Table 2.**

The analysis populations of the EPIDAURUS trial will be specified during the DRM. This includes how the analysis populations will be defined and which outcomes will be analysed according to each analysis population set, as shown in **Table 2**.

The ITT population will include all patients randomized (irrespective of whether they received the intervention or not). All patients who have been randomized to study treatment will be included irrespective of whether they received the intervention, followed the protocol or continued participation in the study. Patients will be analyzed according to their randomized assignment (not to which intervention they actually received) irrespective of whether the event occurred before or following discontinuation of the study intervention. Patients who withdraw consent to participate in the study (or are lost to follow-up) will be included up to the date of their study termination (if applicable) except for vital status known through public records (for use in the analyses of deaths).

Modified ITT analysis (safety analysis) will be done on patients of the ITT who received treatment without protocol deviation (PPS) judged before database lock to affect the interpretability of the safety endpoint.

The as-treated population will include all randomized patients classified according to the treatment strategy actually received and will be used only for sensitivity analyses.

All efficacy secondary and exploratory endpoints will be analyzed according to the ITT principle in the FAS and all safety secondary and exploratory endpoints will be analyzed according to the mITT principle in the PPS.

Table 2: Definition of population analysis sets

Analysis Population	Study Medication
**Intention to treat**	The FAS population includes all patients randomized and will be analyzed according to the randomized assignment.
**Modified intention to treat (safety)**	Modified ITT includes only ITT patients who received treatment according to protocol (PPS).<br>• Patients randomized to intervention but not treated will be excluded from the analysis.<br>• Patients randomized to control group and received intervention will be excluded from the analysis.
**As treated**	As treated set includes all subjects from the ITT analyzed according to the treatment received (FAS):<br>• Patients randomised but not treated will be classified as treated in the control arm.<br>• Patients randomised to the control group and treated will be analysed as treated in the experimental arm.

4.2 Violations and Deviations

4.2.1 Deviation from the Protocol

As per the protocol, during the clinical trial any amendment or modification to the clinical trial protocol must be submitted to the Competent Authorities and ethics committees (ECs). The investigator should not implement any deviation or change to the protocol without prior review and documented approval/favourable opinion from the EC of an amendment, except where necessary to eliminate an immediate hazard(s) to study subjects. Any deviation must be documented in the eCRF.

4.2.2 Protocol Violations

There were no pre-defined major protocol violations (PV) with direct bearing on the primary outcome. All PV will be listed with patient ID, centre, date of the PV, description of the PV, cause, evaluation, procedure and reason. Those regarded as major deviations with impact on a confirmatory

statistical analysis will be coded for handling in the respective statistical analysis (i.e. will not be a part of the as-treated analysis).

5 Primary and Secondary Variables

The Event Adjudication Committee (EAC) will adjudicate the primary efficacy and primary safety endpoints in order to provide a standardized, systematic, independent and unbiased assessment. The three members will review the events reported by the investigators as endpoints to determine whether they meet the specified criteria of endpoint definition stated in the protocol. **Appendix 1** in the protocol shows the list of endpoint definitions used as a reference tool. The additional safety endpoint bleeding events BARC type ≥ 1 at 6 weeks are not subject of EAC adjudication. During event adjudication, members of the committee are blinded to the randomized treatment received by the patient.

For analysis of the event rate, outcome data will be expressed as**:**
• A binary variable dichotomized into "unfavourable" (occurrence of any of the major ischaemic events/bleeding) versus "favourable" (non-occurrence of any of the major ischaemic events/bleeding).

5.1 Primary Variable

The primary efficacy variable is the occurrence of any event in the composite of major ischaemic events: all-cause mortality, myocardial infarction, definite or probable stent thrombosis, ischaemic stroke or systemic thromboembolism (superiority test) at 6 weeks after randomization.

The primary safety variable is the occurrence of bleeding type 2 or higher according to the BARC criteria (non-inferiority test) at 6 weeks after randomization

The EAC members who were blinded to the randomized treatment received by the patient has defined clinical efficacy end-point events according to the protocol definitions with the minimum amount of source data required, to evaluate pre-specified end-point events reported by the investigators.

For our supplementary/additional analysis (time to event) analysis, patients who did not have an endpoint event will be censored at the earliest date of withdrawal of consent (WoC) if their data was not deleted, lost to follow up or the primary endpoint completion date. The completion date is defined as a maximum follow up of 6 months (180 ± 10 days).

5.2 Secondary Variables

5.2.1 Component of the primary endpoint: all-cause mortality at 6 weeks after randomization

The efficacy variable is the occurrence of death from any cause at 6 weeks after randomization

5.2.2 Component of the primary endpoint: myocardial infarction at 6 weeks after randomization

The efficacy variable is the occurrence of MI at 6 weeks after randomization according to the following definition: Detection of MI is adapted from the Fourth Universal Definition of MI. The term acute myocardial infarction should be used when there is acute myocardial injury with clinical evidence of acute myocardial ischaemia and with detection of a rise and/or fall of cTn values with at least one value above the 99th percentile. High-sensitivity (hs)-cTn assays are recommended as preferred biomarkers.CK-MB (and CK) values will be assessed concurrently and used in the case that troponin values are not available.

5.2.3 Component of the primary endpoint: definite or probable stent thrombosis at 6 weeks after randomization

The efficacy variable is the occurrence of definite or probable stent thrombosis at 6 weeks after randomization according to the below definition:

- Definite stent thrombosis: is considered to have occurred by either angiographic or pathological confirmation.
- Probable stent thrombosis: regardless of the time after the index procedure, any myocardial infarction that is related to documented acute ischaemia in the territory of the implanted stent/scaffold without angiographic confirmation of stent thrombosis and in the absence of any other obvious cause.

The incidental angiographic documentation of stent occlusion in the absence of clinical signs or symptoms is not considered stent thrombosis.

5.2.4 Component of the primary endpoint: ischaemic stroke at 6 weeks after randomization

The efficacy variable is the occurrence of ischaemic stroke at 6 weeks after randomization according to the following definition: an acute episode of focal cerebral, spinal, or retinal dysfunction caused by infarction of central nervous system tissue. Haemorrhage may be a consequence of ischaemic stroke. In this situation, the stroke is an ischemic stroke with haemorrhagic transformation and not a haemorrhagic stroke.

5.2.5 Component of the primary endpoint: systemic thromboembolism at 6 weeks after randomization

The efficacy variable is the occurrence of systemic thromboembolism at 6 weeks after randomization according to the following definition: an arterial embolism resulting in clinical ischemia, excluding the central nervous system, coronary and pulmonary arterial circulation.

5.2.6 Cardiovascular mortality at 6 weeks after randomization

The efficacy variable is the occurrence of cardiovascular death at 6 weeks after randomization according to the following definition: death resulting from cardiovascular causes as per the following:

- Death caused by acute MI
- Death caused by sudden cardiac, including unwitnessed, death
- Death resulting from HF
- Death caused by stroke
- Death caused by cardiovascular procedures
- Death resulting from cardiovascular haemorrhage
- Death resulting from other cardiovascular cause

5.2.7 Bleeding (BARC type ≥ 2) at 6 weeks after randomization (superiority testing)

The safety variable is the occurrence of bleeding at 6 weeks after randomization according to the following definition: all bleeding complications occurring after randomization of the patient. Bleeding localization, amount of haemoglobin drop (if available) and the number of blood units transfused must be recorded in the Case Report Form according to Bleeding Academic Research Consortium Definition for Bleeding.

5.2.8 Urgent revascularization at 6 weeks after randomization

The efficacy secondary endpoint is the occurrence of urgent revascularization at 6 weeks after randomization according to the following definition: any PCI or bypass surgery performed for recurrent ischaemia that in the investigator's opinion cannot be delayed for more than 24 hours and is defined by the investigator as a non-elective.

5.2.9 All-cause mortality at 6 months after randomization

The efficacy variable is the occurrence of death from any cause at 6 months after randomization.

5.2.10 Unplanned hospitalization due to acute heart failure or acute coronary syndrome at 6 months after randomization

The efficacy variable is the occurrence of unplanned hospitalization due to acute HF or ACS at 6 months after randomization.

5.2.11 Ischaemic stroke at 6 months after randomization

The efficacy variable is the occurrence of ischaemic stroke at 6 months after randomization according to the following definition: an acute episode of focal cerebral, spinal, or retinal dysfunction caused by infarction of central nervous system tissue. Haemorrhage may be a consequence of ischaemic stroke. In this situation, the stroke is an ischemic stroke with haemorrhagic transformation and not a haemorrhagic stroke.

5.3 Additional Event Evaluation

Any bleeding events (BARC type ≥ 1) at 6 weeks after randomization.

6 Analysis Methods

6.1 General Principles

Multiplicity adjustment is needed for the primary endpoint analysis. Hierarchical testing will be considered as a specific multiplicity procedure. Type I error will be controlled at a one-sided 0.025 level for multiplicity across primary objectives. Statistical significance will be assessed in the order of the endpoints described below. If statistical significance is met for superiority of the primary efficacy endpoint (major ischaemic events) then testing will proceed with full alpha further down the hierarchy for non-inferiority testing of the primary safety endpoint (Bleeding type 2 or higher according to BARC criteria).

STEP 1: Test the superiority hypothesis for major ischaemic events, H_{01} ($\alpha = 0.025$, one-sided). If H_{01} is rejected, then proceed to STEP 2, otherwise testing will be stopped

STEP 2: Test the non-inferiority hypothesis for safety, H_{02} ($\alpha = 0.025$, one-sided).

If H_{02} hypotheses cannot be rejected, testing stops at that point. **Otherwise testing will proceed to the secondary endpoints**. The secondary outcomes are exploratory and the results will only be interpreted as supportive evidence related to the primary outcome.

6.1.1 Consort Diagram

A consort diagram describing patient flow with total screened patients and patients randomized will be included in the analysis. At the necessary levels, patients not included or excluded from the study will be explained. The diagram will present the number of patients crossing over (not receiving the experimental treatment although randomized to that arm or receiving the experimental treatment despite randomization to the control arm).

6.1.2 Stratification of Analyses

(ICH E3; 9.7.1, 11.4.2.4. ICH E9; 3.2)

The primary efficacy analysis of the multi-centre EPIDAURUS trial will be performed without stratifying for any variable. Centres will be represented in the primary analysis by a random effect (frailty).

6.1.3 Incomplete Dates

In statistical analysis, it is sometimes necessary to impute incomplete dates for data derivations. In such situations, the general rules for imputations are:

- If only the year of a date is given (YY), then the date shall be set to 'YY0531'.
- If only the year and month of a date is given (YYMM), then the date shall be set to 'YYMMxx', where xx is the last day of MM.

6.1.4 Estimand for primary Outcomes

ICH E9 (R)

To decide how to account for intercurrent events in intention-to-treat analyses, we will need to choose among several possible definitions of intention-to-treat effect i.e. causal Estimands.

Overview of the framework & what is an Estimand
This ICH E9 draft addendum (ICH E9 (R1) referred to as "addendum" presents a structured framework to link trial objectives to a suitable trial design and tools for estimation and hypothesis testing[13].

Population of interest
ITT population characterized through a comprehensive list of inclusion and exclusion criteria defined in section **2.2** and section **4.1**.

Endpoint of interest
Endpoint characterized by: The event of interest described in section **5.1**.

Intervention effect

- We will apply the "Treatment Policy" strategy.

Summary measure
Odds-Ratio (OR) and/or Hazard Ratio (HR) based on experimental and control will be used.

6.1.5 Hypothesis

The primary endpoint will be tested at the 2.5% one-sided significance level H_0**: OR [intensified: standard] ≥ 1** versus the alternative hypothesis H_1**: OR [intensified: standard] < 1**

The secondary endpoints included in confirmatory statistical testing using a closed testing procedure **Section 5.2** will be based on similar one-sided alternative hypotheses for the respective intervention difference.

6.1.6 Presentation of event analysis

A patient may have one or more events. For composite endpoints, the occurrence of the first event within the composite list will be used. For the analysis of each component of a composite, the occurrence of that component will be used, regardless of other events occurring earlier.

The steering committee is responsible for overseeing the good execution and administrative progress of the protocol. The committee meets regularly to monitor patient accrual, non-compliance with the protocol at individual centres, to act upon recommendations of the Data Safety Monitoring Board, and to determine the policy regarding individual publications arising from data generated from the performance of the trial.

The end-of-study was defined as the time of the last regular visit or phone call or letter for each patient. If none of these is available, 6 months (180 ± 10 days) will be used for end of study.

In general, summary tables of event analyses will include the number and percent of patients with an event per intervention group, event rate, RR with 95% confidence interval and p-value. Kaplan-Meier (KM) estimates of the cumulative proportion of patients with events will be calculated and plotted per treatment group, with the number of patients at risk indicated below the plot at specific time points. The KM plots will be presented for all time to event analyses, including the individual components of the composite endpoints.

6.2 Analysis Method

6.2.1 By-patient Presentations

Treatment group, age, sex and patient identifier will be present in all data listings.

6.2.2 Baseline Value

For each patient, the baseline values are obtained as part of the normal patient care and can be used for screening/baseline visit if collected within 72h prior to PCI.

The following baseline procedures were performed before randomization: demographics, general and cardiovascular medical history, current diseases, clinical parameters, laboratory parameters, concomitant medication. Baseline data will be presented by a randomised group. No p-values will be provided for the baseline data.

Table 3: characteristics of patients randomized to the experimental arm versus control arm

Characteristics	Experimental	Control
**Demographics**		
Age (years)		
Mean (standard deviation)		
Median (range)		
Male sex (No (%))		
Caucasian (No (%))		
Missing (No)		
**Clinical Parameters**		
Body mass index (BMI[2])		
Mean (standard deviation)		
Median (range)		
Missing (No)		
Systolic blood pressure (SBP) in mmHg		
Mean (standard deviation)		
Median (range)		
Missing (No)		
Diastolic blood pressure (DBP) in mmHg		
Mean (standard deviation)		
Median (range)		
Missing (No)		
Heart rate post PCI measured in pulse/min		
Mean (standard deviation)		
Median (range)		
Missing (No)		
ECG at admission		
ECG post PCI		
**Cardiovascular risk factors:**		
Smoking status		
Current Smoker (No (%))		
Ex-Smoker (No (%))		
Missing (No)		
Arterial hypertension		
no. (%)		
Missing (No)		
Diabetes mellitus		
no. (%)		
Missing (No)		
Hypercholesterolaemia		
no. (%)		
Missing (No)		
Family history of coronary artery disease		
no. (%)		
Missing (No)		
**General medical history**		
COPD (Gold)		
no. (%)		
Missing (No)		
Chronic renal failure (CRF)		

no. (%)	
Missing (No)	
Abnormal liver function	
no. (%)	
Missing (No)	
Cancer	
no. (%)	
Missing (No)	
Alcohol abuse	
no. (%)	
Missing (No)	
**Cardiovascular medical history**	
Myocardial infarction	
no. (%)	
Missing (No)	
PCI	
no. (%)	
Missing (No)	
CABG	
no. (%)	
Missing (No)	
Stroke	
no. (%)	
Missing (No)	
Stent thrombosis	
no. (%)	
Missing (No)	
Bleeding BARC ≥ 2[1]	
no. (%)	
Missing (No)	
Peripheral arterial occlusive disease (PAOD)	
no. (%)	
Missing (No)	
Cerebrovascular disease (CVD)	
no. (%)	
Missing (No)	
Atrial fibrillation	
no. (%)	
Missing (No)	
Aortic stenosis	
no. (%)	
Missing (No)	
Aortic insufficiency	
no. (%)	
Missing (No)	
Mitral insufficiency	
no. (%)	
Missing (No)	
Mitral stenosis	
no. (%)	
Missing (No)	

Tricuspid insufficiency	
no. (%)	
Missing (No)	
Pacemaker/ICD/CRT	
no. (%)	
Missing (No)	
**Symptoms**	
Angina pectoris (CCS classification)	
no. (%)	
Missing (No)	
Dyspnea (NYHA classification)	
no. (%)	
Missing (No)	
Syncope	
no. (%)	
Missing (No)	
**Concomitant medication— no. (%)**	
Aspirin (No (%))	
Yes	
No	
Missing (No)	
P2Y12 Inhibitors	
Yes	
No	
Missing (No)	
Oral Anti-coagulant	
Yes	
No	
Missing (No)	
Aldosterone antagonist	
Yes	
No	
Missing (No)	
ACE inhibitor	
Yes	
No	
Missing (No)	
AT2 receptor blocker	
Yes	
No	
Missing (No)	
Beta blocker	
Yes	
No	
Missing (No)	
Aldosterone antagonist	
Yes	
No	
Missing (No)	
Angiotensin-receptor neprilysin inhibitor	
Yes	

No	
Missing (No)	
SGLT2 inhibitor	
Yes	
No	
Missing (No)	

6.2.3 Descriptive Summaries of Continuous Variables

Summaries for continuous variables will present absolute values and changes from baseline, for each treatment group. They will include mean, SD, median and IQR.

6.2.4 Descriptive summaries of categorical variables

Summaries of categorical variables will provide frequencies and percentage for each treatment group.

6.2.5 Eligibility

The number of patients needed to be screened for eligibility is not known at the time of creation of this version of the SAP. Patients not fulfilling any of the eligibility criteria will be presented with their corresponding pat ID, reasons of ineligibility, last visit and whether randomized or not. The number of ineligible patients randomised, if any, will be reported along with reasons for ineligibility.

6.2.6 Withdrawal/Follow-up

Patients must be assured that they may withdraw from the trial at any time for any reason and receive alternative conventional therapy as indicated. For individuals who withdraw their consent, the decision to maintain or delete their data until the point of withdrawal of consent is at the discretion of the principal investigator and will be handled on a case-case basis as deemed appropriate.

Patients will be also withdrawn from the trial/treatment for the following reasons:

- withdrawal of consent by the subject for any reason at any time
- if continued trial participation is not in the best interest of the subject, as judged by the investigator (e.g. due to clinical adverse event, laboratory abnormality or inter-current illness)
- termination of the trial by regulatory authorities or ethics committees
- patients, who become prisoners, involuntarily incarcerated or compulsorily detained for treatment of either a psychiatric of physical illness

The number and percent of patients who completed the study and those who discontinued from the study and reasons for discontinuation will be summarized by treatment group and overall, for all randomized patients. Listings of patients who prematurely discontinued the study will be provided.

The number and percent of patients who discontinued randomized intervention and reasons for discontinuation will be summarized by treatment group and overall, for all randomized patients. Listings of patients who prematurely and permanently discontinued treatment and for whom post-discontinuation data are excluded from the on-treatment population will be provided.

The subject disposition table will be presented for all subjects by intervention group. The table will summarize the percentage of enrollment, withdrawal at each stage and study completion by intervention group for all subjects.

6.2.7 Analysis of the primary efficacy Variable

The EPIDAURUS trial aims to determine whether intensified regimen is superior to standard regimen regarding the number of major ischaemic events at 6 weeks after randomization. As the duration of intervention is specified to 4 weeks, the Principal Investigator anticipates that the maximum effect of treatment would probably occur during the pre-specified 6 weeks' assessment period i.e. between randomization to day 42 post-randomization. The Superiority of intensified regimen versus standard regimen is to be claimed based on the number of major ischaemic events reported by the investigators and confirmed by the Event Adjudication Committee (EAC) to determine whether they meet the specified criteria of endpoint definition in order to provide a standardized, systematic, independent and unbiased assessment

The primary variable is the dichotomized yes/no variable indicating the presence or absence of major ischaemic events (*defined as any of the following: all-cause mortality, myocardial infarction, definite or probable stent thrombosis, ischaemic stroke or systemic thromboembolism*) in the two treatment arms at 6 weeks after randomization. As stated in the protocol, superiority can be claimed based on the number of major ischaemic events.

Data Layout:

- The variable ID is the number that identifies each individual.
- The variable randomization day indicates the day which randomization took place.
- The variable day indicates which day the outcome measurement was taken. This variable is used to provide order for the data within a cluster as in longitudinal studies
- Independent variable: Treatment

By applying the Estimand framework & treatment policy, Loss to follow up and withdrawal of consent will be all censored.

Modelling Approach:

Logistic Model with RE will be applied.

Mixed Model with 2 levels;

- Y_{ij}~Binomial (1, π_{ij})	Binary Outcome	
- logit (Y_{ij}=1) =	*Outcome of individual i*	*level 1*
β_0 +	*Intercept*	
β_1 treatment$_{ij}$+	*Treatment of individual i*	
β_2 Center$_j$+	*Covariate of center j*	*level 2*
μ_{0j}+	*Random Intercept*	

μ_{1j}*Center_j *Random slope*

```
model <- glmer (ischemic events ~ treatment +
  (1 | centre), data = epidaurus, family = binomial)
```

Confidence Interval and P-values

All statistical methods will be based on the International Conference on Harmonization (ICH-E9) document "Statistical Principles for Clinical Trials". All applicable statistical tests will be 2-sided and will be performed using a 5% significance level. Confidence intervals presented will be 95% and two-sided. The superiority of the intensified regimen can be claimed based on the number of major ischaemic events. Estimates of interventions will be accompanied by confidence intervals. P-value < 0.05 is to be considered statistically significant. P-values will be rounded to three decimal places. P-values less than 0.001 will be reported as < 0.001 in tables. P-values greater than 0.999 will be reported as > 0.999.

Kaplan-Maier Curves:

In the analysis of the primary composite endpoint, cumulative hazard curves will be modelled with the use of the Kaplan–Meier method and will be compared with the use of a log-rank test and/or Cox proportional-hazards model.

For composite endpoints, the time to the first event within the composite list will be used. For each component of a composite endpoint, the time to first component event will be used, regardless of other events occurring earlier. In general, summary tables of time-to-event analyses will include the number and percent of patients with an event per study group, event rate, HR with 95% confidence interval and p-value. The event rate will be derived as the number of patients with the event divided by the total duration of follow-up across all patients in a given group, presented as patients with an event per 100 patient-years. Kaplan- Meier (KM) estimates of the cumulative proportion of patients with events will be calculated and plotted per study group, with the number of patients at risk indicated below the plot at specific time points. The KM plots will be presented for all time to event analyses

6.2.8 Secondary Endpoints

All secondary endpoints will be analyzed using Cox proportional-hazards model. As the analysis of secondary endpoints is explorative only fixed effect models will be used without introduction of random effects for the participating centres.

<u>Important assumptions</u>: Censoring is uninformative.

Modelling Approach:

Cox proportional hazards regression model will be applied:

$h(t) = h_0(t) * \exp(b1 * X1)$, where X1 = study group (digital = 1 vs. conventional = 0) and h(t) is the expected hazard at time t, h0(t) is the baseline hazard.

```
package(survival)

cox_model <- coxph (Surv(time2primaryendpoint,primary endpoint) ~ study group, data = epidaurus, contr
ol = coxph.control(timefix = FALSE))
```

The PH assumption will be tested by calculating the correlation coefficient between the scaled Schoenfeld residuals and time and plotting them over time

```
package(survival)

residuals <- cox.zph (cox_model)

plot(residuals)
```

In the case of violation of the PH assumption, logistic regression analysis will be applied:

$Y_i \sim Bin(1, \pi_i)$

$logit(\pi_i) = \beta_0 + \beta_1 * X_i$

```
logit_model <- glm (primary endpoint ~ study group, data = epidaurus, family = binomial)
```

6.2.9 Subgroup Analysis

(ICH E3; 9.7.1, 11.4.2.1. ICH E9; 5.7)

Subgroup analyses to evaluate variation in treatment effect will be performed based on tests for interaction using the Cox regression fixed effect models. A test of interaction between randomized treatment group and the subgroup variable will be performed in each fitted model, including the relevant subgroup variable and the interaction between treatment and the subgroup variable.

The following subgroups will be examined:

- Age (Cut-off ≥/< 65)
- Gender
- STEMI or NSTEMI
- AF (paroxysmal or not)
- Apixaban, Edoxaban, Rivaroxaban
- Ticagrelor, Prasugrel
- On treatment with P2Y12-inhibitor
- On treatment with Aspirin (≤ 7 days or > 7 days)
- On treatment with OAC
- Multivessel disease yes or no
- Bifurcation, ostial, 3-vessel PCI
- Peripheral arterial occlusive disease (PAOD) or cerebrovascular disease (CVD)
- CHA2DS2-VASc ≥ 3

- HAS-BLED ≥ 3
- Previous stroke
- Previous MI
- NYHA > II
- BNP cut-off median
- eGFR < 60 mL/min/1.73m2
- Max CK > 1000 U/L during the first 48h after the index myocardial infarction
- Increased PRD or decreased DC

In addition to the number and percent of patients with an event, event rate estimate, HR with 95% confidence interval and p-value for each subgroup. The p-values for the subgroup analyses and interaction will not be adjusted for multiple comparisons as the tests are exploratory and will be interpreted descriptively.

6.2.10 Sensitivity analysis of the primary efficacy and safety endpoint

Sensitivity Analysis 1:
All endpoints will be analyzed using the ITT, mITT and as-treated principle as sensitivity analysis.

Sensitivity Analysis 2:
Cox proportional-hazards model including a random effect for centres (frailty statement) will be applied as sensitivity analysis.

6.2.11 Additional Analysis

The association between bleeding BARC ≥2 and subsequent ischaemic complications will be calculated using a Cox regression analysis with the introduction of BARC ≥ 2 as a time-dependent co-variate. For this analysis, all ischaemic events that occurred before the bleeding event will be ignored, and only new events that occurred after the bleeding event will be considered.

6.2.12 Analysis of Safety

Safety endpoints will be analysed in a modified intention to treat (mITT) population according to information provided in Table 2.

7 Statistical Software

The study database was stored in Excelya-Server, version 1.0.7. Statistical analyses will be performed using R-Studio, version 1.0.143, and R statistical computing system, version 4.2.2 or newer. All statistical tests are two-sided, and $P < 0.05$ will be considered significant. Logistic regression analyses will be performed using the lme4 package or newer. Survival analyses will be performed using the survival package or newer.

References

1. Gamble C, Krishan A, Stocken D, et al. Guidelines for the Content of Statistical Analysis Plans in Clinical Trials. *Jama* 2017;**318**:2337–2343.

2. Valgimigli M, Bueno H, Byrne RA, et al. 2017 ESC focused update on dual antiplatelet therapy in coronary artery disease developed in collaboration with EACTS: The Task Force for dual antiplatelet therapy in coronary artery disease of the European Society of Cardiology (ESC) and of the European Association for Cardio-Thoracic Surgery (EACTS). *Eur Heart J* 2017;**39**:213–260.

3. Neumann F-J, Sousa-Uva M, Ahlsson A, et al. 2018 ESC/EACTS Guidelines on myocardial revascularization. *Eur Heart J* 2018;**40**:87–165.

4. Vranckx P, Valgimigli M, Eckardt L, et al. Edoxaban-based versus vitamin K antagonist-based antithrombotic regimen after successful coronary stenting in patients with atrial fibrillation (ENTRUST-AF PCI): a randomised, open-label, phase 3b trial. *Lancet Lond Engl* 2019.

5. Lopes RD, Elliott LE, White HD, Hochman JS, Werf FV de, Ardissino D, Nielsen TT, Weaver WD, Widimsky P, Armstrong PW, Granger CB. Antithrombotic therapy and outcomes of patients with atrial fibrillation following primary percutaneous coronary intervention: results from the APEX-AMI trial. *Eur Heart J* 2009;**30**:2019–2028.

6. Cannon CP, Bhatt DL, Oldgren J, et al. Dual Antithrombotic Therapy with Dabigatran after PCI in Atrial Fibrillation. *New Engl J Medicine* 2017;**377**:1513–1524.

7. Gibson CM, Mehran R, Bode C, et al. Prevention of Bleeding in Patients with Atrial Fibrillation Undergoing PCI. *The New England Journal of Medicine* 2016;**375**:2423 2434.

8. Collet J-P, Thiele H, Barbato E, et al. 2020 ESC Guidelines for the management of acute coronary syndromes in patients presenting without persistent ST-segment elevationThe Task Force for the management of acute coronary syndromes in patients presenting without persistent ST-segment elevation of the European Society of Cardiology (ESC). *Eur Heart J* 2020:ehaa575.

9. Gargiulo G, Goette A, Tijssen J, Eckardt L, Lewalter T, Vranckx P, Valgimigli M. Safety and efficacy outcomes of double vs. triple antithrombotic therapy in patients with atrial fibrillation following

percutaneous coronary intervention: a systematic review and meta-analysis of non-vitamin K antagonist oral anticoagulant-based randomized clinical trials. *Eur Heart J* 2019.

10. Lopes RD, Leonardi S, Wojdyla DM, et al. Stent Thrombosis in Patients with Atrial Fibrillation Undergoing Coronary Stenting in the AUGUSTUS Trial. *Circulation* 2019;**141**:781–783.

11. Sibbing D, Aradi D, Jacobshagen C, et al. Guided de-escalation of antiplatelet treatment in patients with acute coronary syndrome undergoing percutaneous coronary intervention (TROPICAL-ACS): a randomised, open-label, multicentre trial. *Lancet* 2017;**390**:1747–1757.

12. Antman EM, Wiviott SD, Murphy SA, Voitk J, Hasin Y, Widimsky P, Chandna H, Macias W, McCabe CH, Braunwald E. Early and Late Benefits of Prasugrel in Patients With Acute Coronary Syndromes Undergoing Percutaneous Coronary Intervention A TRITON–TIMI 38 (TRial to Assess Improvement in Therapeutic Outcomes by Optimizing Platelet InhibitioN with Prasugrel–Thrombolysis In Myocardial Infarction) Analysis. *J Am Coll Cardiol* 2008;**51**:2028–2033.

13. Rufibach K. Treatment effect quantification for time-to-event endpoints–Estimands, analysis strategies, and beyond. *Pharm Stat* 2019;**18**:145–165.

ICH Harmonised Tripartite Guideline. Statistical principles for clinical trials. International Conference on Harmonisation E9 Expert Working Group. Stat Med. 1999;18(15):1905-42.

ICH Harmonised Tripartite Guideline: Structure and content of clinical study reports E3. Step 4. 1995. https://database.ich.org/sites/default/files/E3_Guideline.pdf. Accessed 1 Dec 2022.

ICH Harmonised Tripartite Guideline: Integrated addendum to ICH E6(R1): Guideline for good clinical practice E6(R2). Current Step 4. 2016. https://database.ich.org/sites/default/files/E6_R2_Addendum.pdf
ICH Harmonised Guideline addendum on estimands and sensitivity analysis in clinical trials to the guideline on statistical principles for clinical trials e9(R1). Final Version. 2019. Https://Database.Ich.Org/Sites/Default/Files/E9-R1_Step4_Guideline_2019_1203.Pdf
